# Synthesis, Structure Elucidation, Antibacterial Activities, and Synergistic Effects of Novel Juglone and Naphthazarin Derivatives Against Clinical Methicillin-Resistant *Staphylococcus aureus* Strains

**DOI:** 10.3389/fchem.2021.773981

**Published:** 2021-11-19

**Authors:** Valentin Duvauchelle, Chaimae Majdi, David Bénimélis, Catherine Dunyach-Remy, Patrick Meffre, Zohra Benfodda

**Affiliations:** ^1^ UNIV. NIMES, UPR CHROME, Nîmes, France; ^2^ VBIC, INSERM U1047, Service de Microbiologie et Hygiène Hospitalière, Université de Montpellier, CHU Nîmes, Nîmes, France

**Keywords:** juglone, naphthazarin, antibacterial, clinical MRSA, synergistic studies, MIC determination, structure elucidation

## Abstract

Infections caused by drug-resistant bacteria are a serious threat to human and global public health. Moreover, in recent years, very few antibiotics have been discovered and developed by pharmaceutical companies. Therefore, there is an urgent need to discover and develop new antibacterial agents to combat multidrug-resistant bacteria. In this study, two novel series of juglone/naphthazarin derivatives (43 compounds) were synthesized and evaluated for their antibacterial properties against various clinical and reference Gram-positive MSSA, clinical Gram-positive MRSA, and clinical and reference Gram-negative bacteria *E. coli* and *P. aeruginosa*. These strains are of clinical importance because they belong to ESKAPE pathogens. Compounds **3al**, **5ag**, and **3bg** showed promising activity against clinical and reference MSSA (MIC: 1–8 µg/ml) and good efficacy against clinical MRSA (MIC: 2–8 µg/ml) strains. **5am** and **3bm** demonstrated better activity on both MSSA (MIC: 0.5 µg/ml) and MRSA (MIC: 2 µg/ml) strains. Their MICs were similar to those of cloxacillin against clinical MRSA strains. The synergistic effects of active compounds **3al**, **5ag**, **5am**, **3bg**, and **3bm** were evaluated with reference antibiotics, and it was found that the antibiotic combination with **3bm** efficiently enhanced the antimicrobial activity. Compound **3bm** was found to restore the sensitivity of clinical MRSA to cloxacillin and enhanced the antibacterial activity of vancomycin when they were added together. In the presence of **3bm**, the MIC values of vancomycin and cloxacillin were lowered up to 1/16th of the original MIC with an FIC index of 0.313. Moreover, compounds **3al**, **5ag**, **5am**, **3bg**, and **3bm** did not present hemolytic activity on sheep red blood cells. *In silico* prediction of ADME profile parameter results for **3bm** is promising and encouraging for further development.

## Introduction

The introduction of antibiotics into clinical use was the major therapeutic advance of the 20th century (Trémolières, 2010). Antibiotics are the main treatment for bacterial infections and made possible many modern medical healthcare such as for people with cancers or organ transplants. However, extensive use and misuse of antibiotics in humans, animals, and plants combined with a diminished lack of interest by the pharmaceutical industry has led to the rapid rise of antimicrobial resistance (AMR) (Jabes, 2011; Stanton, 2013).

Unfortunately, the development of new drugs in clinical research is low; only two new classes of antibiotics: oxazolidinones (2000) and lipopeptides (2003) were introduced during the last decade (WHO Antibiotic resistance: No action today, no cure tomorrow, 2011). In addition, accumulation of antibiotics in the environment may be the result of dissemination of resistant bacteria with antibiotic resistance genes (Tan and Shuai, 2015). AMR infections are recognized by the World Health Organization (WHO) as a global concern for public health (WHO | Prioritization of pathogens to guide discovery, research and development of new antibiotics for drug resistant bacterial infections, including tuberculosis, 2017). Bacterial resistance is responsible for approximately 700,000 death annually and will likely increase the death rate in the next few years, and it will become the first leading cause of mortality in the world in 2050 (de Kraker et al., 2016; O’Neil, 2016). Increasing resistance to conventional drugs by strains such as methicillin-resistant *Staphylococcus aureus* (MRSA), *Pseudomonas aeruginosa* (*P. aeruginosa*), and *Escherichia coli* (*E. coli*) is pushing us to find new classes of antibiotics to circumvent multidrug-resistant infections. These pathogens belonging to the ESKAPE group are a priority for the development of new compounds with antibacterial properties (Mulani et al., 2019).

**FIGURE 1:**
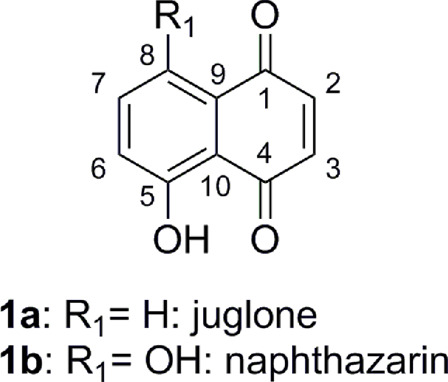
Structures of juglone and naphthazarin.

The development of novel and structurally diverse compounds with potential antimicrobial properties is highly needed. Naphthoquinones are an important class of quinones found in many synthetic and natural products and are known to possess various functions and biological applications (Pinho et al., 2012). Indeed, natural or synthetic 1,4-naphthoquinone derivatives exhibited strong action as antimalarial, anticancer, anti-inflammatory, antifungal, and antibacterial agents (Fernando and Vitor, 2016; Aminin and Polonik, 2020). Naphthoquinones exert their biological properties *via* several mechanisms such as the production of reactive oxygen species (ROS). The 1,4-naphthoquinone moiety is present in many alkaloids such as lawsone, plumbagin, juglone, and naphthazarin. Juglone (5-hydroxy-1,4-naphthoquinone, Figure 1a) is present in Juglandaceae species, and naphthazarin (5,8-dihydroxy-1,4-naphthoquinone, Figure 1b) is derived from the tissues of Boraginaceae, Droseraceae, and Nepenthaceae families (Papageorgiou et al., 1999; Thakur, 2011; Devi et al., 2016). Previously, natural or synthetic juglone/naphthazarin derivatives exhibited antibacterial activity on various bacterial strains (minimal inhibitory concentration (MIC) values ranging from 0.78 to 100 μg/ml) (Clark et al., 1990; Shen et al., 2002; Jeon et al., 2009; Yakubovskaya et al., 2009; Sánchez-Calvo et al., 2016; Wang et al., 2016). However, determination of the MIC following a broth dilution assay on both reference and clinical bacterial strains of *S. aureus* and *E. coli* isolated from patients in a university hospital is an important part in this study. It is for the first time that 1,4-naphthoquinone derivatives are evaluated on clinical bacterial strains. Furthermore, evaluation of the adjuvant effect of the synthesized molecules on clinical resistant bacteria revealed potential restoration of activity between newly synthesized compounds and reference antibiotics. These evaluations against resistant bacteria, known to cause therapeutic problems in hospitals, are less reported in the literature.

With the aim of seeking further antibacterial active substances, we report the synthesis of two classes of 1,4-naphthoquinone derivatives (juglone and naphthazarin derivatives) and the X-ray structural determination of two compounds. The antimicrobial profiles of the compounds against clinical sensitive and clinical resistant Gram-positive and Gram-negative bacteria are evaluated. Additionally, their synergistic effects in combination with two conventional antibiotics cloxacillin and vancomycin were investigated. Finally, we also determined the *in vitro* hemolytic activity of the most active compounds and the *in silico* ADME parameters of all compounds.

## Results and Discussion

### Chemistry

**SCHEME 1:**
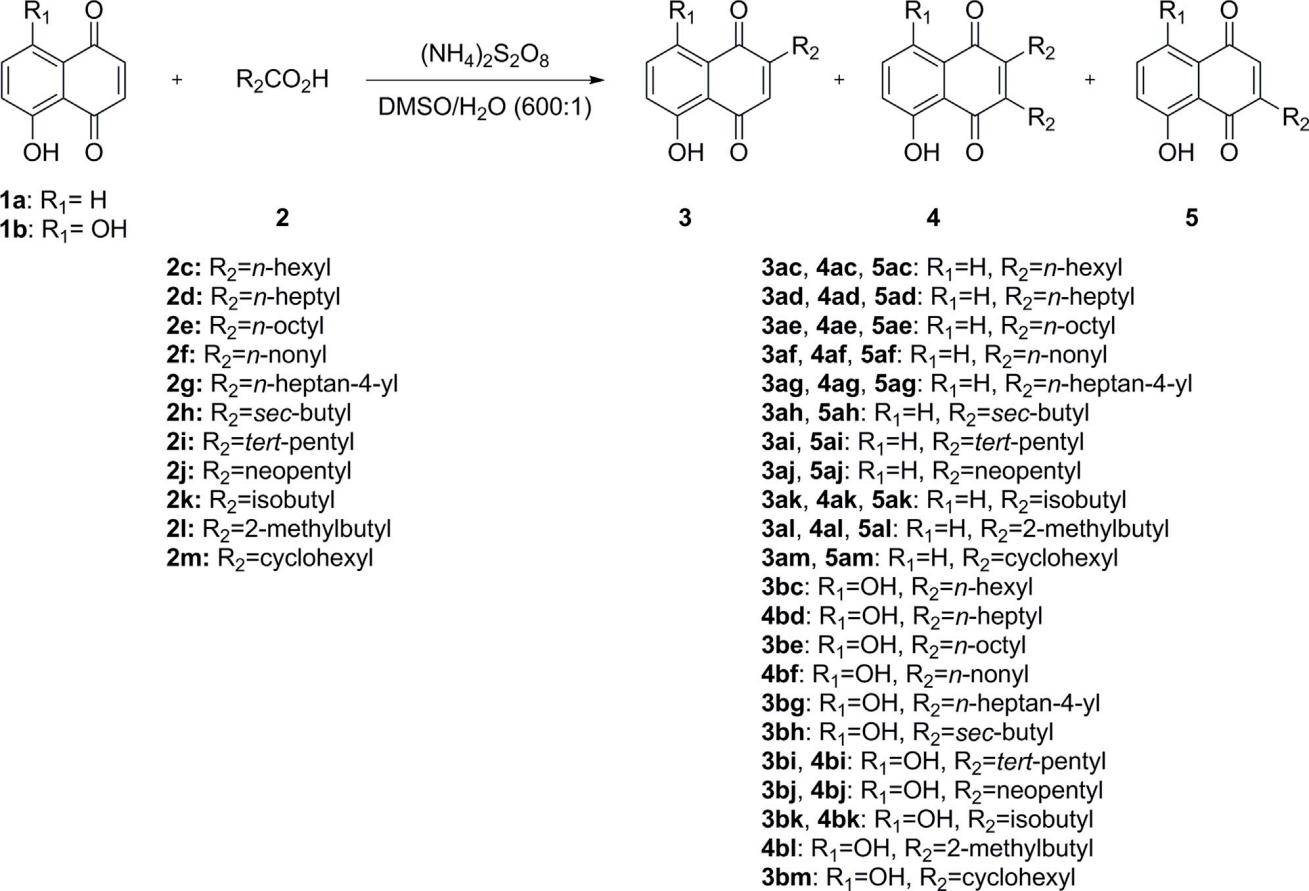
Synthesis of juglone and naphthazarin derivatives (**3**, **4**, and **5**).

The synthesis of juglone and naphthazarin derivatives **3**, **4**, and **5** was accomplished using Minisci-type direct C–H alkylation, as illustrated in Scheme 1 (Sutherland et al., 2018). Commercially, juglone (**1a**) and naphthazarin (**1b**) react with 11 commercially available carboxylic acids **(2)** in the presence of ammonium persulfate to afford 1, 2, or 3 different products with one or two alkylated chains in different positions (**3**, **4**, and **5**) in low to moderate yields (Table 1). Starting from juglone (**1a**), two monoalkylated juglone derivatives (**3** and **5**) and a dialkylated juglone derivative **(4)** were obtained in most cases. Starting from naphthazarin (**1b**), one monoalkylated naphthazarin derivative **(3)** and the dialkylated naphthazarin derivative **(4)** were obtained in most cases. Indeed, for some carboxylic acids, we observed the formation of only one monoalkylated naphthazarin derivative (**3bc**, **3bd**, **3be**, **3bg**, **3bh**, and **3bm**), and for other carboxylic acids, the dialkylated naphthazarin derivatives (**4bf** and **4bl**) were the sole products.

**TABLE 1:** Chemical structure and yields of synthesized compounds.

R1	R2	Cpd	Yield^a^ (%)	Cpd	Yield^a^ (%)	Cpd	Yield^a^ (%)	Total yield (%)
H	*n*-Hexyl	**3ac**	10	**4ac**	2	**5ac**	13	25
H	*n*-Heptyl	**3ad**	10	**4ad**	7	**5ad**	7	24
H	*n*-Octyl	**3ae**	10	**4ae**	7	**5ae**	8	25
H	*n*-Nonyl	**3af**	18	**4af**	5	**5af**	12	35
H	*n*-Heptan-4-yl	**3ag**	19	**4ag**	2	**5ag**	17	38
H	*Sec*-butyl	**3ah**	14	**4ah**	N.O^b^	**5ah**	8	22
H	*Tert*-pentyl	**3ai**	9	**4ai**	N.O^b^	**5ai**	8	17
H	Neopentyl	**3a**j	19	**4aj**	N.O^b^	**5aj**	5	24
H	Isobutyl	**3ak**	9	**4ak**	4	**5ak**	6	19
H	2-Methylbutyl	**3al**	22	**4al**	12	**5al**	11	45
H	Cyclohexyl	**3am**	24	**4am**	N.O^b^	**5am**	8	32

OH	*n*-Hexyl	**3bc**	22	**4bc**	N.O^b^			22
OH	*n*-Heptyl	**3bd**	N.O^b^	**4bd**	9			9
OH	*n*-Octyl	**3be**	40	**4be**	N.O^b^			40
OH	*n*-Nonyl	**3bf**	N.O^b^	**4bf**	6			6
OH	*n*-Heptan-4-yl	**3bg**	23	**4bg**	N.O^b^			23
OH	*Sec*-butyl	**3bh**	42	**4bh**	N.O^b^			42
OH	*Tert*-pentyl	**3bi**	3	**4bi**	11			14
OH	Neopentyl	**3bj**	8	**4bj**	6			14
OH	Isobutyl	**3bk**	16	**4bk**	3			19
OH	2-Methylbutyl	**3bl**	N.O^b^	**4bl**	11			11
OH	Cyclohexyl	**3bm**	33	**4bm**	N.O^b^			33

a Yields obtained after purification (flash chromatography).

b N.O.: not obtained.

The low yields of each compound (**3**, **4**, and **5**) obtained during this reaction are due, on the one hand, to the formation of secondary products (not isolated) and, on the other hand, to the tricky purification of the products (Table 1). Although Sutherland et al. (2018) described the synthesis of **3ac**, **4ac**, and **5ac** with good yields, we did not obtain the same yields. In addition to their studies, we were able to characterize each regioisomer (**3** or **5**) using NMR spectroscopy, in particular heteronuclear multiple bond correlation (HMBC), and X-ray diffraction techniques (see the Supporting Information). The final compounds were fully characterized by ^1^H nuclear magnetic resonance (NMR), ^13^C NMR, HMBC, and infrared (IR) spectroscopy; high-performance liquid chromatography (HPLC); and high-resolution mass spectrometry (HRMS). The spectroscopic data of the compounds are consistent with the assigned structures (see the Experimental Section and the Supporting Information).

### X-Ray Crystallographic Analysis

Two X-ray crystal structures were used to establish the authenticity of the basic structures. This analysis was performed on compounds **5ae** and **3bg** that were obtained from juglone and naphthazarin, respectively, and X-ray and structure refinement statistics are presented in Table 2.

**TABLE 2:** Crystal data and structure refinement details for **5ae** and **3bg**.

Identification code	**5ae**	**3bg**
Formula	C_18_H_22_O_3_	C_17_H_20_O_4_
M.W. (g.mol^−1^)	286.37	288.34
Crystal system, space group	Monoclinic, *P*21/*c*	Monoclinic, C 2/c
*d*calcd (g.cm^−3^)	1.245	1.311
Temperature (K)	123	123
a, b, c (Å)	5.2708 (5), 6.7965 (5), 21.391 (2)	15.2266 (10), 7.6965 (5), 25.8898 (14)
α (°)	90	90
β (°)	94.820 (4)	105.615 (2)
γ (°)	90	90
V (Å^3^)	763.58	2922.08
Z	2	8
Radiation type	Mo *Kα*	Mo *Kα*
*μ* (mm^−1^)	0.08	0.09
Crystal size (mm)	0.25 × 0.10 × 0.01	0.12 × 0.05 × 0.03
*R*int	0.052	0.051
(sin *θ*/*λ*)max (Å^−1^)	0.685	0.617
*R*[*F*2 > 2*σ*(*F*2)], *wR*(*F*2), *S*	0.053, 0.067, 1.59	0.040, 0.102, 1.07
Δ*ρ*max, Δ*ρ*min (e Å^−3^)	0.20, −0.20	0.25, −0.18
Packing coefficient	0.697	0.707
F0000	308	1232
CCDC number	2083159	2083158

**FIGURE 2:**
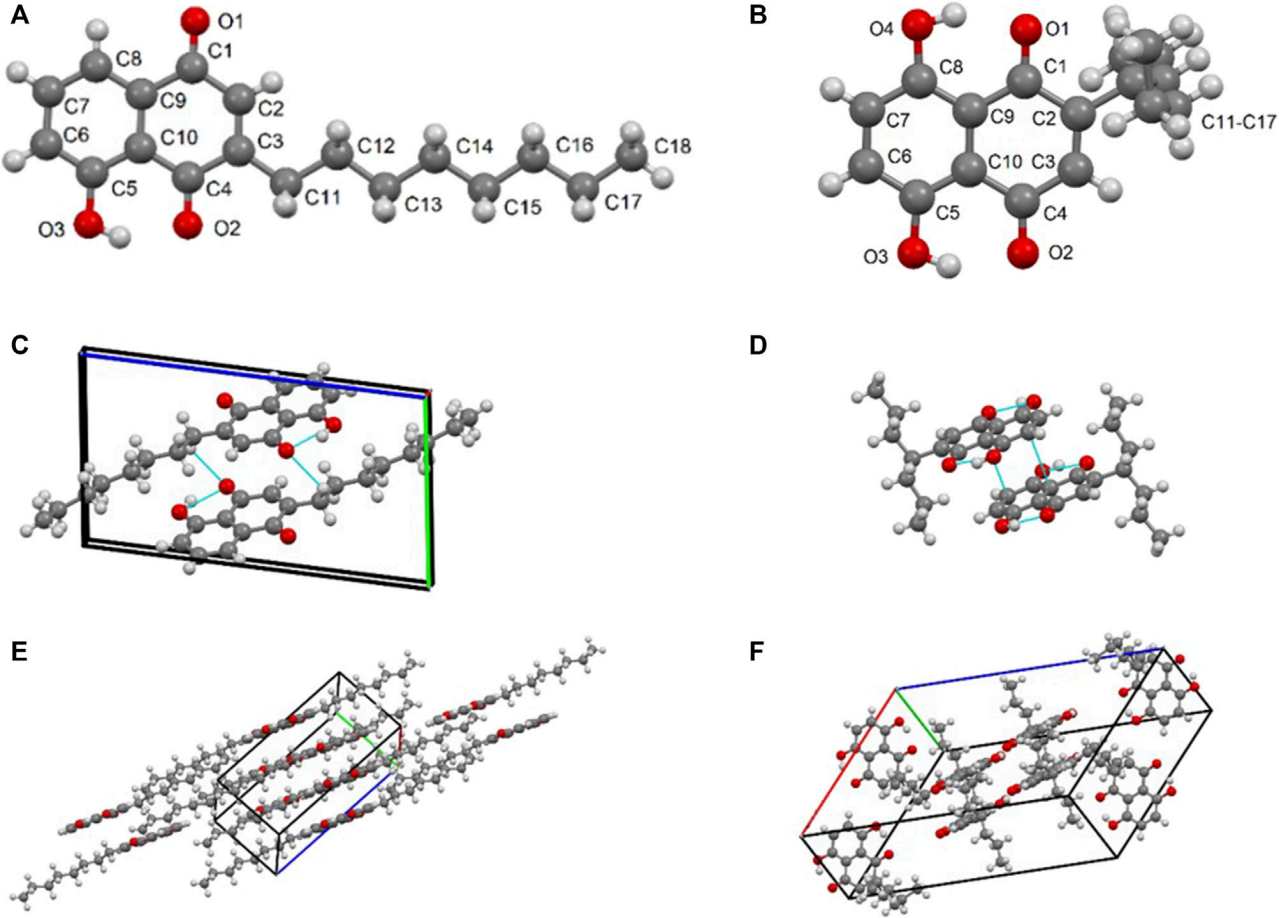
**(A)** XP diagram of compound **5ae** with atomic numbering scheme; **(B)** XP diagram of compound **3bg** with atomic numbering scheme; **(C)** dimeric unit formation of **5ae** through intra- and intermolecular hydrogen bonds; **(D)** dimeric unit formation of **3bg** through intra- and intermolecular hydrogen bonds; **(E)** representation of the packing diagram of compound **5ae** within the crystal mesh; and **(F)** representation of the packing diagram of compound **3bg** within the crystal mesh.

An orange plate-like crystal of compound **5ae** (C_18_H_22_O_3_) with an approximate dimension of 0.25 × 0.10 × 0.01 mm and a black plate-like specimen of compound **3bg** with an approximate dimension of 0.12 × 0.05 × 0.03 mm were used for X-ray diffraction. Compound **5ae** was crystallized in the monoclinic crystal system using the space group P 2_1_, and **3bg** was crystallized in the monoclinic crystal system using the space group C 2/c. For **5ae**, we observed that the structure is completely planar with the *n-*octyl substituent staying linear in the mesh. A very strong intramolecular H-bond is observed between the hydroxyl and the ketone group [O_3_-H_3_ ••• O_2_ 1.801 Å and 147°] (Figures 2A–C). Short contacts are apparent between two molecules in the solid state between mostly oxygen atoms from quinone and naphthol groups and aromatic hydrogens [C_12_-H_12_B ••• C5 2.862 Å and 137°; C_11_-H_11_B ••• C_5_ 2.706 Å and 133°; C_2_-H_2_ ••• O_3_ 2.582 Å and 172°; C_6_-H_6_ ••• O_1_ 2.664 Å and 171°; C_7_-H_7_ ••• O_1_ 2.665 Å and 121°; and C_8_-H_8_ ••• O_1_ 2.604 Å and 124°] (Figures 2C–E). For **3bg**, the structure is nonplanar with the 4-heptyl substituent almost perpendicular to the aromatic moiety in the mesh (91° for C_2_-C_11_-C_13_). Two very strong intramolecular H-bonds are observed between the hydroxyl and ketone groups [O_3_-H_3_ ••• O_2_ 1.781 Å and 149°; O_4_-H_4_ ••• O_1_ 1.734 Å and 152°] (Figures 2B–D). Short contacts are apparent between two molecules in the solid state [C_3_-H_3_A ••• O_4_ 2.523 Å and 171°; C_7_-H_7_ ••• O_2_ 2.604 Å and 170°; and C_13_-H_13_A ••• C_13_-H_13_A 2.320 Å and 171°] (Figures 2D–F). Finally, interactions between the two aromatic rings could be attributed to π-π stacking [C_9_ ••• C_7_ 3.397 Å] (Figures 2D–F).

### *In Vitro* Antibacterial Activity

In this work, we studied the antibacterial activity of our series of compounds derived from juglone and naphthazarin. To our knowledge, none of the newly synthesized molecules has been evaluated for their antibacterial properties against sensitive and resistant clinical strains of *S. aureus.*

#### Antibiotic Susceptibility Testing Against Reference and Sensitive Clinical Strains of *E. coli*, *S. aureus*, and *P. aeruginosa*

The newly synthesized juglone and naphthazarin derivatives **3**, **4**, and **5** were subjected to antibiotic susceptibility testing against reference and sensitive clinical strains of *E. coli*: ATCC 25922 and NEC-S1; *S. aureus*: ATCC 29213 and NSASM-1; and *P. aeruginosa*: PAO1. MICs against Gram-positive and Gram-negative strains were determined by using the broth microdilution assay. These strains are known to be sensitive to reference antibiotics: Cefotaxime is the reference antibiotic to treat sensitive Gram-negative bacteria, and cloxacillin and vancomycin are reference antibiotics to treat sensitive Gram-positive strains (The European Committee on Antimicrobial Susceptibility Testing, 2021). These compounds **3**, **4**, and **5** were then evaluated against different bacterial strains to determine their antibacterial activities (Table 3).

**TABLE 3:** MIC values (μg/ml) of the tested compounds against the Gram-positive and Gram-negative pathogen panel.

Compound	MICs (μg/ml)				
	Gram-positive bacteria		Gram-negative bacteria		
	*S. aureus* ATCC 29213^a^	*S. aureus* NSASM-1^b^	*E. coli* ATCC25922^a^	*E. coli* NEC-S1^b^	*P. aeruginosa* PAO1^a^
**3ac**	128	128	>128	>128	>128
**4ac**	32	32	>128	>128	>128
**5ac**	64	64	>128	>128	>128
**3ad**	>128	>128	>128	>128	>128
**4ad**	>128	>128	>128	>128	>128
**5ad**	32	64	>128	>128	>128
**3ae**	>128	>128	>128	>128	>128
**4ae**	>128	>128	>128	>128	>128
**5ae**	>128	>128	>128	>128	>128
**3af**	>128	>128	>128	>128	>128
**4af**	>128	>128	>128	>128	>128
**5af**	>128	>128	>128	>128	>128
**3ag**	>128	>128	>128	>128	>128
**4ag**	>128	>128	>128	>128	>128
**5ag**	1	1	>128	>128	128
**3ah**	>128	>128	>128	>128	>128
**5ah**	8	8	>128	>128	>128
**3ai**	128	128	>128	>128	>128
**5ai**	128	128	>128	>128	>128
**3aj**	16	16	>128	>128	>128
**5aj**	>128	>128	>128	>128	>128
**3ak**	64	64	>128	>128	>128
**4ak**	>128	>128	>128	>128	>128
**5ak**	>128	>128	>128	>128	>128
**3al**	8	8	>128	>128	>128
**4al**	>128	>128	>128	>128	>128
**5al**	32	32	>128	>128	>128
**3am**	128	128	>128	>128	>128
**5am**	0.5	0.5	>128	>128	128
**3bc**	128	128	>128	>128	>128
**4bd**	>128	>128	>128	>128	>128
**3be**	>128	>128	>128	>128	>128
**4bf**	>128	>128	>128	>128	>128
**3bg**	1	1	>128	>128	128
**3bh**	128	128	>128	>128	>128
**3bi**	4	4	>128	>128	>128
**4bi**	>128	>128	>128	>128	>128
**3bj**	128	64	>128	>128	>128
**4bj**	>128	>128	>128	>128	>128
**3bk**	>128	>128	>128	>128	>128
**4bk**	>128	>128	>128	>128	>128
**4bl**	>128	>128	>128	>128	>128
**3bm**	0.5	0.5	>128	>128	128
Cloxacillin	0.25	0.25	/	/	/
Vancomycin	0.25	0.25	/	/	/
Cefotaxime	/	/	0.03	0.03	/

a Reference sensitive strains.

b Clinical sensitive strains isolated from patients.

Compounds **3**, **4**, and **5** showed a variable antibacterial activity although their structures are similar. We observed a huge difference in terms of activity, varying from an MIC of 0.5 to an MIC superior to 128 µg/ml. The antimicrobial screening results suggested that the synthetized compounds exhibited a selective inhibitory activity against the reference and clinical Gram-positive *S. aureus* strains.

Concerning the juglone moiety, an interesting difference in antibacterial activity was observed. Only one dialkylated compound **4ac** exhibited an activity against methicillin-susceptible *Staphylococcus aureus* (MSSA) with an MIC of 32 µg/ml, whereas two other monoalkylated products with the same alkyl group showed a lower activity on these two strains with an MIC of, respectively, 128 µg/ml for **3ac** and 64 µg/ml for **5ac**.

Second, a difference in antibacterial activity between the two monoalkylated regioisomers was observed. When the alkyl group was grafted on C3 (compounds **5**), antibacterial activity was better than that in the other monoalkylated regioisomer grafted on C2 (compounds **3**). **3am** showed an MIC of 128 µg/ml on the reference and clinical MSSA strains, whereas **5am** showed an MIC of 0.5 µg/ml on MSSA. The same results were observed for **3ag**/**5ag**, **3ad**/**5ad**, **3ah**/**5ah**, and **3ac**/**5ac**. Three exceptions with **3aj**/**5aj**, **3ak**/**5ak**, and **3al**/**5al** were noticed.

Considering naphthazarin derivatives, MIC activities were lower than those for juglone derivatives for most compounds. The only naphthazarin compound that had a better antibacterial activity than juglone was **3bi** with an MIC of 4 µg/ml versus **3ai** with an MIC of 128 µg/ml on MSSA.

No compound showed antimicrobial activity against *E. coli* strains (MIC > 128 µg/ml), and four compounds (**5ag**, **5am**, **3bg**, and **3bm**) showed low activity against the *P. aeruginosa* strain (MIC: 128 µg/ml).

These results are promising in comparison with those described in the literature. Indeed, the *in vitro* antibacterial activity of juglone (Figure 1, **1a**) against *S. aureus* was investigated. The authors showed that juglone exhibits activity against MSSA with an MIC of 12.5 µg/ml (Clark et al., 1990). In our studies, we showed that compounds **5am** and **3bm** showed promising activity against clinical and reference MSSA (MIC: 0.5 µg/ml). The MIC was improved by a 24-fold factor.

#### Antibiotic Susceptibility Testing Against Resistant Clinical Strains of *E. coli* and *S. aureus*

We further evaluated antibacterial activity of compounds against one clinical isolate of methicillin-resistant *Staphylococcus aureus* (MRSA) and one clinical isolate of extended spectrum beta-lactamase (ESBL) *E. coli*. Both strains have been isolated from patients; *E. coli* is considered as resistant because of the high cefotaxime MIC (breakpoint of 2 μg/ml). This strain is an ESBL-producing bacteria and known to be resistant to most beta-lactam antibiotics. *S. aureus* is considered as resistant because of the high cloxacillin MIC (breakpoint of 2 μg/ml). The MRSA strain used is mainly resistant to the penicillin family but not to the glycopeptide family like vancomycin (breakpoint of 2 μg/ml) (Table 4) (Boibessot et al., 2016; Douafer et al., 2019; The European Committee on Antimicrobial Susceptibility Testing, 2021).

**TABLE 4:** MIC values (μg/ml) of the synthesized compounds **(3, 4, and 5)** against clinical resistant bacterial strains.

Compound	MIC (μg/ml)	
	Resistant Gram-positive bacteria: *S. aureus* NSARM-1^a^	Resistant Gram-negative bacteria: *E. coli* NEC-R3^b^
**4ac**	32	>128
**5ac**	64	>128
**5ag**	2	>128
**5ah**	32	>128
**5al**	32	>128
**5am**	2	>128
**3aj**	32	>128
**3ak**	64	>128
**3al**	8	>128
**3bg**	2	>128
**3bi**	16	>128
**3bm**	2	>128
Cloxacillin	2	/
Vancomycin	0.5	/
Cefotaxime	/	>128

a Clinical MRSA strain penicillinase producer resistant to the penicillin family and susceptible to vancomycin.

b Clinical ESBL *E. coli* strain resistant to most beta-lactam antibiotics.

The results are summarized in Table 4. Compounds **5ag**, **5am**, **3bg**, and **3bm** displayed potent antibacterial activity with an MIC of 2 μg/ml against MRSA. Compound **3al** showed antibacterial inhibition with an MIC of 8 μg/ml. Compounds **4ac**, **5ac**, **5ah**, **3aj**, **3ak**, **5al**, and **3bi** exhibited moderate inhibition with an MIC varying from 16 to 64 μg/ml. All compounds were found to be inactive against resistant clinical isolates of *E. coli*.

**FIGURE 3:**
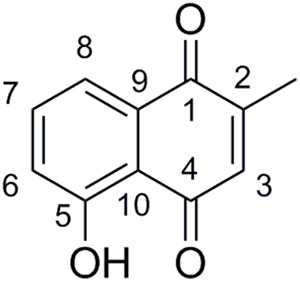
Structure of plumbagin.

These results are encouraging. Indeed, the best compounds of our study (**5ag**, **5am**, **3bg**, and **3bm**) presented an antibacterial activity of 2 μg/ml against clinical MRSA. Comparatively, plumbagin, 5-hydroxy-2-methyl-1,4-naphtoquinone (Figure 3), exhibited consistent activity against isolates displaying resistance to different classes of antibiotics with an MIC range of 4–8 μg/ml (Periasamy et al., 2019).

Our studies showed that the newly synthesized derivatives are not active against *E. coli* strains, the possible reason being that the compounds do not enter the bacteria because of its outer membrane and lack of access of the compounds, or the resistance mechanism of *E. coli* strains limiting the number of entryways and expressing less porins on the bacterial outer layer (Pagès et al., 2008).

#### Structure Activity Relationship Study

**FIGURE 4:**
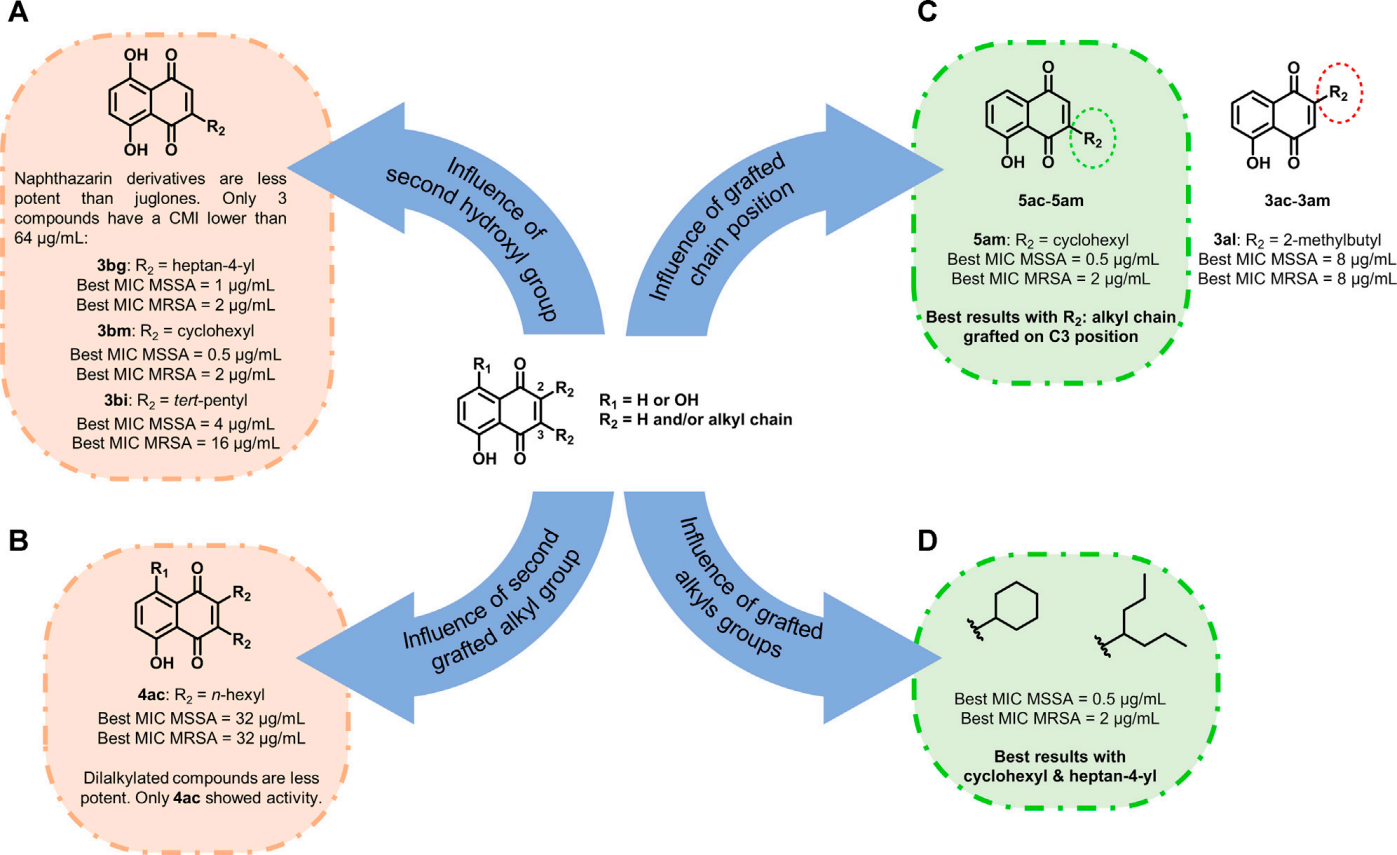
Structure–activity relationship study of juglone and naphthazarin derivatives. **(A)** Influence of second hydroxyl group, **(B)** influence of second grafted alkyl group, **(C)** influence of grafted chain position, and **(D)** influence of grafted alkyls groups.

From our previous observation, some conclusions can be realized on sensitive and resistant bacterial strains. First, naphthazarin derivatives were found to be less potent than juglones. However, three exceptions were observed: **3bg**, **3bm**, and **3bi** with MICs between 0.5 and 16 µg/ml (Figure 4A). Then the presence of a long aliphatic alkyl chain seemed unfavorable for the antibacterial activity. Compounds grafted with *n*-hexyl chains had a low activity (from 32 to 128 µg/ml for **3ac**–**5ac**) except for **5ad**. Moreover, with *n*-octyl and *n*-nonyl moieties, no antibacterial activity was observed for compounds **3ae**–**5af**. The same point was previously observed in the literature with naphthoquinones: The longer the carbon chain is, the higher the lipophilicity is and the lower the antimicrobial activity is (Sánchez-Calvo et al., 2016). Here, when the product is dialkylated, the activity is very low and only **4ac** showed antibacterial activity (Figure 4B). Considering the influence of the position of the grafted chain, we observed that when the alkyl chain was on C3 (compounds **5**), antibacterial activity was way higher than when C2 was substituted (compounds **3**, Figure 4C). Finally, the nature of the alkyl group was investigated. Best results were obtained when the alkyl group was ramified and contained between five and seven carbon atoms, especially with cyclohexyl and heptan-4-yl (Figure 4D).

#### Synergistic Effect of the Synthesized Compounds With Cloxacillin and Vancomycin by Fractional Inhibitory Concentration Index (FICI) Measurements

One strategy to solve the problem of ABR is to use an antibiotic adjuvant in combination with an antibiotic which will slow down the evolution of the bacteria’s resistance profile (Kim et al., 2014; Wright, 2016).

Measurement of the synergy of the newly synthesized derivatives with existing antibiotics is an important strategy to fight drug-resistant bacteria.

**FIGURE 5:**
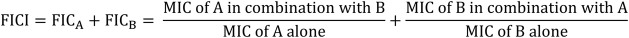
Formula of FICI.

Five compounds (**3al**, **5ag**, **5am**, **3bg**, and **3bm**) with best MICs on MSSA and MRSA (0.5–8 µg/ml) were selected to measure their synergy with two well-known antibiotics: cloxacillin (CLX) and vancomycin (VCM) on clinical resistant Gram-positive bacteria: MRSA (NSARM-1). To quantify this synergy, FICI was measured (Figure 5). Synergistic action can be considered when FICI ≤ 0.5 (Table 5) (Odds, 2003).

**TABLE 5:** Synergistic studies of compounds **3al**, **5ag**, **5am**, **3bg**, and **3bm** against clinical MRSA strains with vancomycin and cloxacillin, respectively.

MICs of drug alone (µg/ml)		MICs of drug when used in combination (µg/ml)		Fold MIC reduction		FICI	Effect
**VCM**	**3al**	**VCM**	**3al**	**VCM**	**3al**	1	Additivity
0.5	32	0.25	16	1/2	1/2		
**VCM**	**5ag**	**VCM**	**5ag**	**VCM**	**5ag**	1	Additivity
0.5	2	0.25	1	1/2	1/2		
**VCM**	**5am**	**VCM**	**5am**	**VCM**	**5am**	2	No synergy
0.5	0.5	0.5	0.5	1	1		
**VCM**	**3bg**	**VCM**	**3bg**	**VCM**	**3bg**	2	No synergy
0.5	1	0.5	1	1	1		
**VCM**	**3bm**	**VCM**	**3bm**	**VCM**	**3bm**	0.313	Synergy
0.5	0.5	0.03	0.125	1/16	1/4		
**CLX**	**3al**	**CLX**	**3al**	**CLX**	**3al**	1	Additivity
2	32	1	16	1/2	1/2		
C**LX**	**5ag**	**CLX**	**5ag**	**CLX**	**5ag**	1	Additivity
2	2	1	1	1/2	1/2		
**CLX**	**5am**	**CLX**	**5am**	**CLX**	**5am**	2	No synergy
2	0.5	2	0.5	1	1		
**CLX**	**3bg**	**CLX**	**3bg**	**CLX**	**3bg**	1	Additivity
2	1	1	0.5	1/2	1/2		
**CLX**	**3bm**	**CLX**	**3bm**	**CLX**	**3bm**	0.313	Synergy
2	0.5	0.125	0.125	1/16	1/4		

**FIGURE 6:**
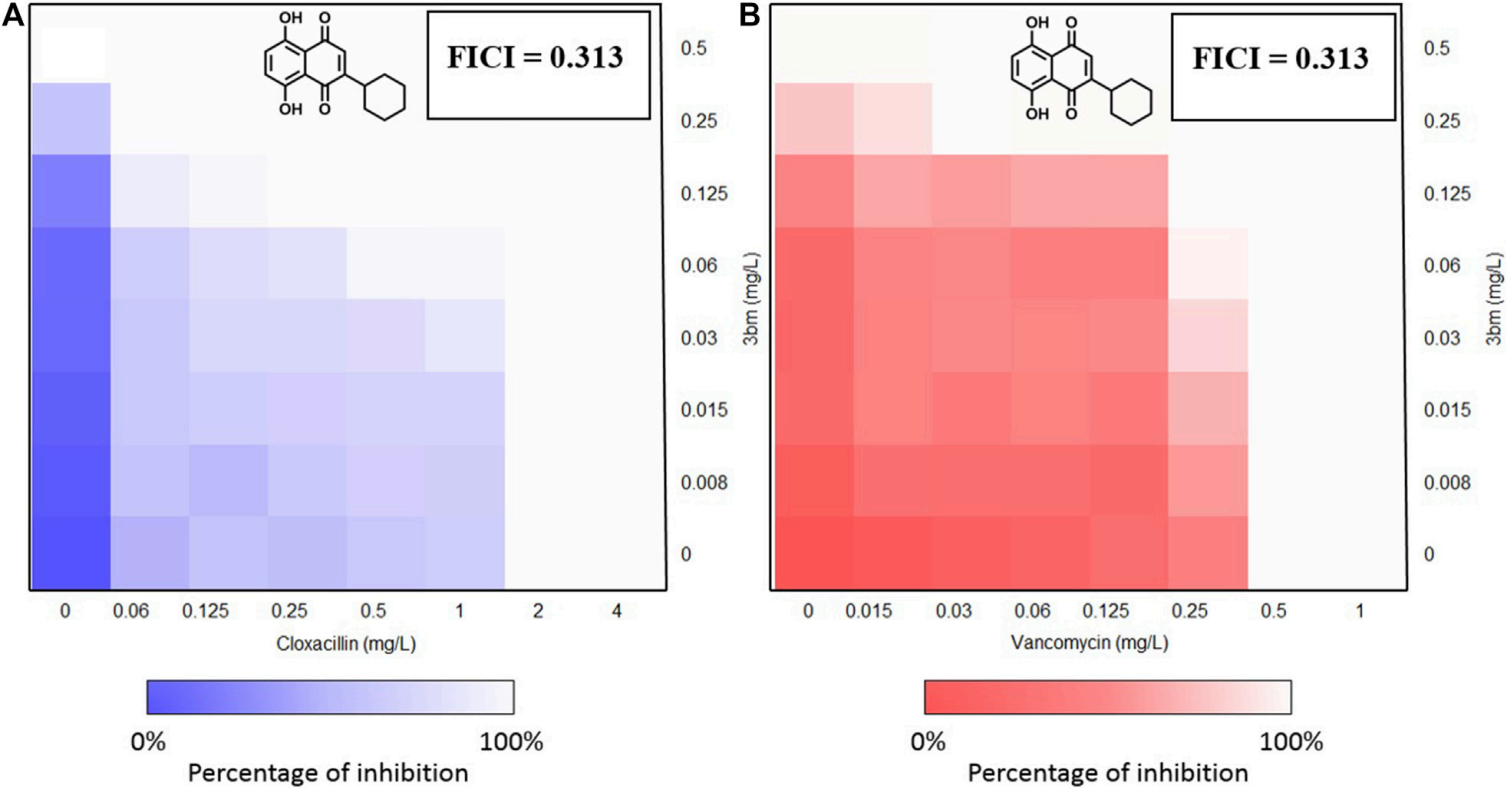
Heat maps of FICI measurements of **3bm** in association with cloxacillin **(A)** and vancomycin **(B)**. FICI calculations were realized at constant concentration when possible.

These results showed the absence of synergy for compounds **5am** and **3bg** with vancomycin and for compound **5am** with cloxacillin (FIC ≥ 1). An additivity phenomenon was observed for compounds **3al** and **5ag** with vancomycin and for compounds **5ag** and **3bg** with cloxacillin (0.5 < FIC < 1). Finally, compound **3bm** showed synergy with both vancomycin and cloxacillin with FIC = 0.313 (Table 5). For **3bm**, this is expressed by a fold potentiation of 16 for vancomycin (0.5–0.03 µg/ml) and cloxacillin (2–0.125 µg/ml). This potentiation is visible on following heat maps. The potentiation is not only due to the antibacterial properties of **3bm** as proven by our FICI measurements (Figure 6).

We also observed the restoration of vancomycin and cloxacillin activities on resistant strains of *S. aureus*. The MIC of cloxacillin and vancomycin on MSSA is 0.25 µg/ml, and the MICs of cloxacillin and vancomycin on MRSA are, respectively, 0.06 µg/ml and 0.03 µg/ml in the presence of 0.25 µg/ml of **3bm**. This observation means that in the presence of 0.25 µg/ml of **3bm**, a concentration of antibiotics 16 times lower is amply enough to inhibit bacterial growth.

**FIGURE 7:**
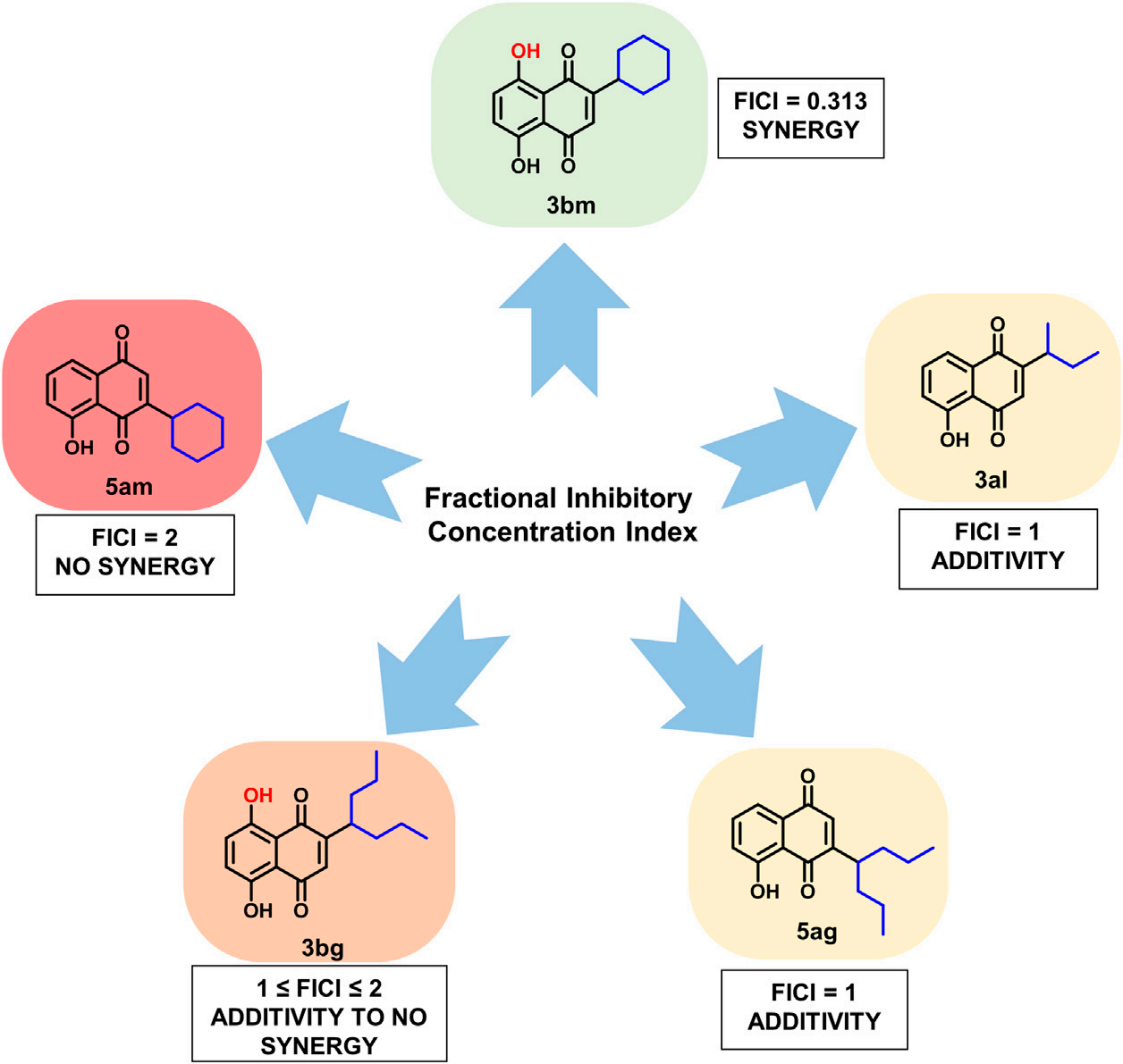
FICI results for compounds **3bm**, **3al**, **5ag**, **3bg**, and **5am** when added with cloxacillin or vancomycin on MRSA strains.

Finally, **3bm** is the only derivative to possess a synergistic effect with known antibiotics and can be, therefore, considered as an interesting compound for further studies (Figure 7).

### Hemolysis

The erythrocytes of mammals represent an excellent model to perform an *in vitro* evaluation of the cytotoxicity of molecules. Many published research studies have documented the possibility of using the resistance of mammalian red blood cell (RBC) membranes as a method of toxicity evaluation, by exposing sheep RBCs, using a standard protocol (material and methods), to different concentrations of compounds of interest (**3al**, **5ag**, **5am**, **3bg**, and **3bm**) (MIC to MRSA, 2 x MIC, 3 x MIC, 4 x MIC, 5 x MIC, and 7.5 x MIC) at room temperature for 1 h. No hemolytic activity was observed, which indicates that the tested molecules are not hemolytic even in a concentration 7.5 times the MIC.

### *In silico* ADME Study

Identifying and predicting the pharmacokinetics (PKs) of molecules is an important step in drug discovery. The PK profile is very often requested in medicinal chemistry projects and increasingly at an early stage of research projects. Modeling approaches are among the tools available to meet this data need. PK is one of the main reasons for stopping the development of a drug candidate; to avoid this problem, different *in silico* models have been recently developed*.* However, in practice *in vitro* tests seem to be essential to confirm the predictions. In this study, we used SwissADME, which is freely accessible and was originally developed to study the impact of orally active compounds on gastrointestinal absorption and bioavailability and delineated the relationship between pharmacokinetic and physicochemical parameters (Daina et al., 2017).

SwissADME, as its name suggests, allowed us to have access to preclinical data, especially *in vitro* data, on absorption, distribution, metabolism, and excretion (ADME). Table 6 summarizes the results obtained from most active juglone and naphthazarin derivatives with MIC ≤ 32 μg/ml and presents some predicted physicochemical and pharmacokinetic parameters. Particular attention has been given to pharmacokinetic properties, for example, gastrointestinal absorption (GI), blood–brain barrier (BBB) permeability, and cytochrome P450 (CYP) enzyme inhibitions. All of the compounds of interest showed good compatibility with Lipinski’s “rule of five” (Lipinski et al., 2012). The predicted values of cLogP are in the ideal range of LogP to be presented in the context of compound development. High gastrointestinal absorption (GI) is shown as well as the metabolism by the major drug-metabolizing CYP isoforms expressed in the liver (CYP1A2, CYP2C19, CY2C9, CYP2D6, and CYP3A4). Moreover, it is also important to estimate the fraction of drugs metabolized by these major CYP isoforms by performing *in vitro* ADME assays. Another important pharmacokinetic parameter blood–brain barrier (BBB) permeability was predicted *in silico*, and BBB was shown to be permeable to all evaluated molecules.

**TABLE 6:** Pharmacokinetic parameter values of interest.

#	CYP inhibitory profile									
	cLogP	Lipinski compatible	GI absorption	BBB permeant	Pgp substrate	CYP1A2	CYP2C19	CYP2C9	CYP2D6	CYP3A4
**4ac**	6.67	Yes	High	Yes	No	No	Yes	Yes	Yes	Yes
**5ag**	4.41	Yes	High	Yes	No	Yes	Yes	Yes	No	No
**5ah**	3.35	Yes	High	Yes	No	Yes	Yes	Yes	No	No
**3aj**	3.78	Yes	High	Yes	No	Yes	Yes	Yes	No	Yes
**5am**	3.86	Yes	High	Yes	No	Yes	Yes	Yes	No	Yes
**3al**	3.76	Yes	High	Yes	No	Yes	Yes	Yes	No	No
**5al**	3.76	Yes	High	Yes	No	Yes	Yes	Yes	No	No
**3bg**	4.23	Yes	High	Yes	No	Yes	Yes	Yes	No	No
**3bi**	3.54	Yes	High	Yes	No	Yes	No	No	No	Yes
**3bm**	3.72	Yes	High	Yes	No	Yes	Yes	Yes	No	No

Abbreviations: BBB, blood–brain barrier; GI, gastrointestinal; cLogP, calculated LogP.

This may be due to the weight of the juglone compounds: their molecular weight does not exceed 450 Da. Lipophilicity has a positive correlation with the ability to cross BBB, usually LogP for neutral compounds, with a minimal hydrophobicity (LogP > 5), which is the case of our compounds (Fong, 2015). And eventually orally administered juglone does not lead to an increase in the activity of Pgp, which is the most important member of active drug transporters, leading to multidrug resistance.

## Experimental Section

### Chemistry

All reagents were purchased from Aldrich Chemical Co. and used without any purification. NMR spectra were recorded with a Bruker Avance 300 spectrometer (300 and 75 MHz for ^1^H and ^13^C NMR, respectively). Chemical shifts (δ) and coupling constants (J) are given in ppm and Hz, respectively, using residual solvent signals as reference for ^1^H and ^13^C. The following abbreviations are used: s = singlet, d = doublet, t = triplet, q = quartet, quint = quintuplet, br s = broad signal, dd = double doublet, dt = double of triplet, and m = multiplet. High-resolution mass spectra (HRMS) were obtained by electrospray ionization using a TOF analyzer platform. IR spectra were obtained using a JASCO FT-IR 410 instrument on a thin film on NaCl disc as stated; only structurally important peaks (υ̅) are presented in cm^−1^. Reactions were monitored with Merck Kieselgel 60F254 precoated aluminum silica gel plates (0.25 mm thickness). Melting points were determined on a Stuart scientific SMP10 apparatus and are uncorrected. Flash chromatography was performed on a Grace Reveleris X2 apparatus using a 40-μm packed silica cartridge. Flash chromatography experiments were carried out on silica gel premium R_*f*_: grade (40–63 μm) or were performed on a Grace Reveleris X2 using a 40-μm packed silica cartridge. HPLC analyses were obtained on a Waters Alliance 2795 system using the following conditions: a Thermo Hypersil C18 column (3 μm, 50 mm L × 2.1 mm ID), 20°C column temperature, 0.2 ml/min flow rate, photodiode array detection (210–400 nm), and mobile phase consistent of a gradient of water and acetonitrile (each containing 0.1% trifluoroacetic acid). The purity of all synthetic compounds was determined by HPLC analysis and was > 95%.

#### General Synthetic Procedure for the Synthesis of **3ac**–**3bm**, **4ac**–**4bl**, and **5ac**–**5al**

To an argon backfilled Schlenk balloon, substrate (1 equiv.), carboxylic acid (10 equiv.), and ammonium persulfate (2–7 equiv.) were added. After a final backfill, DMSO/water (600:1 v/v 20.3 ml/mmol) degassed by bubbling with argon (1 balloon/1.5–2 h) was added, and the reaction was stirred at 40°C until complete substrate consumption, followed by TLC. The reaction mixture was diluted with AcOEt (100 ml) and filtered through a celite pad (5 cm). The organic layer was washed with sat. NaHCO_3_ solution (50 ml). The aqueous layer was then extracted with AcOEt (5 x 50 ml), and the combined organic layers were washed with water (3 x 50 ml), dried over MgSO_4_, and concentrated to give the crude product. The residue was further purified by flash column chromatography (silica gel, cyclohexane/toluene, 100/0 ramping to 0/100, and v/v for **3bc**, **3be**, **3bh**, **3bi**, and **4bi**; silica gel, PE 40–60°C/toluene, 100/0 ramping to 0/100, and v/v for the other compounds).

#### 2-Hexyl-5-hydroxynaphthalene-1,4-dione (**3ac**), 2,3-Dihexyl-5-hydroxynaphthalene-1,4-dione (**4ac**), and 3-Hexyl-5-hydroxynaphthalene-1,4-dione (**5ac**)

The reaction was carried out following the general procedure: 5-Hydroxy-1,4-naphthoquinone **1a** (400 mg, 2.30 mmol), heptanoic acid **2c** (3.26 ml, 23.0 mmol), and sodium persulfate (1.05 g, 4.60 mmol) in 45 ml of DMSO/water (600:1 v/v) were heated under reflux for 87 h at 40°C. **3ac**: Yield: 10% (60 mg); orange needles: mp 50–52°C. R_*f*_: 0.63 (toluene). IR (cm^−1^): 2927 (ν_C-H_), 1642 (ν_C=O_), 1453:1608 (ν_C=CAr_), and 1253 (ν_C-O_). ^1^H NMR (300 MHz, chloroform-d) δ 0.89 (t, *J* = 6.8 Hz, 3H, CH_3_), 1.25–1.42 (m, 6H, 3CH_2_), 1.56 (quint, *J* = 7.6 Hz, 2H, CH_2_), 2.55 (t, *J* = 7.6 Hz, 2H, CH_2_), 6.74 (t, *J* = 1.2 Hz, 1H, CH), 7.23 (dd, *J* = 2.2, 7.4 Hz, 1H, CH_Ar_), 7.55–7.64 (m, 2H, CH_Ar_), and 11.98 (s, 1H, OH). ^13^C NMR (75 MHz, chloroform-d) δ 14.19 (CH_3_), 22.66 (CH_2_), 28.05 (CH_2_), 29.17 (CH_2_), 29.76 (CH_2_), 31.67 (CH_2_), 115.15 (C_Ar_), 119.44 (CH_Ar_), 124.21 (CH_Ar_), 132.38 (C_Ar_), 134.61 (CH), 136.22 (CH_Ar_), 153.62 (C), 161.25 (C-OH), 184.63 (C=O), and 190.63 (C=O). HPLC: *t*_*R*_ *:* 18.54 min; purity: 96%. HRMS: [M + H]^+^ calcd for C_16_H_19_O_3_: 259.1334; found: 259.1345. **4ac**: Yield: 2% (6 mg); orange oil. R_*f*_: 0.65 (toluene). IR (cm^−1^): 2924 (ν_C-H_), 1631 (ν_C=O_), 1457 (ν_C=CAr_), and 1265 (ν_C-O_). ^1^H NMR (300 MHz, chloroform-d) δ 0.86–0.95 (m, 6H, 2CH_3_), 1.24–1.46 (m, 16H, 8CH_2_), 2.55–2.64 (m, 4H, 2CH_2_), 7.21 (dd, *J* = 1.9, 7.7 Hz, 1H, CH_Ar_), 7.52–7.62 (m, 2H, CH_Ar_), and 12.24 (s, 1H, OH). ^13^C NMR (75 MHz, chloroform-d) δ 14.22 (2CH_3_), 22.71 (2CH_2_), 26.64 (CH_2_), 27.29 (CH_2_), 29.75 (2CH_2_), 29.94 (2CH_2_), 31.71 (2CH_2_), 115.26 (C_Ar_), 118.91 (CH_Ar_), 123.79 (CH_Ar_), 132.46 (C_Ar_), 135.97 (CH_Ar_), 147.14 (C), 148.76 (C), 161.28 (C-OH), 184.58 (C=O), and 190.72 (C=O). HPLC: *t*_*R*_ *:* 23.57 min; purity: 95%. HRMS: [M + H]^+^ calcd for C_22_H_31_O_3_: 343.2268; found: 343.2267. **5ac**: Yield: 13% (77 mg); orange needles: mp 86°C. R_*f*_. 0.56 (toluene). IR (cm^−1^): 2961, 2932 (ν_C-H_), 1633 (ν_C=O_), 1456, 1606 (ν_C=CAr_), and 1221 (ν_C-O_). ^1^H NMR (300 MHz, chloroform-d) δ 0.89 (t, *J* = 7.2 Hz, 3H, CH_3_), 1.21–1.42 (m, 4H, 2CH_2_), 1.51–1.61 (m, 2H, CH_2_), 2.54 (t, *J* = 7.5 Hz, 2H, CH_2_), 6.74 (s, 1H, CH), 7.21–7.28 (m, 1H, CH_Ar_), 7.55–7.63 (m, 2H, CH_Ar_), and 12.10 (s, 1H, OH). ^13^C NMR (75 MHz, chloroform-d) δ 14.18 (CH_3_), 22.66 (CH_2_), 28.09 (CH_2_), 29.17 (2CH_2_), 31.66 (CH_2_), 115.33 (C_Ar_), 118.79 (CH_Ar_), 124.29 (CH_Ar_), 132.29 (C_Ar_), 135.71 (CH), 136.48 (CH_Ar_), 152.01 (C), 161.71 (C-OH), 184.58 (C=O), and 190.78 (C=O). HPLC: *t*_*R*_ *:* 18.12 min; purity: 98%. HRMS: [M + H]^+^ calcd for C_14_H_15_O_3_: 259.1334; found: 259.1341.

#### 2-Heptyl-5-hydroxynaphthalene-1,4-dione (**3ad**), 2,3-Diheptyl-5-hydroxynaphthalene-1,4-dione (**4ad**), and 2-Heptyl-8-hydroxynaphthalene-1,4-dione (**5ad**)

The reaction was carried out following the general procedure: 5-Hydroxy-1,4-naphthoquinone **1a** (303 mg, 1.74 mmol), octanoic acid **2d** (2.75 ml, 17.4 mmol), and sodium persulfate (797 mg, 3.49 mmol) in 35 ml of DMSO/water (600:1 v/v) were heated under reflux for 14.5 h at 40°C. **3ad**: Yield: 10% (46 mg); orange needles: mp 57–59°C. R_*f*_: 0.62 (toluene). IR (cm^−1^): 2924, 2853 (ν_C-H_), 1638 (ν_C=O_), 1455, 1607 (ν_C=CAr_), and 1228 (ν_C-O_). ^1^H NMR (300 MHz, chloroform-d) δ 0.88 (t, *J* = 6.8 Hz, 3H, CH_3_), 1.23–1.38 (m, 8H, 4CH_2_), 1.53–1.61 (m, 2H, CH_2_), 2.51–2.59 (m, 2H, CH_2_), 6.75 (t, *J* = 1.1 Hz, 1H, CH), 7.22–7.27 (m, 1H, CH_Ar_), 7.55–7.66 (m, 2H, CH_Ar_), and 11.99 (s, 1H, OH). ^13^C NMR (75 MHz, chloroform-d) δ 14.21 (CH_3_), 22.76 (CH_2_), 28.09 (CH_2_), 29.15 (CH_2_), 29.47 (CH_2_), 29.76 (CH_2_), 31.84 (CH_2_), 115.16 (C_Ar_), 119.44 (CH_Ar_), 124.21 (CH_Ar_), 132.39 (C_Ar_), 134.62 (CH), 136.22 (CH_Ar_), 153.64 (C), 161.26 (C-OH), 184.63 (C=O), and 190.64 (C=O). HPLC: *t*_*R*_ *:* 19.33 min; purity: 96%. [M + H]^+^ calcd for C_17_H_21_O_3_: 273.1485; found: 273.1488. **4ad**: Yield: 7% (44 mg); orange oil. R_*f*_: 0.71 (toluene). IR (cm^−1^): 2923 (ν_C-H_), 1631 (ν_C=O_), and 1457 (ν_C=CAr_). ^1^H NMR (300 MHz, chloroform-d) δ 0.82–0.93 (m, 6H, 2CH_3_), 1.26–1.49 (m, 20H, 10CH_2_), 2.55–2.62 (m, 4H, 2CH_2_), 7.21 (dd, *J* = 1.9, 7.7 Hz, 1H, CH_Ar_), 7.50–7.62 (m, 2H, CH_Ar_), and 12.24 (s, 1H, OH). ^13^C NMR (75 MHz, chloroform-d) δ 14.23 (2CH_3_), 22.79 (2CH_2_), 26.64 (CH_2_), 27.30 (CH_2_), 29.19 (2CH_2_), 29.78 (2CH_2_), 30.24 (2CH_2_), 31.89 (2CH_2_), 115.25 (C_Ar_), 118.89 (CH_Ar_), 123.78 (CH_Ar_), 132.45 (C_Ar_), 135.95 (CH_Ar_), 147.13 (C), 148.75 (C), 161.28 (C-OH), 184.55 (C=O), and 190.71 (C=O). HPLC: *t*_*R*_ *:* 25.11 min; purity: 98%. HRMS: [M + H]^+^ calcd for C_24_H_35_O_3_: 371.2581; found: 371.2586. **5ad**: Yield: 7% (34 mg); orange needles: mp 87–89°C. R_*f*_: 0.50 (toluene). IR (cm^−1^): 2923 (ν_C-H_), 1633 (ν_C=O_), 1455, 1608 (ν_C=CAr_), and 1229 (ν_C-O_). ^1^H NMR (300 MHz, chloroform-d) δ 0.89 (t, *J* = 6.8 Hz, 3H, CH_3_), 1.28–1.37 (m, 8H, 4CH_2_), 1.46–1.58 (m, 2H, CH_2_), 2.51–2.60 (m, 2H, CH_2_), 6.76 (s, 1H, CH), 7.20–7.28 (m, 1H, CH_Ar_), 7.55–7.65 (m, 2H, CH_Ar_), and 12.12 (s, 1H, OH). ^13^C NMR (75 MHz, chloroform-d) δ 14.21 (CH_3_), 22.76 (CH_2_), 28.15 (CH_2_), 29.15 (CH_2_), 29.19 (CH_2_), 29.46 (CH_2_), 31.84 (CH_2_), 115.35 (C_Ar_), 118.81 (CH_Ar_), 124.30 (CH_Ar_), 132.31 (C_Ar_), 135.73 (CH), 136.49 (CH_Ar_), 152.03 (C), 161.73 (C-OH), 184.60 (C=O), and 190.79 (C=O). HPLC: *t*_*R*_ *:* 19.18 min; purity: 96%. HRMS: [M + H]^+^ calcd for C_17_H_21_O_3_: 273.1485; found: 273.1488.

#### 5-Hydroxy-2-octylnaphthalene-1,4-dione (**3ae**), 5-Hydroxy-2,3-dioctylnaphthalene-1,4-dione (**4ae**), and 5-Hydroxy-3-octylnaphthalene-1,4-dione (**5ae**)

The reaction was carried out following the general procedure: 5-Hydroxy-1,4-naphthoquinone **1a** (300 mg, 1.58 mmol), nonanoic acid **2e** (2.26 ml, 15.8 mmol), and sodium persulfate (721 mg, 3.16 mmol) in 35 ml of DMSO/water (600:1 v/v) were heated under reflux for 15 h at 40°C. **3ae**: Yield: 10% (65 mg); orange needles: mp 48–50°C. R_*f*_: 0.70 (toluene). IR (cm^−1^): 2922, 2851 (ν_C-H_), 1637 (ν_C=O_), 1452, 1604 (ν_C=CAr_), and 1229 (ν_C-O_). ^1^H NMR (300 MHz, chloroform-d) δ 0.87 (t, *J* = 7.0 Hz, 3H, CH_3_), 1.21–1.42 (m, 10H, 5CH_2_), 1.55 (quint, *J* = 7.1 Hz, 2H, CH_2_), 2.52 (td, *J* = 1.0, 7.5 Hz, 2H, CH_2_), 6.73 (s, 1H, CH), 7.22 (dd, *J* = 2.4, 7.2 Hz, 1H, CH_Ar_), 7.52–7.64 (m, 2H, CH_Ar_), and 11.90 (s, 1H, OH). ^13^C NMR (75 MHz, chloroform-d) δ 14.21 (CH_3_), 22.76 (CH_2_), 28.06 (CH_2_), 29.28 (CH_2_), 29.28 (CH_2_), 29.44 (CH_2_), 29.49 (CH_2_), 31.94 (CH_2_), 115.11 (C_Ar_), 119.40 (CH_Ar_), 124.17 (CH_Ar_), 132.35 (C_Ar_), 134.57 (CH), 136.18 (CH_Ar_), 153.59 (C), 161.22 (C-OH), 184.58 (C=O), and 190.59 (C=O). HPLC: *t*_*R*_ *:* 19.48 min; purity: 96%. HRMS: [M + H]^+^ calcd for C_18_H_23_O_3_: 287.1647; found: 287.1656. **4ae**: Yield: 7% (10 mg); orange oil. R_*f*_: 0.86 (toluene). IR (cm^−1^): 2924 (ν_C-H_), 1633 (ν_C=O_), 1457 (ν_C=CAr_), and 1262 (ν_C-O_). ^1^H NMR (300 MHz, chloroform-d) δ 0.84–0.91 (m, 6H, 2CH_3_), 1.22–1.54 (m, 24H, 12CH_2_), 2.54–2.62 (m, 4H, 2CH_2_), 7.19 (dd, *J* = 1.9, 7.7 Hz, 1H, CH_Ar_), 7.51–7.61 (m, 2H, CH_Ar_), and 12.23 (s, 1H, OH). ^13^C NMR (75 MHz, chloroform-d) δ 14.24 (2CH_3_), 22.80 (2CH_2_), 26.63 (CH_2_), 27.28 (CH_2_), 29.34 (2CH_2_), 29.48 (2CH_2_), 29.76 (2CH_2_), 30.28 (2CH_2_), 32.00 (2CH_2_), 115.23 (C_Ar_), 118.88 (CH_Ar_), 123.76 (CH_Ar_), 132.43 (C_Ar_), 135.93 (CH_Ar_), 147.11 (C), 148.74 (C), 161.26 (C-OH), 184.53 (C=O), and 190.69 (C=O). HPLC: *t*_*R*_ *:* 25.20 min; purity: 100%. HRMS: [M + H]^+^ calcd for C_26_H_39_O_3_: 399.2899; found: 399.2900. **5ae**: Yield: 8% (52 mg); orange needles: mp 90–92°C. R_*f*_: 0.59 (toluene). IR (cm^−1^): 2920, 2851 (ν_C-H_), 1634 (ν_C=O_), 1453: 1607 (ν_C=CAr_), and 1226 (ν_C-O_). ^1^H NMR (300 MHz, chloroform-d) δ 0.87 (t, *J* = 6.6 Hz, 3H, CH_3_), 1.23–1.43 (m, 10H, 5CH_2_), 1.58 (quint, *J* = 7.0, 2H, CH_2_), 2.54 (t, *J* = 7.0 Hz, 2H, CH_2_), 6.74 (s, 1H, CH), 7.23 (dd, *J* = 2.9, 6.7 Hz, 1H, CH_Ar_), 7.55–7.63 (m, 2H, CH_Ar_), and 12.10 (s, 1H, OH). ^13^C NMR (75 MHz, chloroform-d) δ 14.22 (CH_3_), 22.77 (CH_2_), 28.12 (CH_2_), 29.18 (CH_2_), 29.29 (CH_2_), 29.44 (CH_2_), 29.49 (CH_2_), 31.95 (CH_2_), 115.32 (C_Ar_), 118.78 (CH_Ar_), 124.28 (CH_Ar_), 132.28 (C_Ar_), 135.70 (CH), 136.47 (CH_Ar_), 152.00 (C), 161.70 (C-OH), 184.56 (C=O), and 190.77 (C=O). HPLC: *t*_*R*_ *:* 19.66 min; purity: 98%. HRMS: [M + H]^+^ calcd for C_18_H_23_O_3_: 287.1647; found: 287.1646.

#### 5-Hydroxy-2-nonylnaphthalene-1,4-dione (**3af**), 5-Hydroxy-2,3-dinonylnaphthalene-1,4-dione (**4af**), and 5-Hydroxy-3-nonylnaphthalene-1,4-dione (**5af**)

The reaction was carried out following the general procedure: 5-Hydroxy-1,4-naphthoquinone **1a** (300 mg, 1.73 mmol), decanoic acid **2f** (2.97 ml, 17.2 mmol), and sodium persulfate (788 mg, 3.47 mmol) in 35 ml of DMSO/water (600:1 v/v) were heated under reflux for 22 h at 40°C. **3af**: Yield: 18% (95 mg); orange needles: mp 61°C. R_*f*_: 0.33 (1:1 PE 40–60°C/toluene). IR (cm^−1^): 2923 (ν_C-H_), 1638 (ν_C=O_), 1454, 1607 (ν_C=CAr_), and 1226 (ν_C-O_). ^1^H NMR (300 MHz, chloroform-d) δ 0.87 (t, *J* = 6.6 Hz, 3H, CH_3_), 1.24–1.35 (m, 12H, 6CH_2_), 1.50–1.63 (m, 2H, CH_2_), 2.55 (t, *J* = 7.6 Hz, 2H, CH_2_), 6.74 (s, 1H, CH), 7.24 (dd, *J* = 2.1, 7.5 Hz, 1H, CH_Ar_), 7.54–7.67 (m, 2H, CH_Ar_), and 11.98 (s, 1H, OH). ^13^C NMR (75 MHz, chloroform-d) δ 14.24 (CH_3_), 22.80 (CH_2_), 28.09 (CH_2_), 29.41 (CH_2_), 29.50 (2CH_2_), 29.59 (CH_2_), 29.77 (CH_2_), 31.99 (CH_2_), 115.16 (C_Ar_), 119.45 (CH_Ar_), 124.22 (CH_Ar_), 132.40 (C_Ar_), 134.63 (CH), 136.23 (CH_Ar_), 153.65 (C), 161.27 (C-OH), 184.65 (C=O), and 190.65 (C=O). HPLC: *t*_*R*_ *:* 21.31 min; purity: 98%. [M + H]^+^ calcd for C_19_H_25_O_3_: 301.1798; found: 301.1797. **4af**: Yield: 5% (33 mg); orange oil. mp 61°C. R_*f*_: 0.48 (1:1 PE 40–60°C/toluene). IR (cm^−1^): 2922, 2852 (ν_C-H_), 1631 (ν_C=O_), 1457, 1604 (ν_C=CAr_), and 1259 (ν_C-O_). ^1^H NMR (300 MHz, chloroform-d) δ 0.88 (t, *J* = 6.4 Hz, 6H, 2CH_3_), 1.22–1.53 (m, 28H, 14CH_2_), 2.58 (t, 7.5 Hz, 4H, 2CH_2_), 7.20 (dd, *J* = 1.8, 7.7 Hz, 1H, CH_Ar_), 7.52–7.61 (m, 2H, CH_Ar_), and 12.23 (s, 1H, OH). ^13^C NMR (75 MHz, chloroform-d) δ 14.25 (2CH_3_), 22.81 (2CH_2_), 26.64 (CH_2_), 27.29 (CH_2_), 29.45 (2CH_2_), 29.53 (2CH_2_), 29.64 (2CH_2_), 29.76 (2CH_2_), 30.27 (2CH_2_), 32.02 (2CH_2_), 115.24 (C_Ar_), 118.89 (CH_Ar_), 123.77 (CH_Ar_), 132.44 (C_Ar_), 135.94 (CH_Ar_), 147.12 (C), 148.75 (C), 161.27 (C-OH), 184.54 (C=O), and 190.70 (C=O). HPLC: *t*_*R*_ *:* 30.97 min; purity: 96%. HRMS: [M + H]^+^ calcd for C_28_H_43_O_3_: 427.3207; found: 427.3210. **5af**: Yield: 12% (62 mg); orange needles: mp 87–89°C. R_*f*_. 0.24 (1:1 PE 40–60°C/toluene). IR (cm^−1^): 2915, 2850 (ν_C-H_), 1636 (ν_C=O_), 1456, 1610 (ν_C=CAr_), and 1227 (ν_C-O_). ^1^H NMR (300 MHz, chloroform-d) δ 0.87 (t, *J* = 6.9 Hz, 3H, CH_3_), 1.21–1.43 (m, 12H, 6CH_2_), 1.51–1.63 (m, 2H, CH_2_), 2.50–2.57 (m, 2H, CH_2_), 6.74 (t, *J* = 1.2 Hz, 1H, CH), 7.23 (dd, *J* = 2.8, 6.8 Hz, 1H, CH_Ar_), 7.55–7.63 (m, 2H, CH_Ar_), and 12.09 (s, 1H, OH). ^13^C NMR (75 MHz, chloroform-d) δ 14.22 (CH_3_), 22.78 (CH_2_), 28.12 (CH_2_), 29.17 (CH_2_), 29.40 (CH_2_), 29.48 (2CH_2_), 29.58 (CH_2_), 31.98 (CH_2_), 115.31 (C_Ar_), 118.77 (CH_Ar_), 124.26 (CH_Ar_), 132.28 (C_Ar_), 135.69 (CH), 136.45 (CH_Ar_), 151.99 (C), 161.70 (C-OH), 184.54 (C=O), and 190.76 (C=O). HPLC: *t*_*R*_ *:* 21.17 min; purity: 95%. HRMS: [M + H]^+^ calcd for C_19_H_25_O_3_: 301.1798; found: 301.1795.

#### 2-(Heptan-4-yl)-5-hydroxynaphthalene-1,4-dione (**3ag**), 2,3-Di(heptan-4-yl)-5-hydroxynaphthalene-1,4-dione (**4ag**), and 3-(Heptan-4-yl)-5-hydroxynaphthalene-1,4-dione (**5ag**)

The reaction was carried out following the general procedure: 5-Hydroxy-1,4-naphthoquinone **1a** (301 mg, 1.73 mmol), 2-propylpentanoic acid **2g** (2.80 ml, 17.2 mmol), and sodium persulfate (788 mg, 3.47 mmol) in 35 ml of DMSO/water (600:1 v/v) were heated under reflux for 22 h at 40°C. **3ag**: Yield: 19% (89 mg); orange oil. R_*f*_. 0.41 (1:1 PE 40–60°C/toluene). IR (cm^−1^): 2916, 2850 (ν_C-H_), 1636 (ν_C=O_), 1456, 1610 (ν_C=CAr_), and 1227 (ν_C-O_). ^1^H NMR (300 MHz, chloroform-d) δ 0.87 (t, *J* = 7.3 Hz, 6H, 2CH_3_), 1.18–1.34 (m, 4H, 2CH_2_), 1.43–1.64 (m, 4H, 2CH_2_), 3.01–3.13 (m, 1H, CH), 6.69 (s, 1H, CH), 7.22–7.28 (m, 1H, CH_Ar_), 7.56–7.66 (m, 2H, CH_Ar_), and 11.99 (s, 1H, OH). ^13^C NMR (75 MHz, chloroform-d) δ 14.25 (CH_3_), 20.67 (2CH_2_), 37.05 (2CH_2_), 37.46 (CH), 115.00 (C_Ar_), 119.64 (CH_Ar_), 124.14 (CH_Ar_), 132.51 (C_Ar_), 134.11 (CH), 136.24 (CH_Ar_), 157.34 (C), 161.21 (C-OH), 184.45 (C=O), and 190.76 (C=O). HPLC: *t*_*R*_ *:* 18.39 min; purity: 96%. HRMS: [M + H]^+^ calcd for C_17_H_21_O_3_: 273.1485; found: 273.1493. **4ag**: Yield: 2% (14 mg); orange oil. R_*f*_. 0.64 (1:1 PE 40–60°C/toluene). IR (cm^−1^): 2916, 2850 (ν_C-H_), 1636 (ν_C=O_), 1456, 1610 (ν_C=CAr_), and 1227 (ν_C-O_). ^1^H NMR (300 MHz, chloroform-d) δ 0.85–0.94 (m, 12H, 4CH_3_), 1.10–1.45 (m, 8H, 4CH_2_), 1.62–1.89 (m, 8H, 4CH_2_), 2.86–3.01 (m, 2H, 2CH), 7.18 (dd, *J* = 3.1, 6.4 Hz, 1H, CH_Ar_), 7.50–7.58 (m, 2H, CH_Ar_), and 12.28 (s, 1H, OH). ^13^C NMR (75 MHz, chloroform-d) δ 14.62 (4CH_3_), 22.15 (4CH_2_), 36.65 (4CH_2_), 40.89 (2CH), 115.48 (C_Ar_), 118.57 (CH_Ar_), 123.47 (CH_Ar_), 132.83 (C_Ar_), 135.77 (CH_Ar_), 151.04 (C), 152.93 (C), 161.21 (C-OH), 184.95 (C=O), and 191.15 (C=O). HPLC: *t*_*R*_ *:* 24.25 min; purity: 95%. HRMS: [M + H]^+^ calcd for C_24_H_35_O_3_: 371.2567; found: 371.2582. **5ag**: Yield: 17% (80 mg); orange needles: mp 71–73°C. R_*f*_. 0.28 (1:1 PE 40–60°C/toluene). IR (cm^−1^): 2961, 2932 (ν_C-H_), 1633 (ν_C=O_), 1456, 1606 (ν_C=CAr_), and 1221 (ν_C-O_). ^1^H NMR (300 MHz, chloroform-d) δ 0.88 (t, *J* = 7.2 Hz, 6H, 2CH_3_), 1.20–1.32 (m, 4H, 2CH_2_), 1.45–1.63 (m, 4H, 2CH_2_), 3.01–3.13 (m, 1H, CH), 6.70 (s, 1H, CH), 7.21–7.28 (m, 1H, CH_Ar_), 7.56–7.65 (m, 2H, CH_Ar_), and 12.17 (s, 1H, OH). ^13^C NMR (75 MHz, chloroform-d) δ 14.21 (2CH_3_), 20.63 (2CH_2_), 36.64 (2CH), 37.02 (2CH_2_), 115.37 (C_Ar_), 118.68 (CH_Ar_), 124.34 (CH_Ar_), 132.07 (C_Ar_), 135.13(CH), 136.40 (CH_Ar_), 155.64 (C), 161.81 (C-OH), 184.73 (C=O), and 190.59 (C=O). HPLC: *t*_*R*_ *:* 18.27 min; purity: 97%. HRMS: [M + H]^+^ calcd for C_17_H_21_O_3_: 273.1485; found: 273.1491.

#### 2-(Sec-butyl)-5-hydroxynaphthalene-1,4-dione (**3ah**) and 3-(Sec-butyl)-5-hydroxynaphthalene-1,4-dione (**5ah**)

The reaction was carried out following the general procedure, with 5-hydroxy-1,4-naphthoquinone **1a** (408 mg, 2.30 mmol), 2-methylbutyric acid **2h** (2.50 ml, 23.0 mmol), and sodium persulfate (1.05 g, 4.59 mmol) in 45 ml of DMSO/water (600:1 v/v) was heated under reflux for 129 h at 40°C. **3ah**: Yield: 14% (72 mg); orange needles: mp 54°C. R_*f*_. 0.67 (Toluene). IR (cm^−1^): 2974 (ν_C-H_), 1636 (ν_C=O_), 1451 (ν_C=CAr_), and 1242 (ν_C-O_). ^1^H NMR (300 MHz, Chloroform-d) δ 0.91 (t, *J* = 7.4 Hz, 3H, CH_3_), 1.17 (d, *J* = 6.9 Hz, 3H, CH_3_), 1.40–1.70 (m, 2H, CH_2_), 2.98–3.11 (m, 1H, CH), 6.68 (d, *J* = 0.8 Hz, 1H, CH), 7.22 (dd, *J* = 2.0, 7.5 Hz, 1H, CH_Ar_), 7.55–7.64 (m, 2H, CH_Ar_), and 11.96 (s, 1H, OH). ^13^C NMR (75 MHz, Chloroform-d) δ 11.85 (CH_3_), 19.15 (CH_3_), 28.84 (CH_2_), 33.71 (CH), 114.96 (C_Ar_), 119.51 (CH_Ar_), 124.09 (CH_Ar_), 132.53 (C_Ar_), 133.32 (CH), 136.22 (CH_Ar_), 158.02 (C), 161.17 (C-OH), 184.23 (C=O), and 190.81 (C=O). HPLC: *t*_*R*_ *:* 15.30 min; purity: 96%. HRMS: [M + H]^+^ calcd for C_14_H_15_O_3_: 231.1016; found: 231.1017. **5ah**: Yield: 8% (43 mg); orange needles: mp 62–64°C. R_*f*_. 0.59 (Toluene). IR (cm^−1^): 2925, 2851 (ν_C-H_), 1640 (ν_C=O_), 1446, 1609 (ν_C=CAr_), and 1252 (ν_C-O_). ^1^H NMR (300 MHz, Chloroform-d) δ 0.86 (t, *J* = 7.4 Hz, 3H, CH_3_), 1.11 (d, *J* = 7.0 Hz, 3H, CH_3_), 1.35–1.66 (m, 2H, CH_2_), 2.91–3.06 (m, 1H, CH), 6.64 (s, 1H, CH), 7.14–7.21 (m, 1H, CH_Ar_), 7.52 (m, 2H, CH_Ar_), and 12.09 (s, 1H, OH). ^13^C NMR (75 MHz, Chloroform-d) δ 11.87 (CH_3_), 19.20 (CH_3_), 28.86 (CH_2_), 33.09 (CH), 115.45 (C_Ar_), 118.71 (CH_Ar_), 124.36 (CH_Ar_), 132.09 (C_Ar_), 134.38 (CH), 136.43 (CH_Ar_), 156.43 (C), 161.80 (C-OH), 184.83(C=O), and 190.47 (C=O). HPLC: *t*_*R*_ *:* 15.30 min; purity: 97%. HRMS: [M + H]^+^ calcd for C_14_H_15_O_3_: 231.1016; found: 231.1017.

#### 5-Hydroxy-2-(tert-pentyl)naphthalene-1,4-dione (**3ai**) and 8-Hydroxy-2-(tert-pentyl)naphthalene-1,4-dione (**5ai**)

The reaction was carried out following the general procedure: 5-Hydroxy-1,4-naphthoquinone **1a** (404 mg, 2.32 mmol), 2,2-dimethylbutyric acid **2i** (2.89 ml, 23.2 mmol), and sodium persulfate (1.08 g, 4.64 mmol) in 45 ml of DMSO/water (600:1 v/v) were heated under reflux for 85 h at 40°C. **3ai**: Yield: 10% (56 mg); orange needles: mp 57°C. R_*f*_. 0.59 (1:1 PE 40–60°C/toluene). IR (cm^−1^): 2963 (ν_C-H_), 1455, 1634 (ν_C=O_), 1596 (ν_C=CAr_), 1255 (ν_C-O_). ^1^H NMR (300 MHz, chloroform-d) δ 0.72 (t, *J* = 7.6 Hz, 3H, CH_3_), 1.29 (s, 6H, 2CH_3_), 1.87 (q, *J* = 7.5 Hz, 2H, CH_2_), 6.76 (s, 1H, CH), 7.18–7.24 (m, 1H, CH_Ar_), 7.56–7.62 (m, 2H, CH_Ar_), and 11.87 (s, 1H, OH). ^13^C NMR (75 MHz, chloroform-d) δ 9.58 (CH_3_), 27.44 (2CH_3_), 33.58 (CH_2_), 39.83 (C), 114.91 (C_Ar_), 119.66 (CH_Ar_), 123.53 (CH_Ar_), 133.55 (C_Ar_), 135.58 (CH), 136.33 (CH_Ar_), 159.07 (C), 160.94 (C-OH), 184.30 (C=O), and 190.65 (C=O). HPLC: *t*_*R*_ *:* 16.89 min; purity: 95%. HRMS: [M + H]^+^ calcd for C_15_H_17_O_3_: 245.1172; found: 245.1175. **5ai**: Yield: 8% (56 mg); orange needles: mp 97–98°C. R_*f*_. 0.53 (1:1 PE 40–60°C/toluene). IR (cm^−1^): 2963 (ν_C-H_), 1600 (ν_C=O_), 1455, 1596 (ν_C=CAr_), and 1255 (ν_C-O_). ^1^H NMR (300 MHz, chloroform-d) δ 0.74 (t, *J* = 7.6 Hz, 3H, CH_3_), 1.31 (s, 6H, 2CH_3_), 1.89 (q, *J* = 7.5 Hz, 2H, CH_2_), 6.77 (s, 1H, CH), 7.21–7.28 (m, 1H, CH_Ar_), 7.56–7.60 (m, 2H, CH_Ar_), and 12.35 (s, 1H, OH). ^13^C NMR (75 MHz, chloroform-d) δ 9.61 (CH_3_), 27.57 (2CH_3_), 33.56 (CH_2_), 39.67 (C), 116.13 (C_Ar_), 118.38 (CH_Ar_), 124.65 (CH_Ar_), 131.89 (C_Ar_), 136.16 (CH), 136.56 (CH_Ar_), 157.11 (C), 162.10 (C-OH), 184.95 (C=O), and 191.13 (C=O). HPLC: *t*_*R*_ *:* 17.04 min; purity: 98%. HRMS: [M + H]^+^ calcd for C_15_H_17_O_3_: 245.1172; found: 245.1177.

#### 5-Hydroxy-2,3-di-tert-pentylnaphthalene-1,4-dione (**3aj**) and 5-Hydroxy-3-neopentylnaphthalene-1,4-dione (**5aj**)

The reaction was carried out following the general procedure: 5-Hydroxy-1,4-naphthoquinone **1a** (402 mg, 2.30 mmol), 3:3-dimethylbutyric acid **2j** (2.90 ml, 23.0 mmol), and sodium persulfate (1.05 g, 4.60 mmol) in 45 ml of DMSO/water (600:1 v/v) were heated under reflux for 87 h at 40°C. **3aj**: Yield: 19% (62 mg); orange needles: mp 44–46°C. R_*f*_. 0.68 (toluene). IR (cm^−1^): 2955 (ν_C-H_), 1637 (ν_C=O_), 1456, 1603 (ν_C=CAr_), and 1252 (ν_C-O_). ^1^H NMR (300 MHz, chloroform-d) δ 0.95 (s, 9H, 3CH_3_), 2.51 (s, 2H, CH_2_), 6.74 (t, *J* = 0.7 Hz, 1H, CH), 7.15–7.31 (m, 1H, CH_Ar_), 7.54–7.67 (m, 2H, CH_Ar_), and 11.97 (s, 1H, OH). ^13^C NMR (75 MHz, chloroform-d) δ 29.84 (3CH_3_), 32.73 (C), 41.84 (CH_2_), 115.27 (C_Ar_), 119.68 (CH_Ar_), 124.09 (CH_Ar_), 132.28 (C_Ar_), 136.24 (CH), 137.20 (CH_Ar_), 151.30 (C), 161.19 (C-OH), 184.76 (C=O), and 190.23 (C=O). HPLC: *t*_*R*_ *:* 16.23 min; purity: 95%. HRMS: [M + H]^+^ calcd for C_15_H_17_O_3_: 245.1172; found: 245.1173. **5aj**: Yield: 5% (23 mg); orange needles: mp 140°C. R_*f*_. 0.56 (toluene). IR (cm^−1^): 2957 (ν_C-H_), 1631 (ν_C=O_), 1455, 1604 (ν_C=CAr_), and 1226 (ν_C-O_). ^1^H NMR (300 MHz, chloroform-d) δ 0.96 (s, 9H, 3CH_3_), 2.50 (s, 2H, CH_2_), 6.75 (s, 1H, CH), 7.19–7.27 (m, 1H, CH_Ar_), 7.54–7.66 (m, 2H, CH_Ar_), and 12.15 (s, 1H, OH). ^13^C NMR (75 MHz, chloroform-d) δ 29.82 (3CH_3_), 32.66 (C), 41.11 (CH_2_), 115.18 (C_Ar_), 118.73 (CH_Ar_), 124.36 (CH_Ar_), 132.38 (C_Ar_), 136.46 (CH), 138.38 (CH_Ar_), 149.59 (C), 161.77 (C-OH), 184.31 (C=O), and 190.85 (C=O). HPLC: *t*_*R*_ *:* 15.96 min; purity: 97%. HRMS: [M + H]^+^ calcd for C_15_H_17_O_3_: 245.1172; found: 245.1173.

#### 5-Hydroxy-2-isobutylnaphthalene-1,4-dione (**3ak**), 5-Hydroxy-2,3-diisobutylnaphthalene-1,4-dione (**4ak**), and 5-Hydroxy-3-isobutylnaphthalene-1,4-dione (**5ak**)

The reaction was carried out following the general procedure: 5-Hydroxy-1,4-naphthoquinone **1a** (400 mg, 2.30 mmol), isovaleric acid **2k** (2.50 ml, 23.0 mmol), and sodium persulfate (1.08 g, 4.60 mmol) in 45 ml of DMSO/water (600:1 v/v) were heated under reflux for 87 h at 40°C. **3ak**: Yield: 9% (46 mg); orange needles: mp 69°C. R_*f*_. 0.76 (toluene). IR (cm^−1^): 2959 (ν_C-H_), 1633 (ν_C=O_), 1454, 1608 (ν_C=CAr_), and 1252 (ν_C-O_). ^1^H NMR (300 MHz, chloroform-d) δ 0.97 (d, *J* = 6.7 Hz, 6H, 2CH_3_), 1.86–2.04 (m, 1H, CH), 2.43 (dd, *J* = 1.2, 7.1 Hz, 2H, CH_2_), 6.74 (t, *J* = 1.2 Hz, 1H, CH), 7.20–7.28 (m, 1H, CH_Ar_), 7.56–7.62 (m, 2H, CH_Ar_), and 12.11 (s, 1H, OH). ^13^C NMR (75 MHz, chloroform-d) δ 22.65 (2CH_3_), 27.91 (CH), 38.50 (CH_2_), 115.28 (C_Ar_), 118.77 (CH_Ar_), 124.30 (CH_Ar_), 132.28 (C_Ar_), 136.47 (CH), 136.79 (CH_Ar_), 150.75 (C) 161.71 (C-OH), 184.47 (C=O), and 190.82 (C=O). HPLC: *t*_*R*_ *:* 16.86 min; purity: 96%. HRMS: [M + H]^+^ calcd for C_14_H_15_O_3_: 231.1016; found: 231.1020. **4ak**: Yield: 4% (23 mg); orange oil. R_*f*_. 0.76 (toluene). IR (cm^−1^): 2958 (ν_C-H_), 1631 (ν_C=O_), 1456, 1600 (ν_C=CAr_), and 1265 (ν_C-O_). ^1^H NMR (300 MHz, chloroform-d) δ 0.91–0.99 (m, 12H, 4CH_3_), 1.88–1.94 (m, 2H, 2CH), 2.56 (d, *J* = 7.2 Hz, 4H, 2CH_2_), 7.22 (dd, *J* = 2.0, 7.5 Hz, 1H, CH_Ar_), 7.53–7.62 (m, 2H, CH_Ar_), and 12.25 (s, 1H, OH). ^13^C NMR (75 MHz, chloroform-d) δ 22.95 (4CH_3_), 29.30 (2CH), 35.26 (CH_2_), 35.93 (CH_2_), 115.24 (C_Ar_), 118.95 (CH_Ar_), 123.77 (CH_Ar_), 132.46 (C_Ar_), 135.99 (CH_Ar_), 147.04 (C), 148.84 (C), 161.30 (C-OH), 184.69 (C=O), and 190.80 (C=O). HPLC: *t*_*R*_ *:* 19.20 min; purity: 98%. HRMS: [M + H]^+^ calcd for C_18_H_23_O_3_: 287.1647; found: 287.1656. **5ak**: Yield: 6% (32 mg); orange needles: mp 78°C. R_*f*_. 0.68 (toluene). IR (cm^−1^): 2971 (ν_C-H_), 1630 (ν_C=O_), 1454 (ν_C=CAr_), and 1226 (ν_C-O_). ^1^H NMR (300 MHz, chloroform-d) δ 0.97 (d, *J* = 6.6 Hz, 6H, 2CH_3_), 1.84–2.05 (m, 1H, CH), 2.43 (dd, *J* = 1.0, 7.1 Hz, 2H, CH_2_), 6.73 (t, *J* = 1.0 Hz, 1H, CH), 7.18–7.28 (m, 1H, CH_Ar_), 7.54–7.66 (m, 2H, CH_Ar_), and 11.97 (s, 1H, OH). ^13^C NMR (75 MHz, chloroform-d) δ 22.68 (2CH_3_), 27.92 (CH), 38.98 (CH_2_), 115.18 (C_Ar_), 119.48 (CH_Ar_), 124.19 (CH_Ar_), 132.34 (C_Ar_), 135.68 (CH), 136.22 (CH_Ar_), 152.39 (C), 161.23 (C-OH), 184.47 (C=O), and 190.49 (C=O). HPLC: 15.16 min; purity: 95%. HRMS: [M + H]^+^ calcd for C_14_H_15_O_3_: 231.1016; found: 231.1020.

#### 5-Hydroxy-2-(2-methylbutyl)naphthalene-1,4-dione (**3al**), 5-Hydroxy-2,3-bis(2-methylbutyl)naphthalene-1,4-dione (**4al**), and 5-Hydroxy-3-(2-methylbutyl)naphthalene-1,4-dione (**5al**)

The reaction was carried out following the general procedure: 5-Hydroxy-1,4-naphthoquinone **1a** (400 mg, 2.30 mmol), 3-methylvaleric acid **2l** (2.90 ml, 23.0 mmol), and sodium persulfate (1.06 g, 4.60 mmol) in 45 ml of DMSO/water (600:1 v/v) were heated under reflux for 87 h at 40°C. **3al**: Yield: 22% (125 mg); orange needles: mp 48°C. R_*f*_. 0.62 (toluene). IR (cm^−1^): 2970 (ν_C-H_), 1639 (ν_C=O_), 1454, 1605 (ν_C=CAr_), and 1229 (ν_C-O_). ^1^H NMR (300 MHz, chloroform-d) δ 0.88–0.98 (m, 6H, 2CH_3_), 1.18–1.52 (m, 2H, CH_2_), 1.68–1.74 (m, 1H, CH), 2.29 (dd, *J* = 8.2, 13.6 Hz, 1H, CH_2_), 2.61 (ddd, *J* = 1.0, 5.9, 13.6 Hz, 1H, CH_2_), 6.73 (s, 1H, CH), 7.22–7.26 (m, 1H, CH_Ar_), 7.65–7.56 (m, 2H, CH_Ar_) and 11.98 (s, 1H, OH). ^13^C NMR (75 MHz, chloroform-d) δ 11.48 (CH_3_), 19.23 (CH_3_), 29.73 (CH_2_), 34.25 (CH), 37.11 (CH_2_), 115.20 (C_Ar_), 119.49 (CH_Ar_), 124.20 (CH_Ar_), 132.39 (C_Ar_), 135.75 (CH), 136.23 (CH_Ar_), 152.63 (C), 161.26 (C-OH), 184.72 (C=O), and 190.49 (C=O). HPLC: 16.52 min; purity: 95%. HRMS: [M + H]^+^ calcd for C_15_H_17_O_3_: 245.1172; found: 245.1177. **4al**: Yield: 12% (86 mg); orange oil. R_*f*_. 0.76 (toluene). IR (cm^−1^): 2959 (ν_C-H_), 1629 (ν_C=O_), 1457, 1599 (ν_C=CAr_), 1225 (ν_C-O_). On NMR, a mixture of two diastereoisomers was observed resulting in the presence of additional signals.

^1^H NMR (300 MHz, chloroform-d) δ 0.82–0.99 (m, 12H, 4CH_3_), 1.17–1.31 (m, 2H, CH_2_), 1.35–1.51 (m, 2H, CH_2_), 1.58–1.75 (m, 2H, 2CH), 2.39–2.55 (m, 2H, CH_2_), 2.58–2.75 (m, 2H, CH_2_), 7.20 (dd, *J* = 1.9, 7.6 Hz, 1H, CH_Ar_), 7.50–7.64 (m, 2H, CH_Ar_), and 12.25 (s, 1H, OH). ^13^C NMR (75 MHz, chloroform-d) δ 11.76 (2CH_3_), 19.38 (2CH_3_), 30.12 (CH_2_), 30.19 (CH_2_), 33.47 (CH_2_), 33.54 (CH_2_), 34.16 (CH_2_), 34.23 (CH_2_), 35.69 (2CH), 115.25 (C_Ar_), 118.92 (CH_Ar_), 123.73 (CH_Ar_), 132.48 (C_Ar_), 135.93 (CH), 147.28 (C_Ar_), 149.10 (C), 161.30 (C-OH), 184.65 (C=O), and 190.74 (C=O). HPLC: *t*_*R*_: 20.96 min; purity: 98%. HRMS: [M + H]^+^ calcd for C_20_H_27_O_3_: 315.1955; found: 315.1963. **5al**: Yield: 11% (59 mg); orange needles: mp 65–67°C. R_*f*_. 0.58 (toluene). IR (cm^−1^): 2925, 2851 (ν_C-H_), 1640 (ν_C=O_), 1446, 1609 (ν_C=CAr_), and 1252 (ν_C-O_). ^1^H NMR (300 MHz, chloroform-d) δ 0.88–0.98 (m, 6H, 2CH_3_), 1.18–1.52 (m, 2H, CH_2_), 1.67–1.77 (m, 1H, CH), 2.29 (ddd, *J* = 1.1, 8.2, 13.7 Hz, 1H, CH_2_), 2.61 (ddd, *J* = 1.2, 6.1, 13.6 Hz, 1H, CH_2_), 6.74 (s, 1H, CH), 7.22–7.26 (m, 1H, CH_Ar_), 7.54–7.64 (m, 2H, CH_Ar_), and 12.13 (s, 1H, OH). ^13^C NMR (75 MHz, chloroform-d) δ 11.47 (CH_3_), 19.21 (CH_3_), 29.70 (CH_2_), 34.24 (CH), 36.52 (CH_2_), 115.33 (C_Ar_), 118.78 (CH_Ar_), 124.31 (CH_Ar_), 132.31 (C_Ar_), 136.47 (CH), 136.86 (CH_Ar_), 150.95 (C), 161.73 (C-OH), 184.46 (C=O), and 190.85 (C=O). HPLC: *t*_*R*_: 16.42 min; purity: 95%. HRMS: [M + H]^+^ calcd for C_15_H_17_O_3_: 245.1172; found: 245.1175.

#### 2-Cyclohexyl-5-hydroxynaphthalene-1,4-dione (**3am**) and 3-Cyclohexyl-5-hydroxynaphthalene-1,4-dione (**5am**)

The reaction was carried out following the general procedure: 5-Hydroxy-1,4-naphthoquinone **1a** (304 mg, 1.73 mmol), cyclohexanecarboxylic acid **2m** (2.22 g, 17.2 mmol), and sodium persulfate (788 mg, 3.47 mmol) in 35 ml of DMSO/water (600:1 v/v) were heated under reflux for 22 h at 40°C. **3am**: Yield: 24% (67 mg); orange needles: mp 102°C. R_*f*_. 0.42 (1:1 PE 40–60°C/toluene). IR (cm^−1^): 2974, 2943 (ν_C-H_), 1636 (ν_C=O_), 1451, 1605 (ν_C=CAr_), and 1208 (ν_C-O_). ^1^H NMR (300 MHz, chloroform-d) 1.12–1.51 (m, 6H, 3CH_2_), 1.79–1.90 (m, 4H, 2CH_2_), 2.88 (t, *J* = 11.8 Hz, 1H, CH_2_), 6.67 (s, 1H, CH), 7.21 (dd, *J* = 2.1, 7.4 Hz, 1H, CH_Ar_), 7.53–7.63 (m, 2H, CH_Ar_), and 11.96 (s, 1H, OH). ^13^C NMR (75 MHz, chloroform-d) δ 26.12 (CH_2_), 26.46 (2CH_2_), 32.32 (2CH_2_), 36.94 (CH), 114.95 (C_Ar_), 119.46 (CH_Ar_), 124.05 (CH_Ar_), 132.55 (C_Ar_), 132.98 (CH), 136.17 (CH_Ar_), 157.87 (C), 161.15 (C-OH), 184.10 (C=O), and 190.93 (C=O). HPLC: *t*_*R*_: 17.04 min; purity: 100%. HRMS: [M + H]^+^ calcd for C_16_H_17_O_3_: 257.1159; found: 257.1174. **5am**: Yield: 8% (33 mg); orange needles: mp 131°C. R_*f*_. 0.31 (1:1 PE 40–60°C/toluene). IR (cm^−1^): 2916, 2851 (ν_C-H_), 1633 (ν_C=O_), 1457, 1609 (ν_C=CAr_), and 1225 (ν_C-O_). ^1^H NMR (300 MHz, chloroform-d) δ 1.16–1.53 (m, 6H, 3CH_2_), 1.80–1.91 (m, 4H, 2CH_2_), 2.88 (t, *J* = 11.8 Hz, 1H, CH), 6.70 (d, *J* = 0.9 Hz, 1H, CH), 7.20–7.26 (m, 1H, CH_Ar_), 7.53–7.63 (m, 2H, CH_Ar_), and 12.16 (s, 1H, OH). ^13^C NMR (75 MHz, chloroform-d) δ 26.13 (CH_2_), 26.52 (2CH_2_), 32.38 (2CH_2_), 36.39 (CH), 115.47 (C_Ar_), 118.70 (CH_Ar_), 124.31 (CH_Ar_), 132.10 (C_Ar_), 134.05 (CH), 136.41 (CH_Ar_), 156.29 (C), 161.78 (C-OH), 184.93 (C=O), and 190.37 (C=O). HPLC: *t*_*R*_: 16.86 min; purity: 97%. HRMS: [M + H]^+^ calcd for C_16_H_17_O_3_: 257.1172; found: 257.1177.

#### 2-Hexyl-5,8-dihydroxynaphthalene-1,4-dione (**3bc**)

The reaction was carried out following the general procedure: 5,8-Dihydroxy-1,4-naphthoquinone **1b** (330 mg, 1.58 mmol), heptanoic acid **2c** (2.26 ml, 15.8 mmol), and sodium persulfate (721 mg, 3.16 mmol) in 35 ml of DMSO/water (600:1 v/v) were heated under reflux for 15 h at 65°C. Yield: 22% (94 mg); dark red needles: mp 96–97°C. R_*f*_. 0.38 (1:1 PE 40–60°C/toluene). IR (cm^−1^): 2926 (ν_C-H_), 1597 (ν_C=O_), 1453: 1597 (ν_C=CAr_), and 1239 (ν_C-O_). ^1^H NMR (300 MHz, chloroform-d) δ 0.89 (t, *J* = 6.7 Hz, 3H, CH_3_), 1.27–1.43 (m, 6H, 3CH_2_), 1.52–1.64 (m, 2H, CH_2_), 2.54–2.61 (m, 2H, CH_2_), 6.82 (t, *J* = 1.1 Hz, 1H, CH), 7.17 (s, 2H, CH_Ar_), 12.44 (s, 1H, OH), and 12.59 (s, 1H, OH). ^13^C NMR (75 MHz, chloroform-d) δ 14.17 (CH_3_), 22.65 (CH_2_), 28.18 (CH_2_), 29.16 (CH_2_), 29.49 (CH_2_), 31.66 (CH_2_), 111.77 (C_Ar_), 112.03 (C_Ar_), 130.82 (CH_Ar_), 131.15 (CH_Ar_), 134.37 (2CH), 152.35 (2C), 162.06 (C-OH), 162.74 (C-OH), 183.37 (C=O), and 183.39 (C=O). HPLC: *t*_*R*_: 18.73 min; purity: 95%. HRMS: [M + H]^+^ calcd for C_16_H_19_O_4_: 275.1283; found: 275.1295.

#### 2,3-Diheptyl-5,8-dihydroxynaphthalene-1,4-dione (**4bd**)

The reaction was carried out following the general procedure: 5,8-Dihydroxy-1,4-naphthoquinone **1b** (331 mg, 1.58 mmol), octanoic acid **2d** (2.50 ml, 15.8 mmol), and sodium persulfate (2.44 g, 10.6 mmol) in 35 ml of DMSO/water (600:1 v/v) were heated under reflux for 13.5 h at 65°C. Yield: 9% (56 mg); dark red needles: mp 72°C. R_*f*_. 0.14 (95:5 PE 40–60°C/toluene). IR (cm^−1^): 2924, 1605 (ν_C=O_), 2852 (ν_C-H_), 1396, 1605 (ν_C=CAr_), and 1171 (ν_C-O_). ^1^H NMR (300 MHz, chloroform-d) δ 0.84–0.94 (m, 3H, CH_3_), 1.23–1.61 (m, 10H, 5CH_2_), 2.56–2.66 (m, 2H, CH_2_), 7.20 (s, 1H, CH_Ar_), and 12.71 (s, 1H, OH). ^13^C NMR (75 MHz, chloroform-d) δ 14.24 (CH_3_), 22.79 (CH_2_), 26.83 (CH_2_), 29.19 (CH_2_), 29.76 (CH_2_), 30.25 (CH_2_), 31.89 (CH_2_), 111.99 (C_Ar_), 129.36 (CH_Ar_), 148.41 (C), 158.60 (C-OH), and 186.99 (C=O). HPLC: *t*_*R*_: 21.08 min; purity: 99%. HRMS: [M + H]^+^ calcd for C_24_H_35_O_4_: 387.2543; found: 387.2526.

#### 5,8-Dihydroxy-2-octylnaphthalene-1,4-dione (**3be**)

The reaction was carried out following the general procedure: 5,8-Dihydroxy-1,4-naphthoquinone **1b** (333 mg, 1.58 mmol), nonanoic acid **2e** (2.77 ml, 15.8 mmol), and sodium persulfate (721 mg, 13.6 mmol) in 35 ml of DMSO/water (600:1 v/v) were heated under reflux for 17 h at 65°C. Yield: 40% (190 mg); dark red needles: mp 89°C. R_*f*_. 0.46 (1:1 PE 40–60°C/toluene). IR (cm^−1^): 2925 (ν_C-H_), 1567 (ν_C=O_), 1411 (ν_C=CAr_), and 1208 (ν_C-O_). ^1^H NMR (300 MHz, chloroform-d) δ 0.86 (t, *J* = 6.6 Hz, 3H, CH_3_), 1.22–1.42 (m, 10H, 5CH_2_), 1.51–1.67 (m, 2H, CH_2_), 2.52–2.63 (m, 2H, CH_2_), 6.82 (s, 1H, CH), 7.17 (s, 2H, 2CH_Ar_), 12.44 (s, 1H, OH), and 12.59 (s, 1H, OH). ^13^C NMR (75 MHz, chloroform-d) δ 14.19 (CH_3_), 22.75 (CH_2_), 29.18 (CH_2_), 29.21 (CH_2_), 29.29 (CH_2_), 29.44 (2CH_2_), 29.49 (CH_2_), 31.93 (CH_2_), 111.77 (C_Ar_), 112.02 (C_Ar_), 130.83 (CH_Ar_), 131.15 (CH_Ar_), 134.36 (CH), 152.36 (C), 162.08 (C-OH), 162.76 (C-OH), 180.28 (C=O), and 183.36 (C=O). HPLC: *t*_*R*_: 20.19 min; purity: 97%. [M + H]^+^ calcd for C_18_H_23_O_4_: 303.1591; found 303.1602.

#### 5,8-Dihydroxy-2,3-dinonylnaphthalene-1,4-dione (**4bf**)

The reaction was carried out following the general procedure: 5,8-Dihydroxy-1,4-naphthoquinone **1b** (330 mg, 1.58 mmol), decanoic acid **2f** (2.72 g, 15.8 mmol), and sodium persulfate (2.43 g, 10.6 mmol) in 35 ml of DMSO/water (600:1 v/v) were heated under reflux for 13.5 h at 65°C. Yield: 6% (41 mg); dark red needles: mp 70°C. R_*f*_. 0.11 (95:5 PE 40–60°C/toluene). IR (cm^−1^): 2915 (ν_C-H_), 1601 (ν_C=O_), 1457, 1601 (ν_C=CAr_), and 1181 (ν_C-O_). Doubling of some signals on ^13^C NMR was reported for this molecule and can be explained by the presence of polymorphs as described by Olivieri et al. (1990).

^1^H NMR (300 MHz, chloroform-d) δ 0.88 (t, *J* = 6.7 Hz, 3H, CH_3_), 1.22–1.43 (m, 12H, 6CH_2_), 1.56–1.62 (m, 2H, CH_2_), 2.58–2.66 (m, 2H, CH_2_), 6.92 (s, 1H, CH_Ar_), and 12.84 (s, 1H, OH). ^13^C NMR (75 MHz, chloroform-d) δ 14.25 (2CH_3_), 22.81 (2CH_2_), 26.82 (2CH_2_), 29.45 (2CH_2_), 29.53 (2CH_2_), 29.63 (2CH_2_), 29.75 (2CH_2_), 29.83 (2CH_2_), 32.02 (2CH_2_), 111.98 (2C_Ar_), 129.34 (2CH_Ar_), 148.40 (2C), 158.58 (2C-OH), 172.53 (C=O), and 173.10 (C=O). HPLC: *t*_*R*_: 33.64 min; purity: 100%. [M + H]^+^ calcd for C_28_H_43_O_4_: 443.3161; found: 443.3158.

#### 2-(Heptan-4-yl)-5,8-dihydroxynaphthalene-1,4-dione (**3bg**)

The reaction was carried out following the general procedure: 5,8-Dihydroxy-1,4-naphthoquinone **1b** (441 mg, 2.09 mmol), 2-propylpentanoic acid **2g** (3.35 g, 21.0 mmol), and sodium persulfate (960 mg, 4.21 mmol) in 42 ml of DMSO/water (600:1 v/v) were heated under reflux for 13.5 h at 65°C. Yield: 23% (139 mg); dark red needles: mp 100–102°C. R_*f*_. 0.76 (toluene). IR (cm^−1^): 2972, 2855 (ν_C-H_), 1406 (ν_C=CAr_), and 1228 (ν_C-O_). ^1^H NMR (300 MHz, chloroform-d) δ 0.88 (t, *J* = 6.7 Hz, 6H, 2CH_3_), 1.22–1.43 (m, 4H, 2CH_2_), 1.56–1.67 (m, 4H, 2CH_2_), 3.08–3.21 (m, 1H, CH), 6.82 (s, 1H, CH), 7.20 (s, 2H, 2CH_Ar_), 12.49 (s, 1H, OH), and 12.72 (s, 1H, OH). ^13^C NMR (75 MHz, chloroform-d) δ 14.24 (2CH_3_), 20.66 (2CH_2_), 37.04 (CH), 37.19 (2CH_2_), 111.68 (C_Ar_), 112.15 (C_Ar_), 131.15 (CH_Ar_), 131.30 (CH_Ar_), 133.59 (CH), 156.06 (C), 162.68 (C-OH), 163.50 (C-OH), 182.81 (C=O), and 182.98 (C=O). HPLC: *t*_*R*_: 18.48 min; purity: 98%. [M + H]^+^ calcd for C_17_H_21_O_4_: 289.1434; found: 289.1437.

#### 2-(Sec-butyl)-5,8-dihydroxynaphthalene-1,4-dione (**3bh**)

The reaction was carried out following the general procedure: 5,8-Dihydroxy-1,4-naphthoquinone **1b** (336 mg, 1.58 mmol), 2-methylbutyric acid **2h** (1.72 ml, 15.8 mmol), and sodium persulfate (721 mg, 3.16 mmol) in 35 ml of DMSO/water (600:1 v/v) were heated under reflux for 87 h at 65°C. Yield: 42% (163 mg); dark red needles: mp 114°C. R_*f*_. 0.68 (1:1 PE 40–60°C/toluene). IR (cm^−1^): 2925 (ν_C-H_), 1601 (ν_C=O_), 1411, 1567 (ν_C=CAr_), and 1208 (ν_C-O_). ^1^H NMR (300 MHz, chloroform-d) δ 0.91 (t, *J* = 7.4 Hz, 3H, CH_3_), 1.18 (d, *J* = 6.9 Hz, 3H, CH_3_), 1.42–1.72 (m, 2H, CH_2_), 3.02–3.15 (m, 1H, CH), 6.79 (s, 1H, CH), 7.17 (s, 2H, CH_Ar_), 12.44 (s, 1H, OH), and 12.66 (s, 1H, OH). ^13^C NMR (75 MHz, chloroform-d) δ 11.86 (CH_3_), 19.22 (CH_3_), 28.90 (CH_2_), 33.39 (CH), 111.60 (C_Ar_), 112.14 (C_Ar_), 130.81 (CH_Ar_), 130.97 (CH_Ar_), 132.97 (CH), 156.86 (C), 161.85 (C-OH), 162.62 (C-OH), 183.25 (C=O), and 183.70 (C=O). HPLC: *t*_*R*_: 16.44 min; purity: 96%. [M + H]^+^ calcd for C14H15O4: 247.0965, found 247.0972.

#### 5,8-Dihydroxy-2-(tert-pentyl)naphthalene-1,4-dione (3bi) and 5,8-Dihydroxy-2,3-di-tert-pentylnaphthalene-1,4-dione (**4bi**)

The reaction was carried out following the general procedure: 5,8-Dihydroxy-1,4-naphthoquinone **1b** (333 mg, 1.58 mmol), 2,2-dimethylbutyric acid **2i** (1.98 ml, 15.8 mmol), and sodium persulfate (2.45 g, 10.6 mmol) in 35 ml of DMSO/water (600:1 v/v) were heated under reflux for 15.5 h at 65°C. **3bi**: Yield: 3% (29 mg); dark red needles: mp 143°C. R_*f*_. 0.44 1:1 PE 40–60°C/toluene). IR (cm^−1^): 2924 (ν_C-H_), 1653 (ν_C=O_), 1460, 1598 (ν_C=CAr_), and 1191 (ν_C-O_). ^1^H NMR (300 MHz, chloroform-d) δ 0.73 (t, *J* = 7.5 Hz, 3H, CH_3_), 1.33 (s, 6H, 2CH_3_), 1.91 (q, *J* = 7.5 Hz, 2H, CH_2_), 6.86 (s, 1H, CH), 7.21 (s, 2H, CH_Ar_), 12.43 (s, 1H, OH), and 12.92 (s, 1H, OH). ^13^C NMR (75 MHz, chloroform-d) δ 9.64 (CH_3_), 27.53 (2CH_3_), 33.42 (CH_2_), 39.99 (C), 111.84 (C_Ar_), 112.82 (C_Ar_), 130.09 (CH_Ar_), 130.72 (CH_Ar_), 135.60 (CH), 158.06 (C), 160.28 (C-OH), 161.50 (C-OH), 185.10 (C=O), and 185.67 (C=O). HPLC: *t*_*R*_: 17.66 min; purity: 95%. [M + H]^+^ calcd for C_15_H_17_O_4_: 261.1127; found: 261.1126. **4bi**: Yield: 11% (59 mg); dark red needles: mp 125°C. R_*f*_. 0.44 (PE/toluene: 50/50 v/v). IR (cm^−1^): 2962 (ν_C-H_), 1594 (ν_C=O_), 1404, 1594 (ν_C=CAr_), and 1214 (ν_C-O_). Doubling of some signals on ^13^C NMR was reported for this molecule and can be explained by the presence of polymorphs as described by Olivieri et al. (1990).

^1^H NMR (300 MHz, chloroform-d) δ 0.70 (t, *J* = 7.5 Hz, 3H, CH_3_), 1.34 (s, 6H, 2CH_3_), 1.93 (q, *J* = 7.5 Hz, 2H, CH_2_), 6.98 (s, 1H, CH_Ar_), and 13.36 (s, 1H, OH). ^13^C NMR (75 MHz, chloroform-d) δ 9.64 (2CH_3_), 27.47 (4CH_3_), 33.11 (2CH_2_), 39.92 (2C), 111.41 (2C_Ar_), 132.25 (2CH_Ar_), 154.63 (2C), 173.23 (2C-OH), and 173.51 (2C=O). HPLC: *t*_*R*_: 21.89 min; purity: 100%. [M + H]^+^ calcd for C_20_H_27_O_4_: 331.1909; found: 331.1917.

#### 5,8-Dihydroxy-2-neopentylnaphthalene-1,4-dione (**3bj**) and 5,8-Dihydroxy-2,3-dineopentylnaphthalene-1,4-dione (**4bj**)

The reaction was carried out following the general procedure: 5,8-Dihydroxy-1,4-naphthoquinone **1b** (332 mg, 1.58 mmol), 3:3-dimethylbutyric acid **2j** (2.01 ml, 15.8 mmol), and sodium persulfate (2.45 g, 10.6 mmol) in 35 ml of DMSO/water (600:1 v/v) were heated under reflux for 13.5 h at 65°C. **3bj**: Yield: 8% (32 mg); dark red needles: mp 127°C. R_*f*_. 0.44 (1:1 PE 40–60°C/toluene). IR (cm^−1^): 2953 (ν_C-H_), 1457, 1595 (ν_C=CAr_), and 1212 (ν_C-O_). ^1^H NMR (300 MHz, chloroform-d) δ 0.96 (s, 9H, 3CH_3_), 2.54 (s, 2H, CH_2_), 6.84 (s, 1H, CH), 7.18 (s, 2H, 2CH_Ar_), 12.45 (s, 1H, OH), and 12.68 (s, 1H, OH). ^13^C NMR (75 MHz, chloroform-d) δ 29.79 (3CH_3_), 32.91 (C), 41.52 (CH_2_), 111.92 (C_Ar_), 111.98 (C_Ar_), 131.31 (CH_Ar_), 131.45 (CH_Ar_), 136.84 (CH), 149.66 (C), 163.14 (C-OH), 163.86 (C-OH), 181.95 (C=O), and 182.65 (C=O). HPLC: *t*_*R*_: 17.60 min; purity: 97%. [M + H]^+^ calcd for C_15_H_17_O_4_: 261.1127; found: 261.1126. **4bj**: Yield: 6% (33 mg); dark red needles: mp 126°C. R_*f*_. 0.61 (1:1 PE 40–60°C/toluene). IR (cm^−1^): 2954 (ν_C-H_), 1598 (ν_C=O_), 1412, 1599 (ν_C=CAr_), and 1200 (ν_C-O_). Doubling of some signals on ^13^C NMR was reported for this molecule and can be explained by the presence of polymorphs as described by Olivieri et al. (1990).

^1^H NMR (300 MHz, chloroform-d) δ 0.97 (s, 18H, 6CH_3_), 2.58 (s, 4H, 2CH_2_), 6.92 (s, 2H, 2CH_Ar_), 12.55 (s, 1H, OH), and 13.27 (s, 1H, OH). ^13^C NMR (75 MHz, chloroform-d) δ 29.76 (6CH_3_), 32.98 (2C), 41.91 (2CH_2_), 110.99 (C_Ar_), 111.36 (C_Ar_), 134.47 (2CH_Ar_), 146.83 (2C), 171.96 (2C-OH), and 173.51 (2C=O). HPLC: *t*_*R*_: 21.44 min; purity: 96%. [M + H]^+^ calcd for C_20_H_27_O_4_: 331.1909; found: 331.1916.

#### 5,8-Dihydroxy-2-isobutylnaphthalene-1,4-dione (**3bk**) and 5,8-Dihydroxy-2,3-diisobutylnaphthalene-1,4-dione (**4bk**)

The reaction was carried out following the general procedure: 5,8-Dihydroxy-1,4-naphthoquinone **1b** (336 mg, 1.58 mmol), isovaleric acid **2k** (1.72 ml, 15.8 mmol), and sodium persulfate (730 mg, 3.16 mmol) in 35 ml of DMSO/water (600:1 v/v) were heated under reflux for 15 h at 65°C. **3bk**: Yield: 16% (36 mg); dark red needles: mp 96°C. R_*f*_. 0.44 (1:1 PE 40–60°C/toluene). IR (cm^−1^): 2928 (ν_C-H_), 1641 (ν_C=O_), 1452, 1608 (ν_C=CAr_), and 1253 (ν_C-O_). ^1^H NMR (300 MHz, chloroform-d) δ 0.96 (d, *J* = 6.6 Hz, 6H, 2CH_3_), 1.85–2.01 (m, 1H, CH), 2.46 (dd, *J* = 1.0, 7.1 Hz, 2H, CH_2_), 6.82 (s, 1H, CH), 7.18 (s, 2H, CH_Ar_), 12.45 (s, 1H, OH), and 12.62 (s, 1H, OH). ^13^C NMR (75 MHz, chloroform-d) δ 22.64 (2CH_3_), 28.03 (CH), 38.73 (CH_2_), 111.83 (C_Ar_), 112.02 (C_Ar_), 131.07 (CH_Ar_), 131.36 (CH_Ar_), 135.34 (CH), 151.00 (C), 162.68 (C-OH), 163.37 (C-OH), 182.65 (C=O), and 182.91 (C=O). HPLC: *t*_*R*_: 16.93 min; purity: 97%. [M + H]^+^ calcd for C_14_H_15_O_4_: 247.0965; found 247.0972. **4bk**: Yield: 3% (12 mg); dark red needles: mp 82°C. R_*f*_. 0.74 (1:1 PE 40–60°C/toluene). IR (cm^−1^): 2925, 2851 (ν_C-H_), 1640 (ν_C=O_), 1446, 1609 (ν_C=CAr_), and 1252 (ν_C-O_). ^1^H NMR (300 MHz, chloroform-d) δ 0.96 (d, *J* = 6.7 Hz, 6H, 2CH_3_), 1.82–2.04 (m, 1H, CH), 2.59 (d, *J* = 7.2 Hz, 2H, CH_2_), 7.21 (s, 1H, CH_Ar_), and 12.73 (s, 1H, OH). ^13^C NMR (75 MHz, chloroform-d) δ 22.94 (2CH_3_), 29.35 (CH), 35.48 (CH_2_), 111.96 (C_Ar_), 129.39 (CH_Ar_), 148.36 (C), 158.67 (C-OH), and 187.04 (C=O). HPLC: *t*_*R*_: 19.78 min; purity: 96%. [M + H]^+^ calcd for C_18_H_23_O_4_: 303.1591; found: 303.1604.

#### 5,8-Dihydroxy-2,3-bis(2-methylbutyl)naphthalene-1,4-dione (**4bl**)

The reaction was carried out following the general procedure: 5,8-Dihydroxy-1,4-naphthoquinone **1b** (334 mg, 1.58 mmol), 3-methylvaleric acid **2l** (1.97 ml, 15.8 mmol), and sodium persulfate (2.43 g, 10.6 mmol) in 35 ml of DMSO/water (600:1 v/v) were heated under reflux for 15.5 h at 65°C. Yield: 11% (54 mg); dark red needles: mp 45°C. R_*f*_. 0.65 (1:1 PE 40–60°C/toluene). IR (cm^−1^): 2961 (ν_C-H_), 1597 (ν_C=O_), 1435, 1597 (ν_C=CAr_), and 1225 (ν_C-O_). On NMR, a mixture of two diastereoisomers was observed resulting in the presence of additional signals. ^1^H NMR (300 MHz, chloroform-d) δ 0.83–0.98 (m, 6H, 2CH_3_), 1.15–1.50 (m, 2H, CH_2_), 1.69–1.73 (m, 1H, CH), 2.42–2.78 (m, 2H, CH_2_), 7.20 (s, 1H, CH_Ar_), and 12.74 (s, 1H, OH). ^13^C NMR (75 MHz, chloroform-d) δ 11.79 (2CH_3_), 19.35 (CH_3_), 19.40 (CH_3_), 30.12 (CH_2_), 30.20 (CH_2_), 33.70 (CH_2_), 33.79 (CH_2_), 35.74 (2CH), 111.98 (2C_Ar_), 129.35 (2CH_Ar_), 148.62 (2C), 158.65 (2C-OH), and 187.05 (2C=O) HPLC: *t*_*R*_: 21.50 min; purity: 95%. [M + H]^+^ calcd for C_20_H_27_O_4_: 331.1904; found: 331.1913.

#### 2-Cyclohexyl-5,8-dihydroxynaphthalene-1,4-dione (**3bm**)

The reaction was carried out following the general procedure: 5,8-Dihydroxy-1,4-naphthoquinone **1b** (442 mg, 2.10 mmol), cyclohexanecarboxylic acid **2m** (2.65 mg, 21.0 mmol), and sodium persulfate (961 g, 4.21 mmol) in 35 ml of DMSO/water (600:1 v/v) were heated under reflux for 65 h at 40°C. Yield: 33% (130 mg); dark red needles: mp 188–190 °C. R_*f*_. 0.65 (toluene). IR (cm^−1^): 2927, 2854 (ν_C-H_), 1607 (ν_C=O_), 1403, 1607 (ν_C=CAr_), and 1208 (ν_C-O_). ^1^H NMR (300 MHz, chloroform-d) δ 1.15–1.53 (m, 6H, 3CH_2_), 1.79–1.92 (m, 4H, 2CH_2_), 2.87–3.01 (m, 1H, CH), 6.81 (d, *J* = 0.7 Hz, 1H, CH), 7.20 (s, 2H, 2CH_Ar_), 12.48 (s, 1H, OH), and 12.68 (s, 1H, OH). ^13^C NMR (75 MHz, chloroform-d) δ 26.15 (CH_2_), 26.55 (CH_2_), 32.44 (CH_2_), 36.75 (CH), 111.71 (C_Ar_), 112.26 (C_Ar_), 130.66 (CH_Ar_), 130.86 (CH_Ar_), 132.90 (CH), 156.94 (C), 161.34 (C-OH), 162.12 (C-OH), 183.83 (C=O), and 184.51 (C=O). HPLC: *t*_*R*_: 17.13 min; purity: 95%. [M + H]^+^ calcd for C_16_H_17_O_4_: 273.1121; found: 273.1121.

### X-Ray Crystallographic Studies

The X-ray single crystal data of **5ae** and **3bg** were collected with monochromated Mo-Kα radiation (0.71073 Å) on a Bruker Apex3 CCD diffractometer. Intensity data were collected at 123 K using the θ-scan method. Data reduction of each compound was carried out using Bruker SAINT software. Multi-scan absorption correction was applied to all intensity data using the SADABS 2016/2 program (Krause et al., 2015). The structures were solved by a combination of direct methods with SHELXT-2014/5 and refined with full-matrix least-squares based on F^2^ using SHELXL 2018/3 (Sheldrick, 2015). The hydrogen atoms were treated by a mixture of independent and constrained refinement. Molecular and the crystal packing diagrams were drawn with Mercury software. Bruker SHELXTL was used to prepare material for publication and realize molecular graphics. All of the crystal data are described in supporting information. The crystal structures were deposited at the Cambridge Crystallographic Database Centre (CCDC) and given the numbers CCDC 2083158 and 2083159.

### Biological Activity

#### Antibacterial Assays

All used bacteria were donated by the CHU of Nîmes and were cultivated in Mueller–Hinton broth (MHB) in this study. For plating bacteria, 1.7% agar was added to the respective broth. Briefly, six bacterial strains were cultivated. One reference Gram-positive strain: *Staphylococcus aureus* (ATCC 29213), and two clinical Gram-positive strains isolated from a patient and given by the Nîmes University Hospital: sensitive *Staphylococcus aureus*, NSASM-1, and resistant *Staphylococcus aureus*, NSARM-1, were used. NSASM-1 is a sensitive strain to reference antibiotics cloxacillin or vancomycin, and NSARM-1 is a *S. aureus* strain and is considered as resistant because of the high cloxacillin MIC (breakpoint of 2 μg/ml); hence, this strain is resistant to the penicillin family but not to the glycopeptide family like vancomycin (breakpoint of 2 μg/ml). Two reference Gram-negative strains: *Escherichia coli* (ATCC 25922) and *Pseudomonas aeruginosa* (PAO1) and two clinical Gram-negative strains isolated from a patient and given by the Nîmes University Hospital: sensitive *Escherichia* coli, NEC-S1, and resistant *Escherichia coli*, NEC-R3 (extended spectrum beta lactamase (ESBL)), were used. NEC-S1 is the sensitive *E. coli* strain to the reference antibiotics cefotaxime. NEC-R3 is the *E. coli* strain considered as resistant because of the high cefotaxime MIC (breakpoint of 2 μg/ml). This strain is an ESBL-producing bacteria and known to be resistant to most beta-lactam antibiotics. All bacteria were routinely grown at 37°C under agitation at 200 rpm. 5.12 mg of all compounds were solubilized in a 15-ml flask with 2 ml of DMSO and 8 ml of Mueller–Hinton broth (C_m_ = 512 µg/ml). Then serial dilutions of 1:2 were realized from this solution (0.5 < C_m_ < 256 µg/ml). The final concentration in the wells was 0.25–128 µg/ml. The MIC of the tested compounds was determined by using the 2-fold serial broth (MHB medium) dilution method in 96-well (U-base TPP) plates according to the European Committee of Antimicrobial Susceptibility Testing (EUCAST) guidelines (The European Committee on Antimicrobial Susceptibility Testing, 2021). Briefly, each well contained a cell density of (3−7) × 10^5^ CFU/ml and compound concentrations ranging from 0.5 to 128 μg/ml. After incubation for 20 h at 37°C, MICs were read as the lowest concentration of the compound that completely inhibited growth.

#### Determination of the Fractional Inhibitory Concentration Index

The fractional inhibitory index of most active compounds (**5ag**, **3al**, **5am**, **3bg**, and **3bm**) was determined as follows. To test the effect of adjuvants on bacterial resistance, a clinical bacterial strain was selected: *Staphylococcus aureus* isolate NSARM-1. The MICs of a panel of antibiotics including cloxacillin (CXL) and vancomycin (VCM) were determined by the broth microdilution method as described by EUCAST (The European Committee on Antimicrobial Susceptibility Testing, 2021). The bacterial inoculate was set at 5.10^5^ CFU/ml. The 96-well plates were used to allow concentrations of each antimicrobial to be varied along different axes (from 0.015 to 32 µg/ml for synthesized compounds and from 0.015 to 4 µg/ml for antibiotics), thus ensuring that each well of the plate represents a different combination. The synergistic effect was calculated with the fractional inhibitory concentration index (FICI), following the equation described below (Figure 5). Synergistic action can be considered when the FICI is below 0.5; the additivity effect when the FICI is between 0.5 and 1; indifference when the FICI is between 1 and 4; and antagonism when the FICI is below 4. All the plates were realized in duplicates. Absorption at 600 nm of each well was read with an EPOCH2C microplate reader, BioTek Instrument, Inc. (Highland Park, Winooski, VT.) with Gen5 software (version 2.06.10). The negative control average was considered as 100% inhibition, and the positive control average was considered as 0% inhibition. Heat maps were realized with RStudio software (see Supplementary Information).

### Hemolysis Assay

Hemolysis assay on juglone derivatives was performed according to previous reports with minor modifications (Kent et al., 1988; Mesdaghinia et al., 2019). After washing and resuspending in phosphate-buffered saline (PBS), 250 µl of 5% (v/v) of sheep erythrocyte solution was added to Eppendorf tubes, and then the same volume of compound was added at various concentrations (**3al**, **5ag**, **5am**, **3bg**, and **3bm**). Each hemolysis reaction contained 5% (v/v) RBCs, 5% (v/v) DMSO, and a specific concentration of the respective compounds. The samples were incubated for 1 h at 37°C under agitation at 100 rpm. 0.5% Triton X-100 and PBS were used as positive control and negative control, respectively. After incubation, the RBCs were separated from the reaction mixture by centrifugation at 1000 g and 4°C. The experiments were performed in triplicate. Hemolytic activities of the compounds or lack thereof was determined visually.

### ADME Predictions

*In silico* ADME studies were performed by using SwissADME (Daina et al., 2017). Structures of the compounds were uploaded into the SwissADME predictor website for further evaluations. Various pharmacokinetic and physicochemical properties were estimated for all compounds. The results of the compounds with the best MIC on MSSA and MRSA were shown previously (Table 6).

## Conclusion

With the extensive use and misuse of antibiotics, the problem of bacterial resistance is worrying and the development of new antibiotics is necessary. In this study, we synthesized a series of 29 juglone derivatives and 14 naphthazarin derivatives in one step. All the investigated compounds (**3**, **4**, and **5**) exhibited selective inhibitory activity against reference and clinical Gram-positive *S. aureus* strains and low to no activity against Gram-negative bacteria.

The juglone derivative **5am** was the most active against sensitive Gram-positive strains with a good MIC of 0.5 μg/ml. Compounds **5ag**, **5am**, **3bg**, and **3bm** displayed correct antibacterial activity with an MIC of 2 μg/ml against clinical resistant *S. aureus* strains. Additionally, **3bm** demonstrated very interesting synergistic actions with various conventional antibiotics against *S. aureus* NSARM-1 pathogens, thus indicating its ability as promising adjuncts to combination therapy.

Moreover, compound **3bm** did not exhibit hemolytic activity against sheep RBCs. *In silico* ADME evaluation in oral administration indicates that by linking physicochemical properties to the pharmacokinetic profile, these compounds present good bioavailability, and they are compatible with Lipinski’s “rule of five” which is often associated with the term “drug-like.” Based on the above studies, the newly synthesized juglone/naphthazarin derivatives were found to have promising antibacterial activity particularly on clinical resistant *S. aureus* and have the potential for further development, especially as adjuvant for a combination strategy between a reference antibiotic and **3bm**.

## Data Availability

The datasets presented in this study can be found in online repositories. The names of the repository/repositories and accession number(s) can be found at: https://www.ccdc.cam.ac.uk/ 2083159, 2083158.
