# Supplementary material for: Synthesis, Structure Elucidation, Antibacterial Activities, and Synergistic Effects of Novel Juglone and Naphthazarin Derivatives Against Clinical Methicillin-Resistant Staphylococcus aureus Strains
Source: Front Chem. 2021 Nov 19;9:773981. doi: 10.3389/fchem.2021.773981 (PMC8640087; doi:10.3389/fchem.2021.773981)

Supplementary Material

**Synthesis, structure elucidation, antibacterial activities and synergistic effects of novel juglone and naphthazarin derivatives against clinical Methicillin-Resistant *Staphylococcus aureus*** **strains**

Valentin Duvauchelle,^a^ Chaimae Majdi,^a^ David Bénimélis,^a^ Catherine Dunyach-Rémy,^b^ Patrick Meffre,^a^ and Zohra Benfodda^a*^

^a^UNIV. NIMES, EA 7352 CHROME, F-30021 Nîmes cedex 1, France

^b^Institut National de la Santé et de la Recherche Médicale, U1047, Montpellier University, CHU de Nîmes, Place du Pr R. Debré, 30029 Nîmes

*corresponding author: Zohra Benfodda; [zohra.benfodda@unimes.fr](mailto:zohra.benfodda@unimes.fr); University of Nîmes, CHROME laboratory, Rue du Dr. G. Salan, 30021 Nîmes Cedex 1, France.

|  |  | **Page** |
| --- | --- | --- |
| **1.** | **Structural elucidation of regioisomers using NMR HMBC spectroscopy** | S4 |
| **1.1** | Example of **3ah** and **5ah** |  |
|  |  |  |
| **2.** | **Heatmaps corresponding to synergistic evaluations** |  |
| **2.1** | Heatmaps for compounds **3al**, **5am**, **3bg** and **5ag** in combination with vancomycin. | S8 |
| **2.2** | Heatmaps for compounds **3al**, **5am**, **3bg** and **5ag** in combination with cloxacillin. | S9 |
| **3.** | **X-ray crystallographic supporting data** |  |
| **3.1** | X-ray crystallographic supporting data for **5ae** | S10 |
| **3.2** | X-ray crystallographic supporting data for **3bg** | S15 |
| **4.** | **Characterization of final compounds (^1^H, ^13^C, DEPT-135, HRMS, HPLC)** |  |
| **4.1** | *2-hexyl-5-hydroxynaphthalene-1,4-dione (****3ac****)* | S21 |
| **4.2** | *2,3-dihexyl-5-hydroxynaphthalene-1,4-dione (****4ac****)* | S26 |
| **4.3** | *3-hexyl-5-hydroxynaphthalene-1,4-dione* (**5ac**) | S31 |
| **4.4** | *2-heptyl-5-hydroxynaphthalene-1,4-dione (****3ad****),* | S36 |
| **4.5** | *2,3-diheptyl-5-hydroxynaphthalene-1,4-dione (****4ad****)* | S41 |
| **4.6** | *2-heptyl-8-hydroxynaphthalene-1,4-dione* (**5ad**) | S46 |
| **4.7** | *5-hydroxy-2-octylnaphthalene-1,4-dione* (**3ae**) | S51 |
| **4.8** | *5-hydroxy-2,3-dioctylnaphthalene-1,4-dione (****4ae****)* | S56 |
| **4.9** | *5-hydroxy-3-octylnaphthalene-1,4-dione* (**5ae**) | S61 |
| **4.10** | *5-hydroxy-2-nonylnaphthalene-1,4-dione (****3af****)* | S66 |
| **4.11** | *5-hydroxy-2,3-dinonylnaphthalene-1,4-dione (****4af****)* | S71 |
| **4.12** | *5-hydroxy-3-nonylnaphthalene-1,4-dione* (**5af**) | S76 |
| **4.13** | *2-(heptan-4-yl)-5-hydroxynaphthalene-1,4-dione (****3ag****)* | S81 |
| **4.14** | *2,3-di(heptan-4-yl)-5-hydroxynaphthalene-1,4-dione (****4ag****)* | S86 |
| **4.15** | *3-(heptan-4-yl)-5-hydroxynaphthalene-1,4-dione (****5ag****)* | S91 |
| **4.16** | *2-(sec-butyl)-5-hydroxynaphthalene-1,4-dione(****3ah****)* | S96 |
| **4.17** | *3-(sec-butyl)-5-hydroxynaphthalene-1,4-dione(****5ah****)* | S101 |
| **4.18** | *5-hydroxy-2-(tert-pentyl)naphthalene-1,4-dione (****3ai****,* | S106 |
| **4.19** | *8-hydroxy-2-(tert-pentyl)naphthalene-1,4-dione* (**5ai**) | S111 |
| **4.20** | *5-hydroxy-2,3-di-tert-pentylnaphthalene-1,4-dione (****3aj****)* | S116 |
| **4.21** | *5-hydroxy-3-neopentylnaphthalene-1,4-dione (****5aj****)* | S121 |
| **4.22** | *5-hydroxy-2-isobutylnaphthalene-1,4-dione* (**3ak**) | S126 |
| **4.23** | *5-hydroxy-2,3-diisobutylnaphthalene-1,4-dione (****4ak****)* | S131 |
| **4.24** | *5-hydroxy-3-isobutylnaphthalene-1,4-dione (****5ak****)* | S136 |
| **4.25** | *5-hydroxy-2-(2-methylbutyl)naphthalene-1,4-dione (****3al****)* | S141 |
| **4.26** | *5-hydroxy-2,3-bis(2-methylbutyl)naphthalene-1,4-dione (****4al****)* | S146 |
| **4.27** | *5-hydroxy-3-(2-methylbutyl)naphthalene-1,4-dione (****5al****)* | S151 |
| **4.28** | *2-cyclohexyl-5-hydroxynaphthalene-1,4-dione (****3am****)* | S156 |
| **4.29** | *3-cyclohexyl-5-hydroxynaphthalene-1,4-dione (****5am****)* | S161 |
| **4.30** | *2-hexyl-5,8-dihydroxynaphthalene-1,4-dione (****3bc****)* | S166 |
| **4.31** | *2,3-diheptyl-5,8-dihydroxynaphthalene-1,4-dione* (**4bd**) | S171 |
| **4.32** | *5,8-dihydroxy-2-octylnaphthalene-1,4-dione (****3be****)* | S176 |
| **4.33** | *5,8-dihydroxy-2,3-dinonylnaphthalene-1,4-dione (****4bf****)* | S181 |
| **4.34** | *2-(heptan-4-yl)-5,8-dihydroxynaphthalene-1,4-dione (****3bg****)* | S186 |
| **4.35** | *2-(sec-butyl)-5,8-dihydroxynaphthalene-1,4-dione (****3bh****)* | S191 |
| **4.36** | *5,8-dihydroxy-2-(tert-pentyl)naphthalene-1,4-dione (****3bi****)* | S196 |
| **4.37** | *5,8-dihydroxy-2,3-di-tert-pentylnaphthalene-1,4-dione (****4bi****)* | S201 |
| **4.38** | *5,8-dihydroxy-2-neopentylnaphthalene-1,4-dione* (***3bj***) | S206 |
| **4.39** | *5,8-dihydroxy-2,3-dineopentylnaphthalene-1,4-dione (****4bj****)* | S211 |
| **4.40** | *5,8-dihydroxy-2-isobutylnaphthalene-1,4-dione (****3bk****)* | S216 |
| **4.41** | *5,8-dihydroxy-2,3-diisobutylnaphthalene-1,4-dione (****4bk****)* | S221 |
| **4.42** | *5,8-dihydroxy-2,3-bis(2-methylbutyl)naphthalene-1,4-dione (****4bl****)* | S226 |
| **4.43** | *2-cyclohexyl-5,8-dihydroxynaphthalene-1,4-dione (****3bm****)* | S231 |

| 1. **Structural elucidation of regioisomers using NMR HMBC spectroscopy** |
| --- |
| 1.1 Example of **3ah** and **5ah** |
|  |

By means of Heteronuclear multiple bond correlation (HMBC) spectroscopy technic, we were able to determine either regioisomer **3ah** or **5ah** were purified (Fig S1.)

**Figure S1**. Structures of **3ah** and **5ah**

**
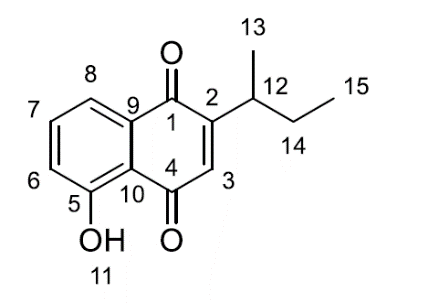
**

**3al**


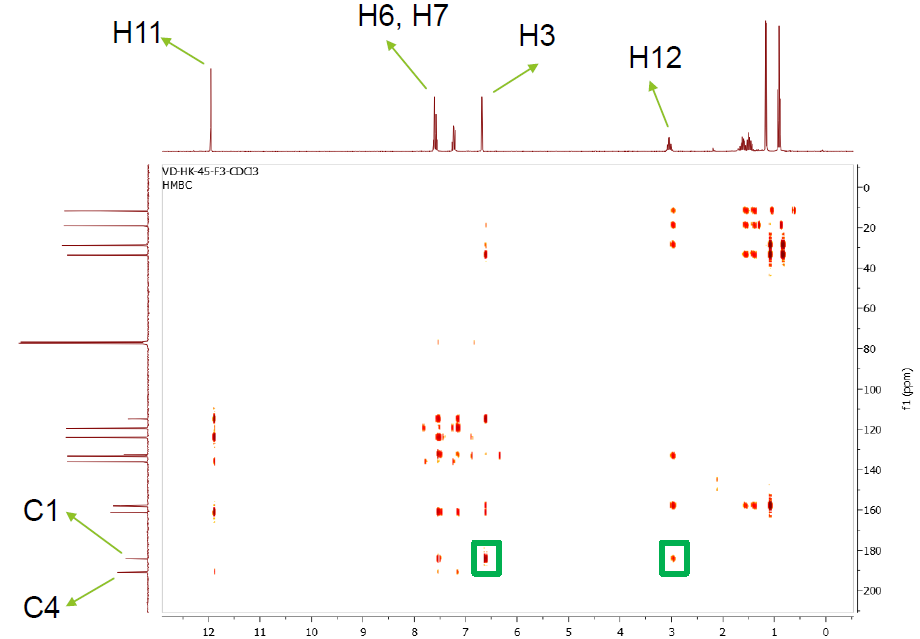


H7, H8

Figure S2*.* HMBC spectra of 3ah

3ah


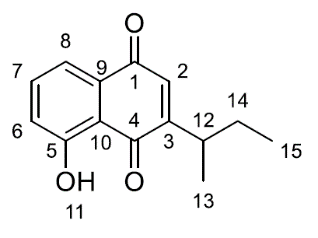


**5al**

H7, H8

5ah


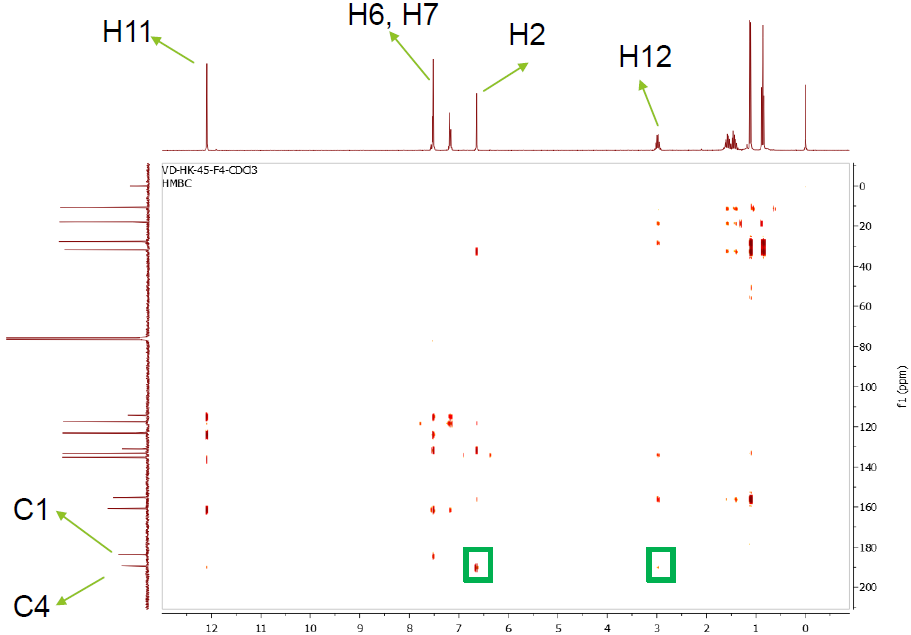


Figure S3*.* HMBC spectra of 5ah

On these two figures (Figure S2, S3) we can observe coupling between hydrogen atoms and carbon atoms at high distance. Firstly, we know the chemical shift of C1 and C4 because of correlation between H11 and C4. No correlation is possible between H11 and C1 because they are too far of each other. Then, are observed for **3ah** correlation between C1, H12 and between C1 and H3. It indicates proximity between these groups. No correlation is observed between C4 and H12 because of the too big distance between these two atoms. Concerning **5ah**, are observed correlation between C4 and H2 and between C4 and H12. It indicates proximity between these groups. No correlation is observed between C1 and H12 because of the too big distance between these two atoms. In both cases, we observe correlation between C1 and H8 because of their proximity. Correlation between H7, H8 and C4 is not possible because they are too far of each other. For all other regioisomers this method has been applied to determine the right structure by analogy. This technique alone is not sufficient to attest the correct structure, which is why we have confirmed with X-ray diffraction technique.

| **2.** | **Heatmaps corresponding to synergistic evaluations** |
| --- | --- |
| **2.1** | Heatmaps for compounds **3al**, **5am**, **3bg** and **5ag** in combination with vancomycin. |


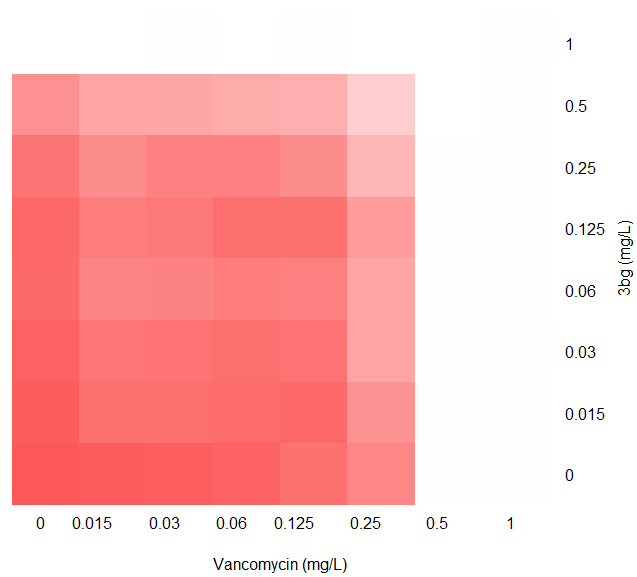

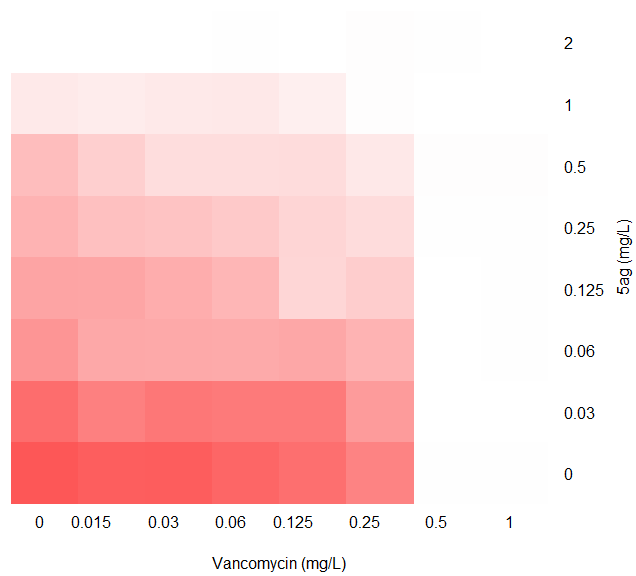

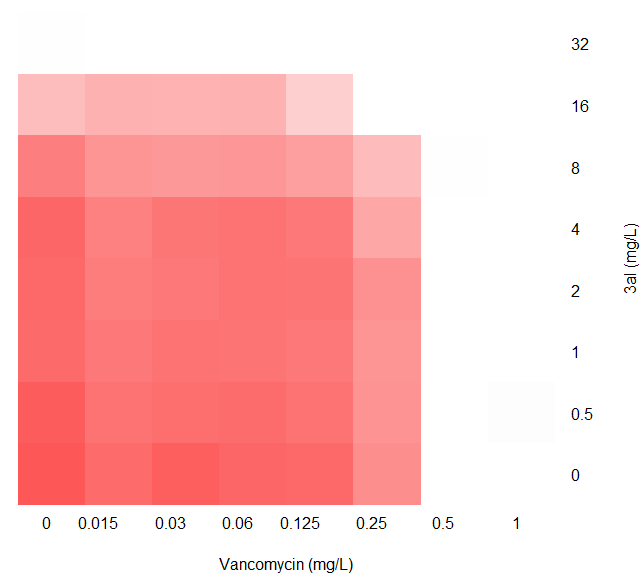


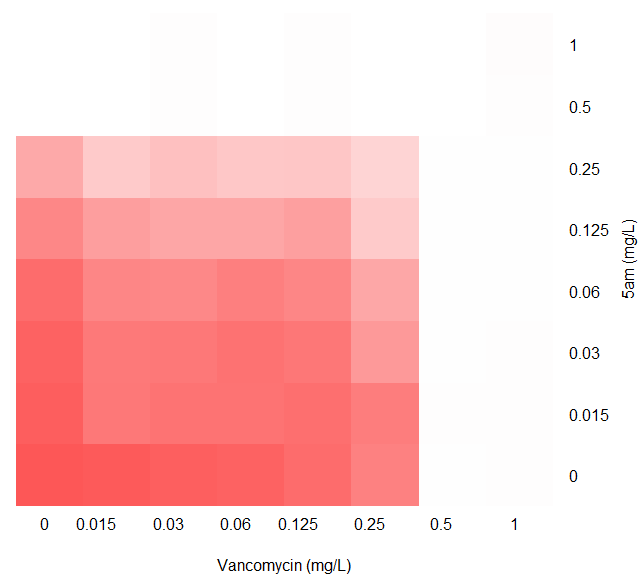


**Figure S1:** Heatmaps for compounds **3al**, **5am**, **3bg** and **5ag** in combination with vancomycin.

| **2.2** | Heatmaps for compounds **3al**, **5am**, **3bg** and **5ag** in combination with cloxacillin. |
| --- | --- |


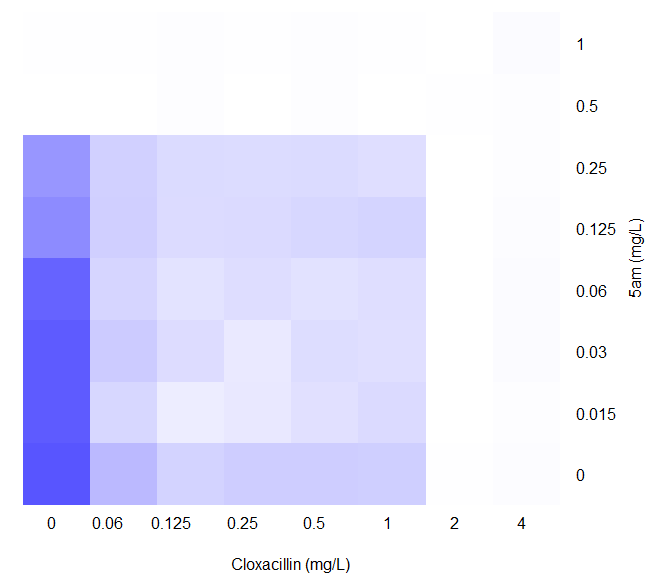

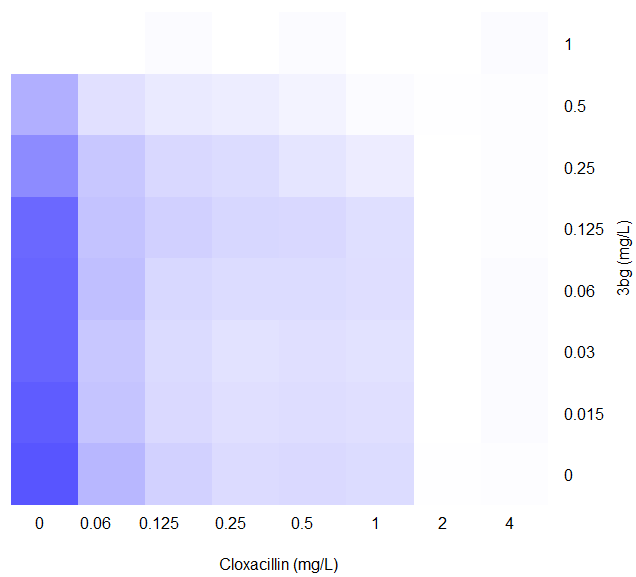

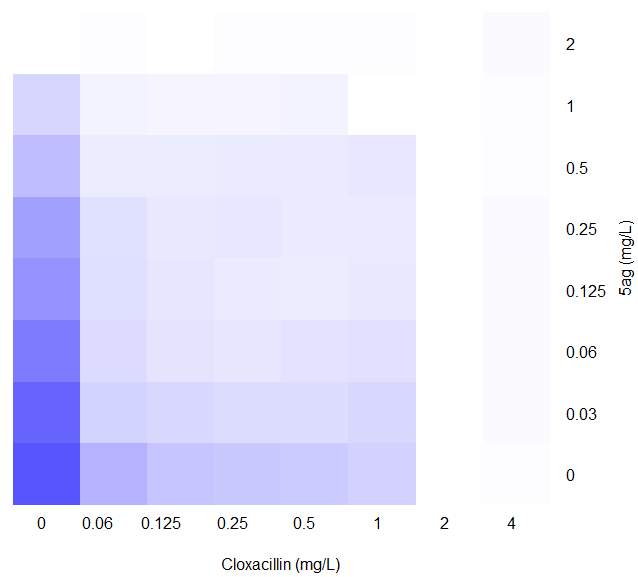

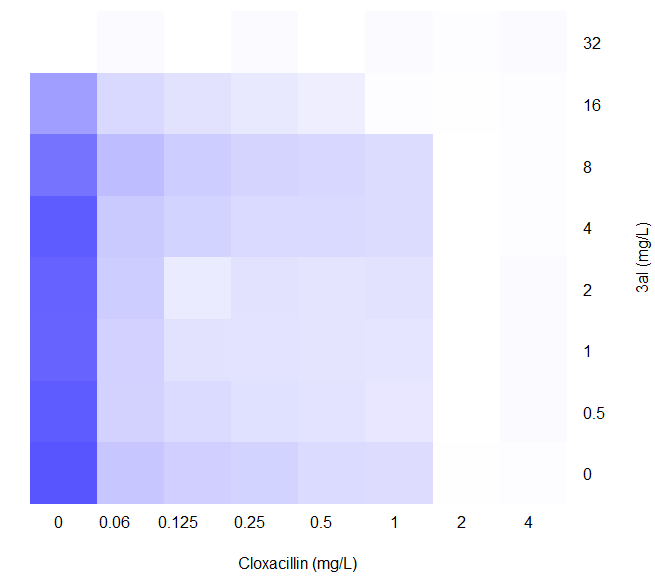


| **3.** | **X-ray crystallographic supporting data** |
| --- | --- |
| **3.1** | X-ray crystallographic supporting data for **5ae** |

 CCDC 2083159 contains the supplementary crystallographic data for this paper. These data can be obtained free of charge from The Cambridge Crystallographic Data Center via www.ccdc.cam.ac.uk/data_request/cif.

| O3-C5  O3-H3  O1-C1  O2-C4  C7-H7  C7-C8  C7-C6  C8-H8  C8-C9  C9-C1  C9-C10  C1-C2  C2-H2  C2-C3  C3-C11  C3-C4  C11-H11A  C11-H11B  C11-C12  C12-H12A  C12-H12B  C12-C13 | \| 1.359(3) \| \| --- \| \| 0.87(2) \| \| 1.226(3) \| \| 1.243(3) \| \| 0.95 \| \| 1.389(3) \| \| 1.380(3) \| \| 0.95 \| \| 1.379(3) \| \| 1.482(3) \| \| 1.411(3) \| \| 1.468(3) \| \| 0.95 \| \| 1.343(3) \| \| 1.499(3) \| \| 1.483(3) \| \| 0.99 \| \| 0.99 \| \| 1.523(3) \| \| 0.99 \| \| 0.99 \| \| 1.516(3) \| | C13-H13A  C13-H13B  C13-C14  C14-H14A  C14-H14B  C14-C15  C15-H15A  C15-H15B  C15-C16  C16-H16A  C16-H16B  C16-C17  C17-H17A  C17-H17B  C17-C18  C18-H18A  C18-H18B  C18-H18C  C6-H6  C6-C5  C5-C10  C10-C4 | \| 0.99 \| \| --- \| \| 0.99 \| \| 1.523(3) \| \| 0.99 \| \| 0.99 \| \| 1.518(3) \| \| 0.99 \| \| 0.99 \| \| 1.519(3) \| \| 0.99 \| \| 0.99 \| \| 1.517(3) \| \| 0.99 \| \| 0.99 \| \| 1.518(3) \| \| 0.98 \| \| 0.98 \| \| 0.98 \| \| 0.95 \| \| 1.389(3) \| \| 1.404(3) \| \| 1.460(3) \| |
| --- | --- | --- | --- | --- | --- | --- | --- | --- | --- | --- | --- | --- | --- | --- | --- | --- | --- | --- | --- | --- | --- | --- | --- | --- | --- | --- | --- | --- | --- | --- | --- | --- | --- | --- | --- | --- | --- | --- | --- | --- | --- | --- | --- | --- | --- | --- | --- |

**Table S1.** Bond lenghts (Å).

| C5-O3-H3 | 109(1) | H14A-C14-H14B | 107.5 |
| --- | --- | --- | --- |
| H7-C7-C8 | 119.8 | H14A-C14-C15 | 108.6 |
| H7-C7-C6 | 119.8 | H14B-C14-C15 | 108.6 |
| C8-C7-C6 | 120.4(2) | C14-C15-H15A | 108.9 |
| C7-C8-H8 | 119.8 | C14-C15-H15B | 108.9 |
| C7-C8-C9 | 120.4(2) | C14-C15-C16 | 113.4(2) |
| H8-C8-C9 | 119.8 | H15A-C15-H15B | 107.7 |
| C8-C9-C1 | 120.9(2) | H15A-C15-C16 | 108.9 |
| C8-C9-C10 | 120.2(2) | H15B-C15-C16 | 108.9 |
| C1-C9-C1 | 119.0(2) | C15-C16-H16A | 108.8 |
| O1-C1-C9 | 121.3(2) | C15-C16-H16B | 108.8 |
| O1-C1-C2 | 120.4(2) | C15-C16-C17 | 114.1(2) |
| C9-C1-C2 | 118.3(2) | H16A-C16-H16B | 107.6 |
| C1-C2-H2 | 118.3 | H16A-C16-C17 | 108.7 |
| C1-C2-C3 | 123.4(2) | H16B-C16-C17 | 108.7 |
| H2-C2-C3 | 118.3 | C16-C17-H17A | 108.9 |
| C2-C3-C11 | 124.3(2) | C16-C17-H17B | 108.9 |
| C2-C3-C4 | 119.0(2) | C16-C17-C18 | 113.5(2) |
| C11-C3-C4 | 116.6(2) | H17A-C17-H17B | 107.7 |
| C3-C11-H11A | 108.6 | H17A-C17-C18 | 108.9 |
| C3-C11-H11B | 108.6 | H17B-C17-C18 | 108.9 |
| C3-C11-C12 | 114.7(2) | C17-C18-H18A | 109.5 |
| H11A-C11-H11B | 107.5 | C17-C18-H18B | 109.5 |
| H11A-C11-C12 | 108.6 | C17-C18-H18C | 109.5 |
| H11B-C11-C12 | 108.6 | H18A-C18-H18B | 109.5 |
| C11-C12-H12A | 108.8 | H18A-C18-H18C | 109.5 |
| C11-C12-H12B | 108.8 | H18B-C18-H18C | 109.5 |
| C11-C12-C13 | 113.9(2) | C7-C6-H6 | 120 |
| H12A-C12-H12B | 107.7 | C7-C6-C5 | 119.9(2) |
| H12A-C12-C13 | 108.8 | H6-C6-C5 | 120 |
| H12B-C12-C13 | 108.7 | O3-C5-C6 | 118.0(2) |
| C12-C13-H13A | 109.2 | O3-C5-C10 | 121.5(2) |
| C12-C13-H13B | 109.2 | C6-C5-C10 | 120.5(2) |
| C12-C13-C14 | 112.1(2) | C9-C10-C5 | 118.6(2) |
| H13A-C13-H13B | 107.9 | C9-C10-C4 | 120.6(2) |
| H13A-C13-C14 | 109.2 | C5-C10-C4 | 120.8(2) |
| H13B-C13-C14 | 109.2 | O2-C4-C3 | 118.8(2) |
| C13-C14-H14A | 108.6 | O2-C4-C10 | 121.4(2) |
| C13-C14-H14B | 108.6 | C3-C4-C10 | 119.7(2) |
| C13-C14-C15 | 114.8(2) |  |  |

**Table S2.** Bond angles (°).

| \| H3-O3-C5-C6 \| \| --- \| \| H3-O3-C5-C10 \| \| H7-C7-C8-H8 \| \| H7-C7-C8-C9 \| \| C6-C7-C8-H8 \| \| C6-C7-C8-C9 \| \| H7-C7-C6-H6 \| \| H7-C7-C6-C5 \| \| C8-C7-C6-H6 \| \| C8-C7-C6-C5 \| \| C7-C8-C9-C1 \| \| C7-C8-C9-C10 \| \| H8-C8-C9-C1 \| \| H8-C8-C9-C10 \| \| C8-C9-C1-O1 \| \| C8-C9-C1-C2 \| \| C10-C9-C1-O1 \| \| C10-C9-C1-C2 \| \| C8-C9-C10-C5 \| \| C8-C9-C10-C4 \| \| C1-C9-C10-C5 \| \| C1-C9-C10-C4 \| \| O1-C1-C2-H2 \| \| O1-C1-C2-C3 \| \| C9-C1-C2-H2 \| \| C9-C1-C2-C3 \| \| C1-C2-C3-C11 \| \| C1-C2-C3-C4 \| \| H2-C2-C3-C11 \| \| H2-C2-C3-C4 \| \| C2-C3-C11-H11A \| \| C2-C3-C11-H11B \| \| C2-C3-C11-C12 \| \| C4-C3-C11-H11A \| \| C4-C3-C11-H11B \| \| C4-C3-C11-C12 \| \| C2-C3-C4-O2 \| \| C2-C3-C4-C10 \| \| C11-C3-C4-O2 \| \| C11-C3-C4-C10 \| \| C3-C11-C12-H12 \| \| C3-C11-C12-H12 \| \| C3-C11-C12-C13 \| \| H11A-C11-C12-H12A \| \| H11A-C11-C12-H12B \| \| H11A-C11-C12-C13 \| \| H11B-C11-C12-H12A \| \| H11B-C11-C12-H12B \| \| H11B-C11-C12-C13 \| \| C11-C12-C13-H13A \| \| C11-C12-C13-H13B \| \| C11-C12-C13-C14 \| \| H12A-C12-C13-H13A \| \| H12A-C12-C13-H13B \| \| H12A-C12-C13-C14 \| \| H12B-C12-C13-H13A \| \| H12B-C12-C13-H13B \| \| H12B-C12-C13-C14 \| | \| 179(2) \| \| --- \| \| 0(2) \| \| -0.6 \| \| 179.4 \| \| 179.4 \| \| -0.6(3) \| \| 0.1 \| \| -179.9 \| \| -179.9 \| \| 0.2(3) \| \| 179.7(2) \| \| 0.3(3) \| \| -0.3 \| \| -179.7 \| \| -0.8(3) \| \| -179.8(2) \| \| 178.6(2) \| \| -0.4(3) \| \| 0.5(3) \| \| 179.9(2) \| \| -178.9(2) \| \| 0.5(3) \| \| 1.3 \| \| -178.7(2) \| \| -179.7 \| \| 0.3(3) \| \| 179.5(2) \| \| -0.3(3) \| \| -0.5 \| \| 179.7 \| \| -125.6 \| \| 117.7 \| \| -4.0(3) \| \| 54.1 \| \| -62.5 \| \| 175.8(2) \| \| 179.7(2) \| \| 0.4(3) \| \| -0.1(3) \| \| -179.4(2) \| \| -58.3 \| \| 58.7 \| \| -179.8(2) \| \| 63.4 \| \| -179.6 \| \| -58.1 \| \| -180 \| \| -62.9 \| \| 58.5 \| \| 58.7 \| \| -59 \| \| 179.8(2) \| \| -62.8 \| \| 179.5 \| \| 58.3 \| \| -179.8 \| \| 62.5 \| \| -58.7 \| | \| C12-C13-C14-H14A \| \| --- \| \| C12-C13-C14-H14B \| \| C12-C13-C14-C15 \| \| H13A-C13-C14-H14A \| \| H13A-C13-C14-H14B \| \| H13A-C13-C14-C15 \| \| H13B-C13-C14-H14A \| \| H13B-C13-C14-H14B \| \| H13B-C13-C14-C15 \| \| C13-C14-C15-H14A \| \| C13-C14-C15-H14B \| \| C13-C14-C15-C16 \| \| H14A-C14-C15-H15A \| \| H14A-C14-C15-H15B \| \| H14A-C14-C15-C16 \| \| H14B-C14-C15-H15A \| \| H14B-C14-C15-H15B \| \| H14B-C14-C15-C16 \| \| C14-C15-C16-H16A \| \| C14-C15-C16-H16B \| \| C14-C15-C16-C17 \| \| H15A-C15-C16-H16A \| \| H15A-C15-C16-H16B \| \| H15A-C15-C16-C17 \| \| H15B-C15-C16-H16A \| \| H15B-C15-C16-H16B \| \| H15B-C15-C16-C17 \| \| C15-C16-C17-H17A \| \| C15-C16-C17-H17B \| \| C15-C16-C17-C18 \| \| H16A-C16-C17-H17A \| \| H16A-C16-C17-H17B \| \| H16A-C16-C17-C18 \| \| H16B-C16-C17-H17A \| \| H16B-C16-C17-H17B \| \| H16B-C16-C17-C18 \| \| C16-C17-C18-H18A \| \| C16-C17-C18-H18B \| \| C16-C17-C18-H18C \| \| H17A-C17-C18-H18A \| \| H17A-C17-C18-H18B \| \| H17A-C17-C18-H18C \| \| H17B-C17-C18-H18A \| \| H17B-C17-C18-H18B \| \| H17B-C17-C18-H18C \| \| C7-C6-C5-O3 \| \| C7-C6-C5-C10 \| \| H6-C6-C5-O3 \| \| H6-C6-C5-C10 \| \| O3-C5-C10-C9 \| \| O3-C5-C10-C4 \| \| C6-C5-C10-C9 \| \| C6-C5-C10-C4 \| \| C9-C10-C4-O2 \| \| C9-C10-C4-C3 \| \| C5-C10-C4-O2 \| \| C5-C10-C4-C3 \| | \| -55.6 \| \| --- \| \| 61 \| \| -177.3(2) \| \| 65.5 \| \| -177.9 \| \| -56.2 \| \| -176.8 \| \| -60.1 \| \| 61.5 \| \| -59.5 \| \| 57.7 \| \| 179.1(2) \| \| 178.8 \| \| -64 \| \| 57.4 \| \| 62.1 \| \| 179.4 \| \| -59.3 \| \| 57.2 \| \| -59.7 \| \| 178.8(2) \| \| -64.2 \| \| 178.9 \| \| 57.4 \| \| 178.6 \| \| 61.7 \| \| -59.9 \| \| -58.3 \| \| 58.8 \| \| -179.7(2) \| \| 63.2 \| \| -179.7 \| \| -58.2 \| \| -179.9 \| \| -62.7 \| \| 58.7 \| \| -62.8 \| \| 177.2 \| \| 57.2 \| \| 175.8 \| \| 55.8 \| \| -64.2 \| \| 58.6 \| \| -61.4 \| \| 178.6 \| \| -178.4(2) \| \| 0.7(3) \| \| 1.6 \| \| -179.3 \| \| 178.1(2) \| \| -1.3(3) \| \| -1.0(3) \| \| 179.6(2) \| \| -179.8(2) \| \| -0.5(3) \| \| -0.4(3) \| \| 178.9(2) \| |
| --- | --- | --- | --- | --- | --- | --- | --- | --- | --- | --- | --- | --- | --- | --- | --- | --- | --- | --- | --- | --- | --- | --- | --- | --- | --- | --- | --- | --- | --- | --- | --- | --- | --- | --- | --- | --- | --- | --- | --- | --- | --- | --- | --- | --- | --- | --- | --- | --- | --- | --- | --- | --- | --- | --- | --- | --- | --- | --- | --- | --- | --- | --- | --- | --- | --- | --- | --- | --- | --- | --- | --- | --- | --- | --- | --- | --- | --- | --- | --- | --- | --- | --- | --- | --- | --- | --- | --- | --- | --- | --- | --- | --- | --- | --- | --- | --- | --- | --- | --- | --- | --- | --- | --- | --- | --- | --- | --- | --- | --- | --- | --- | --- | --- | --- | --- | --- | --- | --- | --- | --- | --- | --- | --- | --- | --- | --- | --- | --- | --- | --- | --- | --- | --- | --- | --- | --- | --- | --- | --- | --- | --- | --- | --- | --- | --- | --- | --- | --- | --- | --- | --- | --- | --- | --- | --- | --- | --- | --- | --- | --- | --- | --- | --- | --- | --- | --- | --- | --- | --- | --- | --- | --- | --- | --- | --- | --- | --- | --- | --- | --- | --- | --- | --- | --- | --- | --- | --- | --- | --- | --- | --- | --- | --- | --- | --- | --- | --- | --- | --- | --- | --- | --- | --- | --- | --- | --- | --- | --- | --- | --- | --- | --- | --- | --- | --- | --- | --- | --- | --- | --- | --- | --- | --- | --- | --- | --- | --- | --- | --- | --- | --- | --- | --- |

**Table S3.** Torsion angles (°)

|  | | | | |
| --- | --- | --- | --- | --- |
|  | **Donor-H** | **Acceptor-H** | **Donor-Acceptor** | **Angle** |
| O3-H3...O2 | 0.873 | 1.801 | 2.575 | 147 |

**Table S4.** Hydrogen bond distances (Å) and angles (°).

| **3.** | **X-ray crystallographic supporting data** |
| --- | --- |
| **3.2** | X-ray crystallographic supporting data for **3bg** |

CCDC 2083158 contains the supplementary crystallographic data for this paper. These data can be obtained free of charge from The Cambridge Crystallographic Data Center via www.ccdc.cam.ac.uk/data_request/cif.

| O2-C4  O4-C8  O4-H4  O1-C1  O3-C5  O3-H3  C9-C1  C9-C10  C9-C8  C3-H3A  C3-C4  C3-C2  C4-C10  C1-C2  C2-C11  C10-C5  C8-C7  C7-H7  C7-C6  C5-C6  C11-H11 | \| 1.244(2) \| \| --- \| \| 1.347(2) \| \| 0.90(2) \| \| 1.242(2) \| \| 1.343(2) \| \| 0.88(2) \| \| 1.459(2) \| \| 1.419(2) \| \| 1.396(2) \| \| 0.95 \| \| 1.461(2) \| \| 1.341(2) \| \| 1.456(2) \| \| 1.486(2) \| \| 1.514(2) \| \| 1.394(2) \| \| 1.407(2) \| \| 0.95 \| \| 1.364(2) \| \| 1.407(2) \| \| 1 \| | C11-C12  C11-C15  C6-H6  C12-H12A  C12-H12B  C12-C13  C15-H15A  C15-H15B  C15-C16  C16-H16A  C16-H16B  C16-C17  C13-H13A  C13-H13B  C13-C14  C14-H14A  C14-H14B  C14-H14C  C17-H17A  C17-H17B  C17-H17C | \| 1.531(2) \| \| --- \| \| 1.538(2) \| \| 0.95 \| \| 0.99 \| \| 0.99 \| \| 1.514(2) \| \| 0.99 \| \| 0.99 \| \| 1.523(2) \| \| 0.99 \| \| 0.99 \| \| 1.505(3) \| \| 0.99 \| \| 0.99 \| \| 1.515(2) \| \| 0.98 \| \| 0.98 \| \| 0.98 \| \| 0.98 \| \| 0.98 \| \| 0.98 \| |
| --- | --- | --- | --- | --- | --- | --- | --- | --- | --- | --- | --- | --- | --- | --- | --- | --- | --- | --- | --- | --- | --- | --- | --- | --- | --- | --- | --- | --- | --- | --- | --- | --- | --- | --- | --- | --- | --- | --- | --- | --- | --- | --- | --- | --- | --- |

**Table S5.** Bond lengths (Å).

| C8-O4-H4  C5-O3-H3  C1-C9-C10  C1-C9-C8  C10-C9-C8  H3A-C3-C4  H3A-C3-C2  C4-C3-C2  O2-C4-C3  O2-C4-C10  C3-C4-C10  O1-C1-C9  O1-C1-C2  C9-C1-C2  C3-C2-C1  C3-C2-C11  C1-C2-C11  C9-C10-C4  C9-C10-C5  C4-C10-C5  O4-C8-C9  O4-C8-C7  C9-C8-C7  C8-C7-H7  C8-C7-C6  H7-C7-C6  O3-C5-C10  O3-C5-C6  C10-C5-C6  C2-C11-H11  C2-C11-C12  C2-C11-C15  H11-C11-C12  H11-C11-C15  C12-C11-C15  C7-C6-C5  C7-C6-H6 | \| 104(1) \| \| --- \| \| 106(1) \| \| 120.3(1) \| \| 120.3(1) \| \| 119.4(1) \| \| 118.2 \| \| 118.2 \| \| 123.5(1) \| \| 119.9(1) \| \| 121.8(1) \| \| 118.3(1) \| \| 121.0(1) \| \| 120.0(1) \| \| 119.1(1) \| \| 119.1(1) \| \| 122.2(1) \| \| 118.7(1) \| \| 119.5(1) \| \| 119.9(1) \| \| 120.5(1) \| \| 122.4(1) \| \| 117.8(1) \| \| 119.8(1) \| \| 119.7 \| \| 120.6(1) \| \| 119.7 \| \| 122.4(1) \| \| 118.1(1) \| \| 119.5(1) \| \| 108 \| \| 110.2(1) \| \| 111.6(1) \| \| 108 \| \| 108 \| \| 110.9(1) \| \| 120.7(1) \| \| 119.6 \| | C5-C6-H6  C11-C12-H12A  C11-C12-H12B  C11-C12-C13  H12A-C12-H12B  H12A-C12-C13  H12B-C12-C13  C11-C15-H15A  C11-C15-H15B  C11-C15-C16  H15A-C15-H15B  H15A-C15-C16  H15B-C15-C16  C15-C16-H16A  C15-C16-H16B  C15-C16-C17  H16A-C16-H16B  H16A-C16-C17  H16B-C16-C17  C12-C13-H13A  C12-C13-H13B  C12-C13-C14  H13A-C13-H13B  H13A-C13-C14  H13B-C13-C14  C13-C14-H14A  C13-C14-H14B  C13-C14-H14C  H14A-C14-H14B  H14A-C14-H14C  H14B-C14-H14C  C16-C17-H17A  C16-C17-H17B  C16-C17-H17C  H17A-C17-H17B  H17A-C17-H17C  H17B-C17-H17C | \| 119.6 \| \| --- \| \| 108.5 \| \| 108.5 \| \| 115.2(1) \| \| 107.5 \| \| 108.5 \| \| 108.5 \| \| 108.5 \| \| 108.5 \| \| 115.0(1) \| \| 107.5 \| \| 108.5 \| \| 108.5 \| \| 108.7 \| \| 108.7 \| \| 114.2(1) \| \| 107.6 \| \| 108.7 \| \| 108.7 \| \| 109 \| \| 109 \| \| 112.8(1) \| \| 107.8 \| \| 109 \| \| 109 \| \| 109.5 \| \| 109.5 \| \| 109.5 \| \| 109.5 \| \| 109.5 \| \| 109.5 \| \| 109.5 \| \| 109.5 \| \| 109.4 \| \| 109.5 \| \| 109.5 \| \| 109.5 \| |
| --- | --- | --- | --- | --- | --- | --- | --- | --- | --- | --- | --- | --- | --- | --- | --- | --- | --- | --- | --- | --- | --- | --- | --- | --- | --- | --- | --- | --- | --- | --- | --- | --- | --- | --- | --- | --- | --- | --- | --- | --- | --- | --- | --- | --- | --- | --- | --- | --- | --- | --- | --- | --- | --- | --- | --- | --- | --- | --- | --- | --- | --- | --- | --- | --- | --- | --- | --- | --- | --- | --- | --- | --- | --- | --- | --- | --- | --- |

**Table S6.** Bond angles (°).

| H4-O4-C8-C9  H4-O4-C8-C7  H3-O3-C5-C10  H3-O3-C5-C6  C10-C9-C1-O1  C10-C9-C1-C2  C8-C9-C1-O1  C8-C9-C1-C2  C1-C9-C10-C4  C1-C9-C10-C5  C8-C9-C10-C4  C8-C9-C10-C5  C1-C9-C8-O4  C1-C9-C8-C7  C10-C9-C8-O4  C10-C9-C8-C7  H3A-C3-C4-O2  H3A-C3-C4-C10  C2-C3-C4-O2  C2-C3-C4-C10  H3A-C3-C2-C1  H3A-C3-C2-C11  C4-C3-C2-C1  C4-C3-C2-C11  O2-C4-C10-C9  O2-C4-C10-C5  C3-C4-C10-C9  C3-C4-C10-C5  O1-C1-C2-C3  O1-C1-C2-C11  C9-C1-C2-C3  C9-C1-C2-C11  C3-C2-C11-H11  C3-C2-C11-C12  C3-C2-C11-C15  C1-C2-C11-H11  C1-C2-C11-C12  C1-C2-C11-C15  C9-C10-C5-O3  C9-C10-C5-C6  C4-C10-C5-O3  C4-C10-C5-C6  O4-C8-C7-H7  O4-C8-C7-C6  C9-C8-C7-H7  C9-C8-C7-C6  C8-C7-C6-C5  C8-C7-C6-H6  H7-C7-C6-C5  H7-C7-C6-H6  O3-C5-C6-C7  O3-C5-C6-H6  C10-C5-C6-C7  C10-C5-C6-H6 | \| -2(2) \| \| --- \| \| 178(2) \| \| 3(1) \| \| -177(1) \| \| -178.2(1) \| \| 1.7(2) \| \| 0.5(2) \| \| -179.5(1) \| \| 1.1(2) \| \| -180.0(1) \| \| -177.7(1) \| \| 1.2(2) \| \| -0.4(2) \| \| 180.0(1) \| \| 178.3(1) \| \| -1.3(2) \| \| 0.5 \| \| -179 \| \| -179.5(1) \| \| 1.0(2) \| \| -178.1 \| \| 3.6 \| \| 1.9(2) \| \| -176.4(1) \| \| 178.0(1) \| \| -0.9(2) \| \| -2.5(2) \| \| 178.6(1) \| \| 176.8(1) \| \| -4.9(2) \| \| -3.2(2) \| \| 175.1(1) \| \| -175.3 \| \| 67.0(2) \| \| -56.7(2) \| \| 6.4 \| \| -111.3(1) \| \| 125.0(1) \| \| 179.7(1) \| \| -0.1(2) \| \| -1.3(2) \| \| 178.9(1) \| \| 0.5 \| \| \| -179.5(1) \| \| \| -179.9 \| \| \| 0.1(2) \| \| \| 1.1(2) \| \| \| -178.9 \| \| \| -178.9 \| \| \| 1.1 \| \| \| 179.1(1) \| \| \| -0.9 \| \| \| -1.1(2) \| \| \| 178.9 \| \| | C2-C11-C12-H12A  C2-C11-C12-H12B  C2-C11-C12-C13  H11-C11-C12-H12A  H11-C11-C12-H12B  H11-C11-C12-C13  C15-C11-C12-H12A  C15-C11-C12-H12B  C15-C11-C12-C13  C2-C11-C15-H15A  C2-C11-C15-H15B  C2-C11-C15-C16  H11-C11-C15-H15A  H11-C11-C15-H15B  H11-C11-C15-C16  C12-C11-C15-H15A  C12-C11-C15-H15B  C12-C11-C15-C16  C11-C12-C13-H13A  C11-C12-C13-H13B  C11-C12-C13-C14  H12A-C12-C13-H13A  H12A-C12-C13-H13B  H12A-C12-C13-C14  H12B-C12-C13-H13A  H12B-C12-C13-H13B  H12B-C12-C13-C14  C11-C15-C16-H16A  C11-C15-C16-H16B  C11-C15-C16-C17  H15A-C15-C16-H16A  H15A-C15-C16-H16B  H15A-C15-C16-C17  H15B-C15-C16-H16A  H15B-C15-C16-H16B  H15B-C15-C16-C17  C15-C16-C17-H17A  C15-C16-C17-H17B  C15-C16-C17-H17C  H16A-C16-C17-H17A  H16A-C16-C17-H17B  H16A-C16-C17-H17C  H16B-C16-C17-H17A  H16B-C16-C17-H17B  H16B-C16-C17-H17C  C12-C13-C14-H14A  C12-C13-C14-H14B  C12-C13-C14-H14C  H13A-C13-C14-H14A  H13A-C13-C14-H14B  H13A-C13-C14-H14C  H13B-C13-C14-H14A  H13B-C13-C14-H14B  H13B-C13-C14-H14C | 177.5  -66  55.8(2)  59.8  176.3  -62  -58.4  58.1  179.8(1)  60.2  176.8  -61.5(2)  178.8  -64.6  57  -63  53.5  175.2(1)  58.6  -58.8  179.9(1)  -63.1  179.4  58.1  -179.6  62.9  -58.4  56.2  173  -65.4(2)  -65.6  51.3  172.8  177.9  -65.3  56.3  -58.8  -178.8  61.2  179.6  59.6  -60.4  62.8  -57.3  -177.2  -60.4  179.6  59.5  60.8  -59.2  -179.2  178.3  58.3  -61.8 |
| --- | --- | --- | --- | --- | --- | --- | --- | --- | --- | --- | --- | --- | --- | --- | --- | --- | --- | --- | --- | --- | --- | --- | --- | --- | --- | --- | --- | --- | --- | --- | --- | --- | --- | --- | --- | --- | --- | --- | --- | --- | --- | --- | --- | --- | --- | --- | --- | --- | --- | --- | --- | --- | --- | --- | --- | --- | --- | --- | --- | --- | --- | --- | --- | --- | --- | --- | --- | --- | --- |

**Table S7.** Torsion angles (°).

|  | | | | |
| --- | --- | --- | --- | --- |
|  | **Donor-H** | **Acceptor-H** | **Donor-Acceptor** | **Angle** |
| O3-H3...O2 | 0.885 | 1.781 | 2.582 | 149 |
| O4-H4...O1 | 0.897 | 1.734 | 2.561 | 152 |

**Table S8.** Hydrogen bond distances (Å) and angles (°)

| **4.** | **Characterization of final compounds (^1^H, ^13^C, DEPT-135, HRMS, HPLC)** |
| --- | --- |


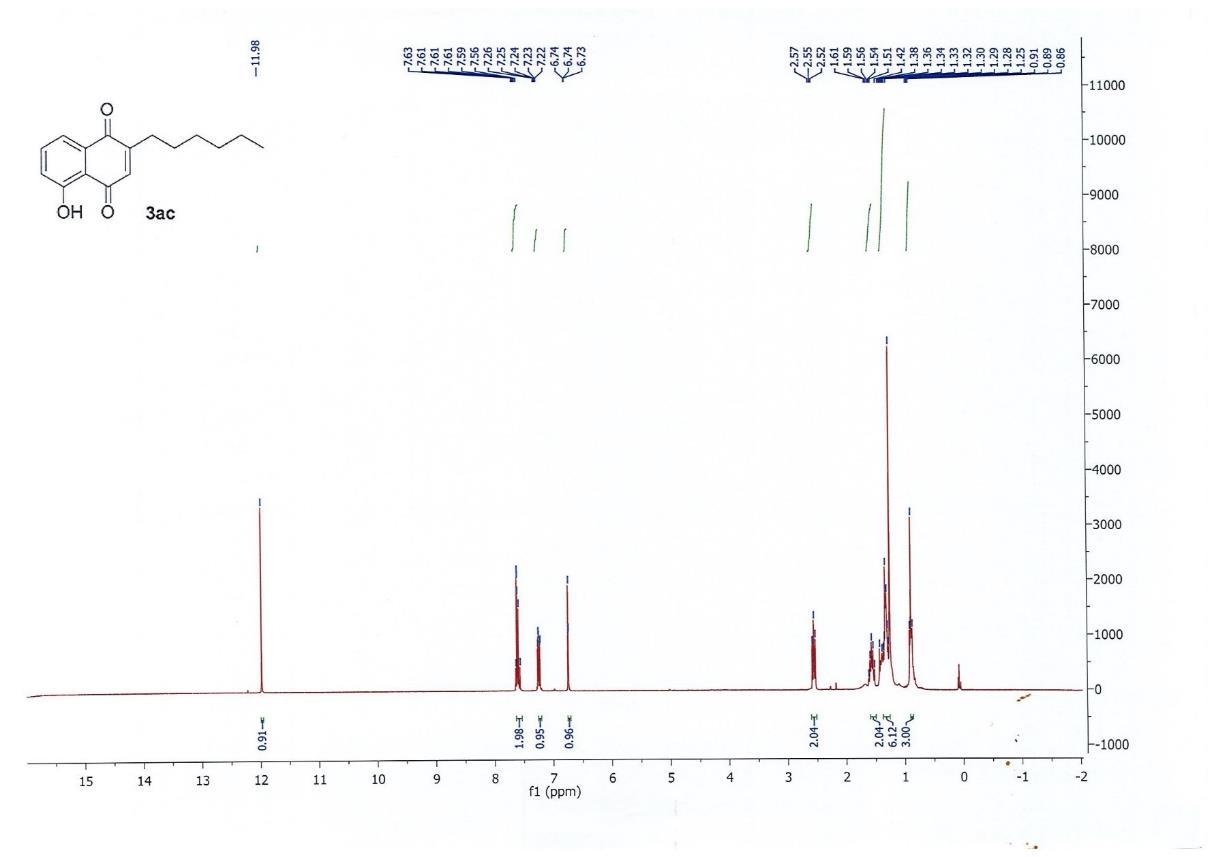


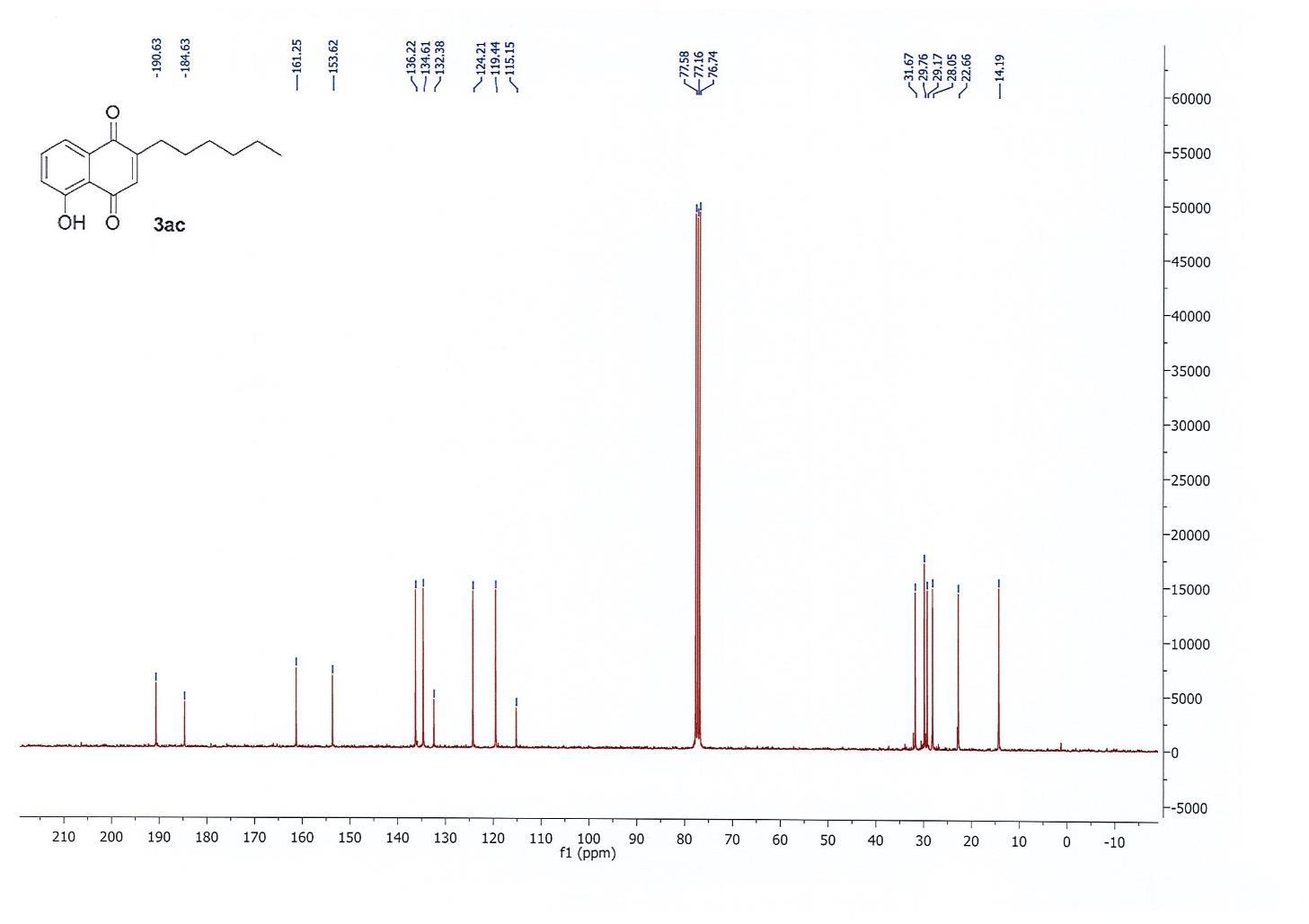


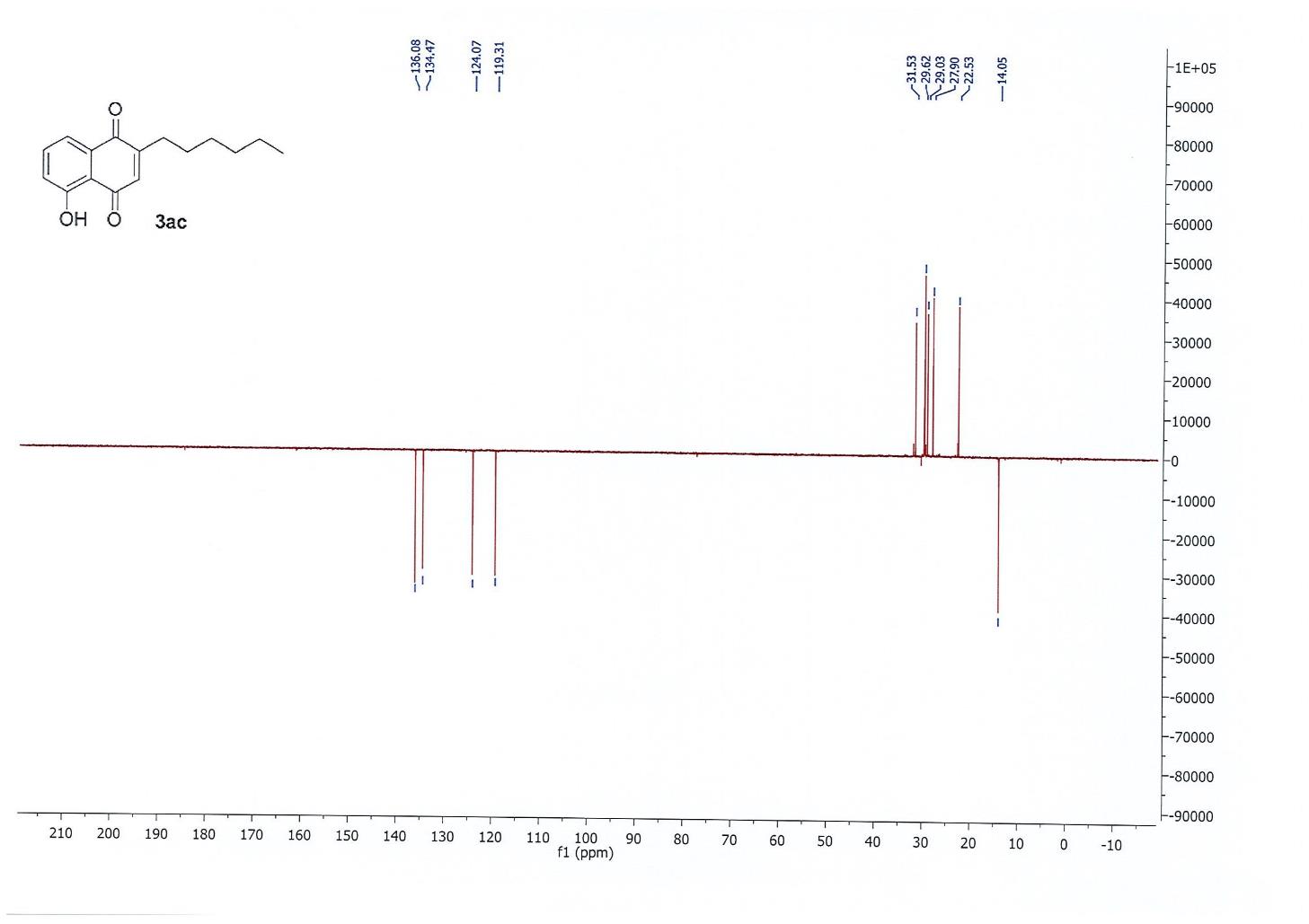


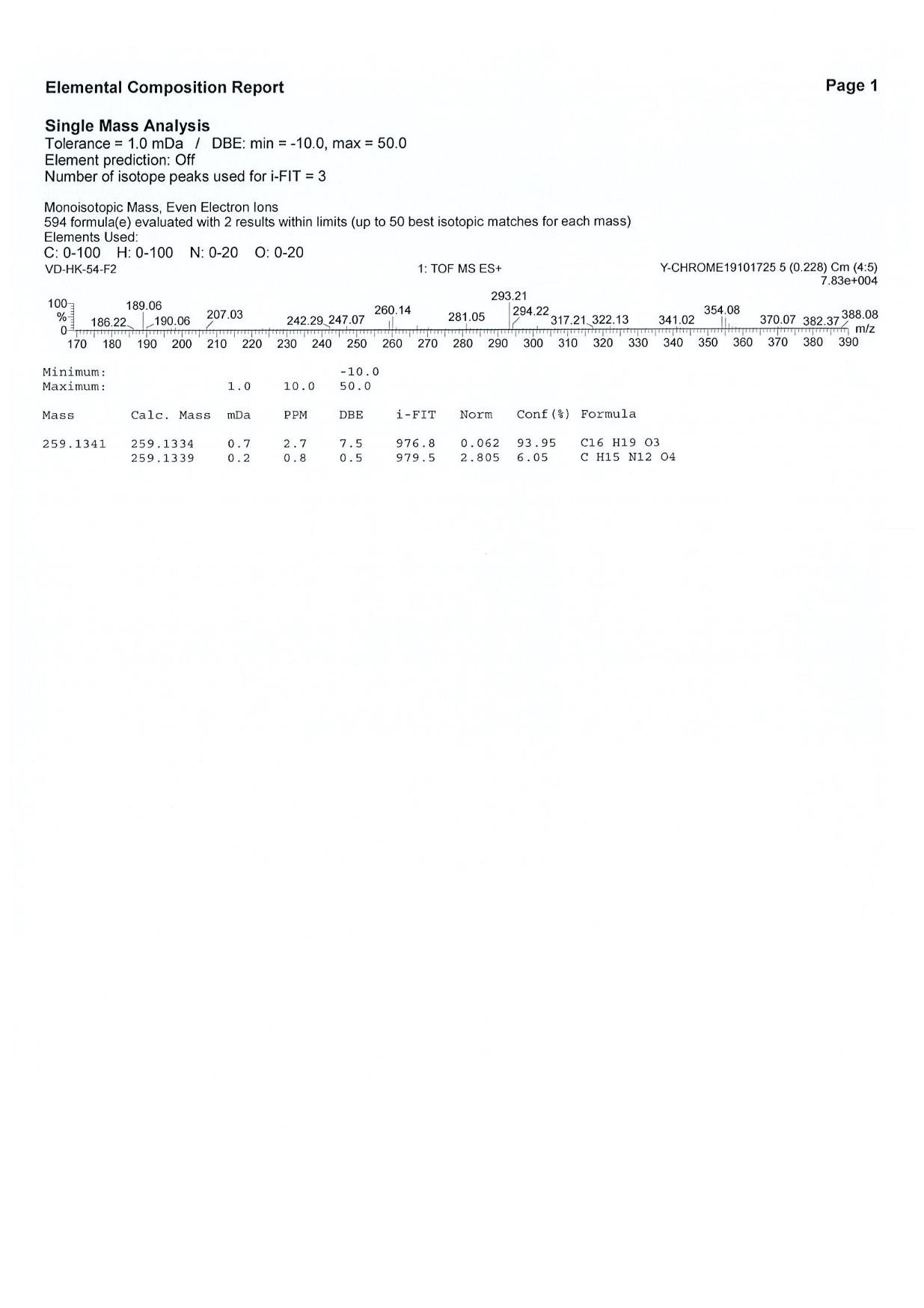


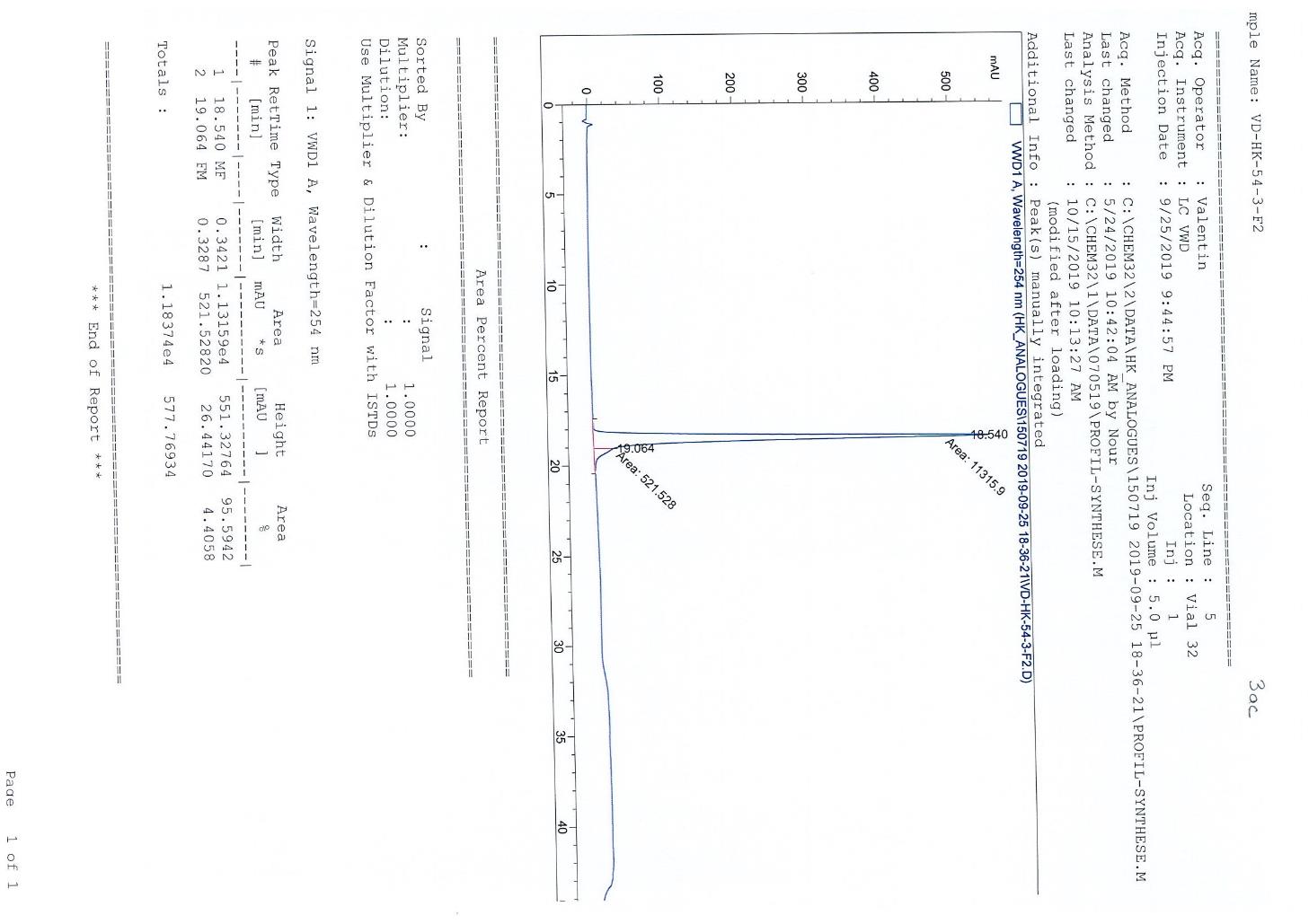


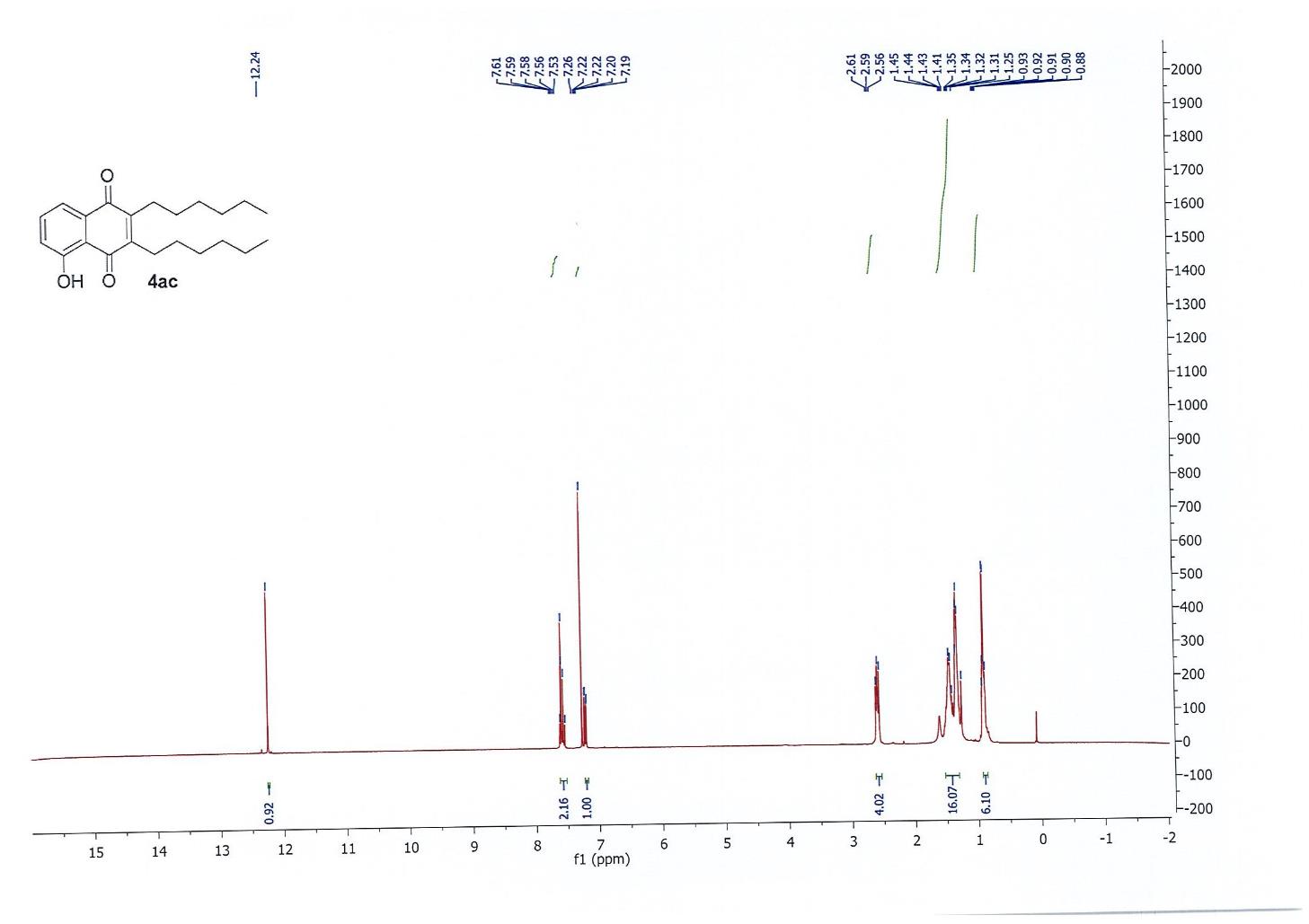


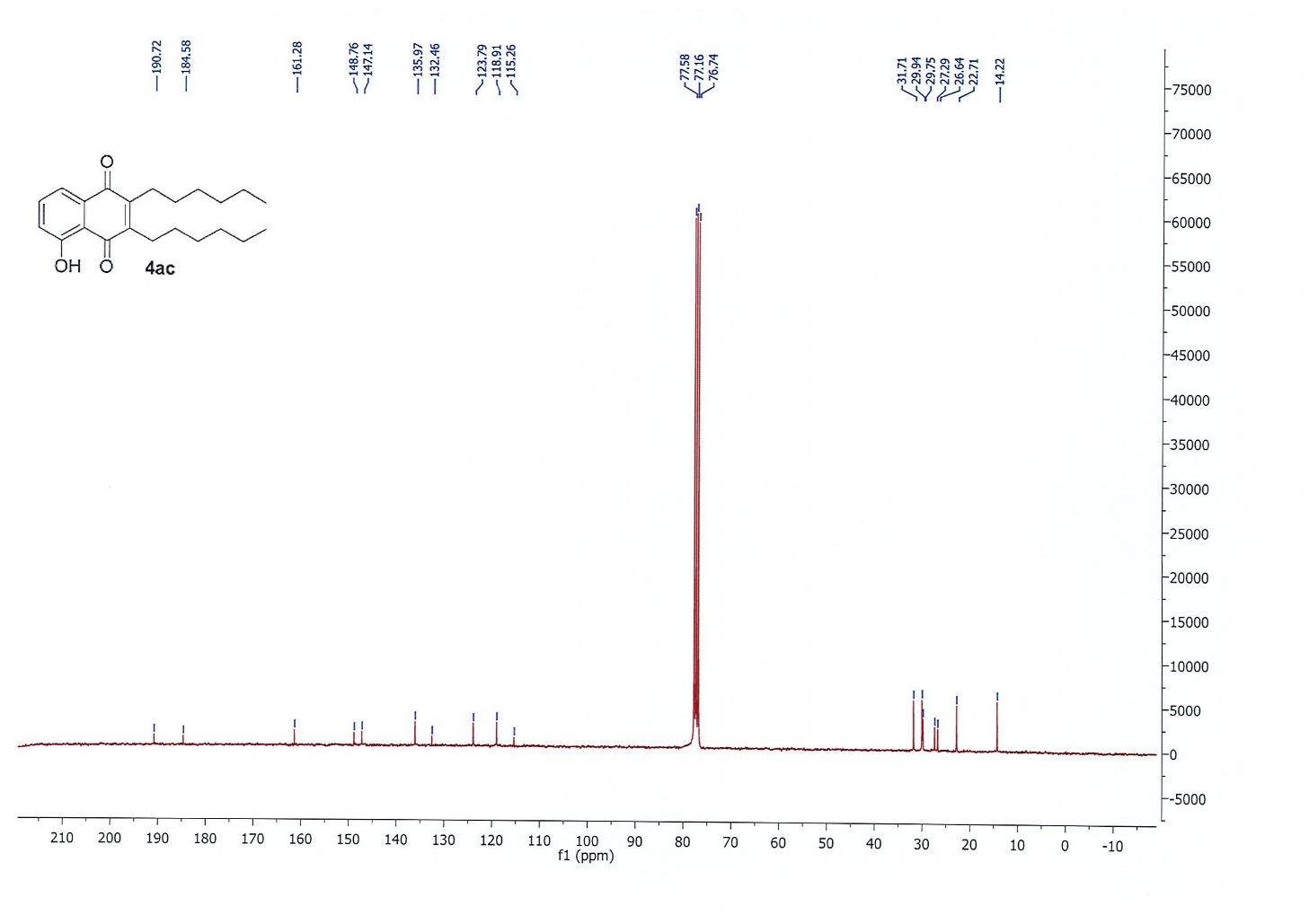


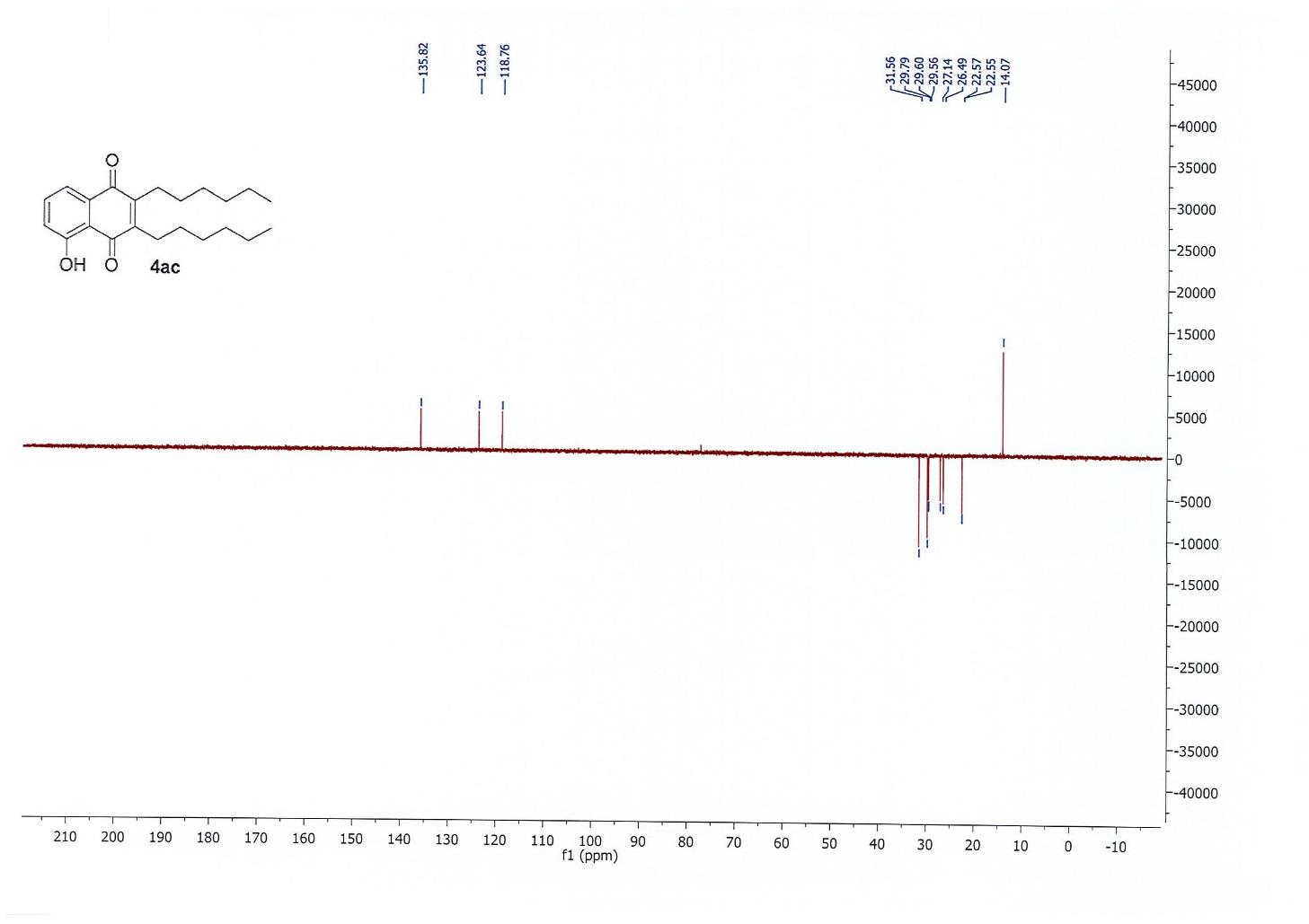


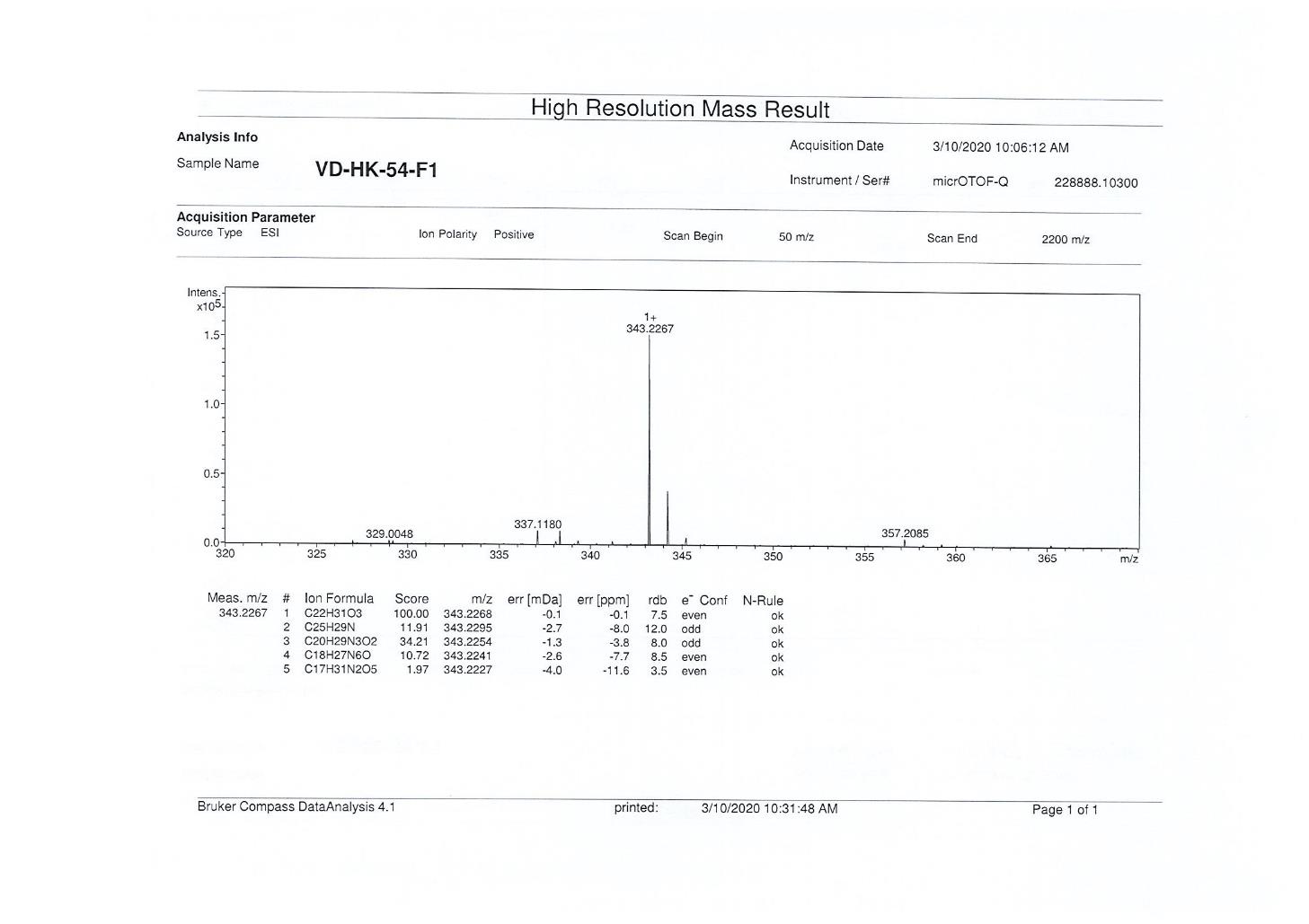


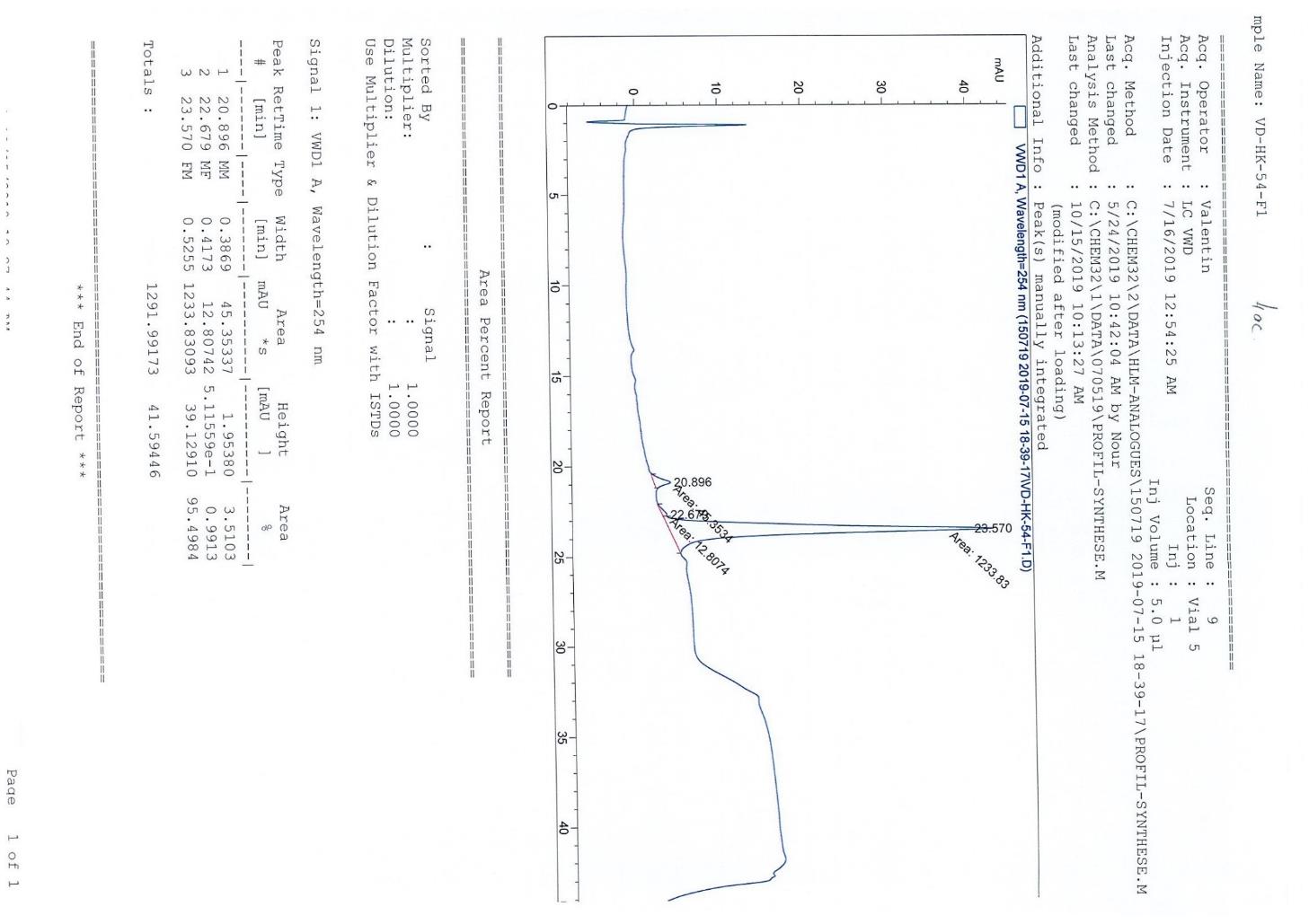


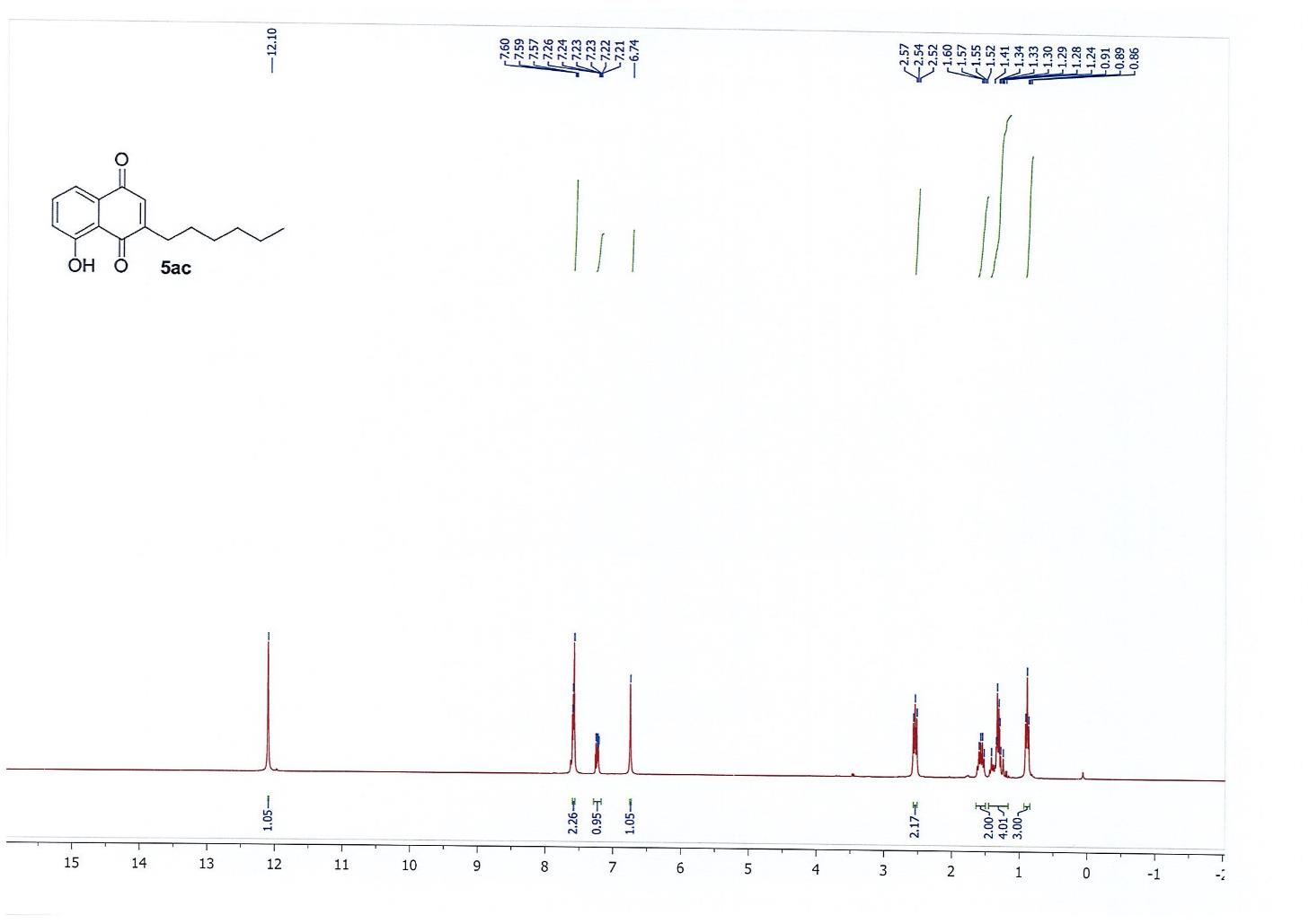


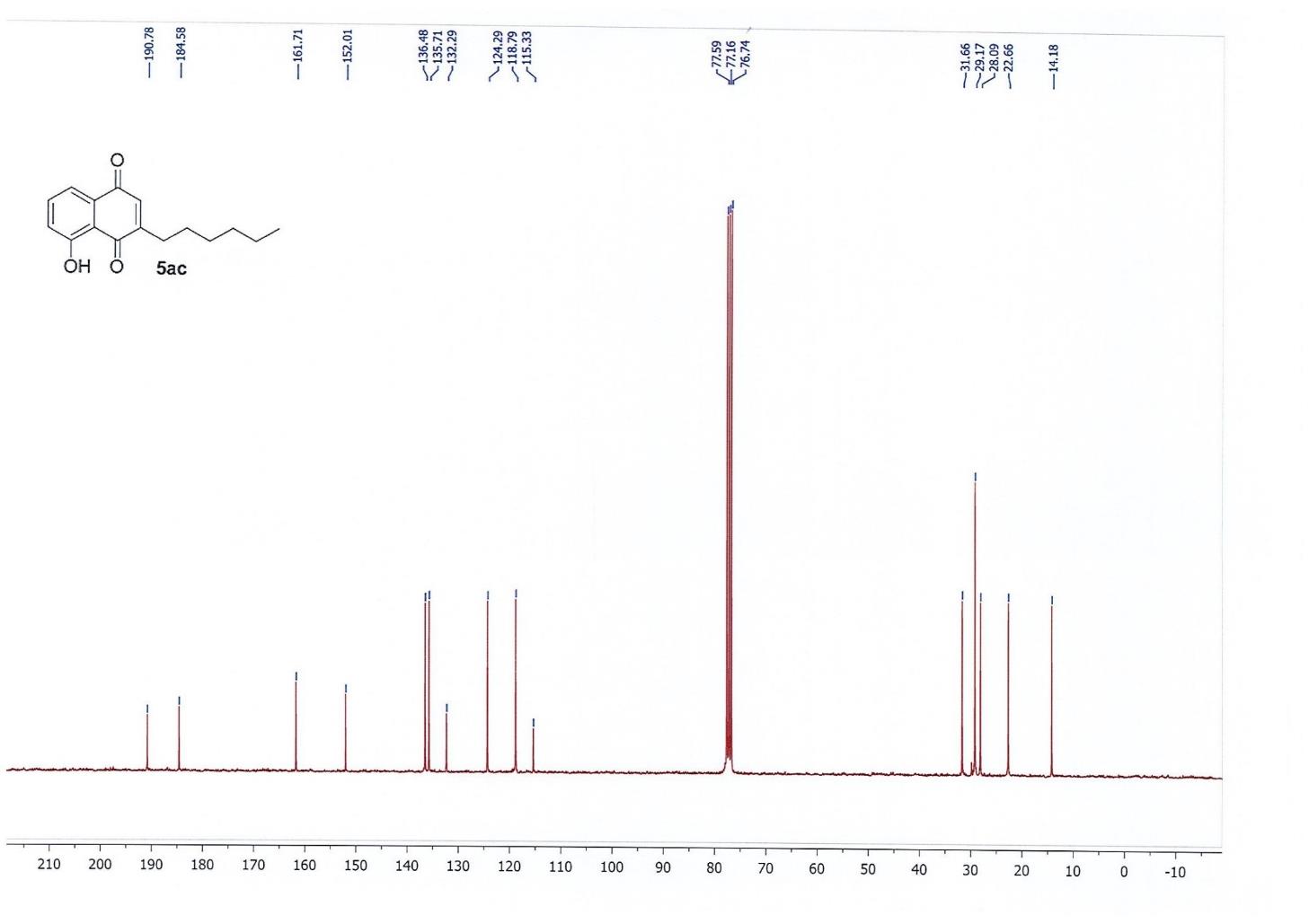


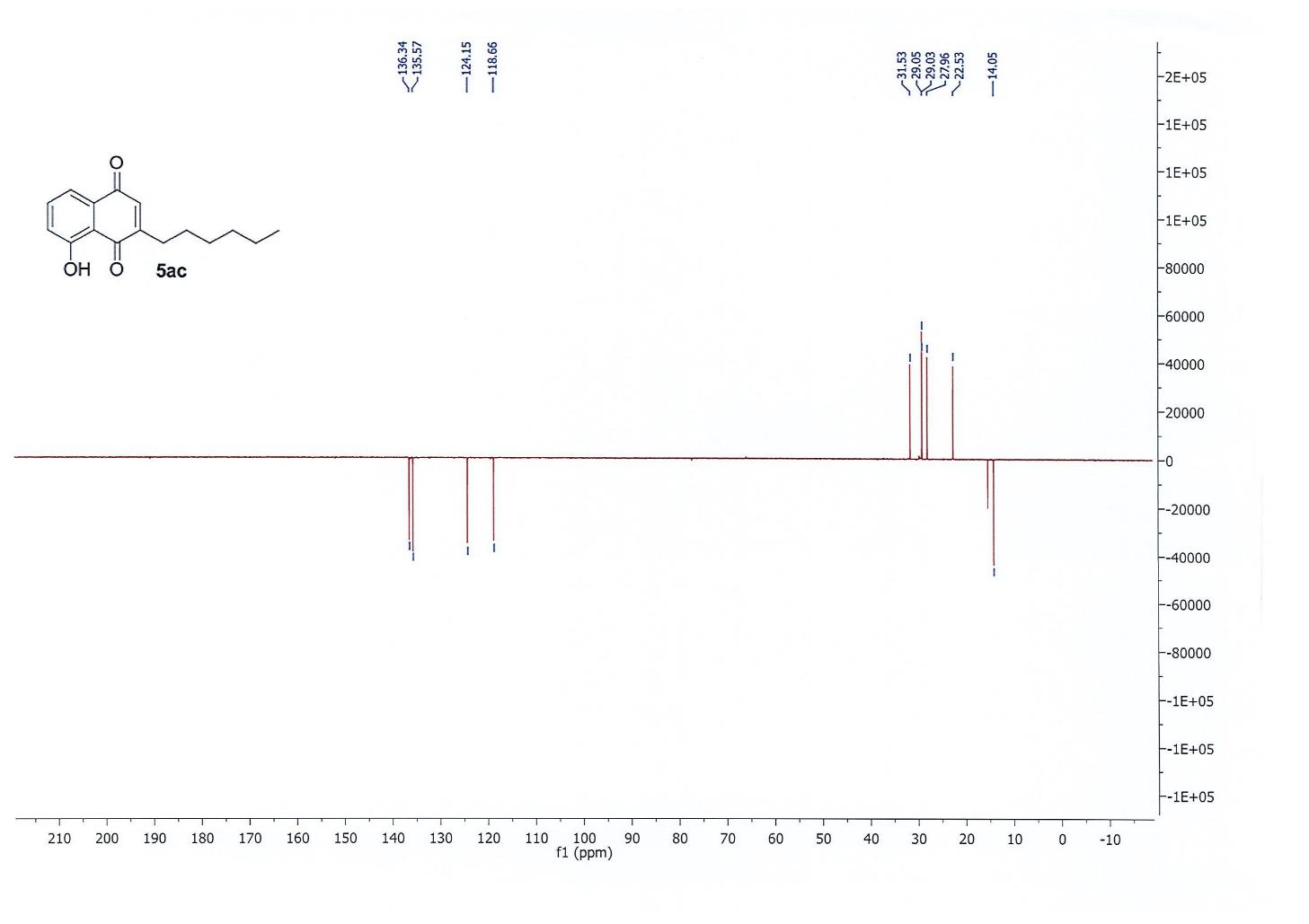


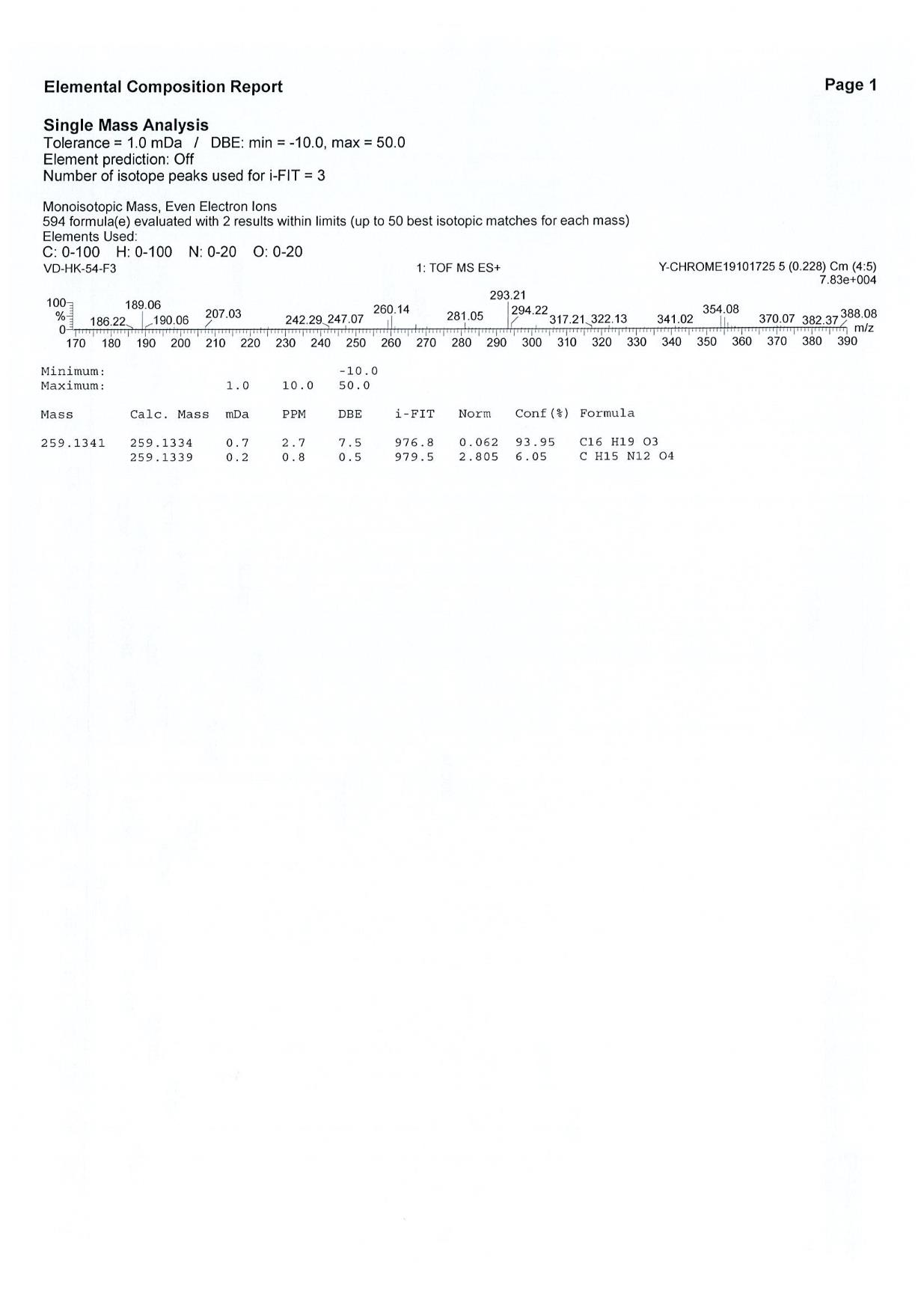


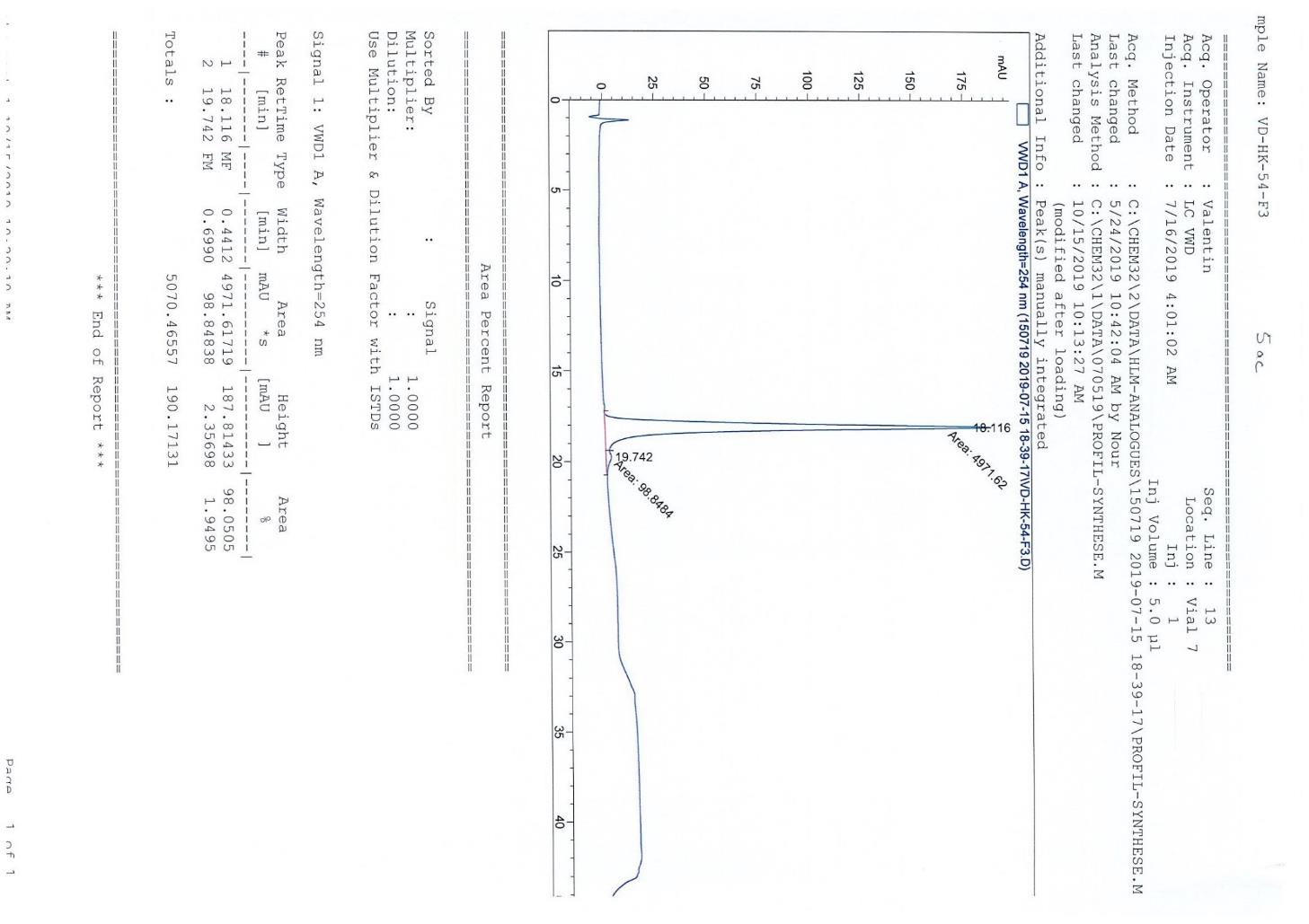


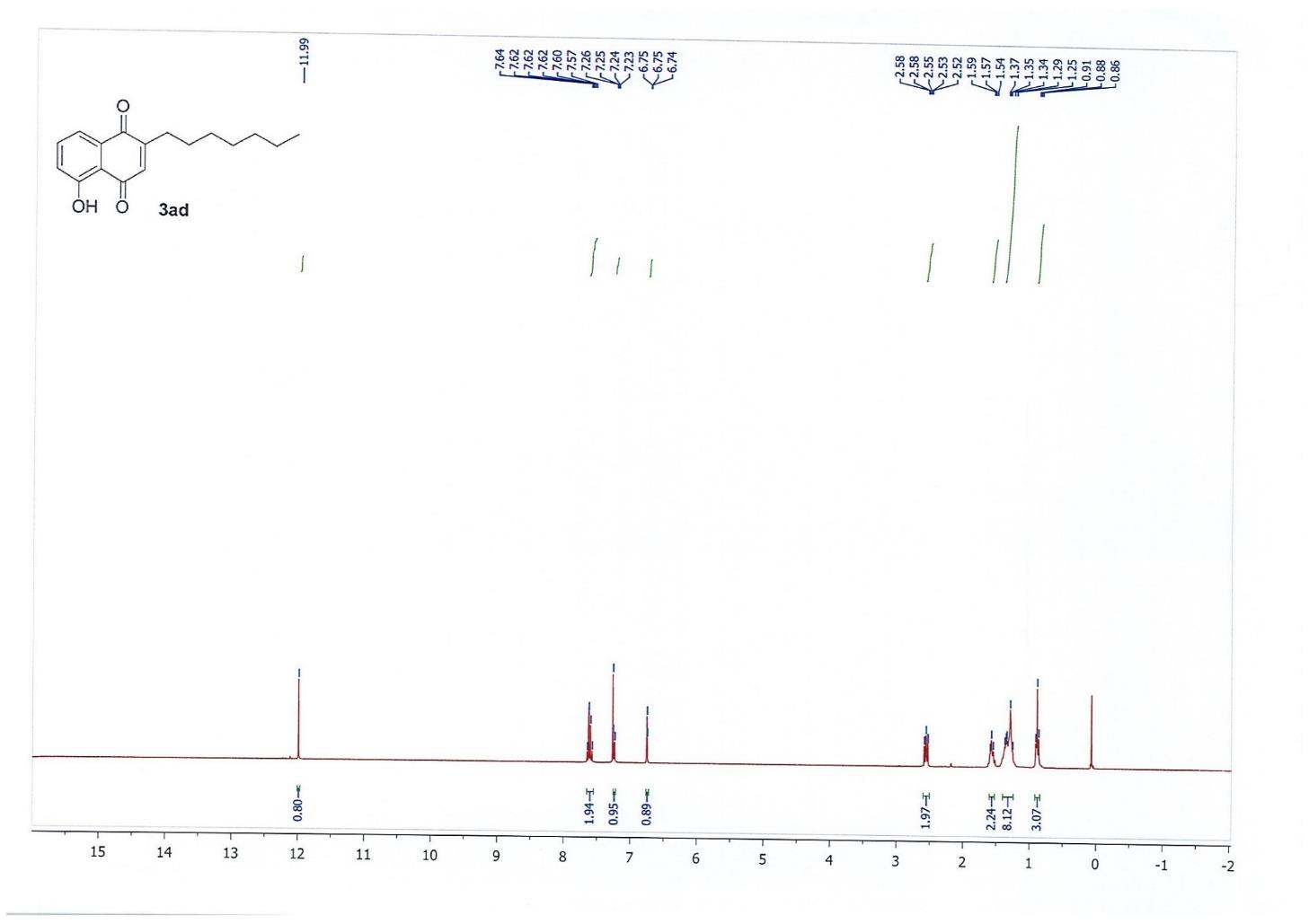


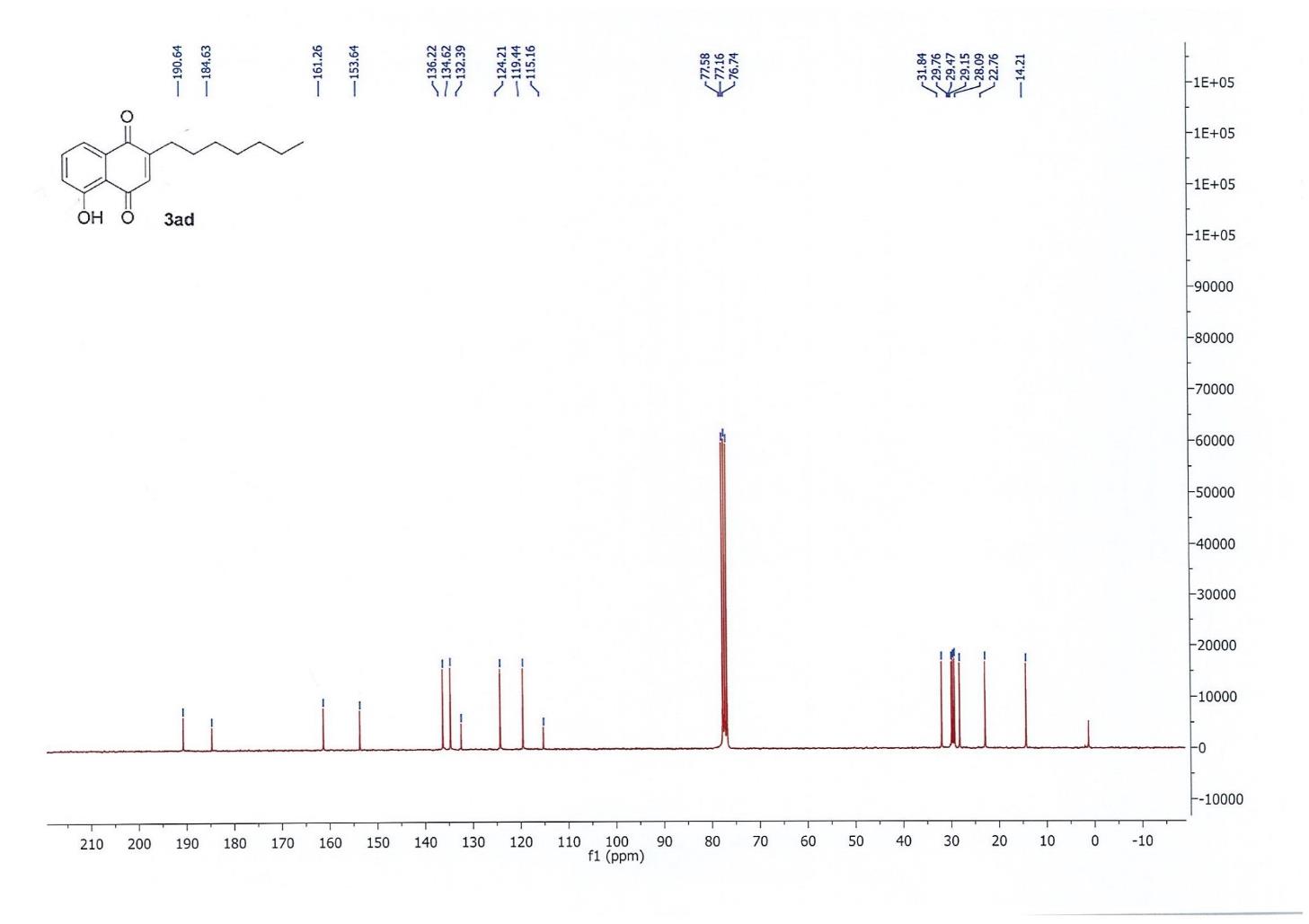


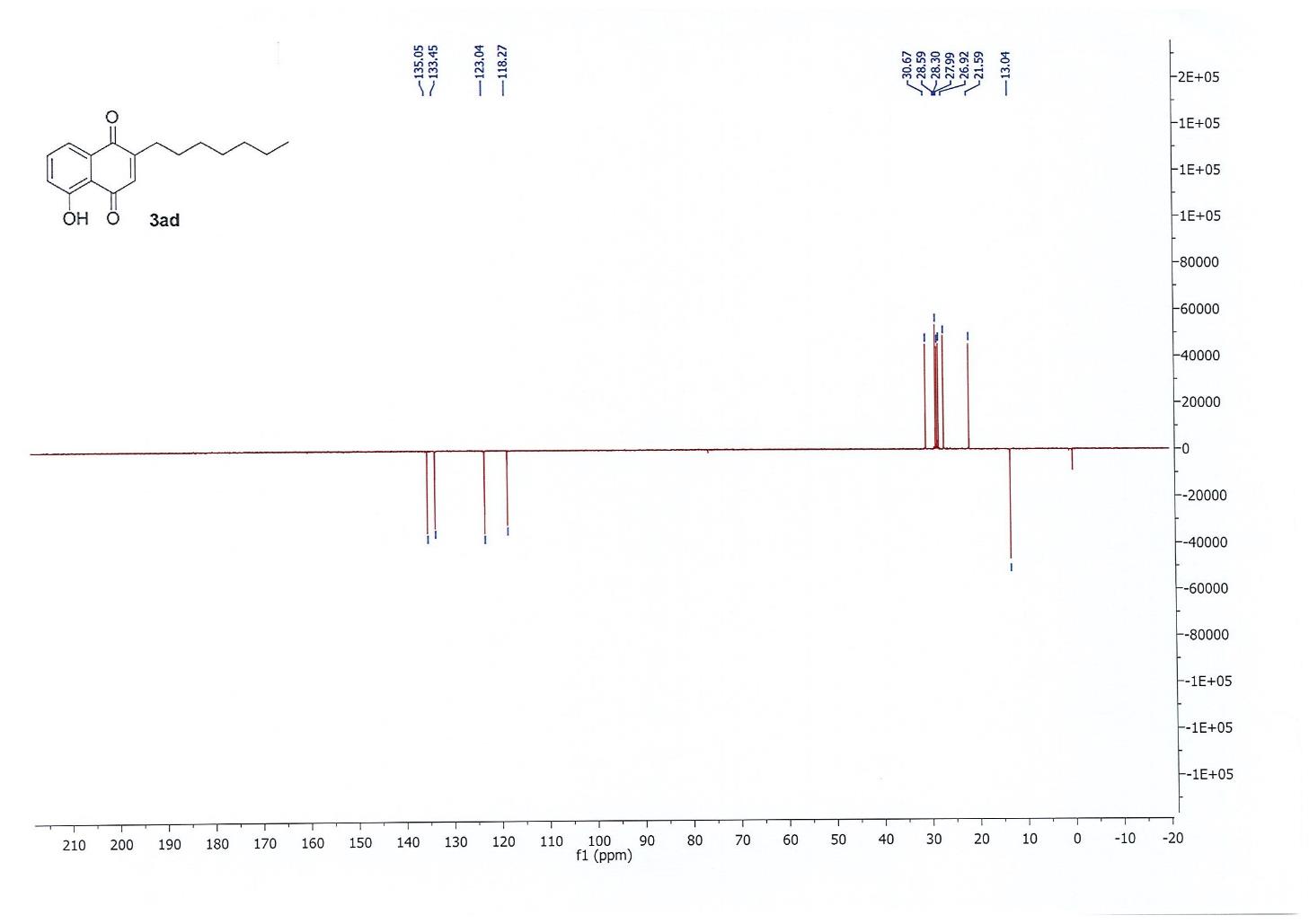


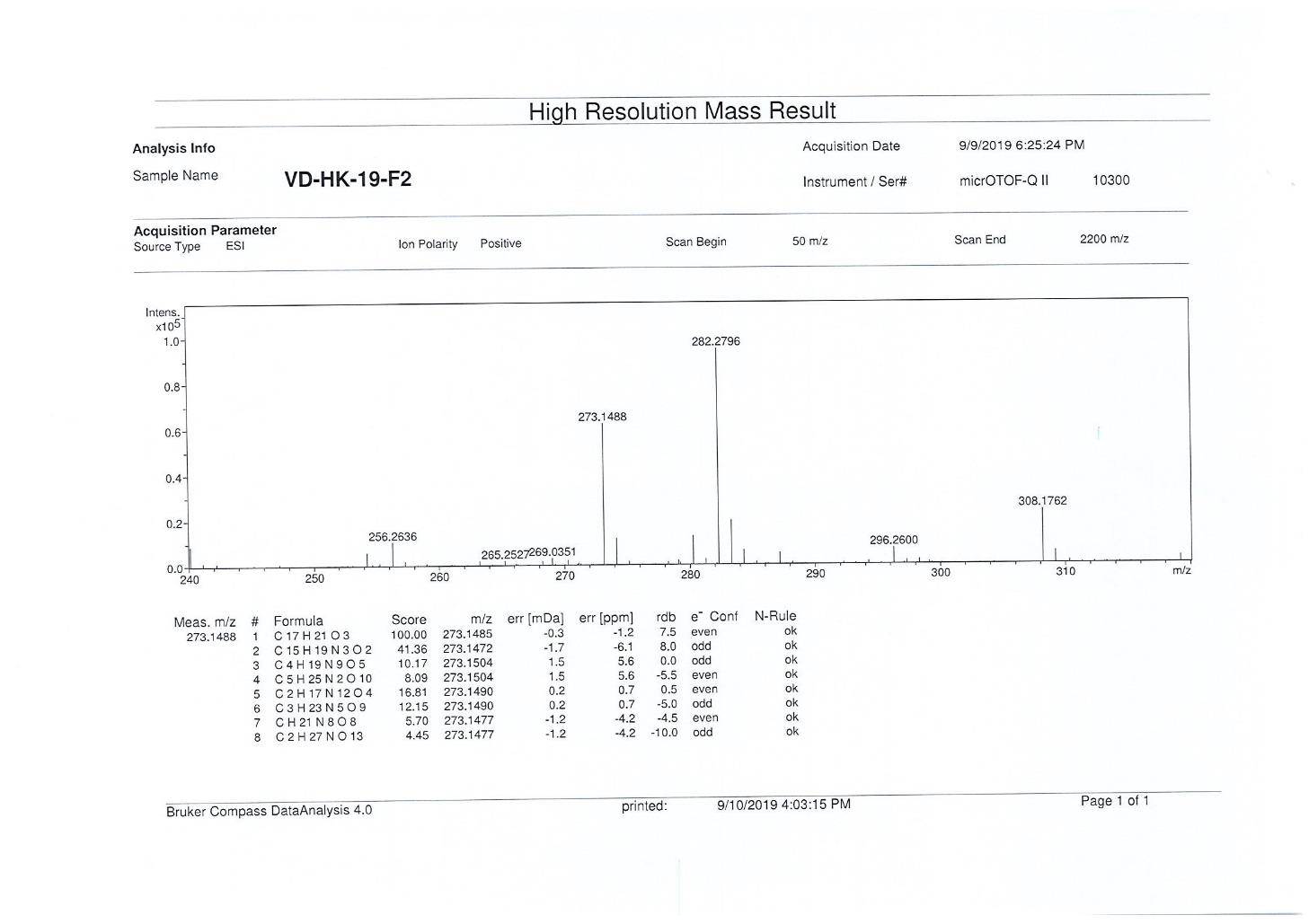


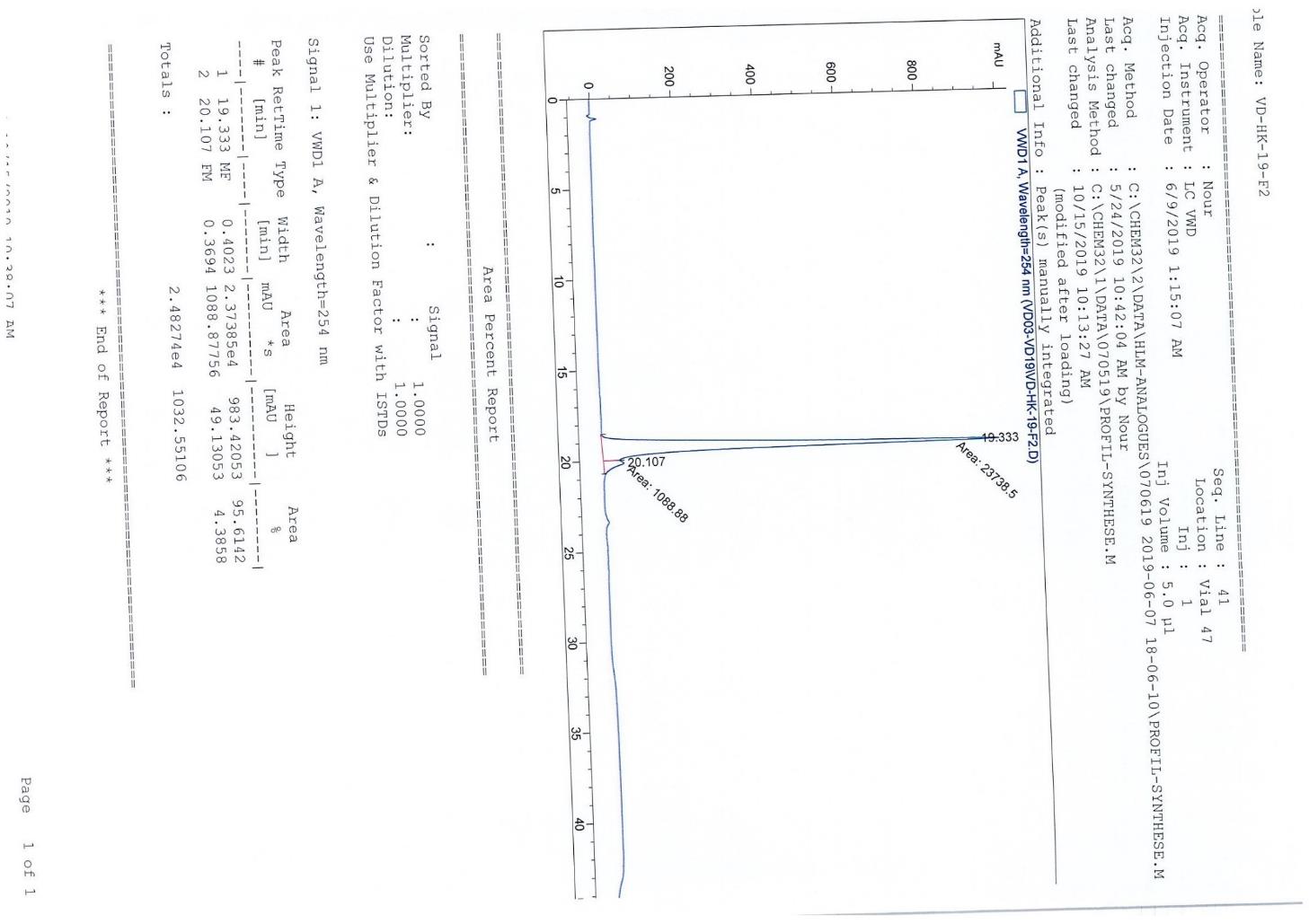


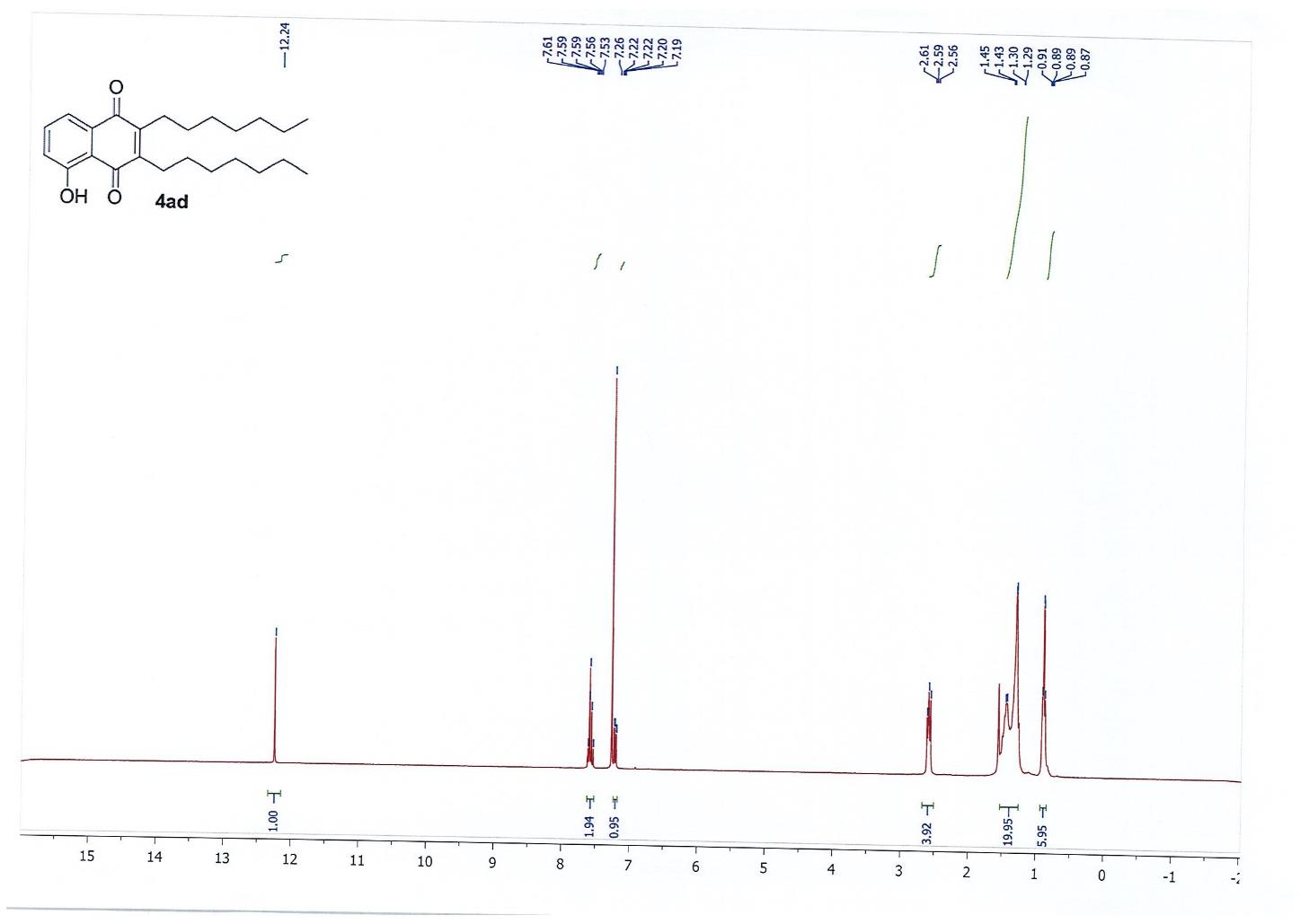


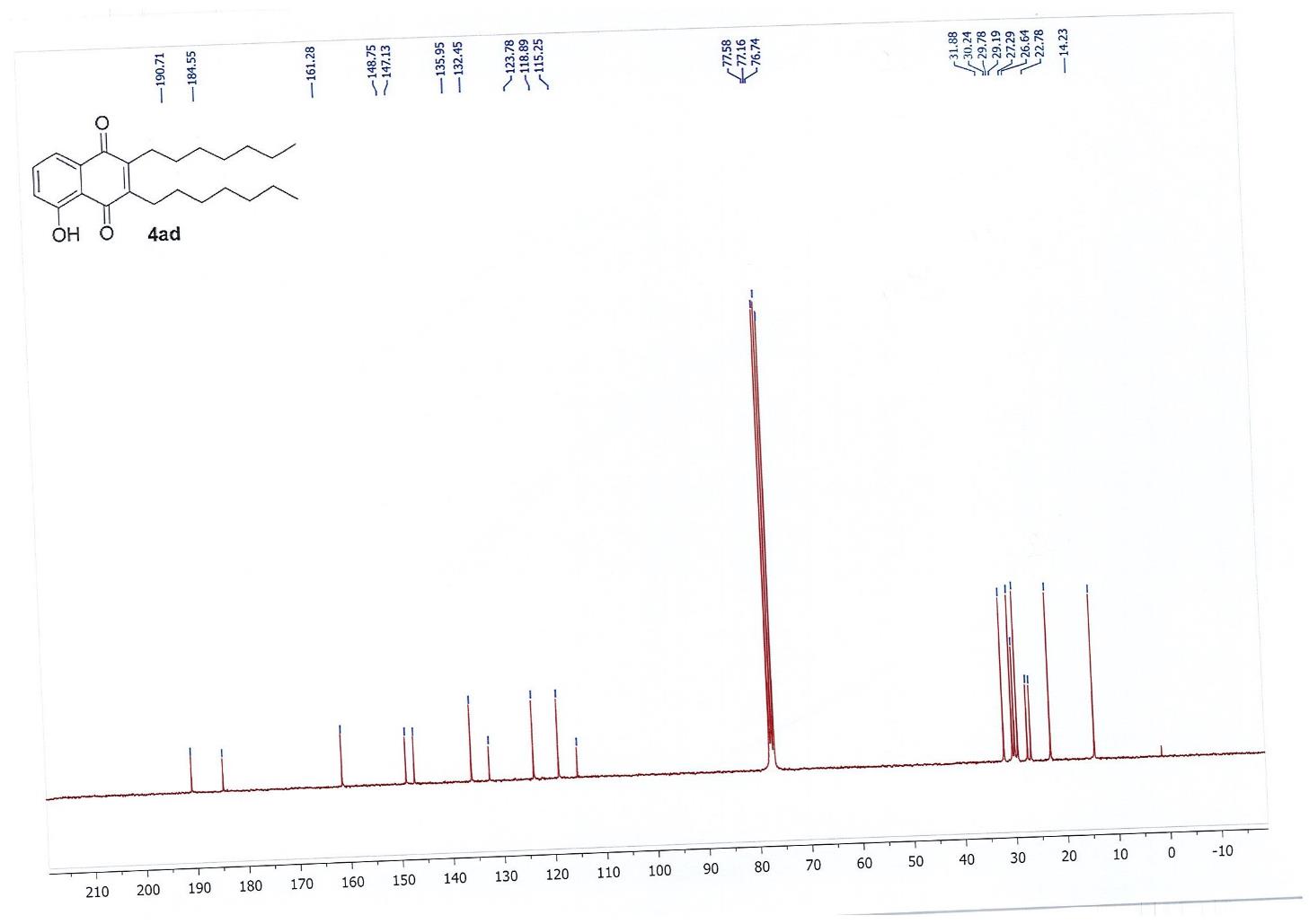


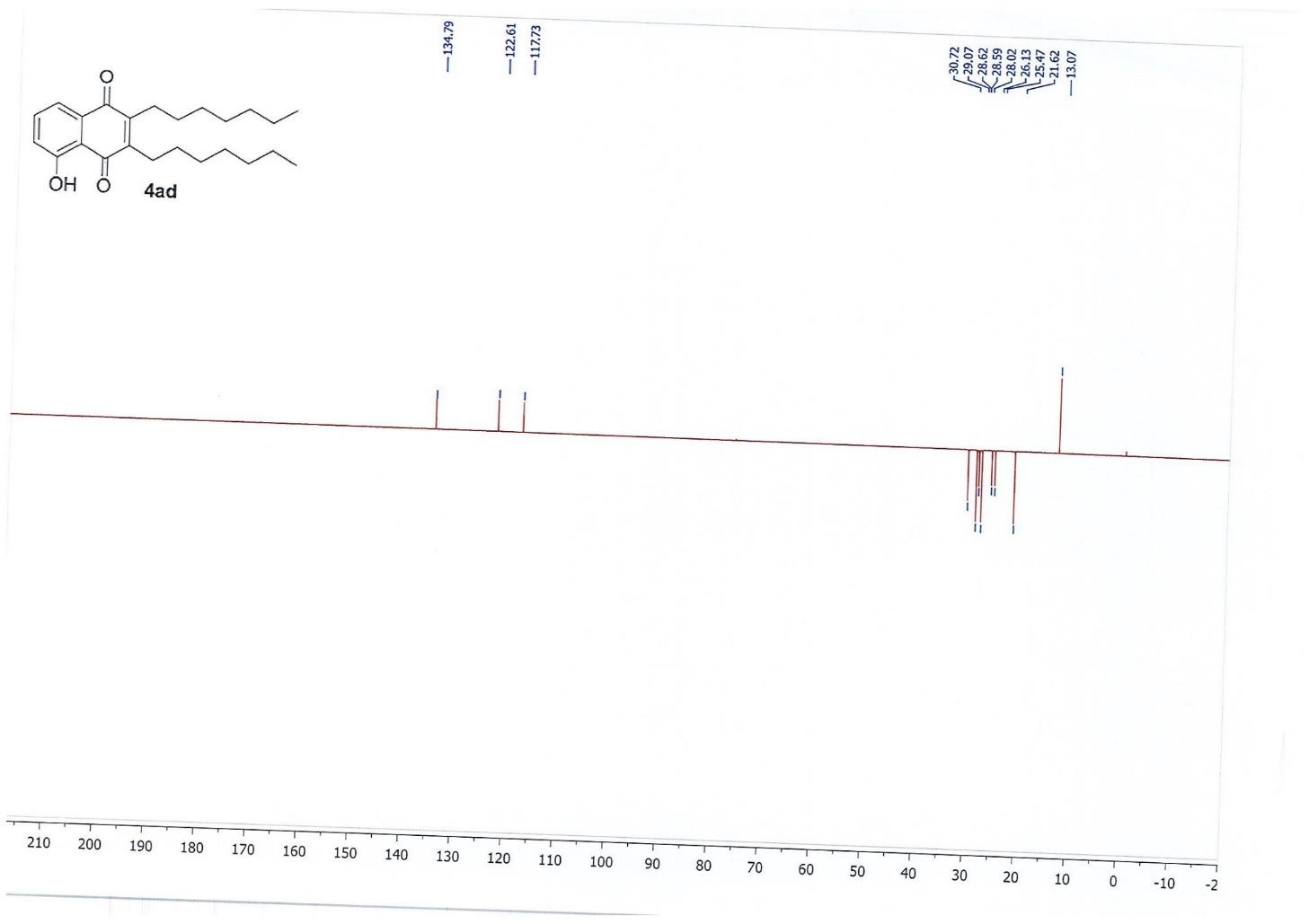


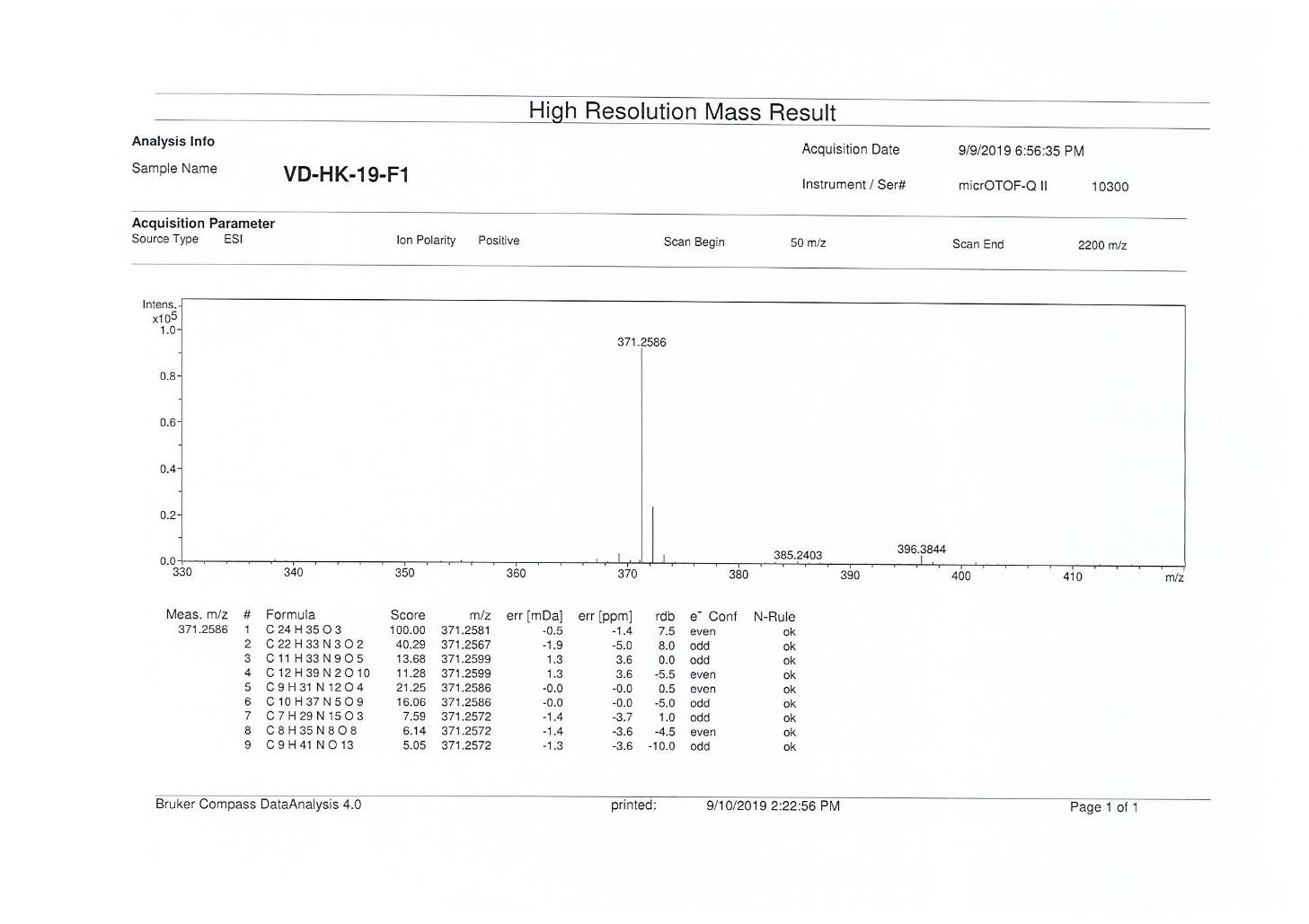


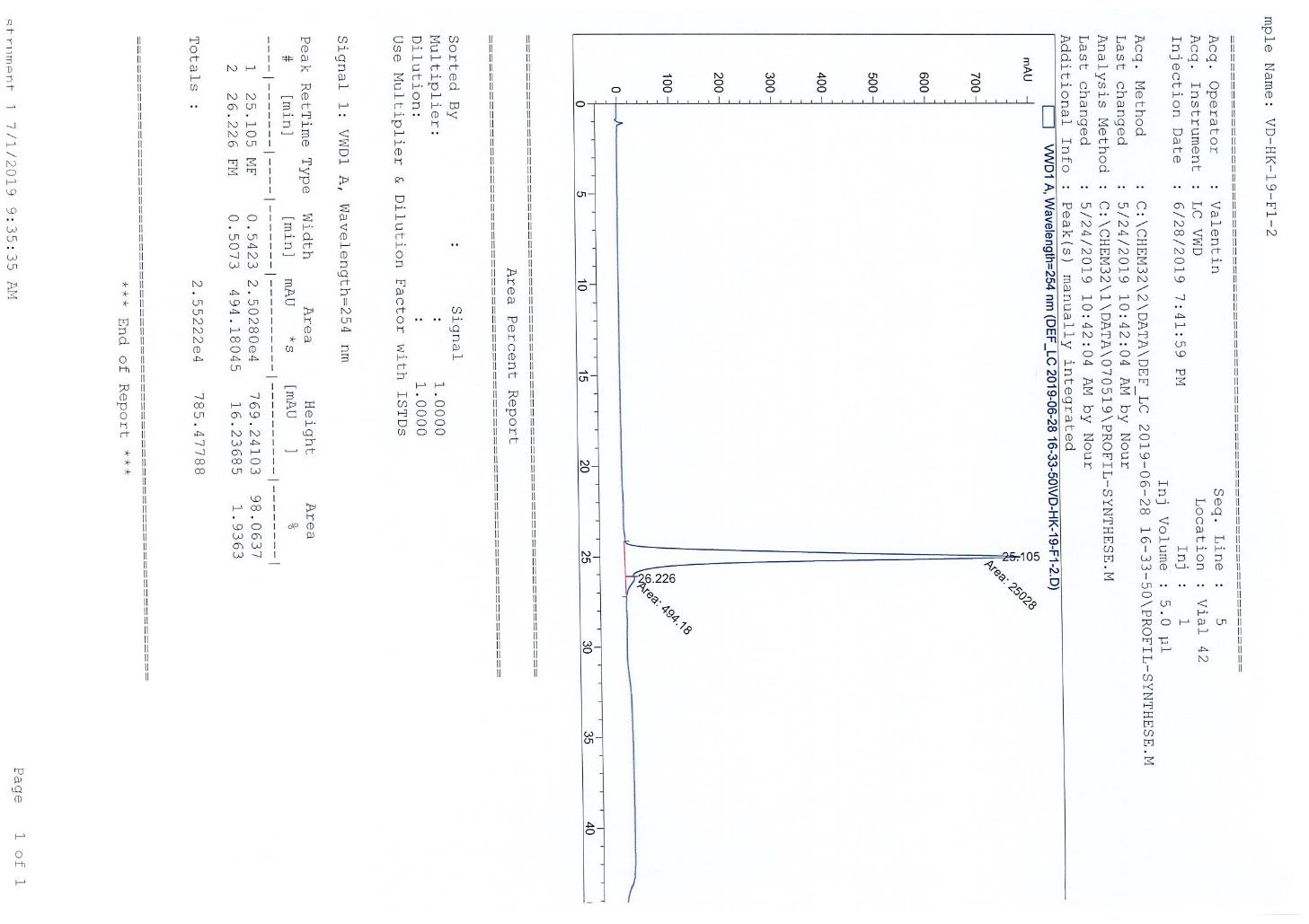


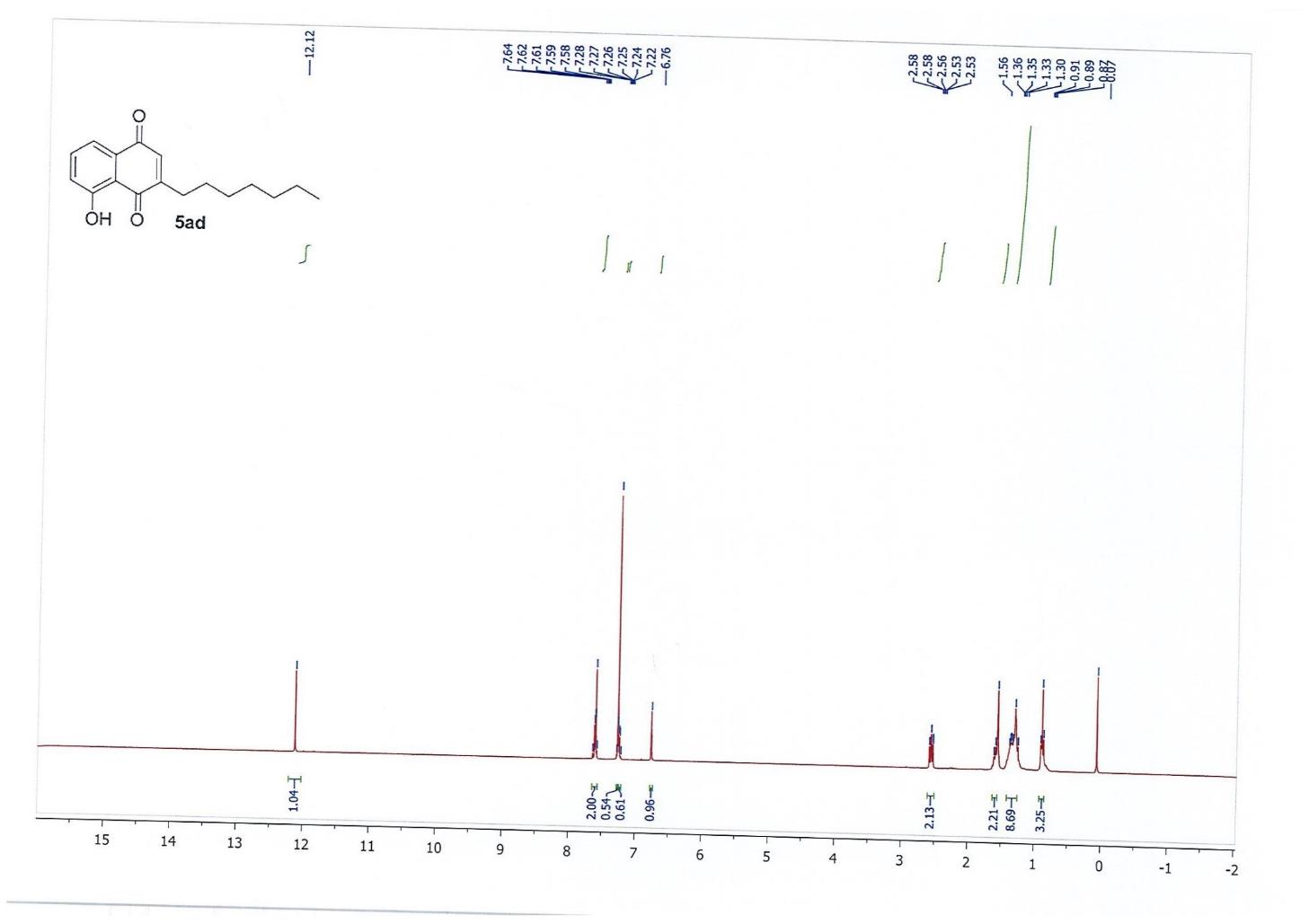


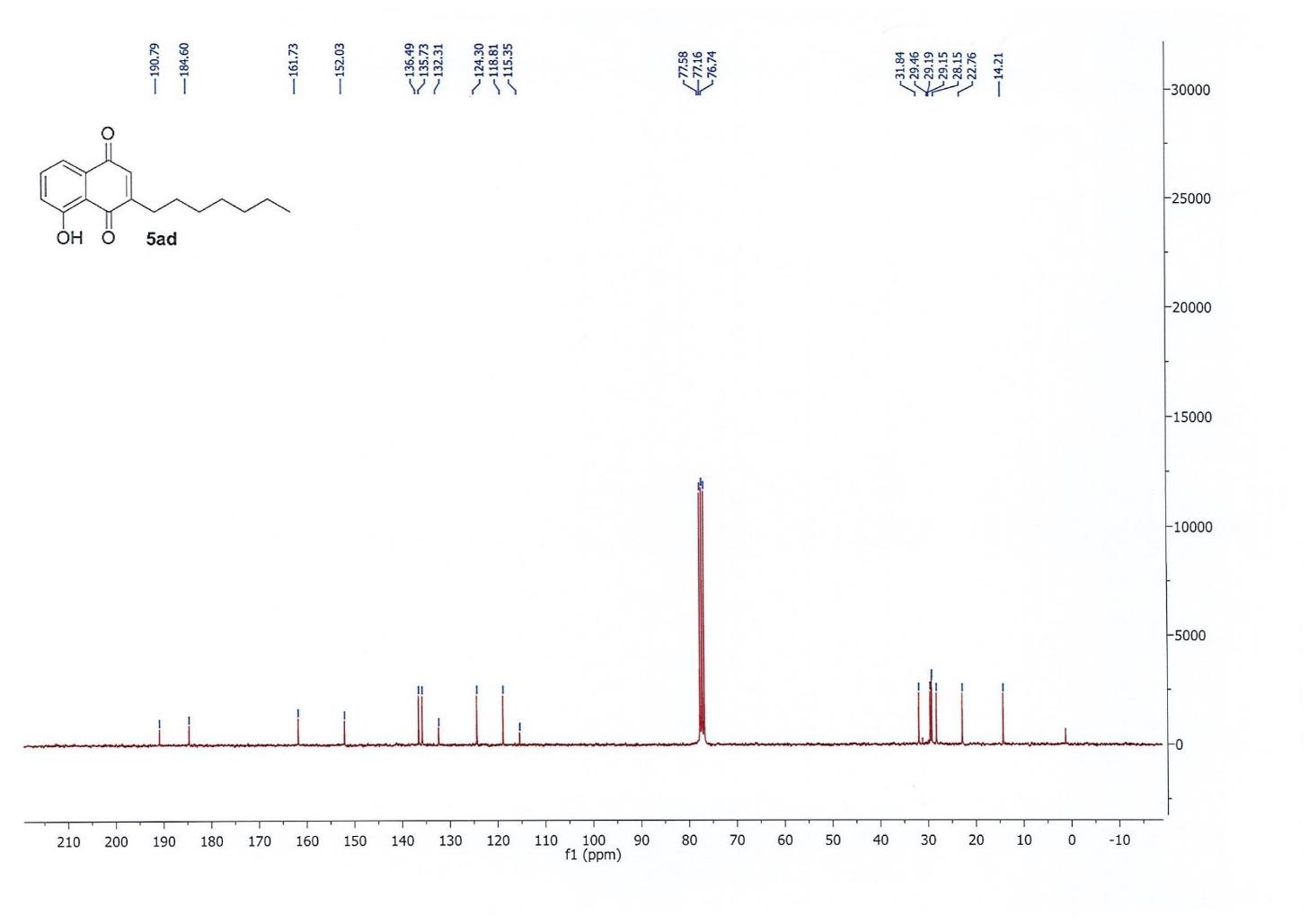


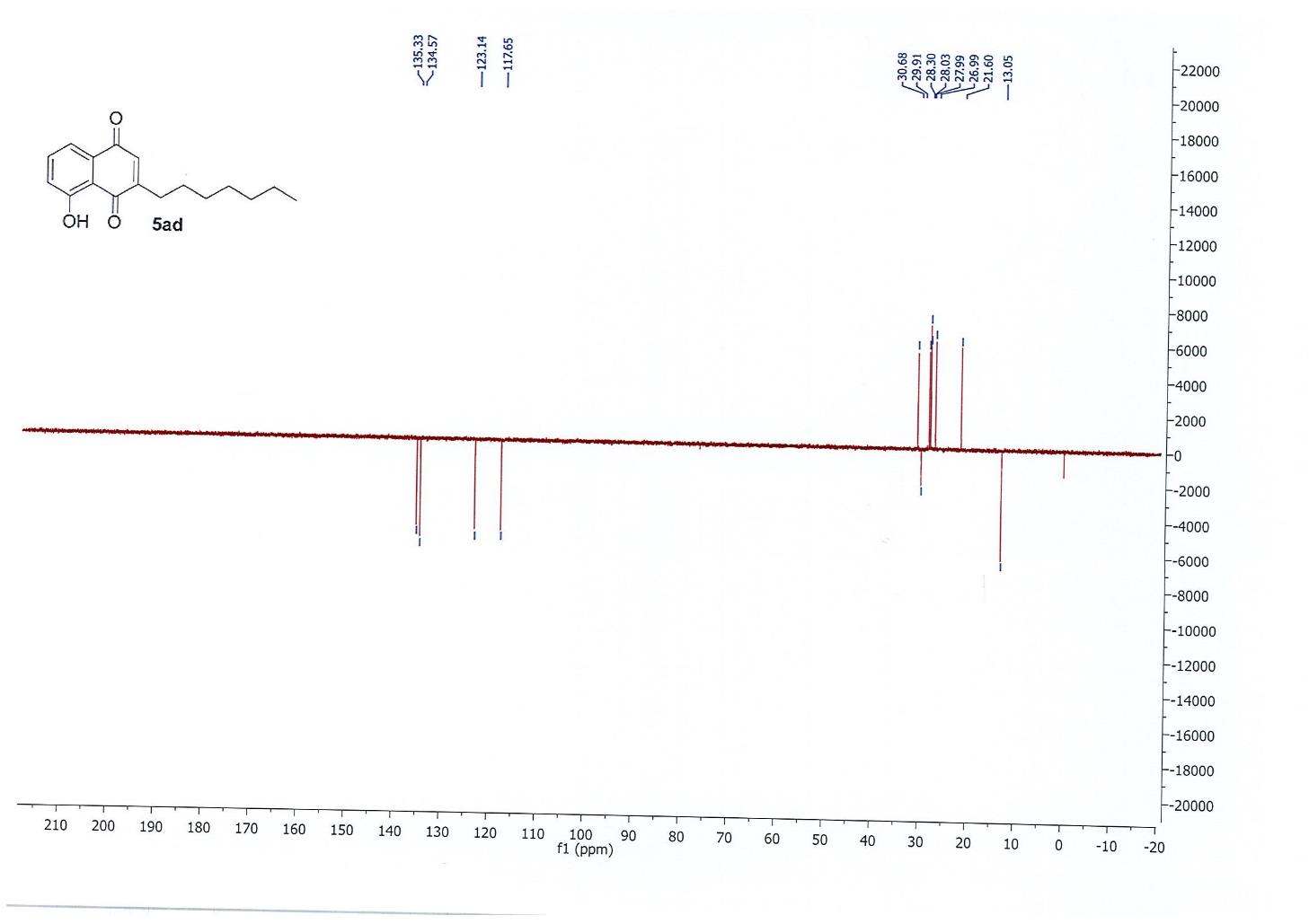


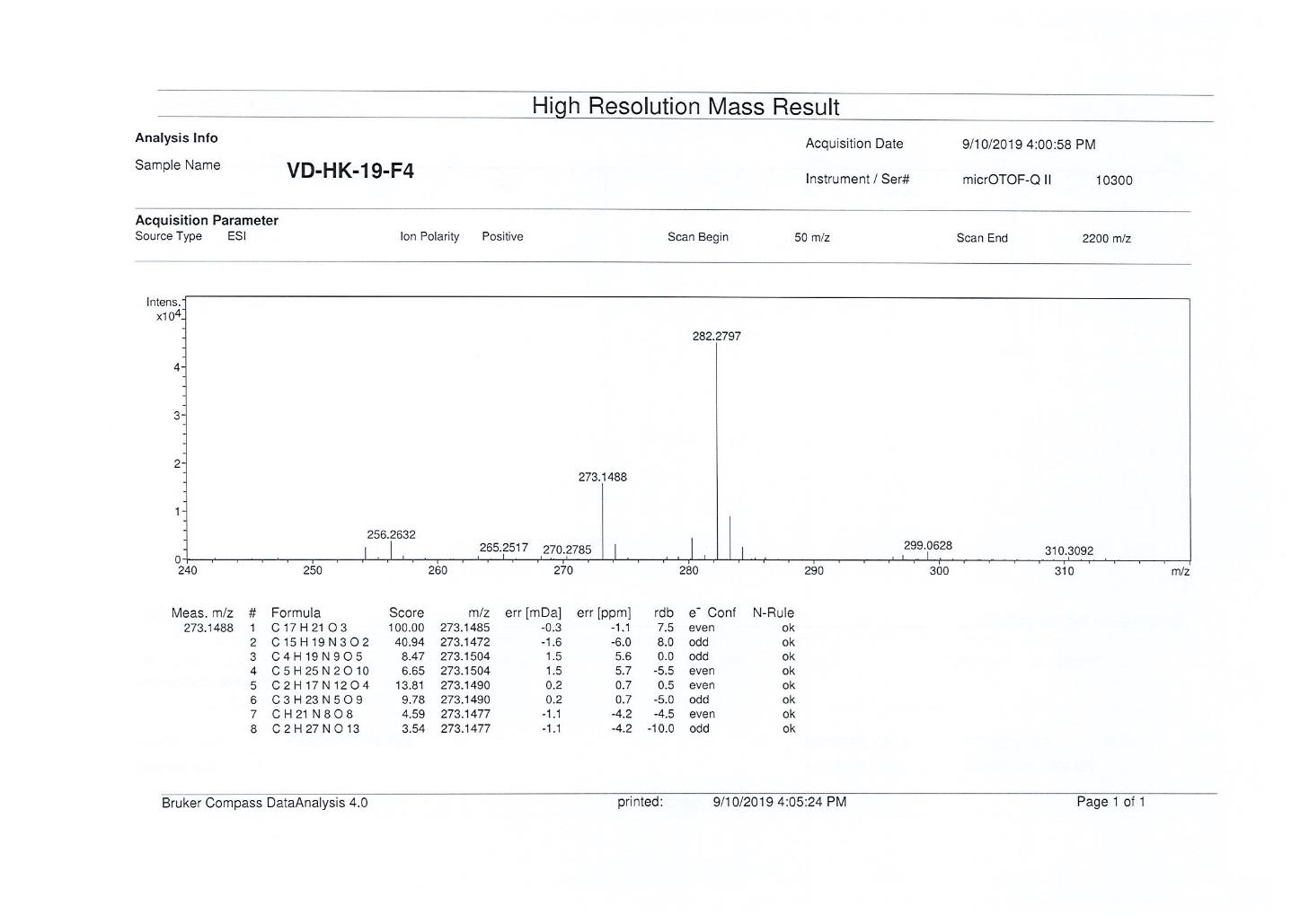


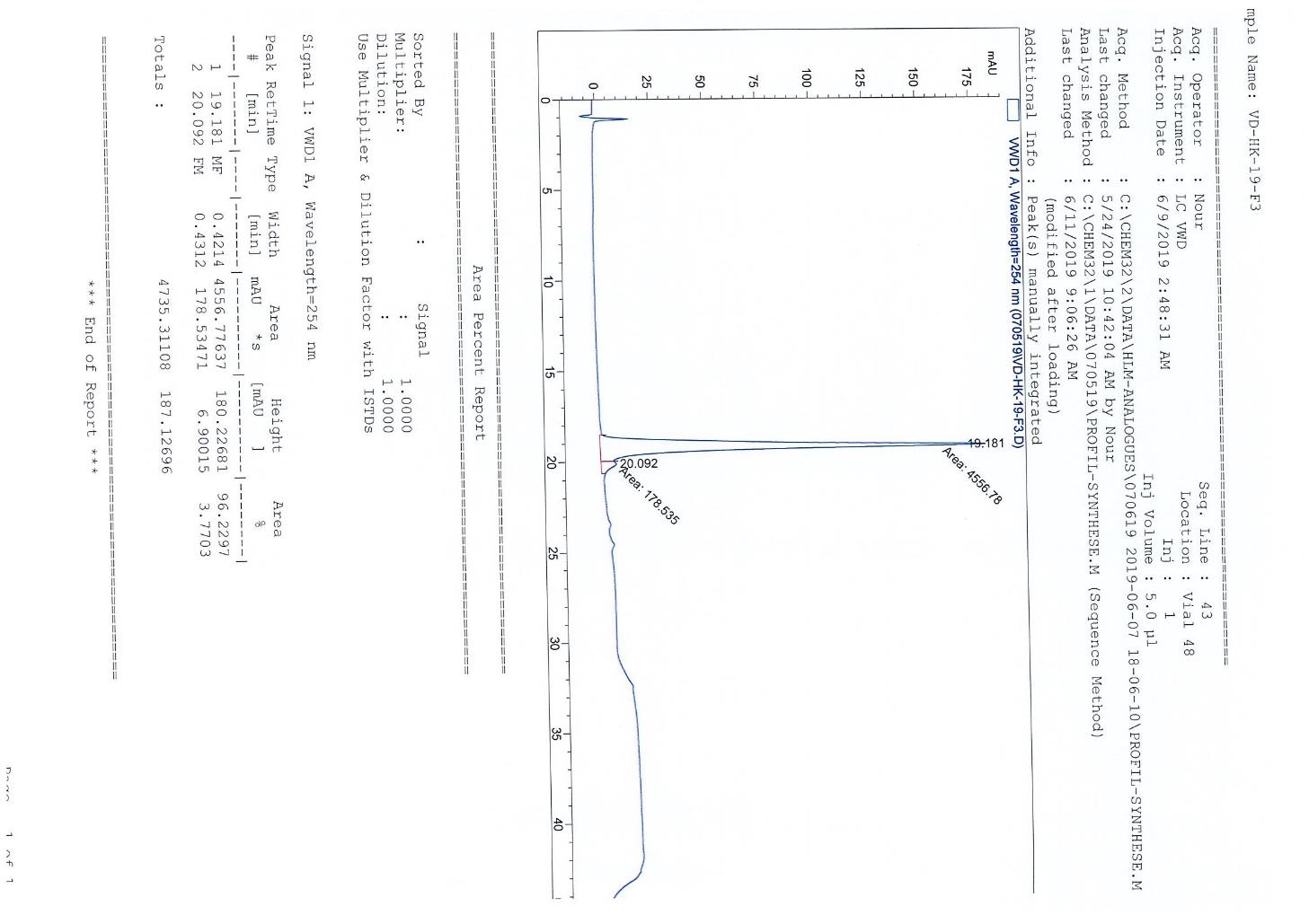


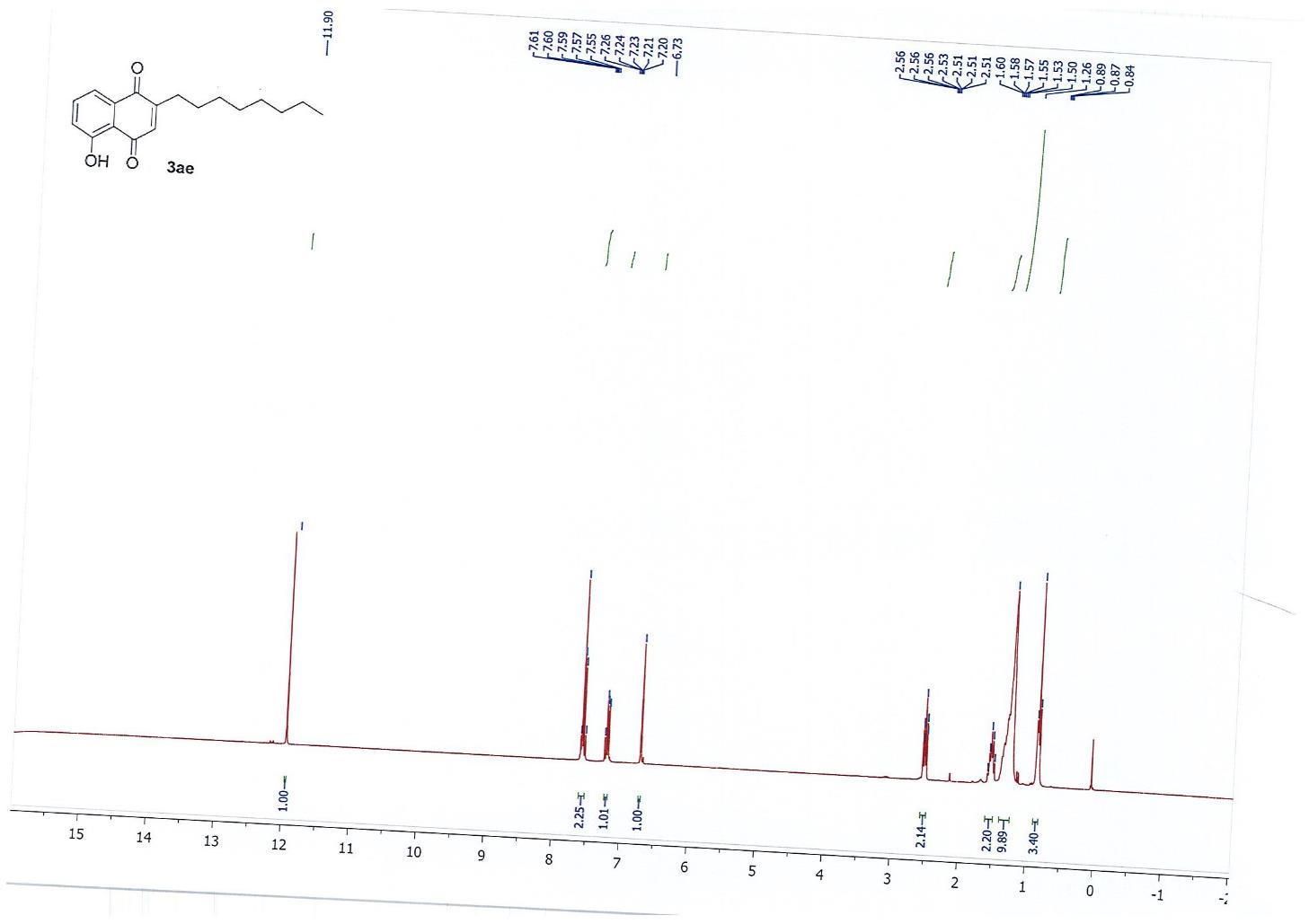


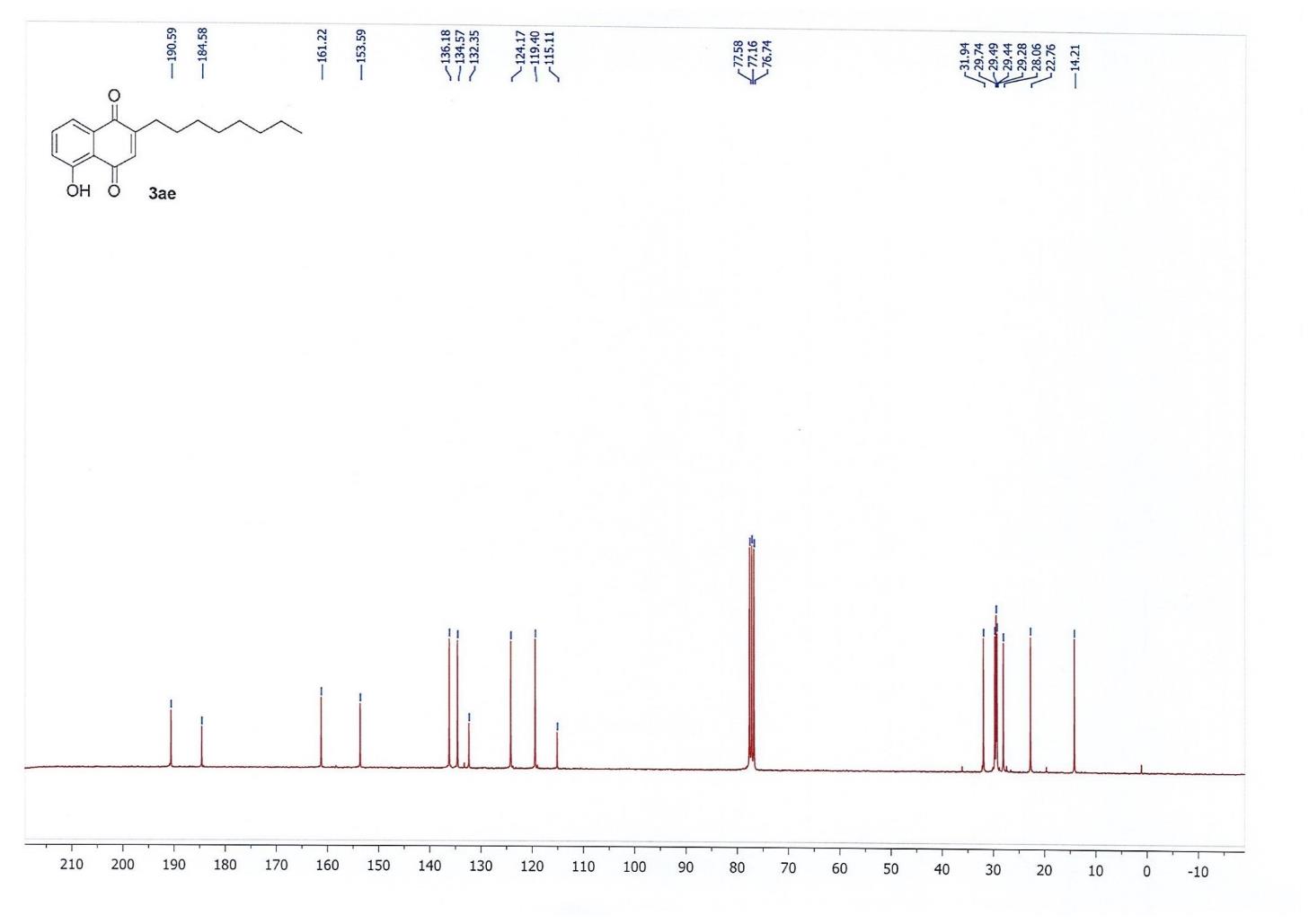


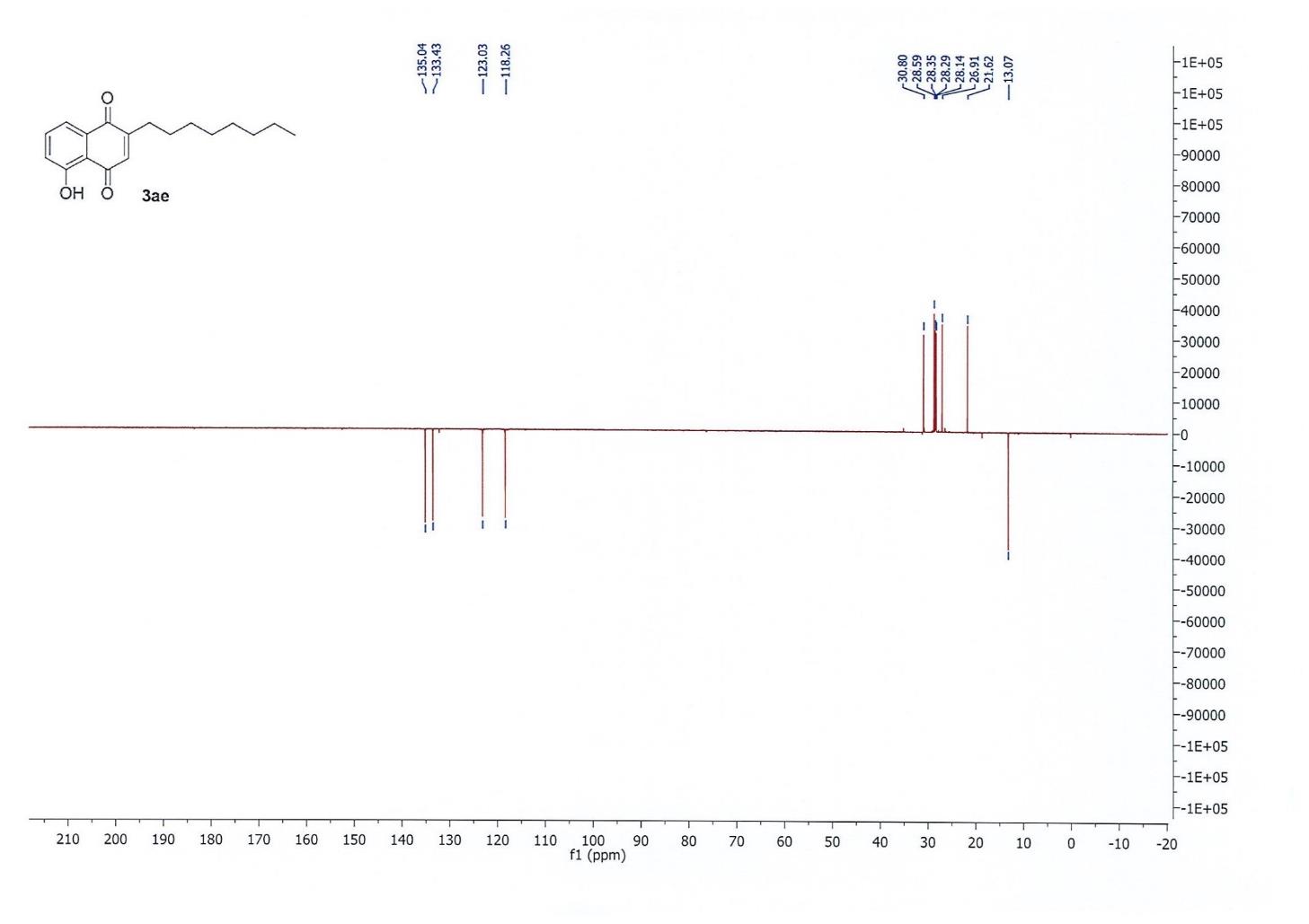


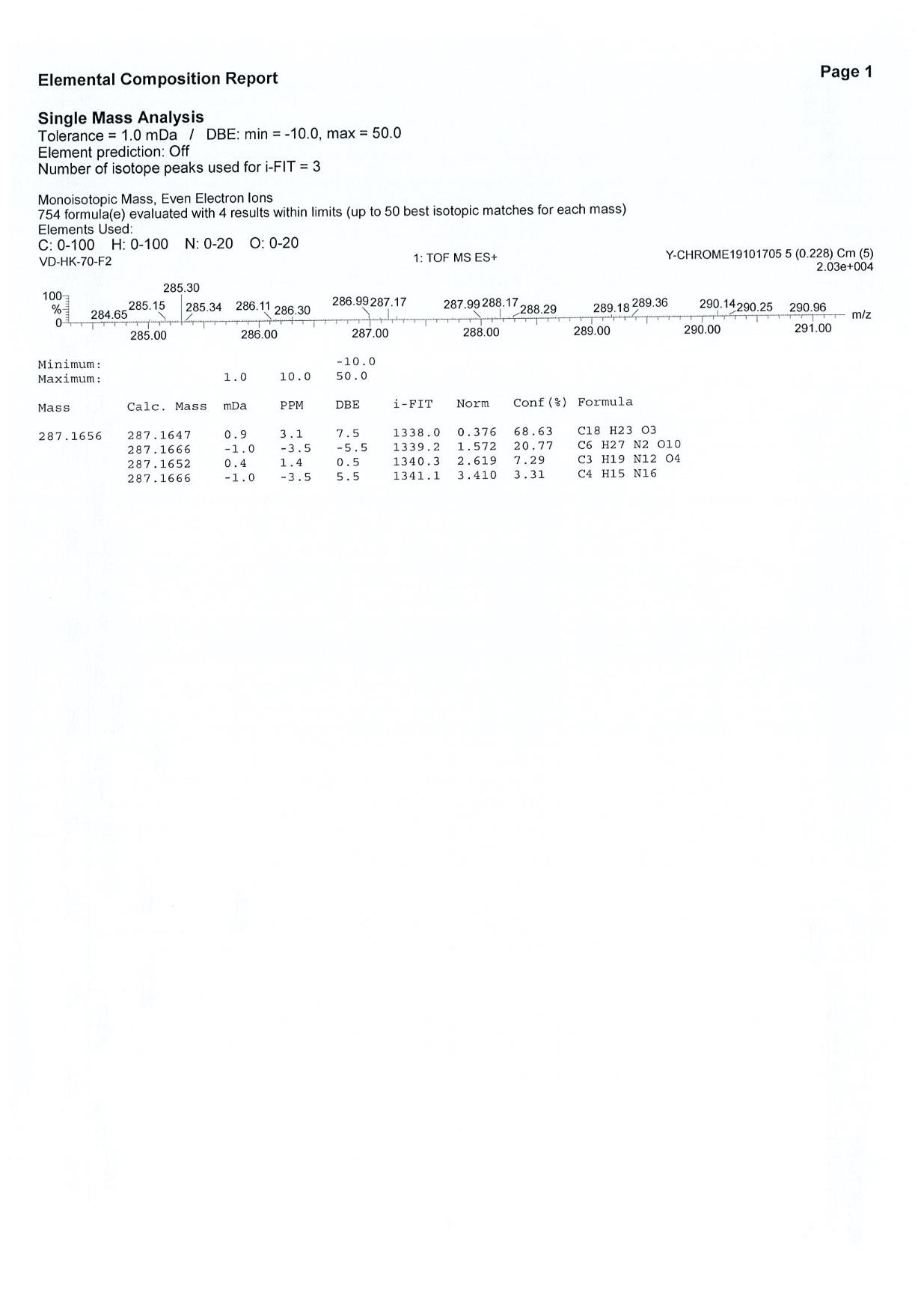


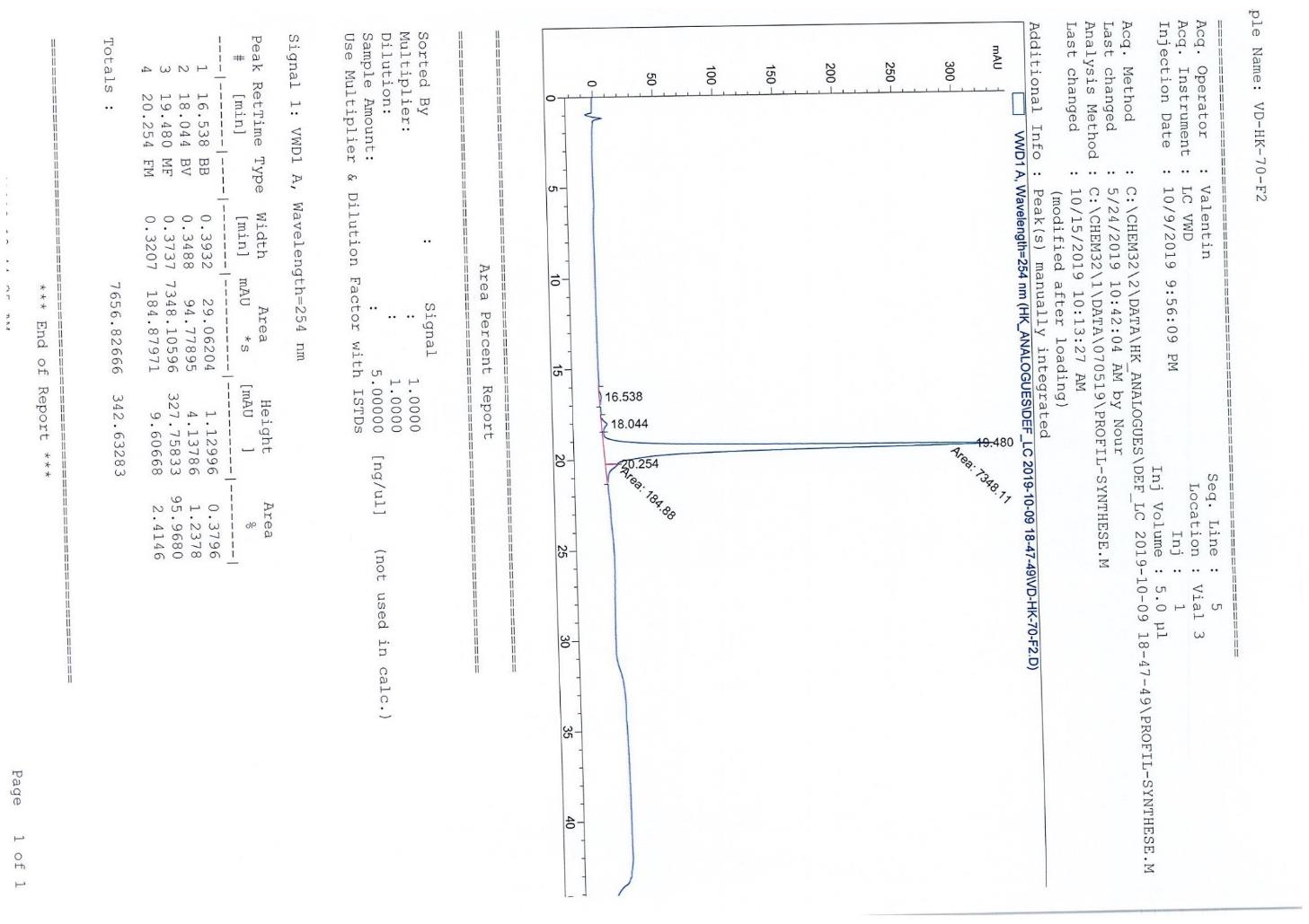


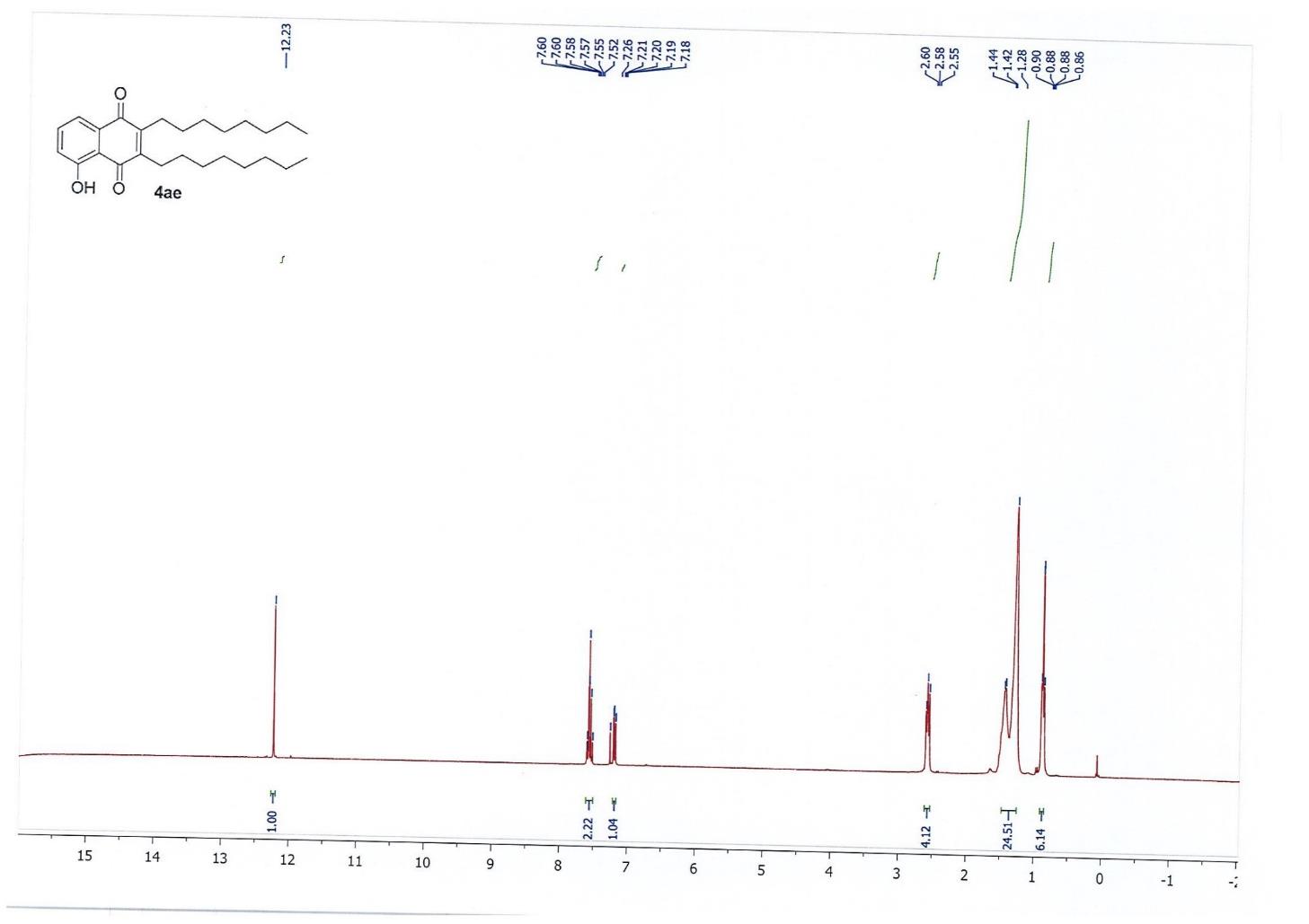


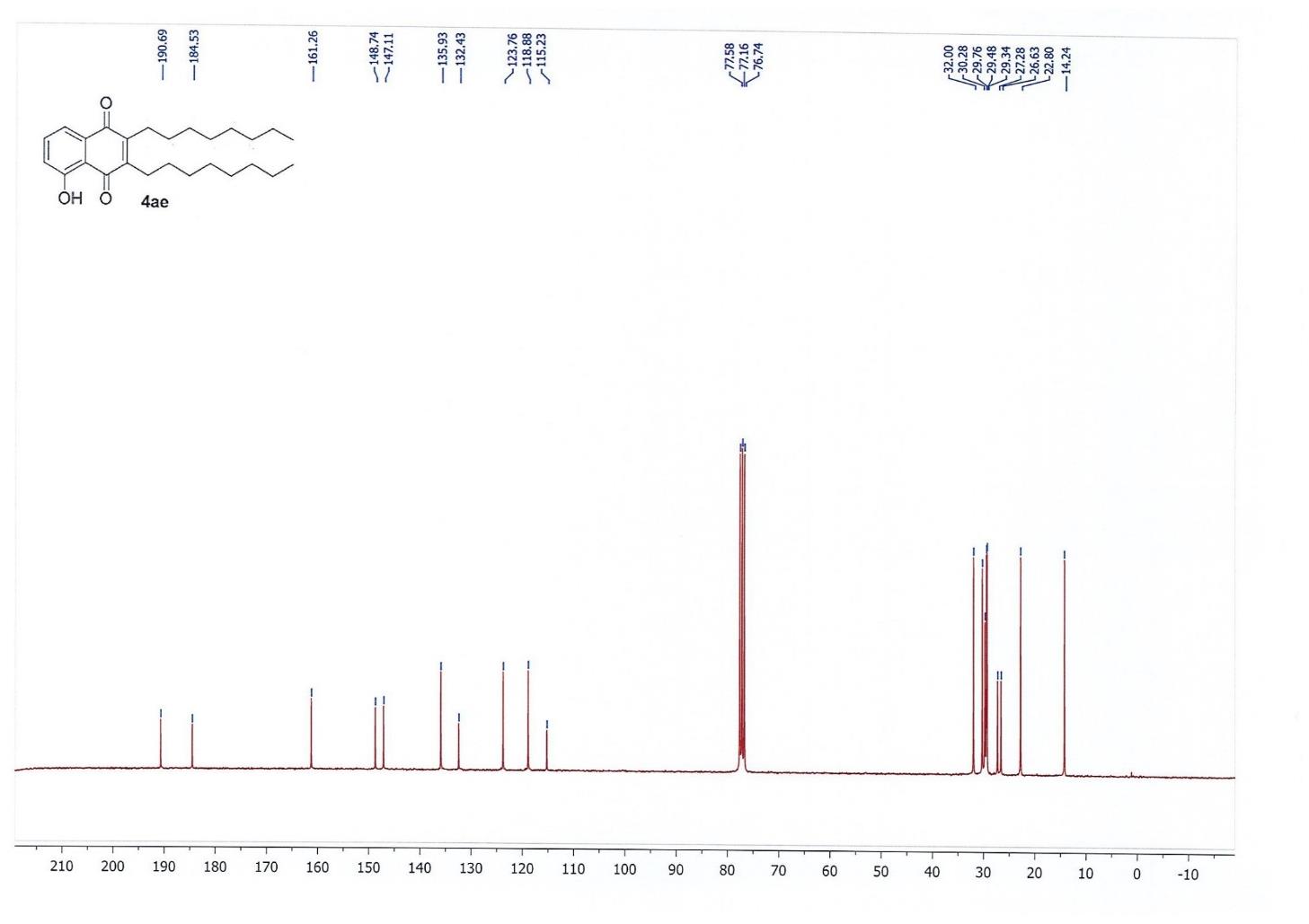


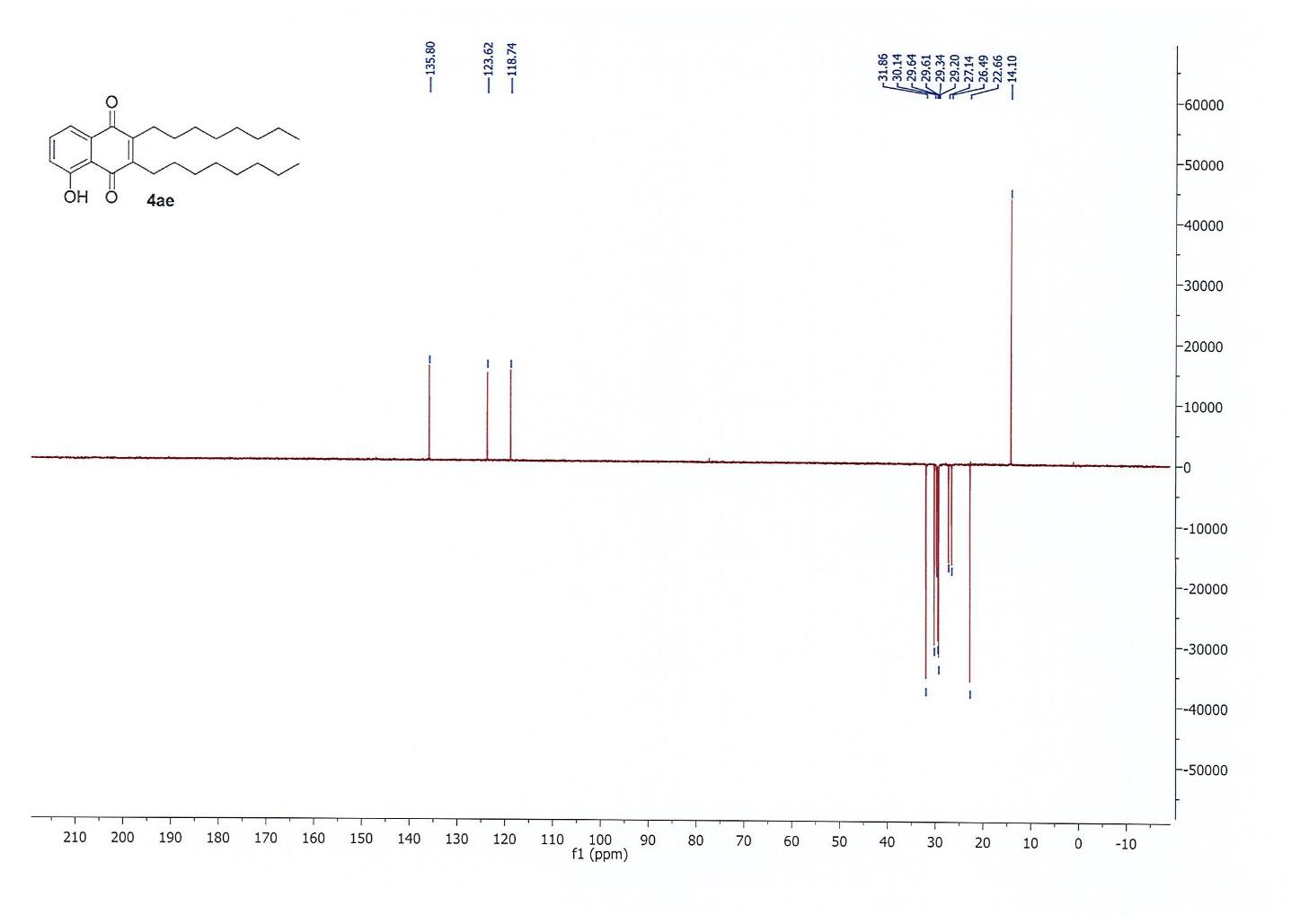


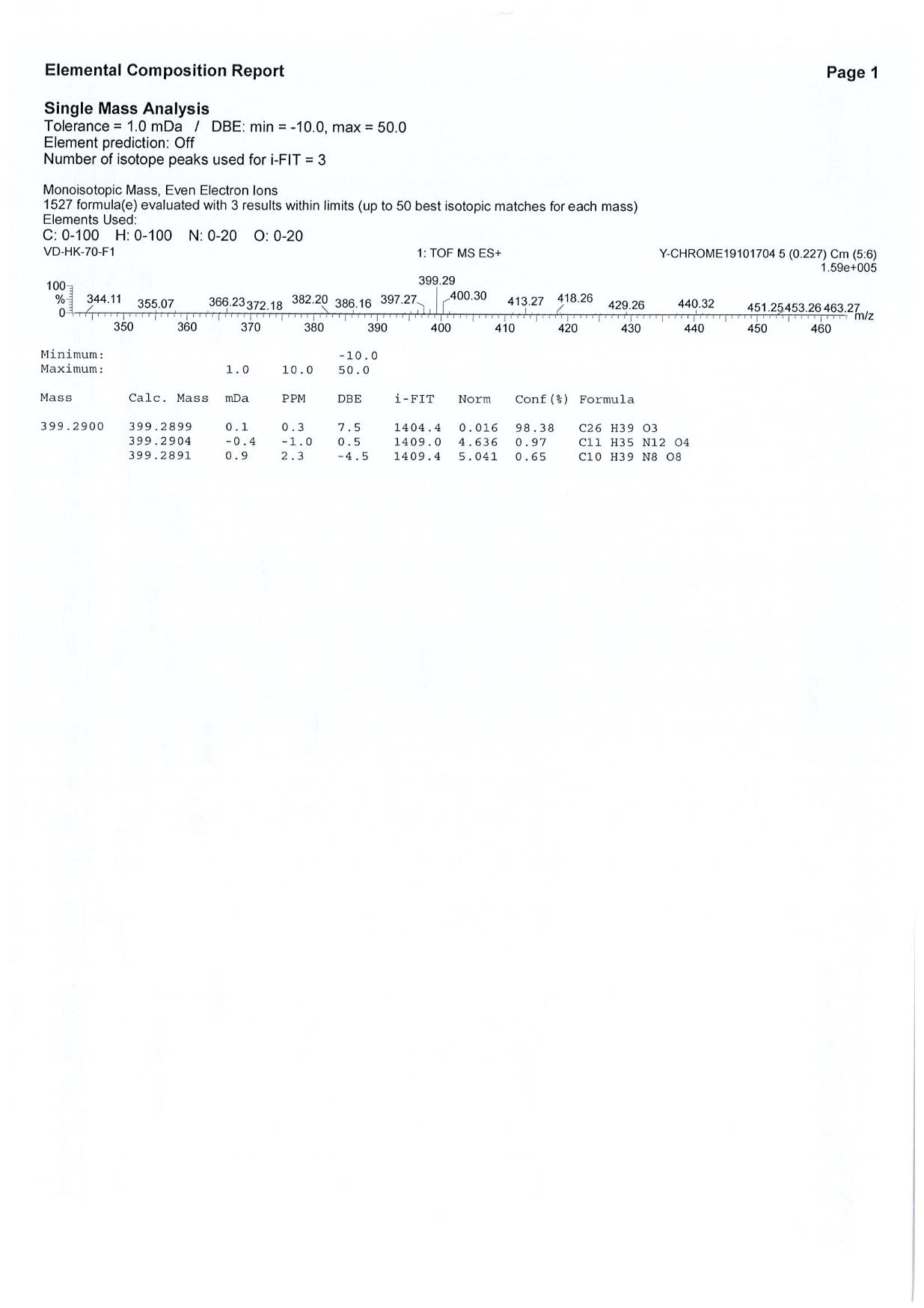


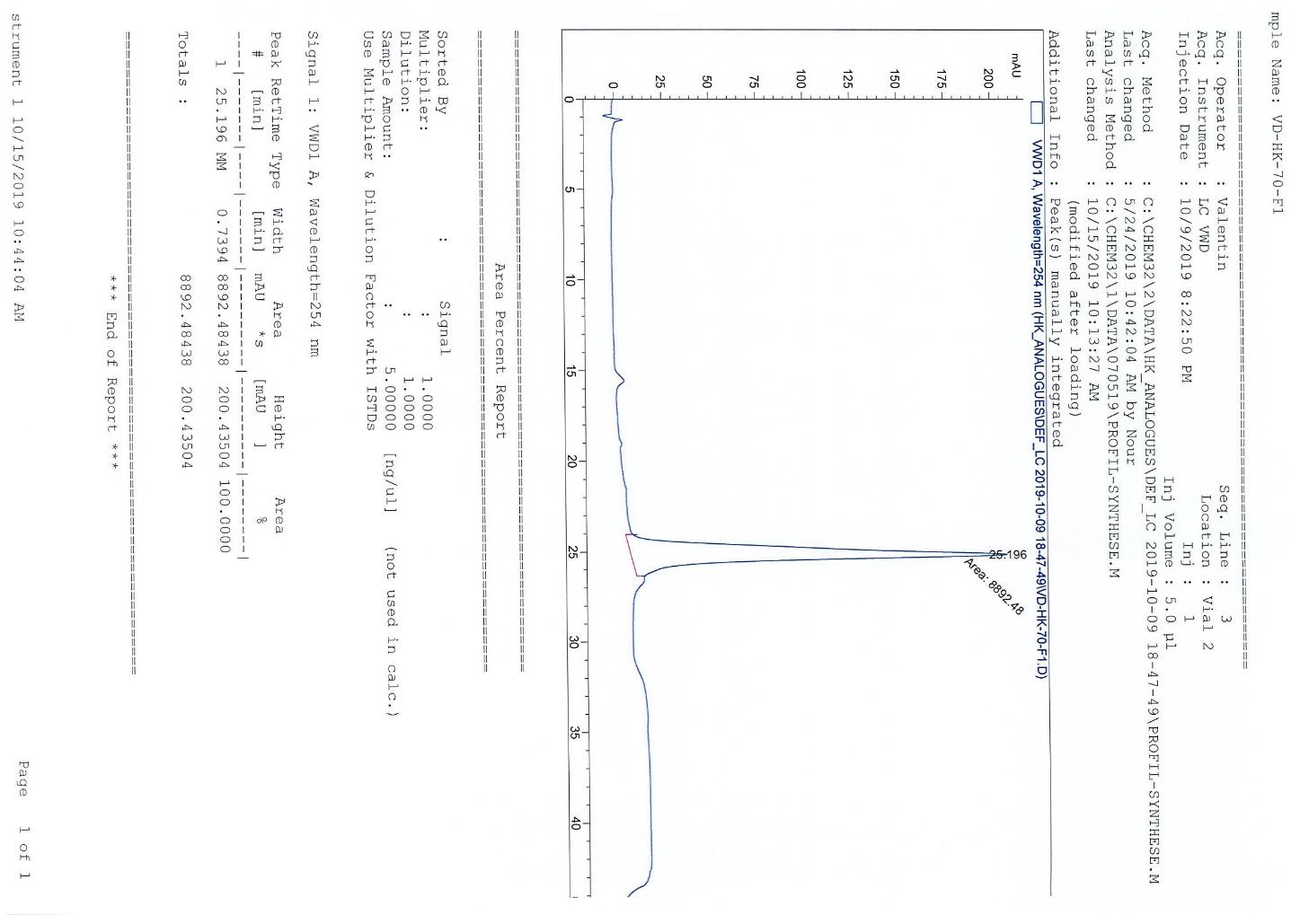


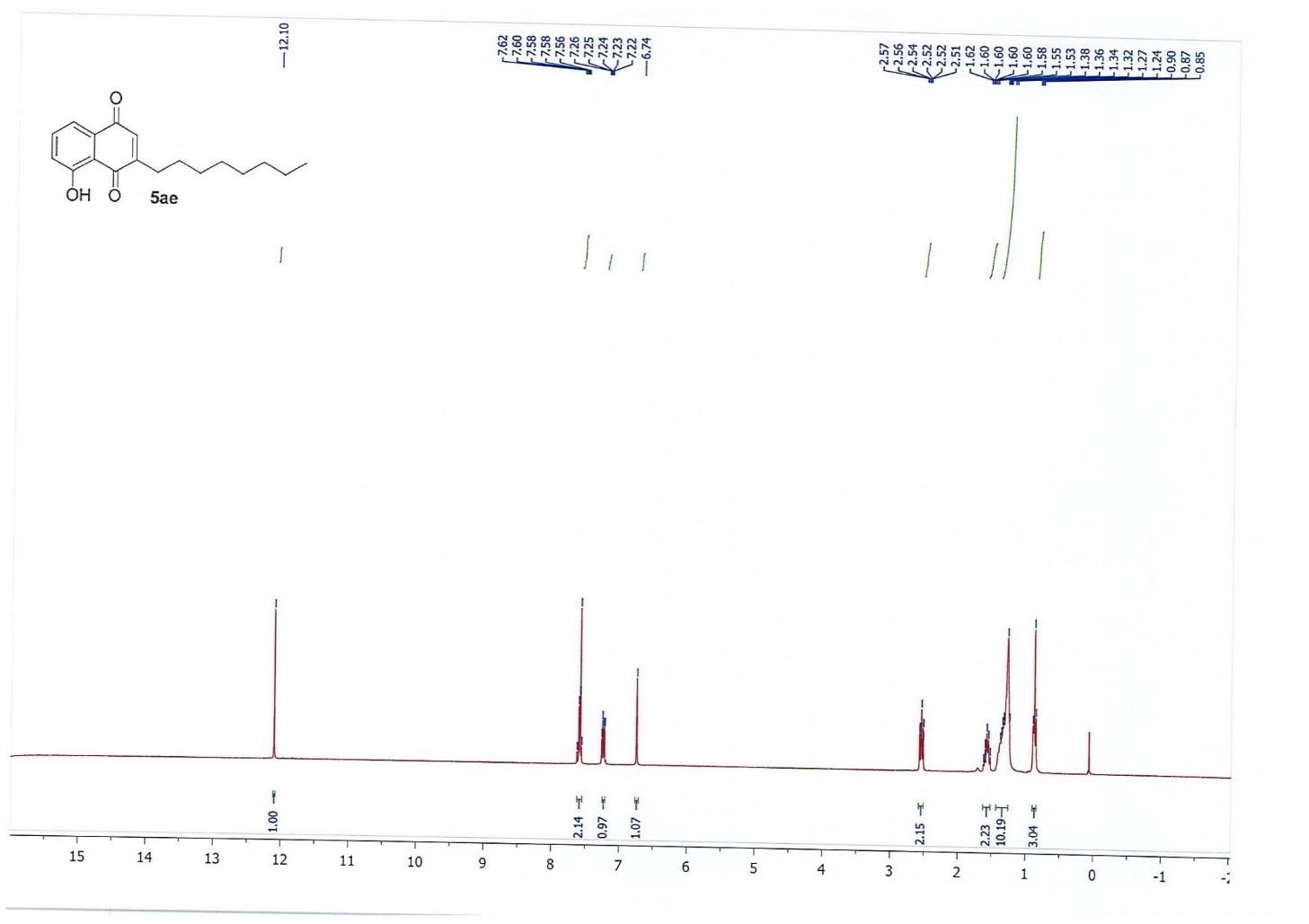


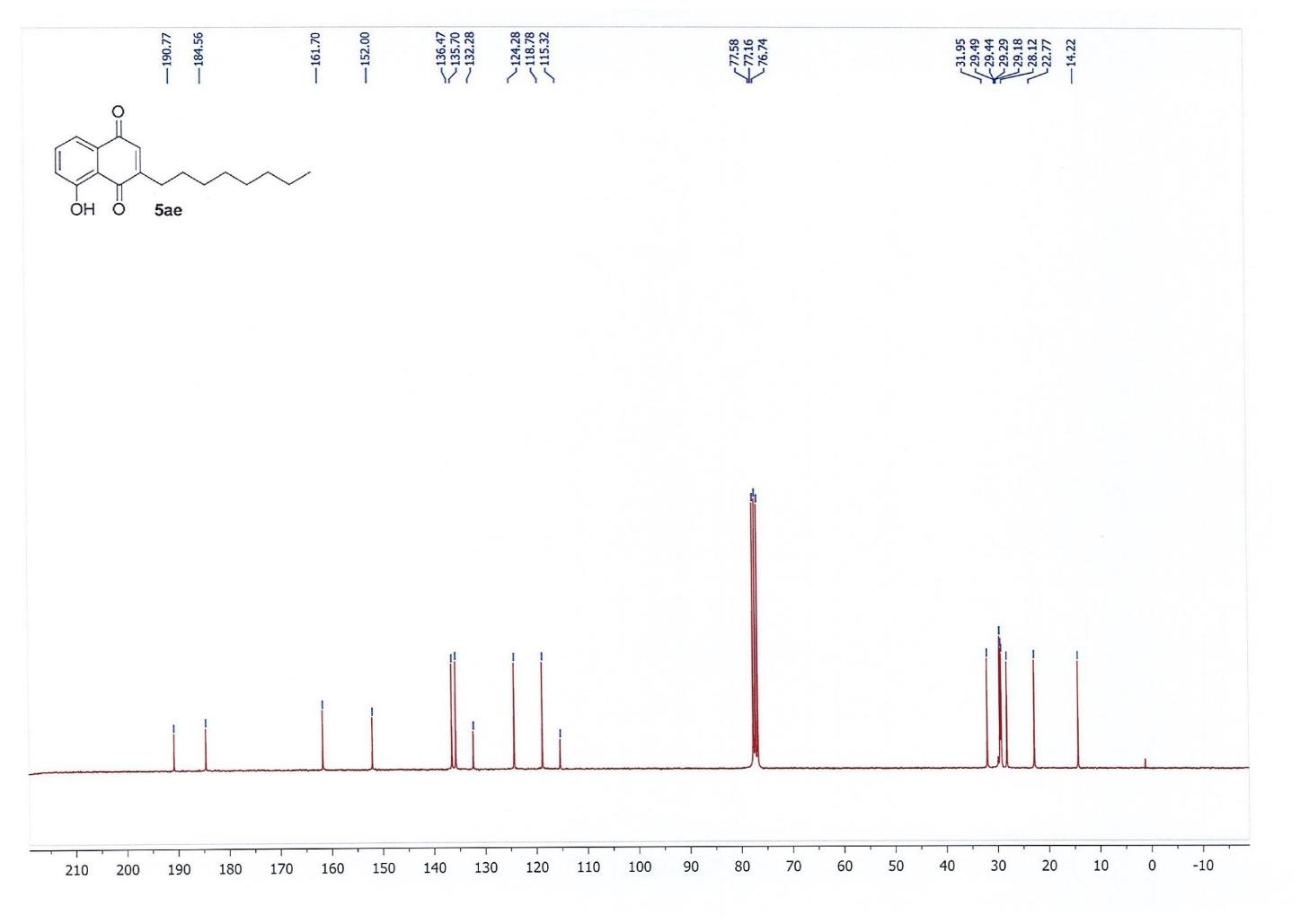


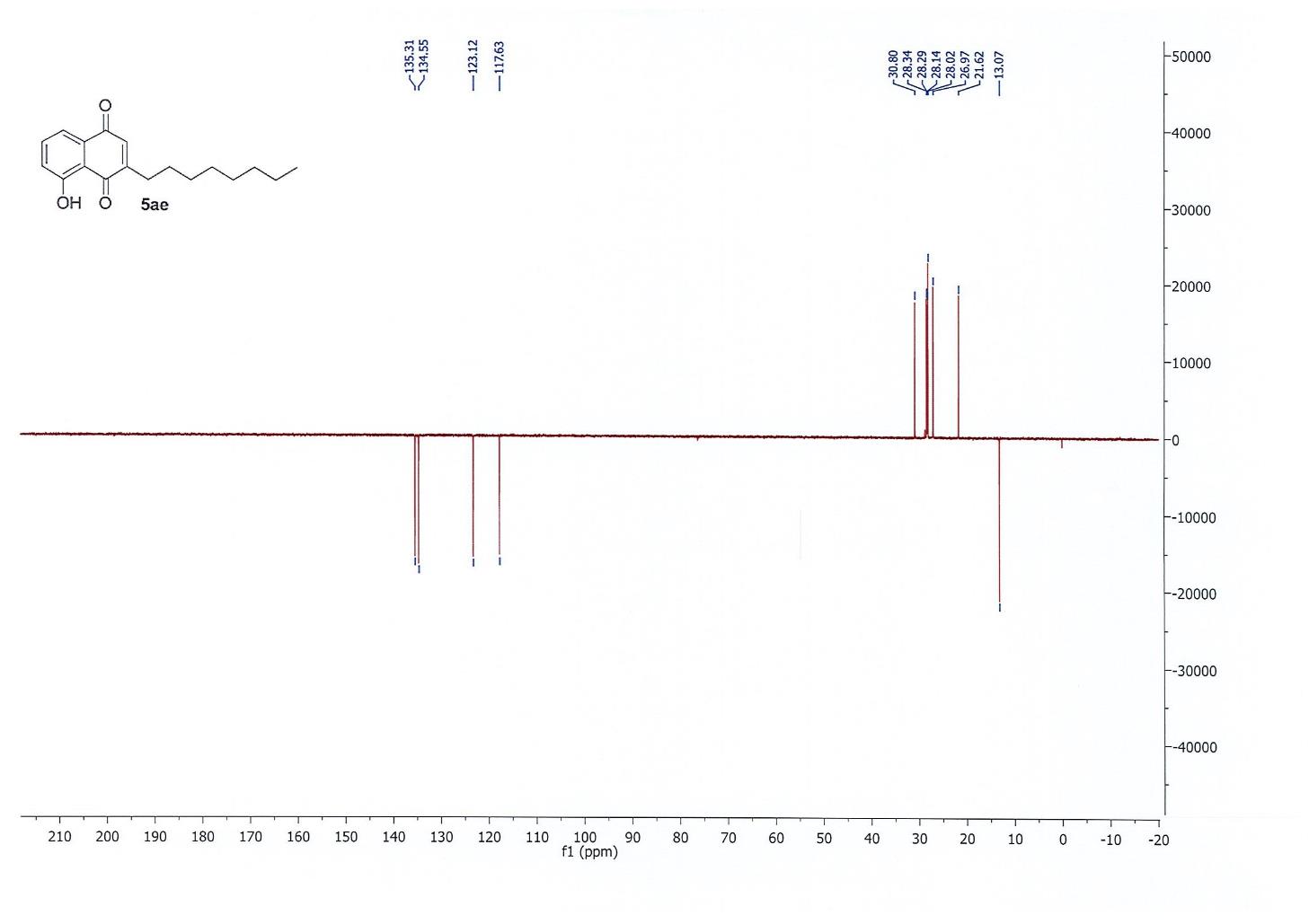


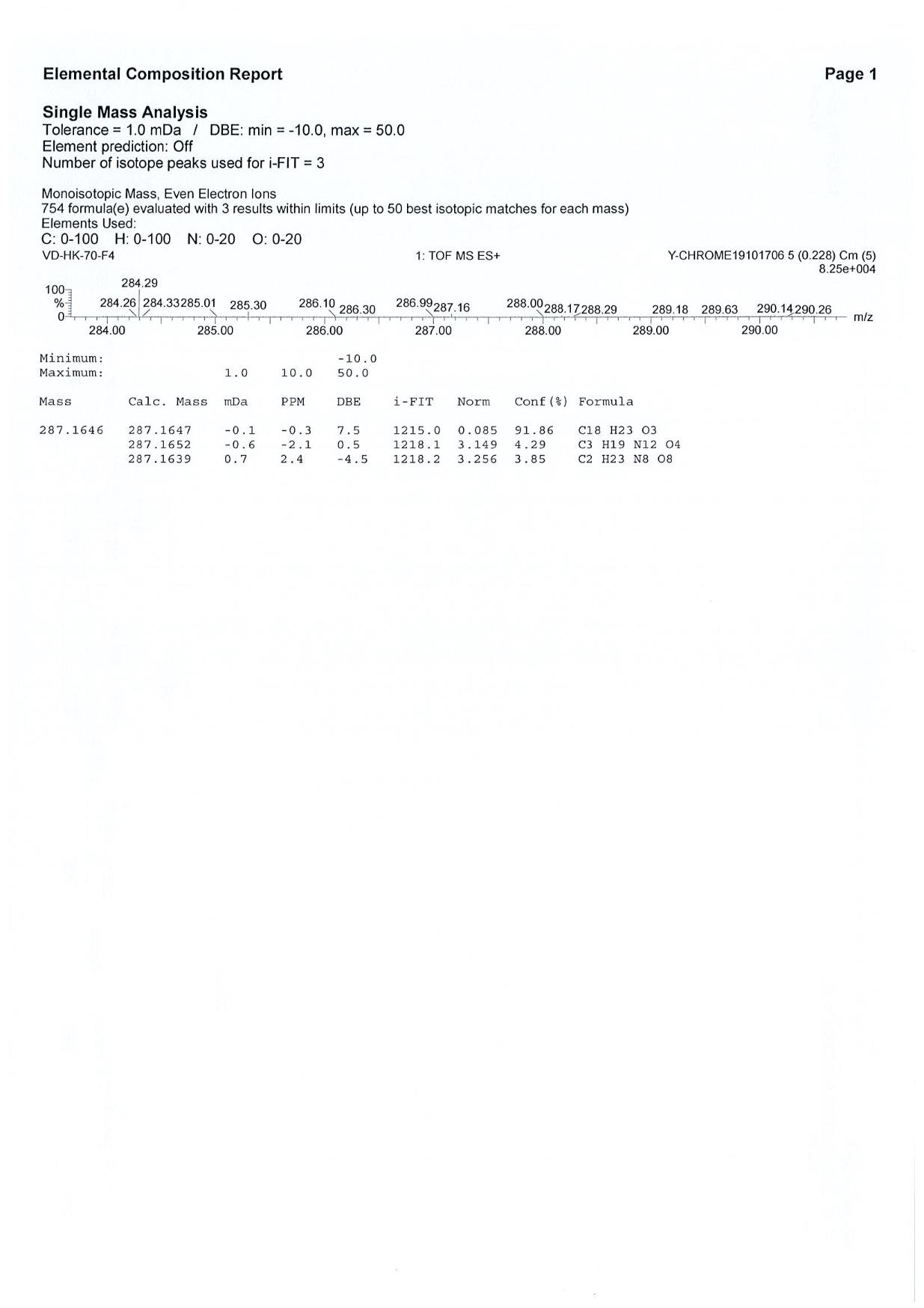


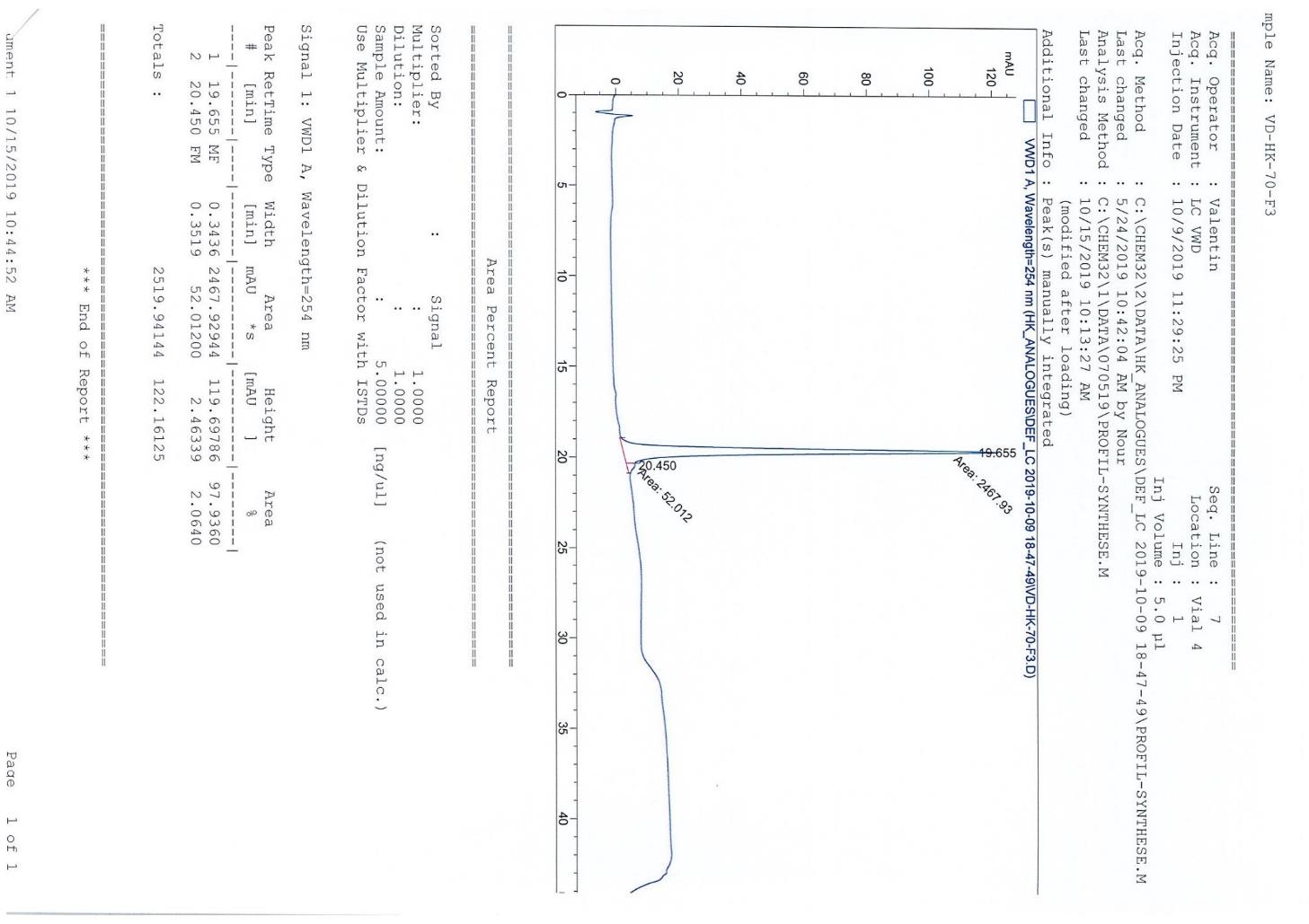


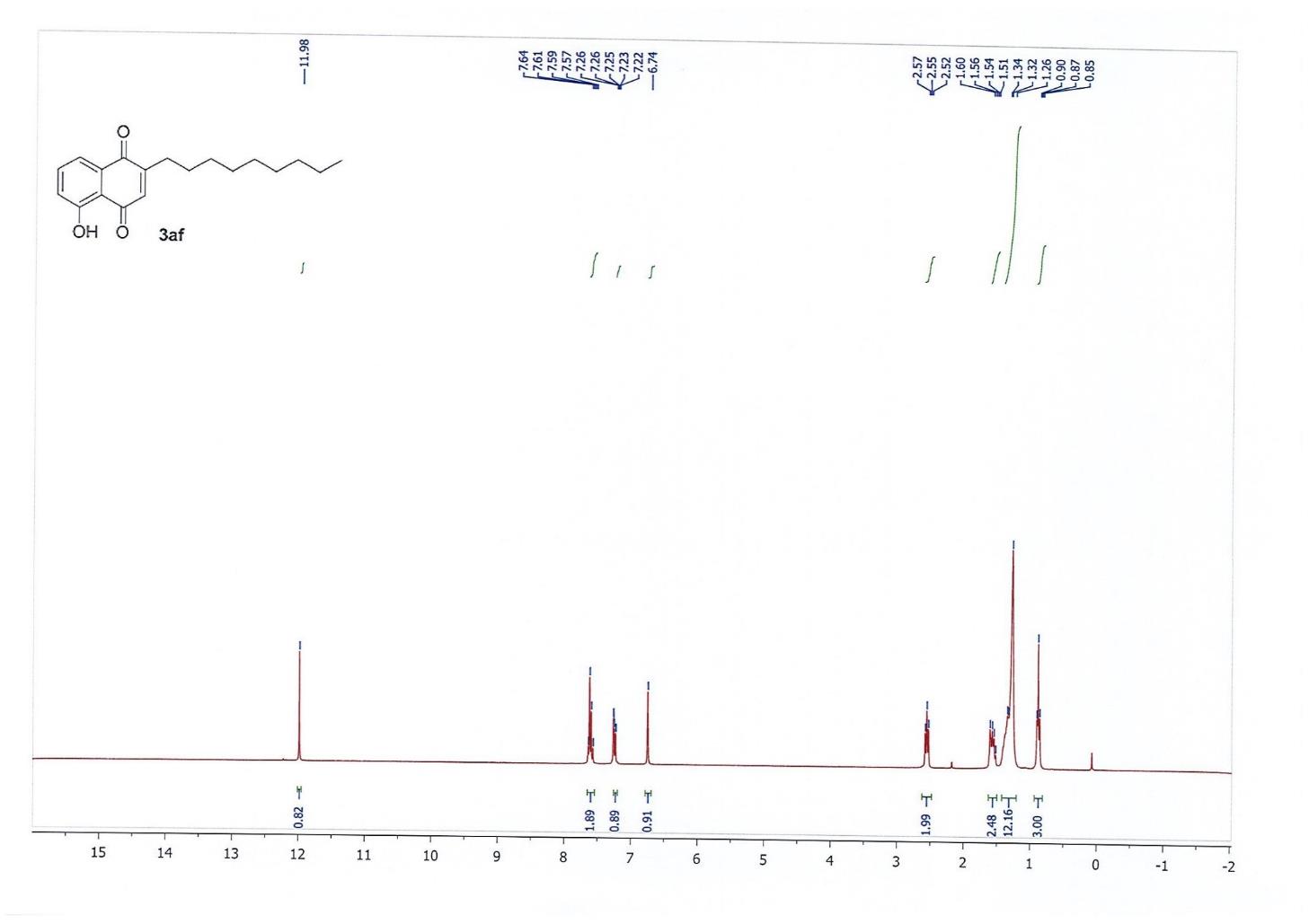


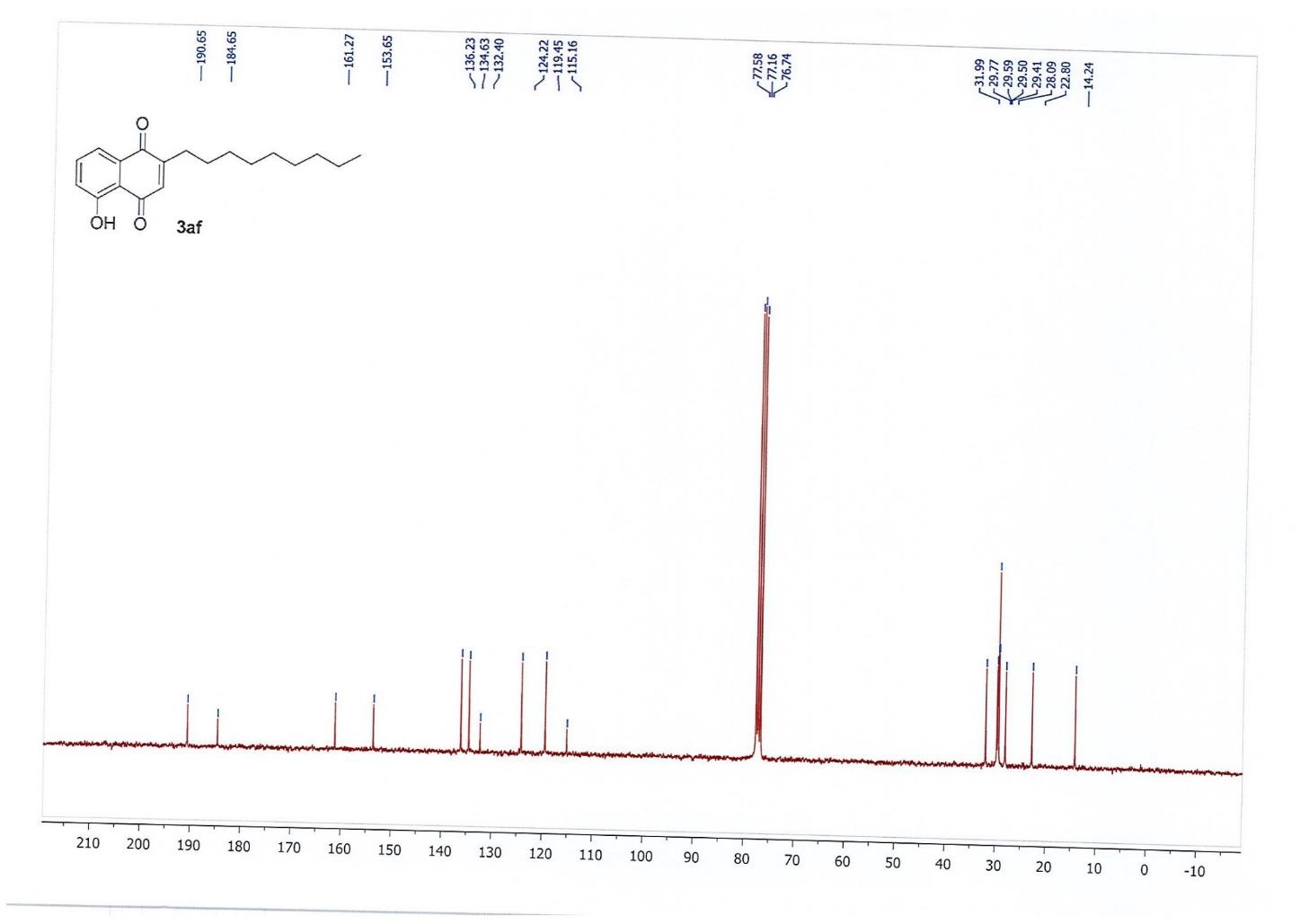


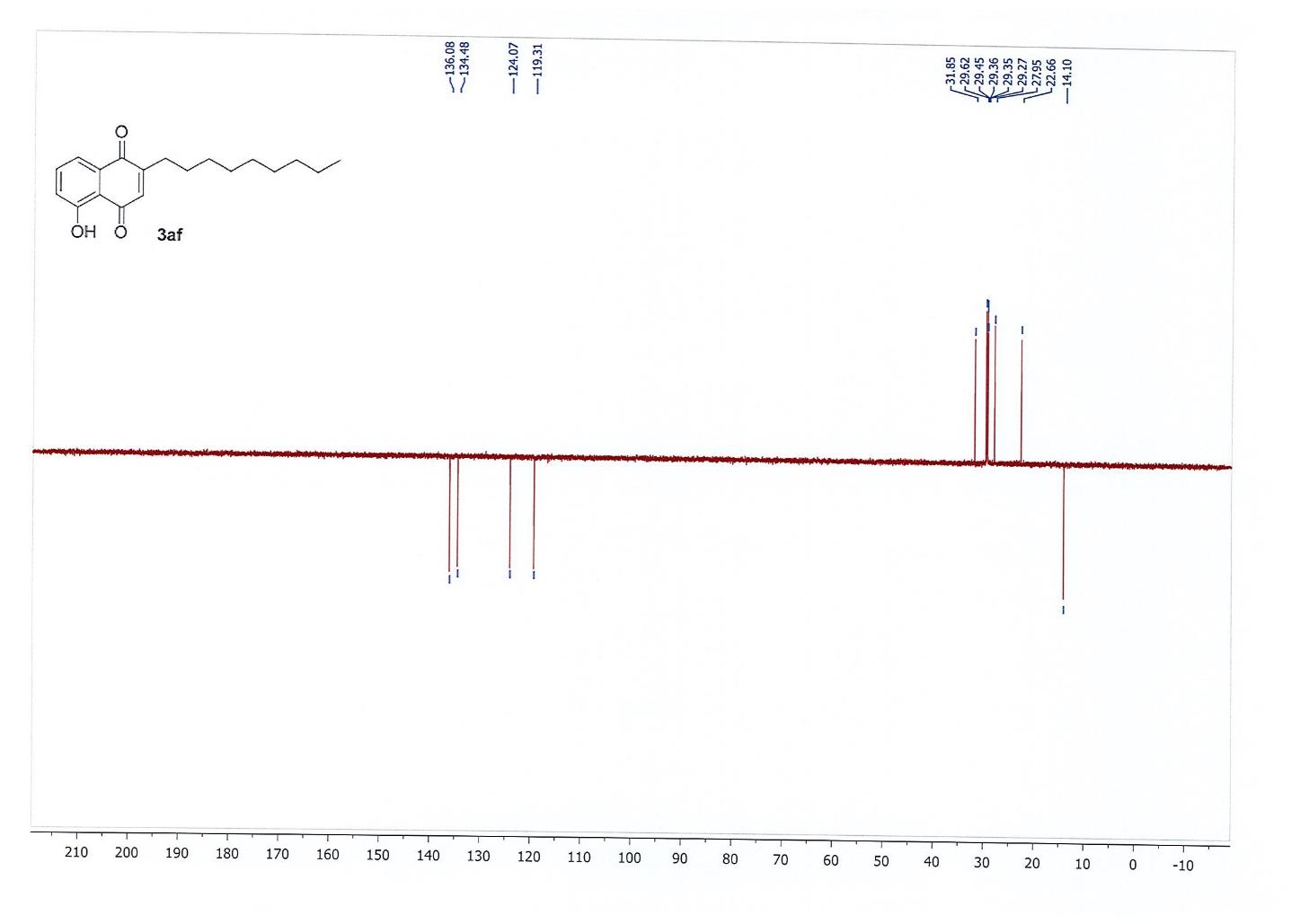


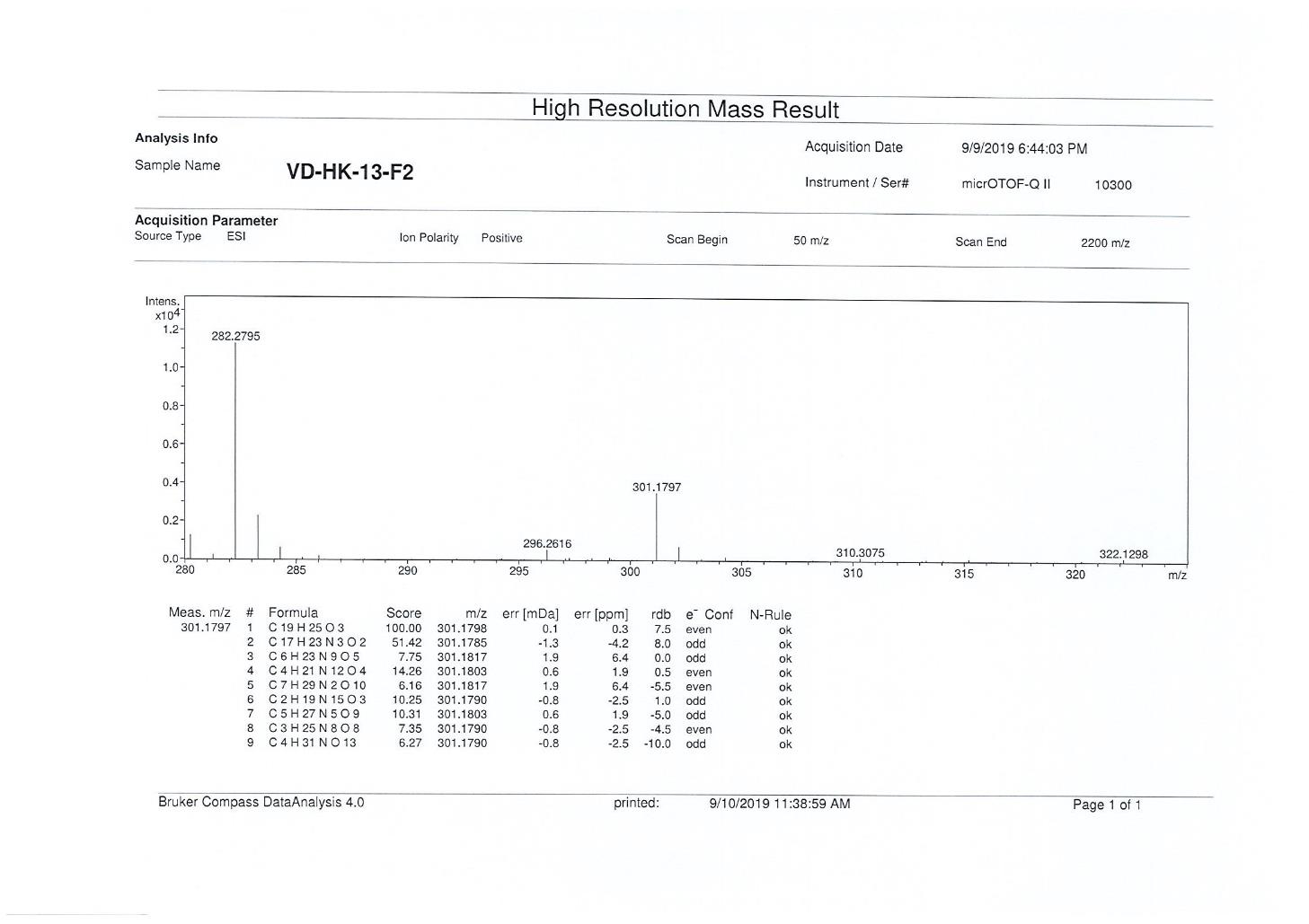


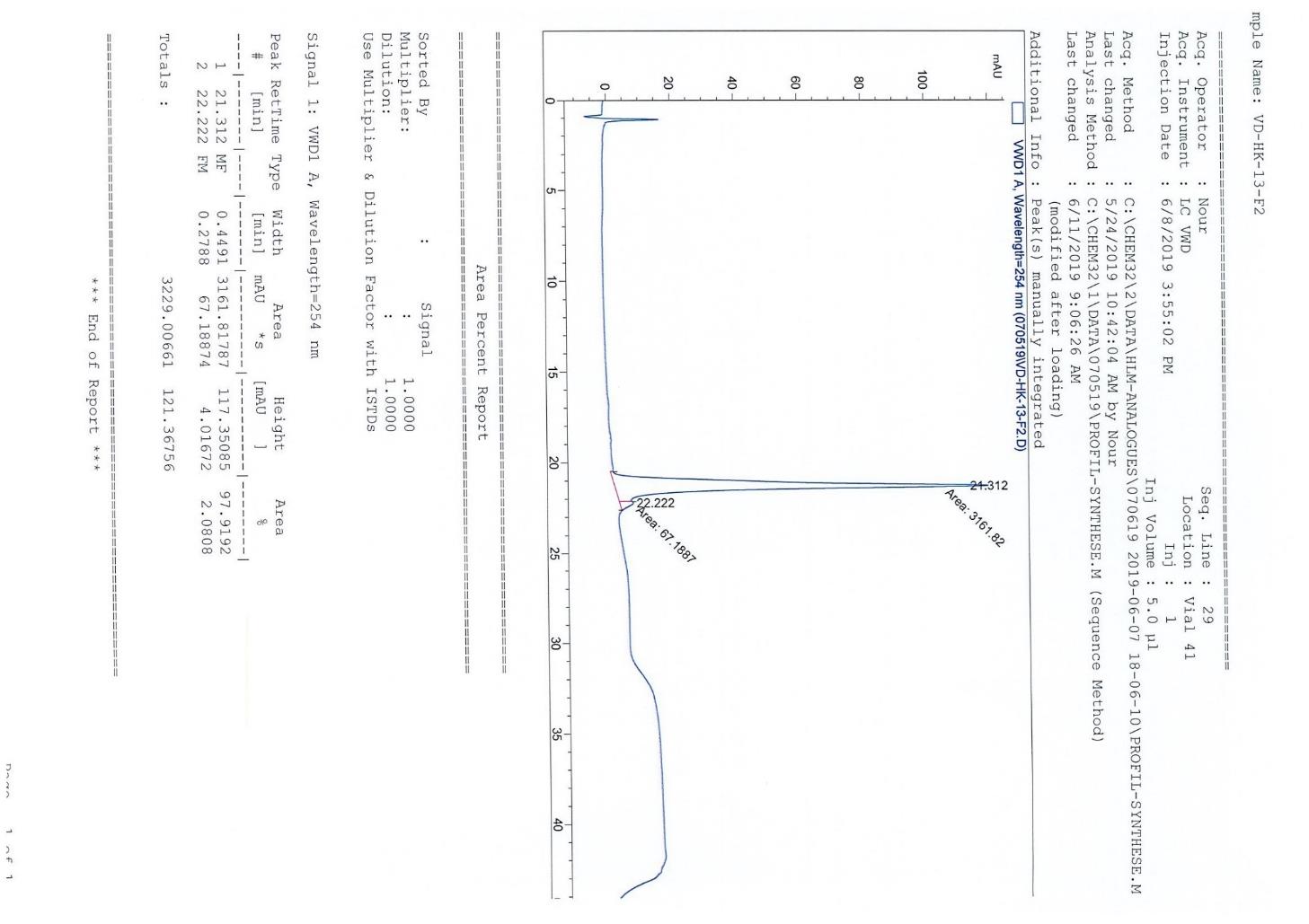


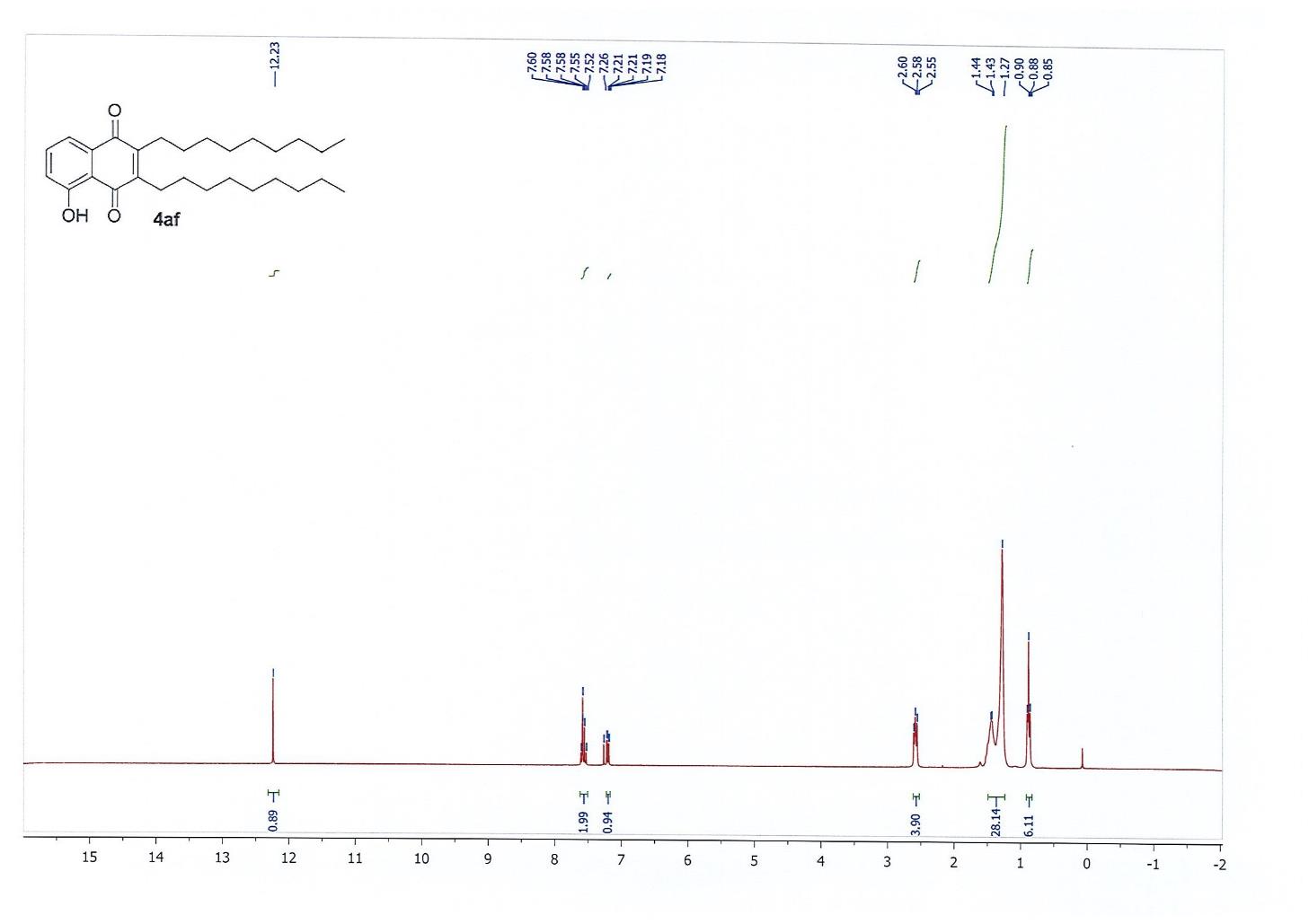


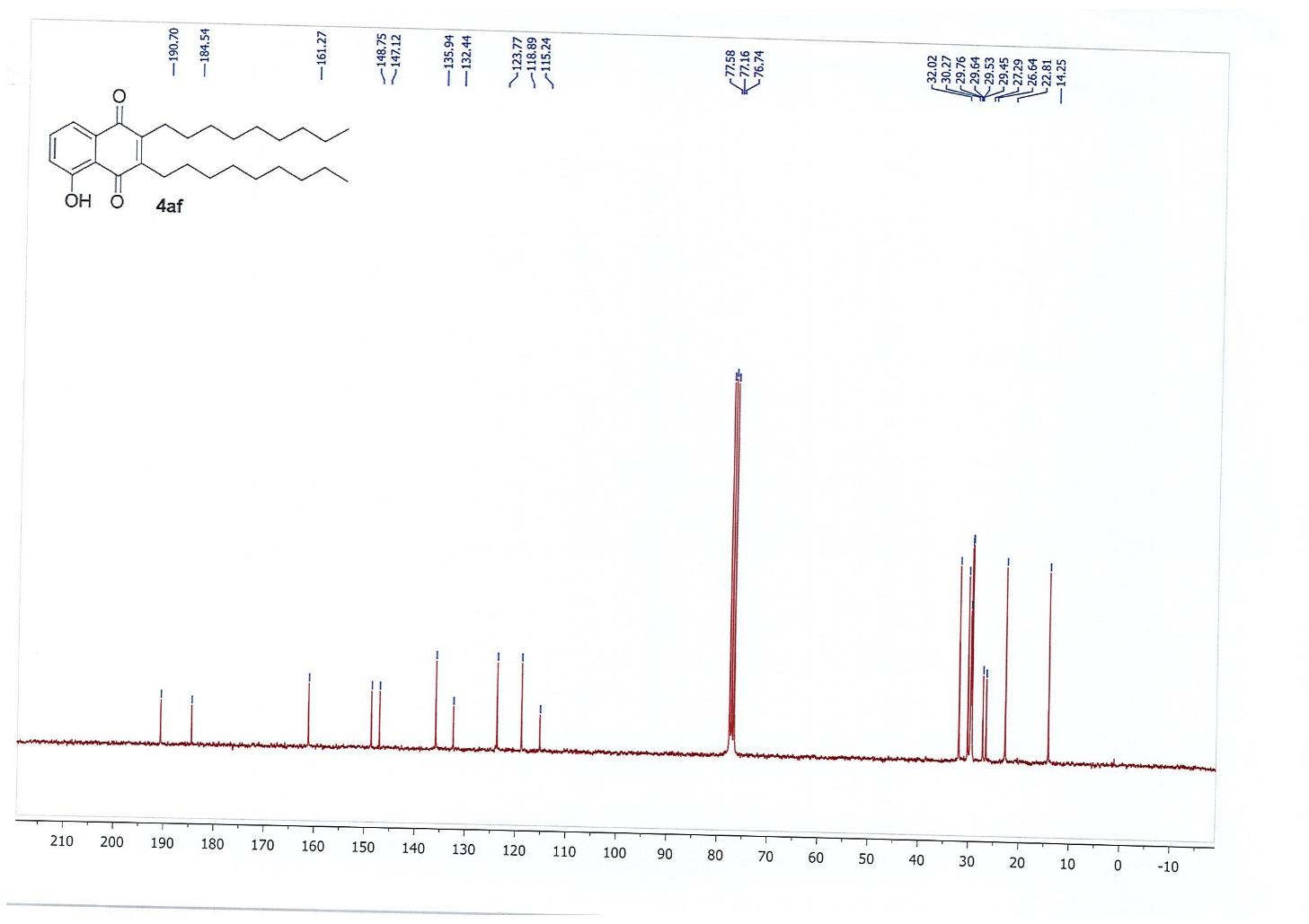


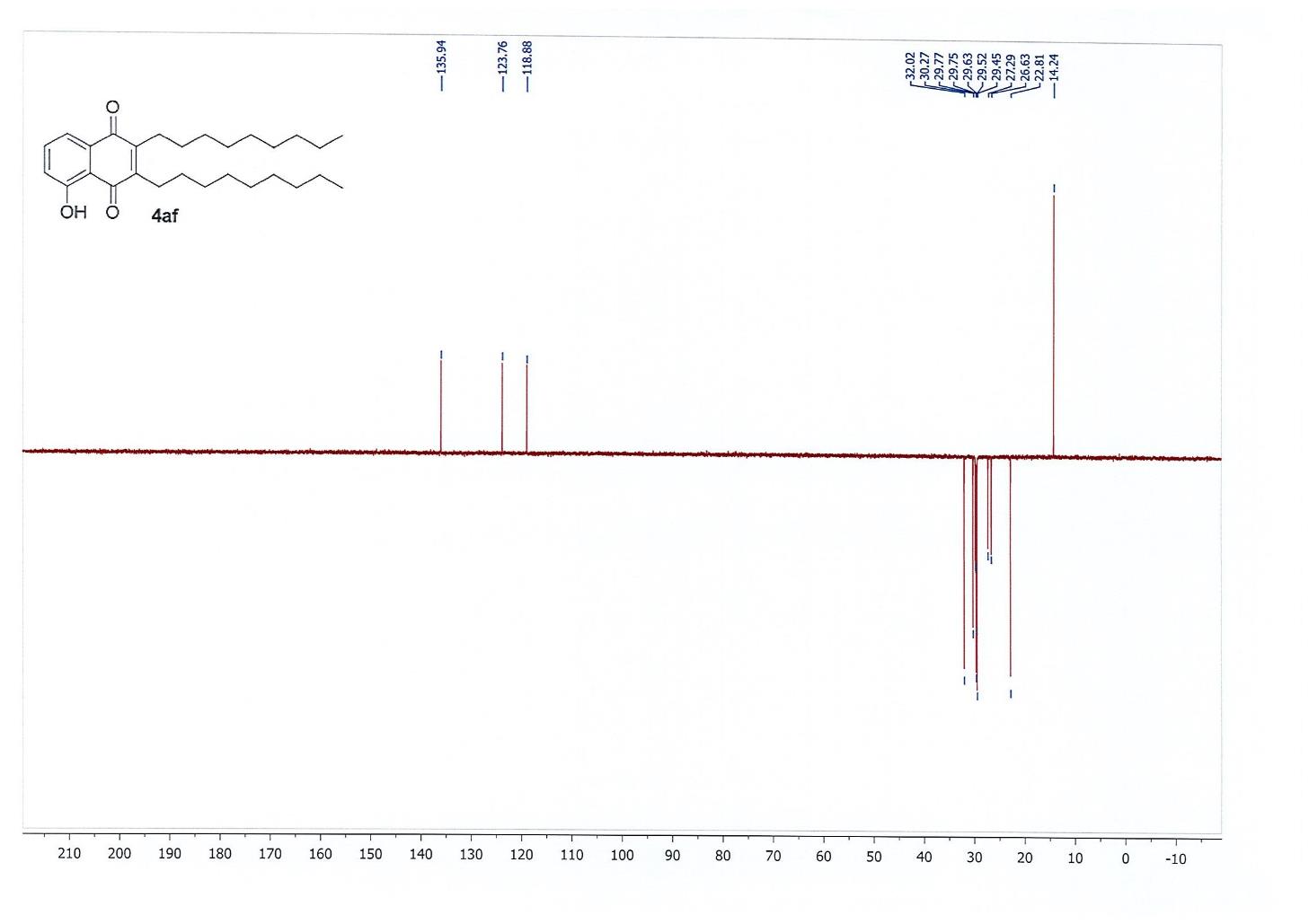


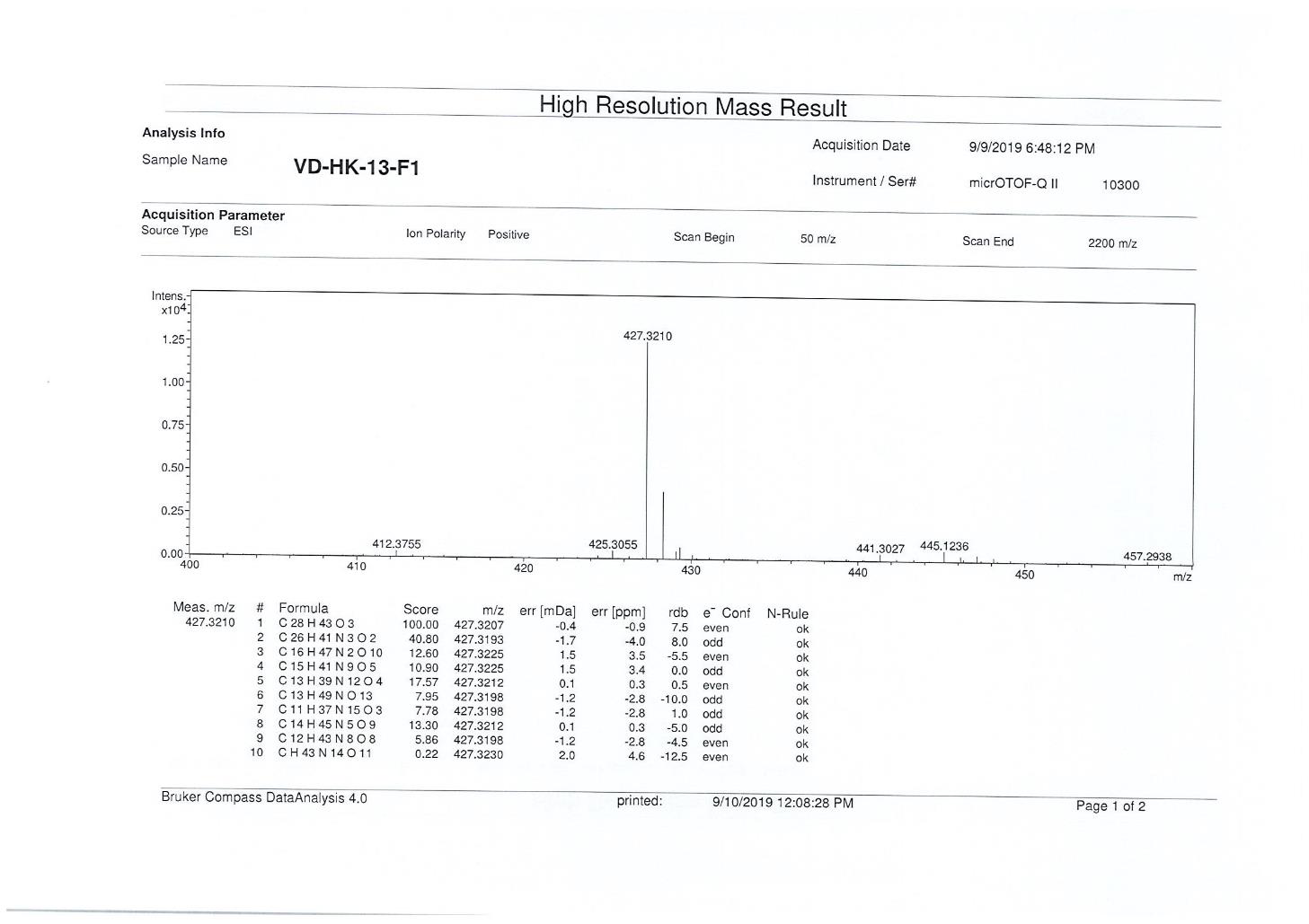


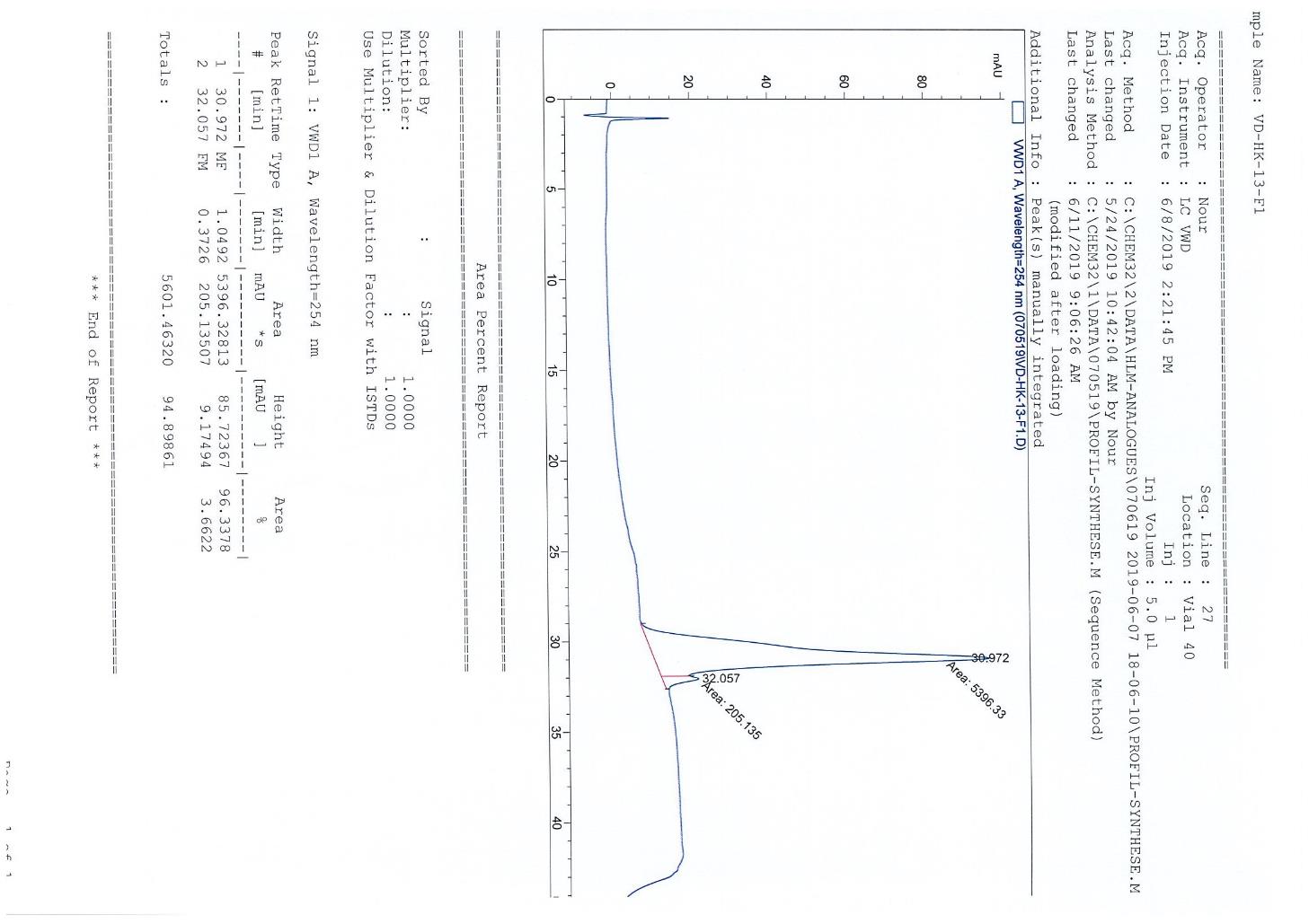


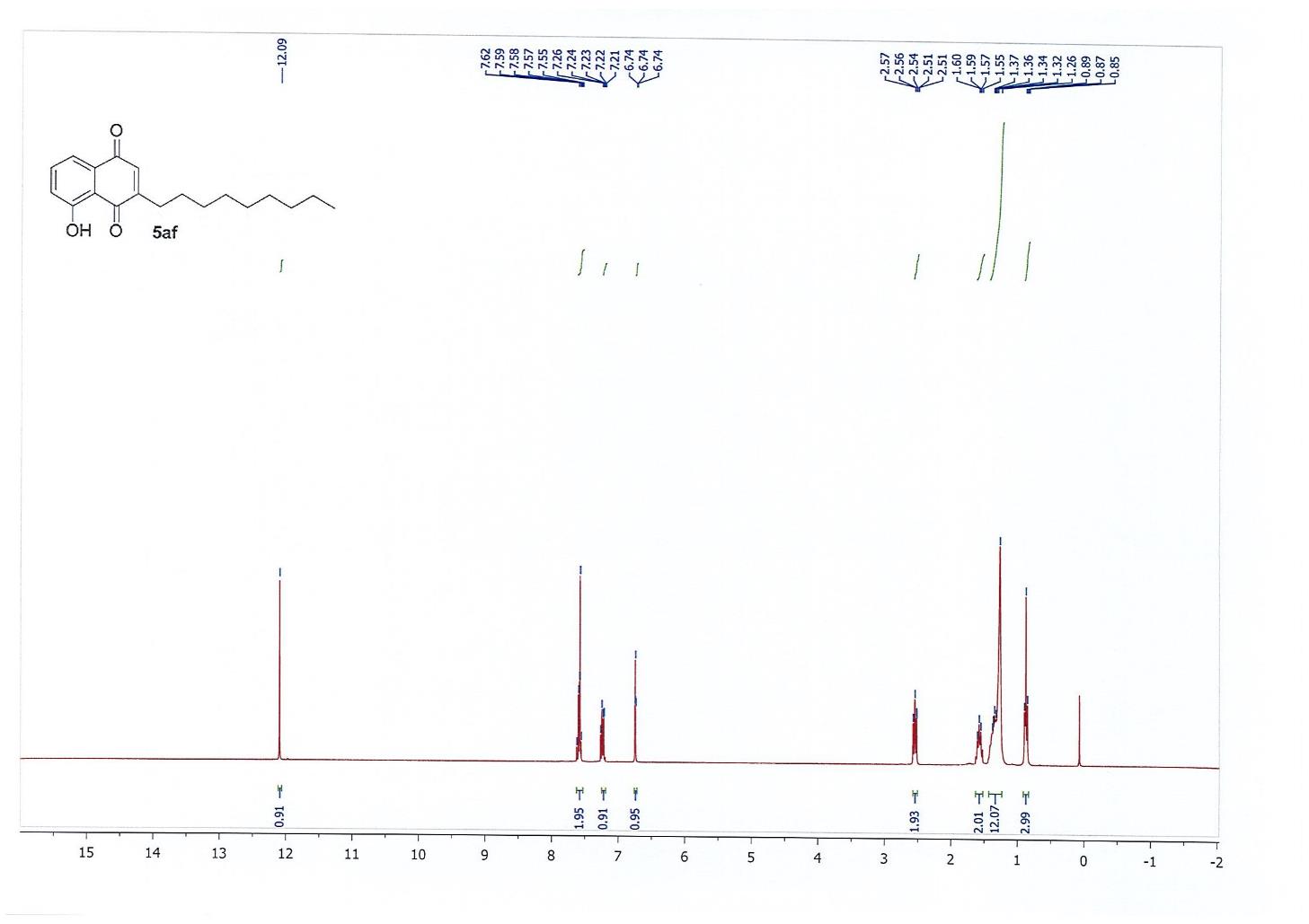


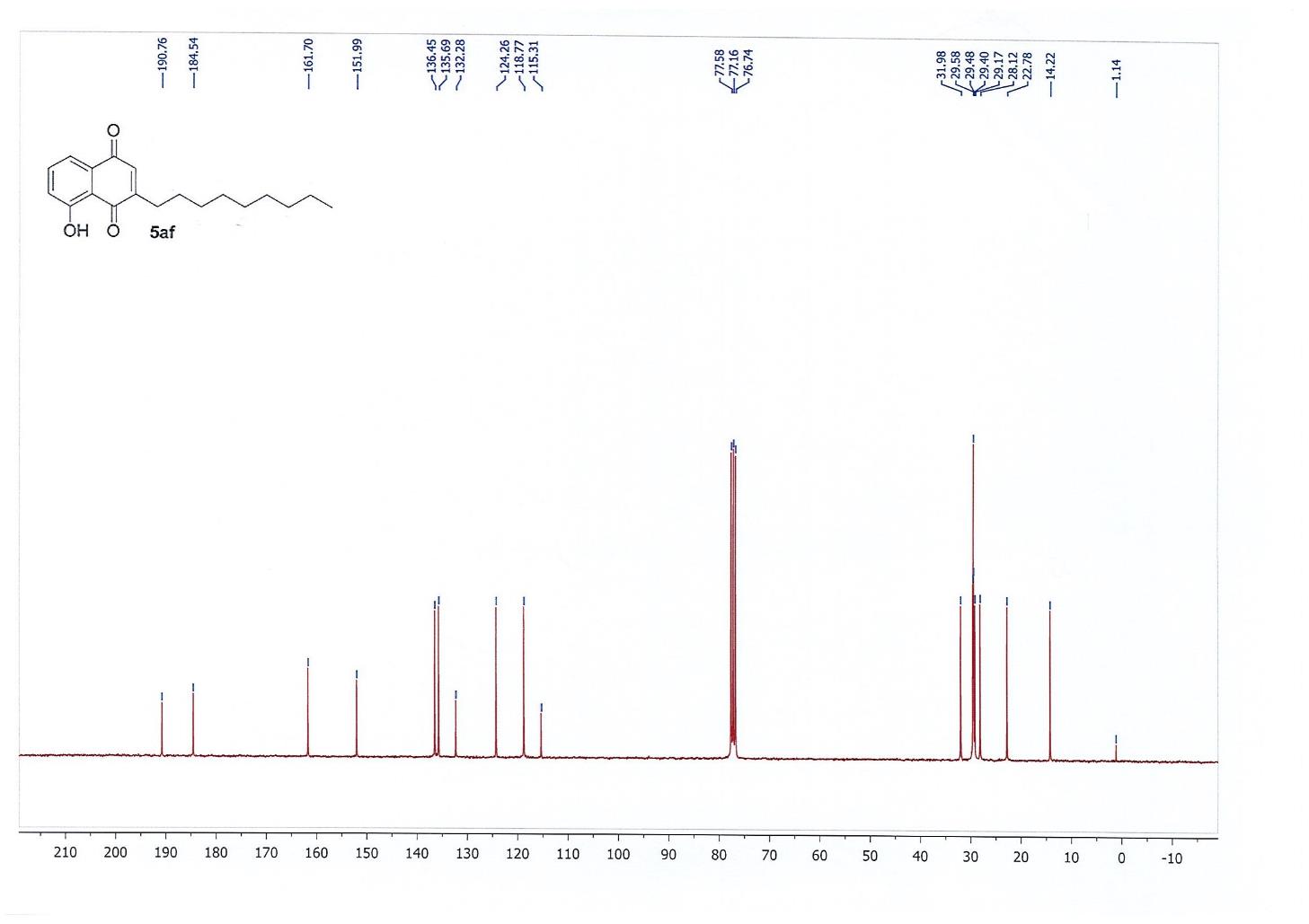


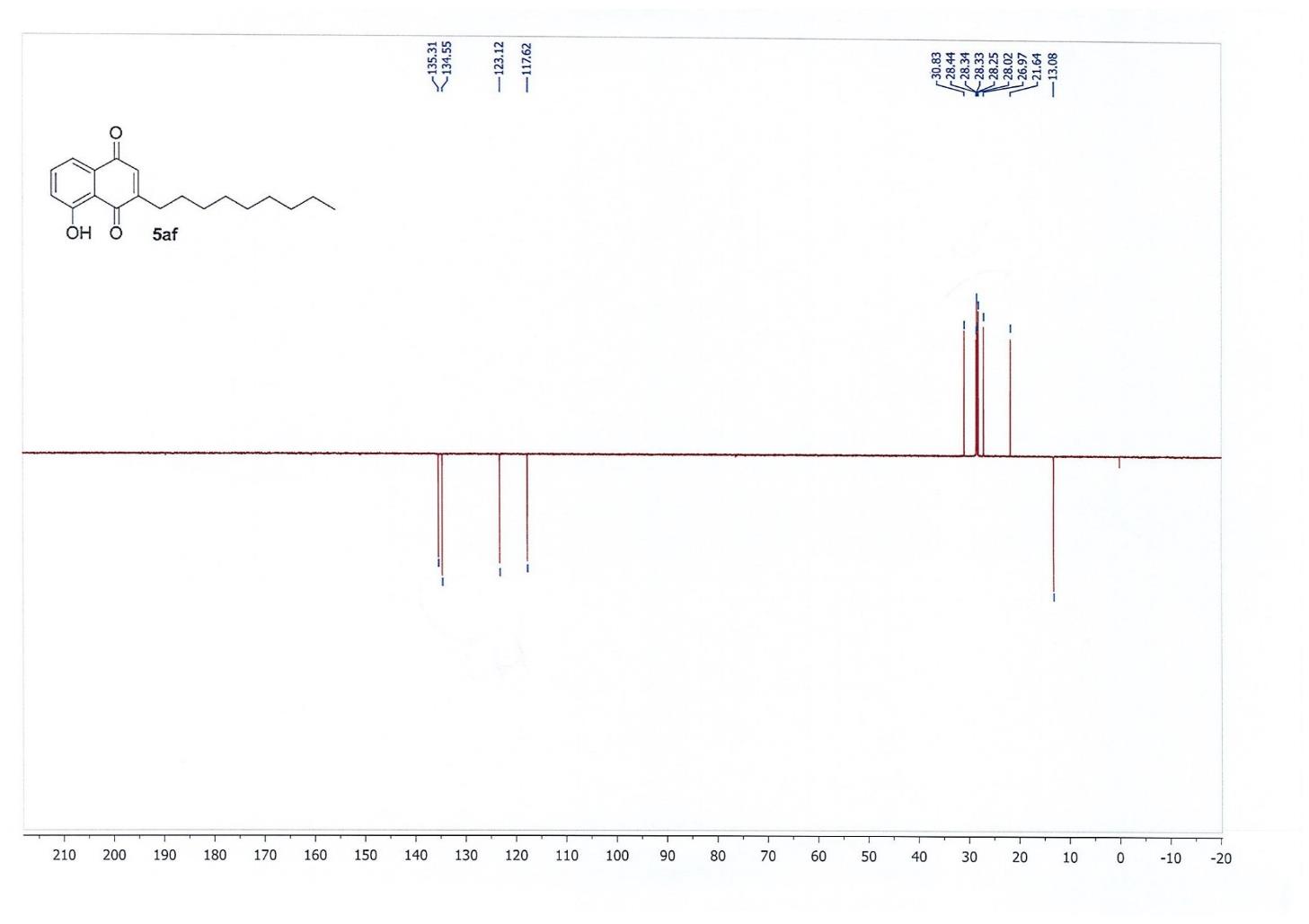


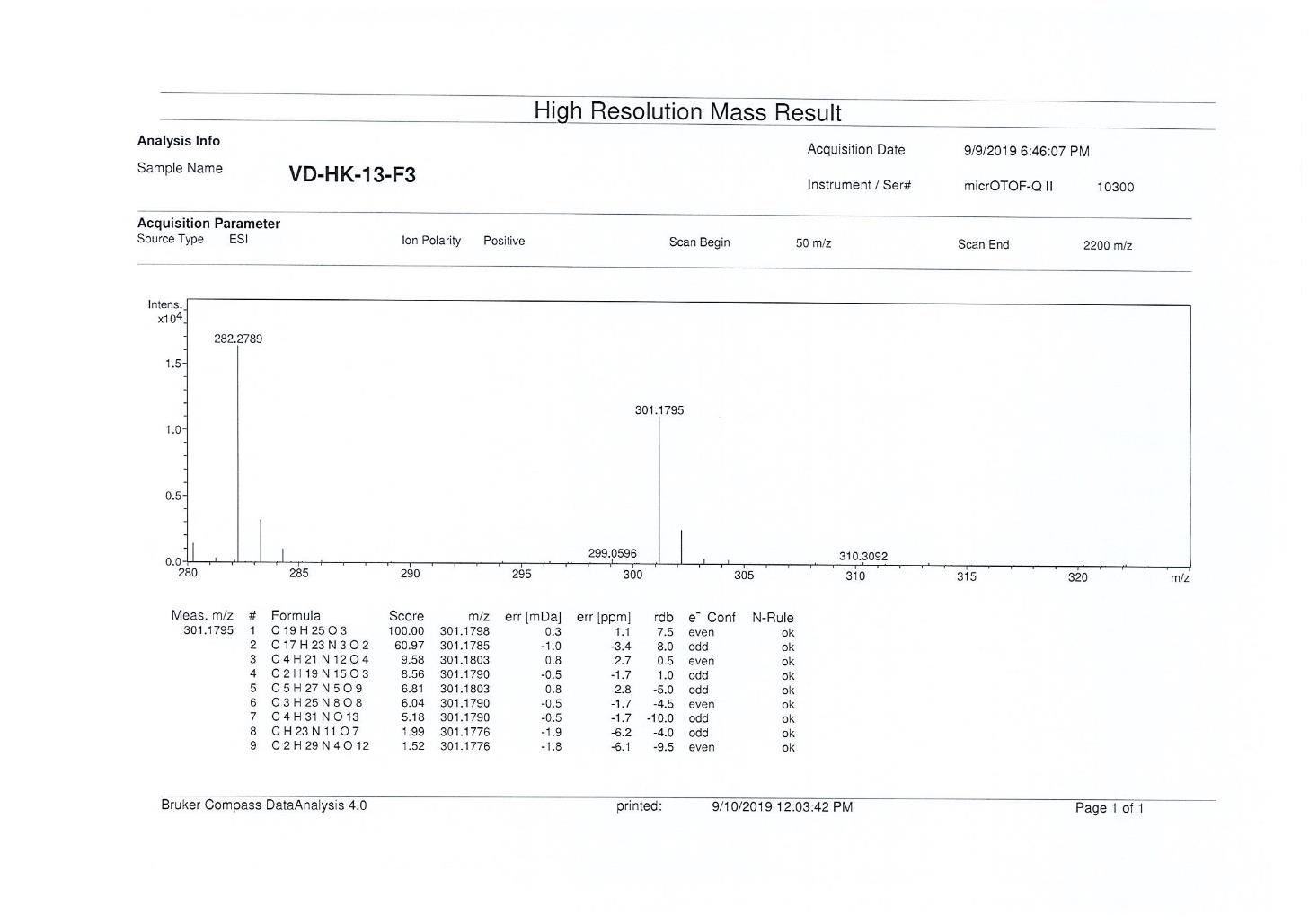


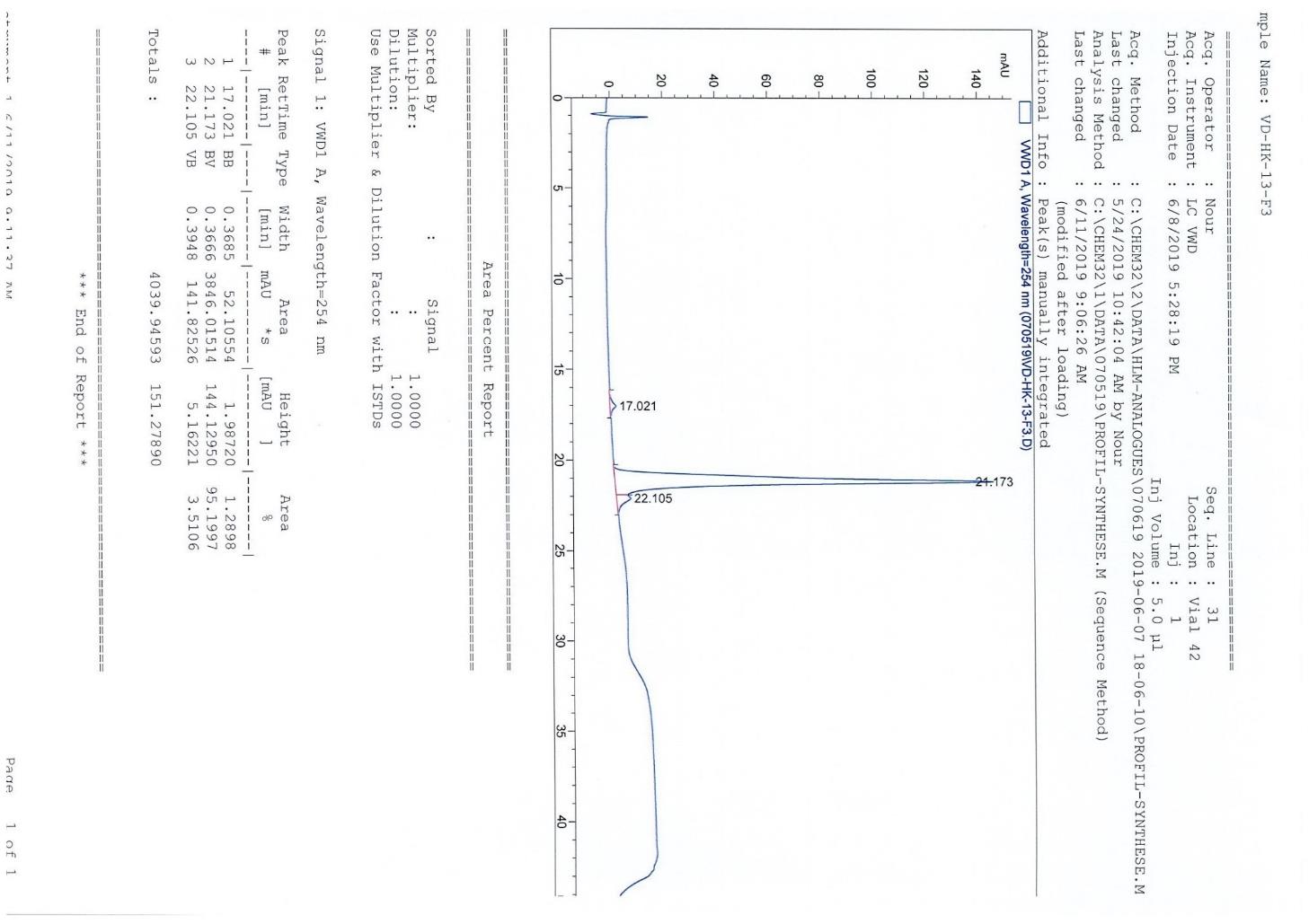


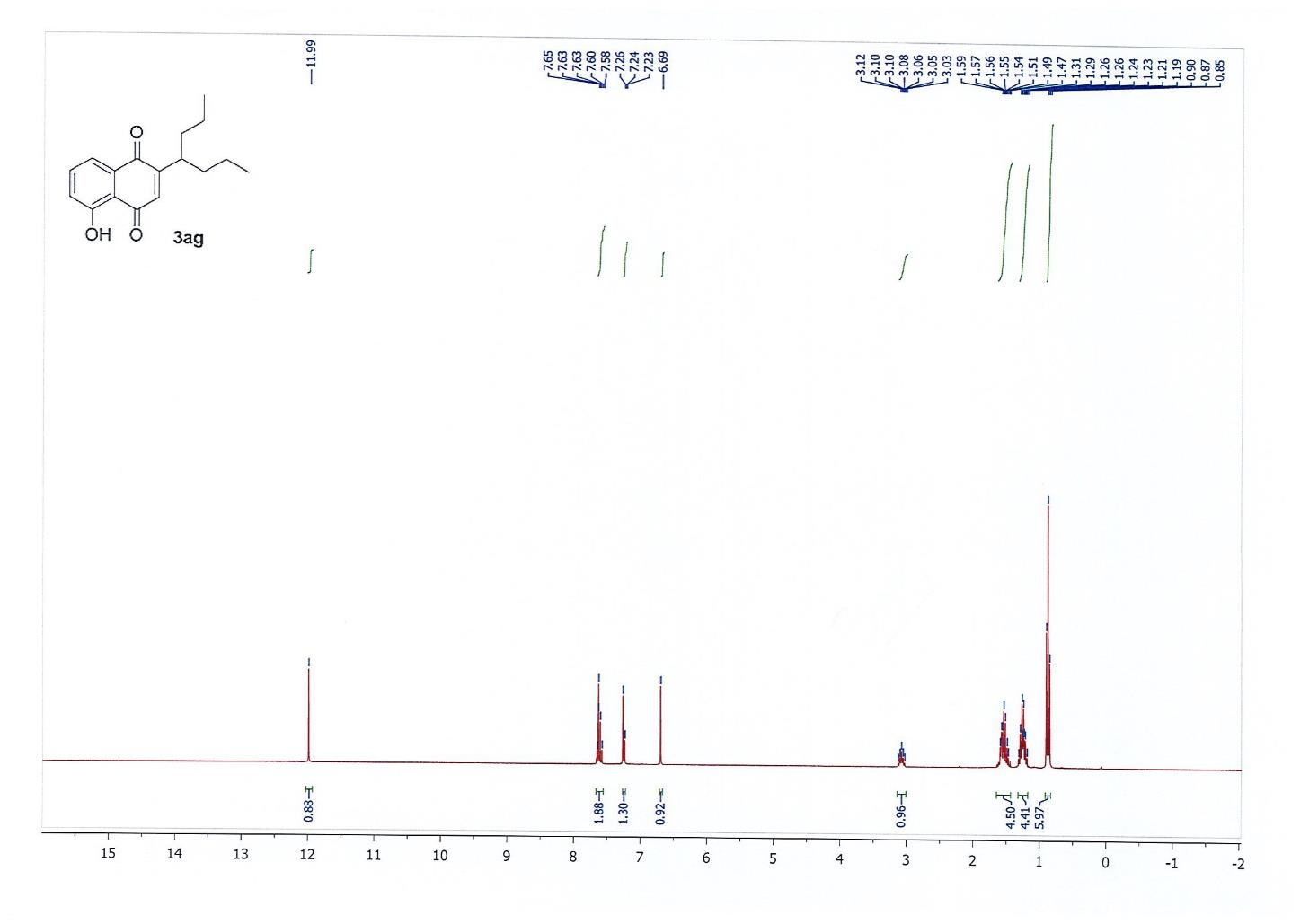


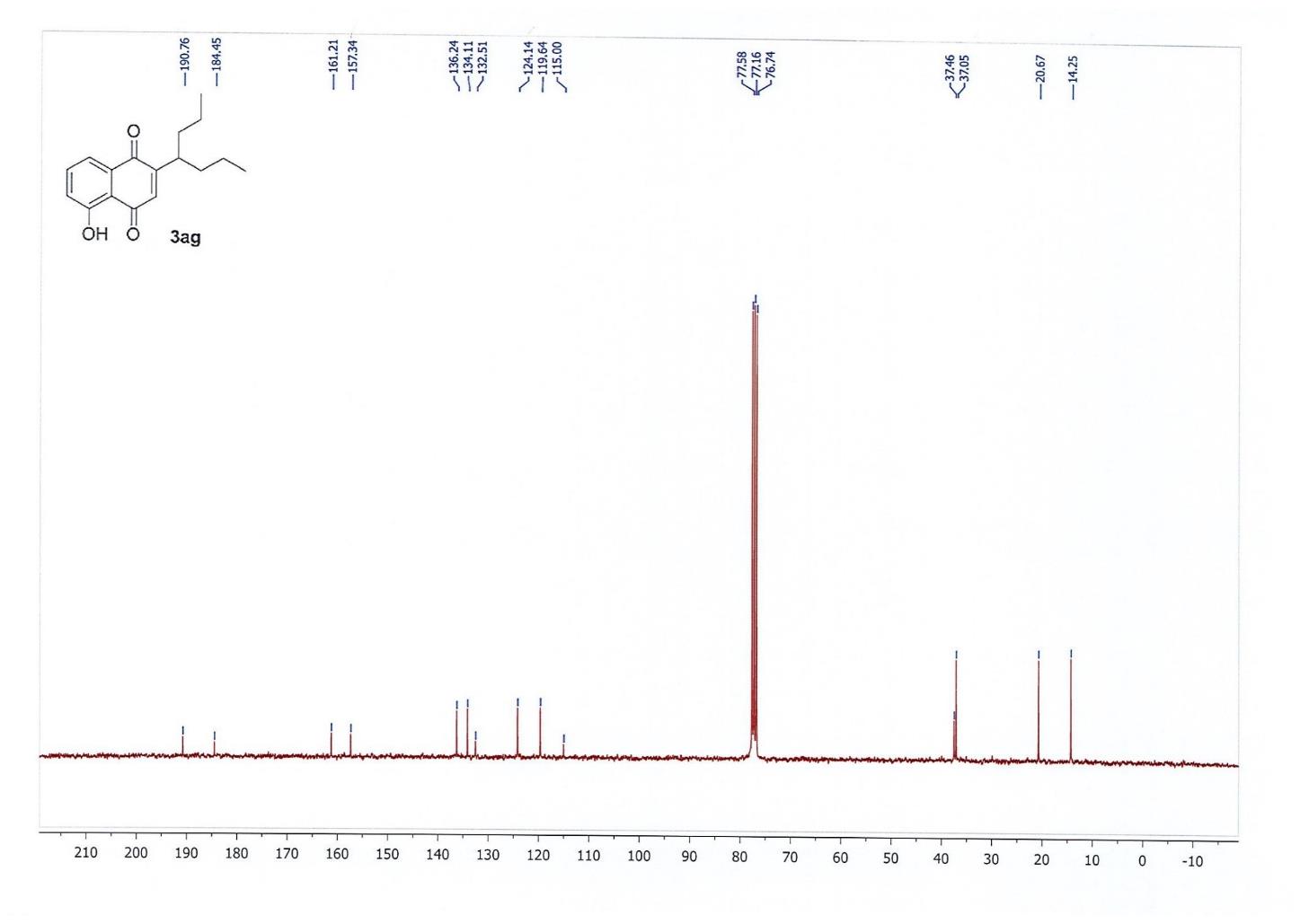


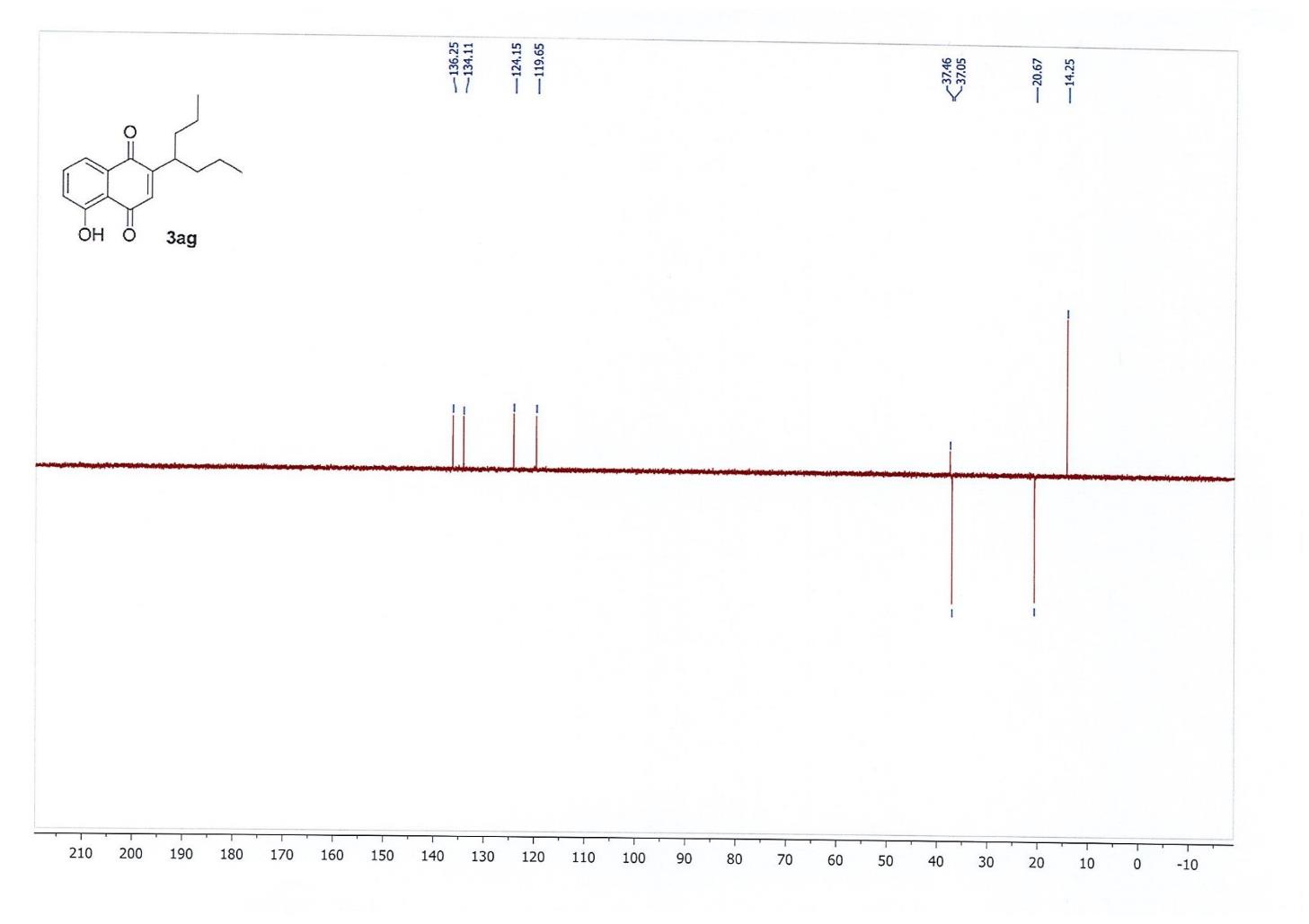


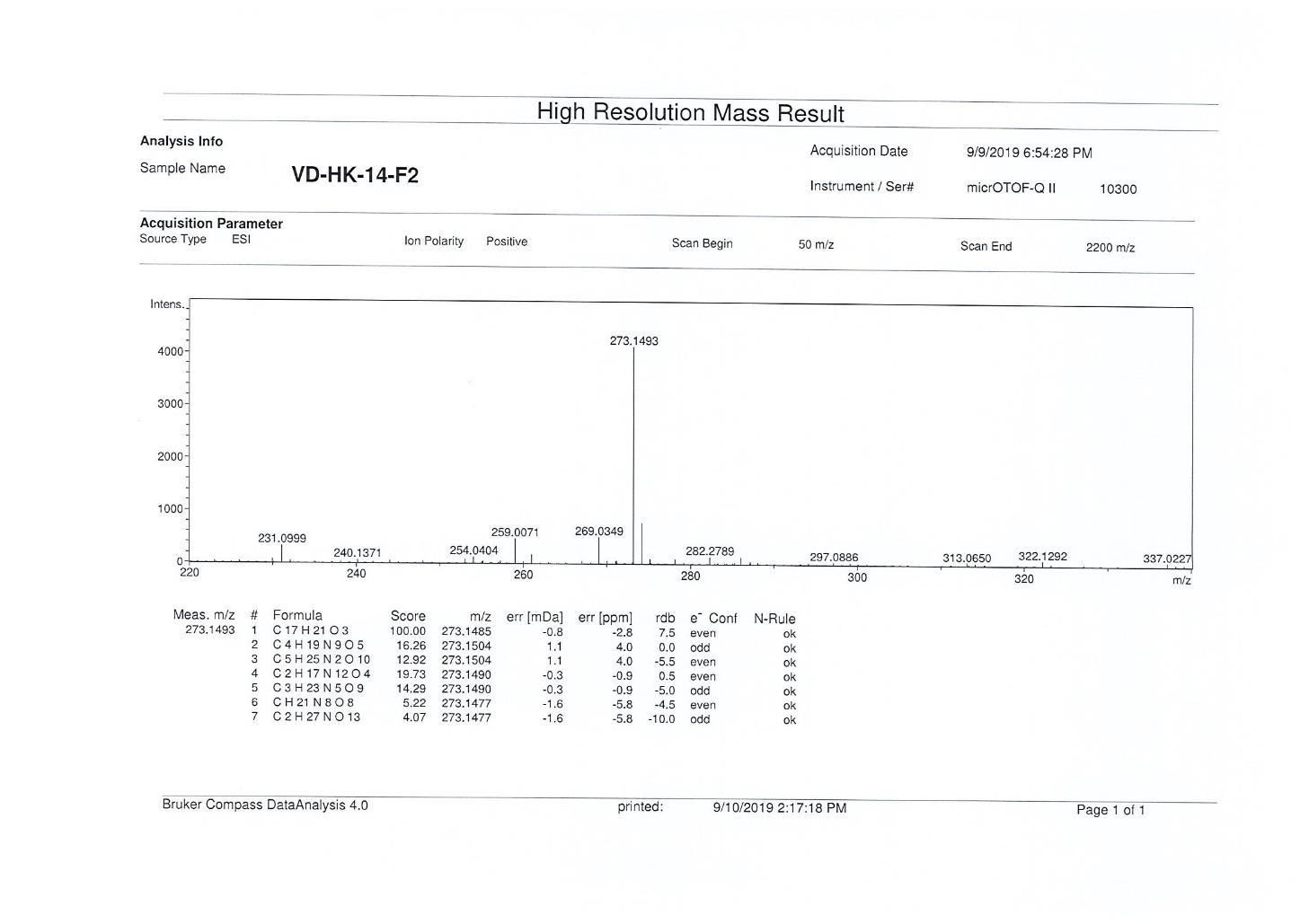


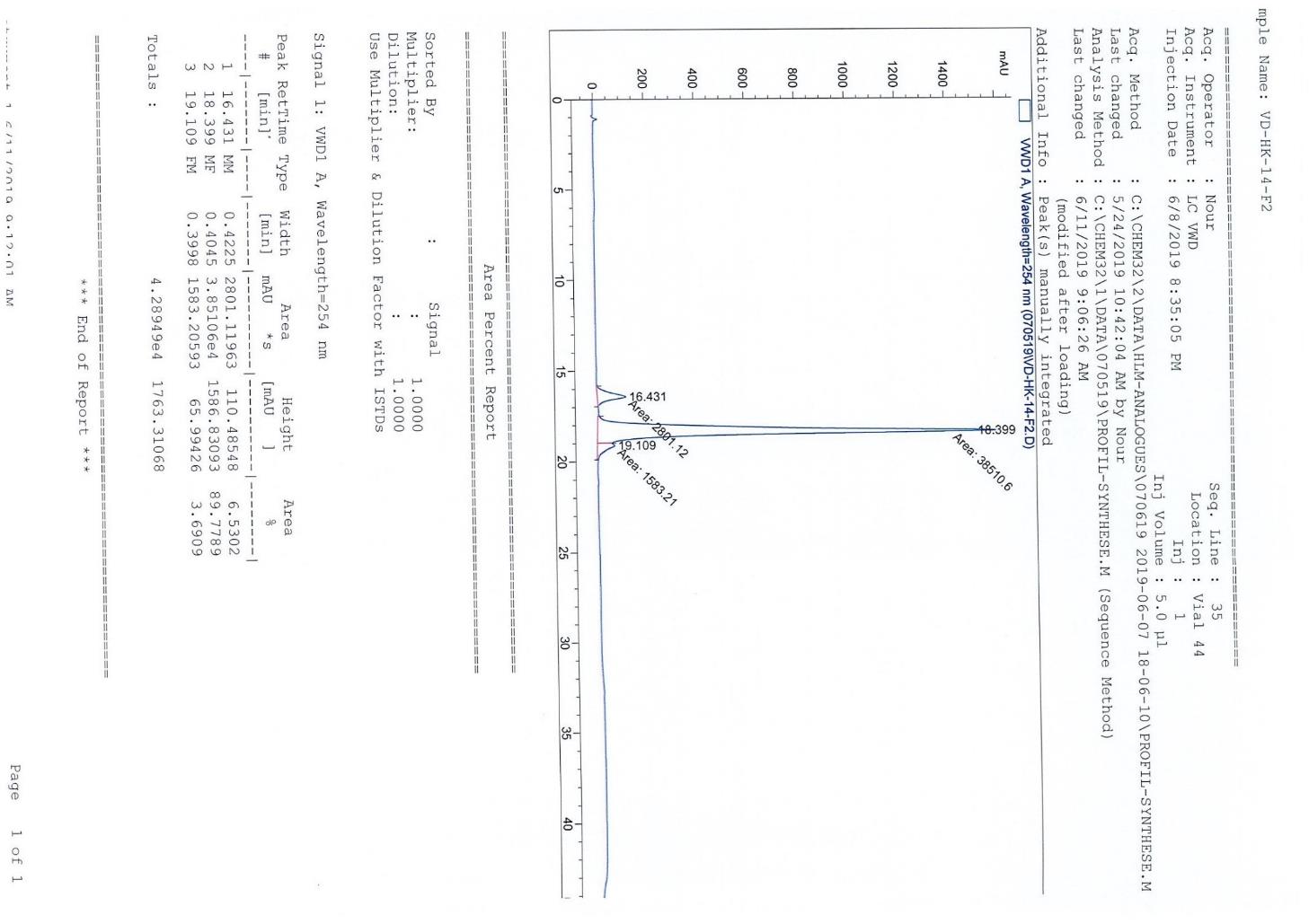


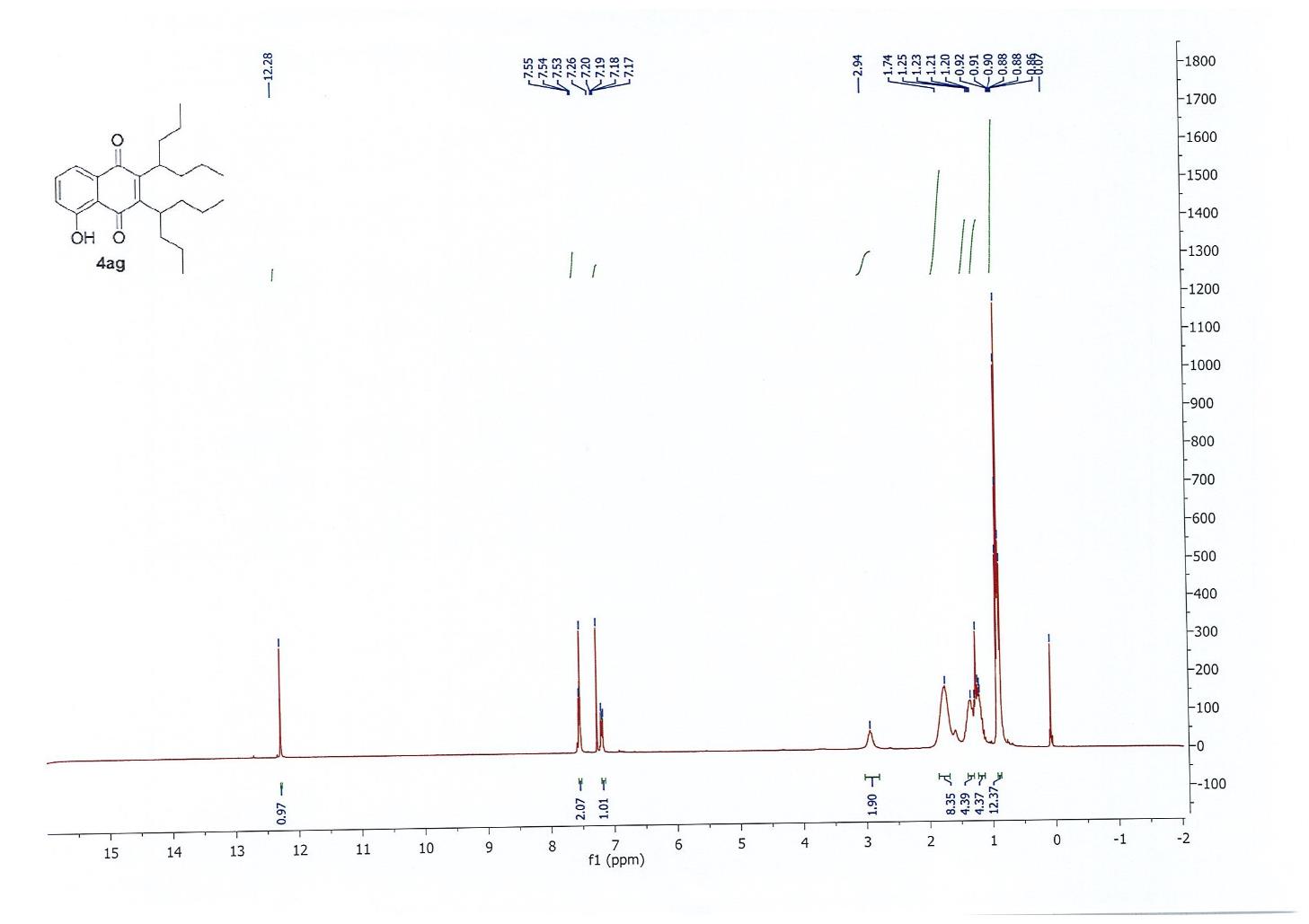


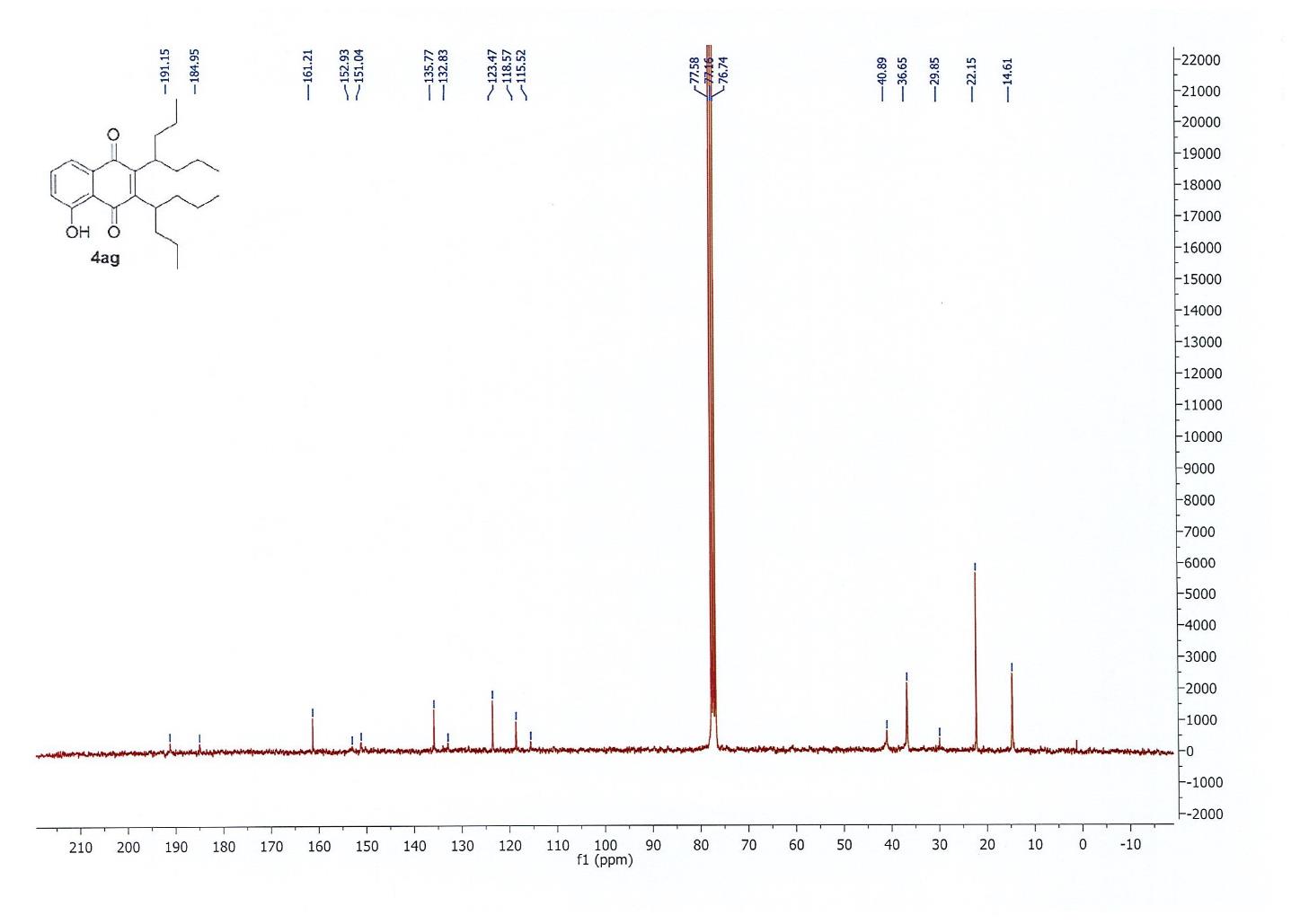


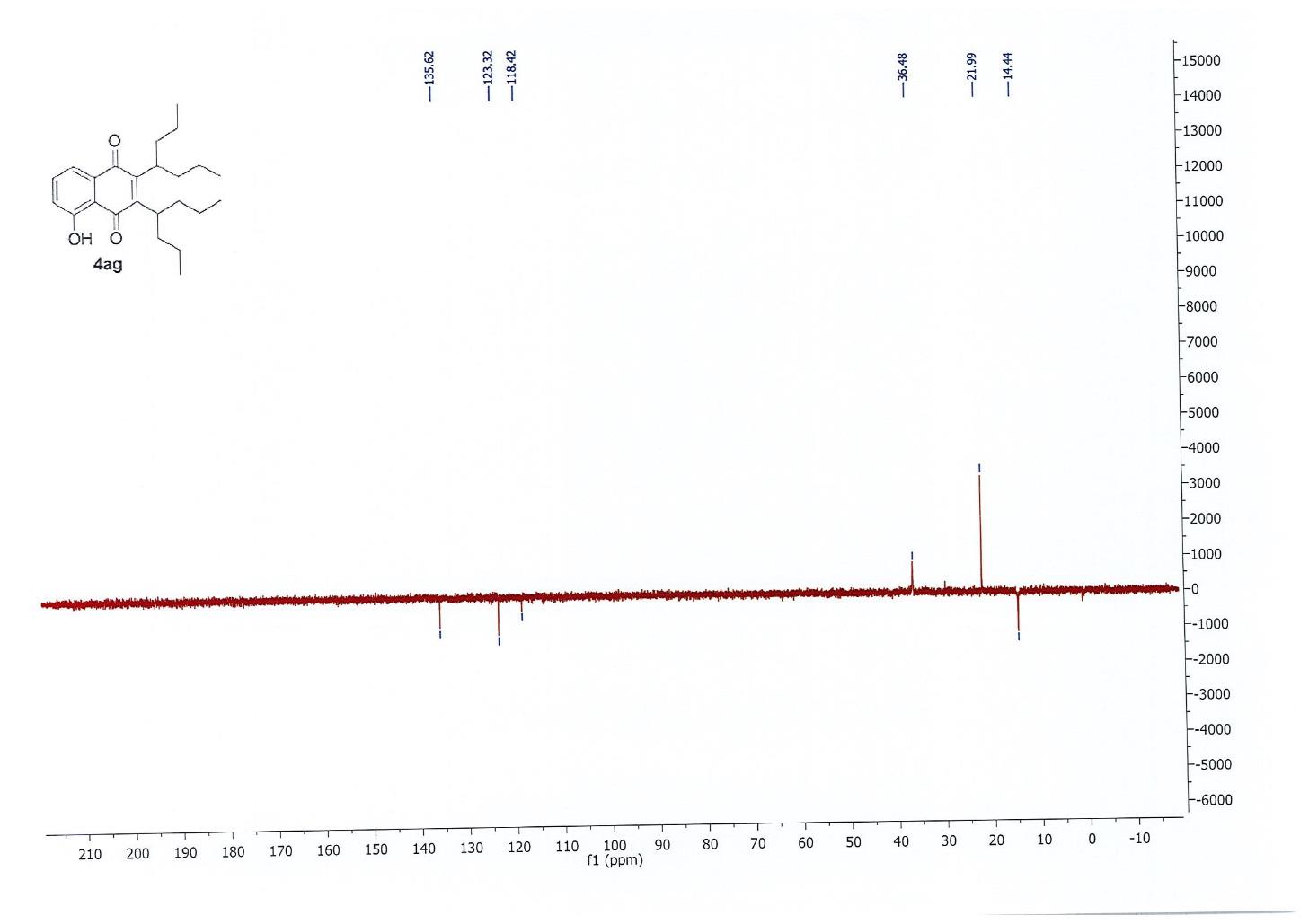


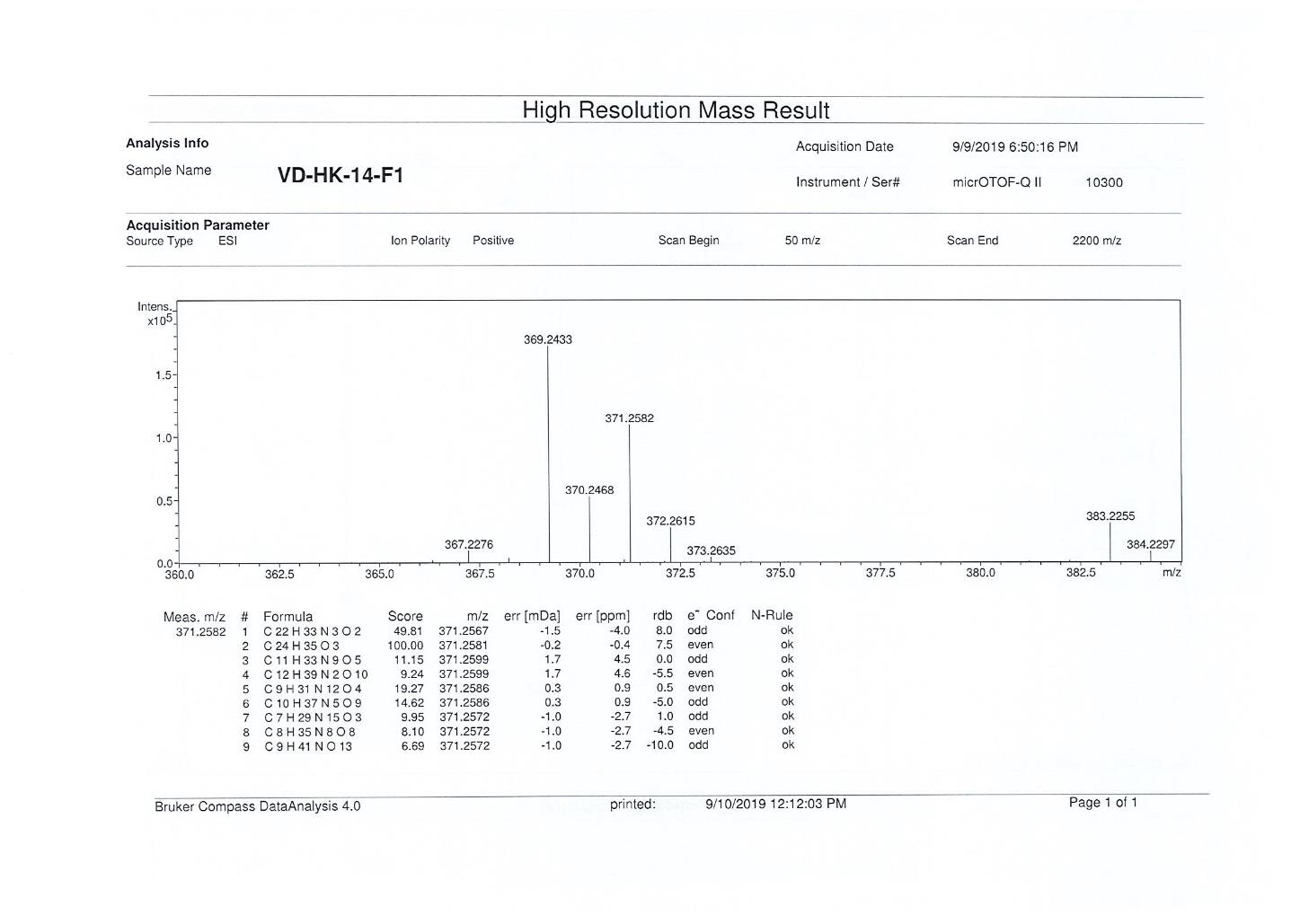


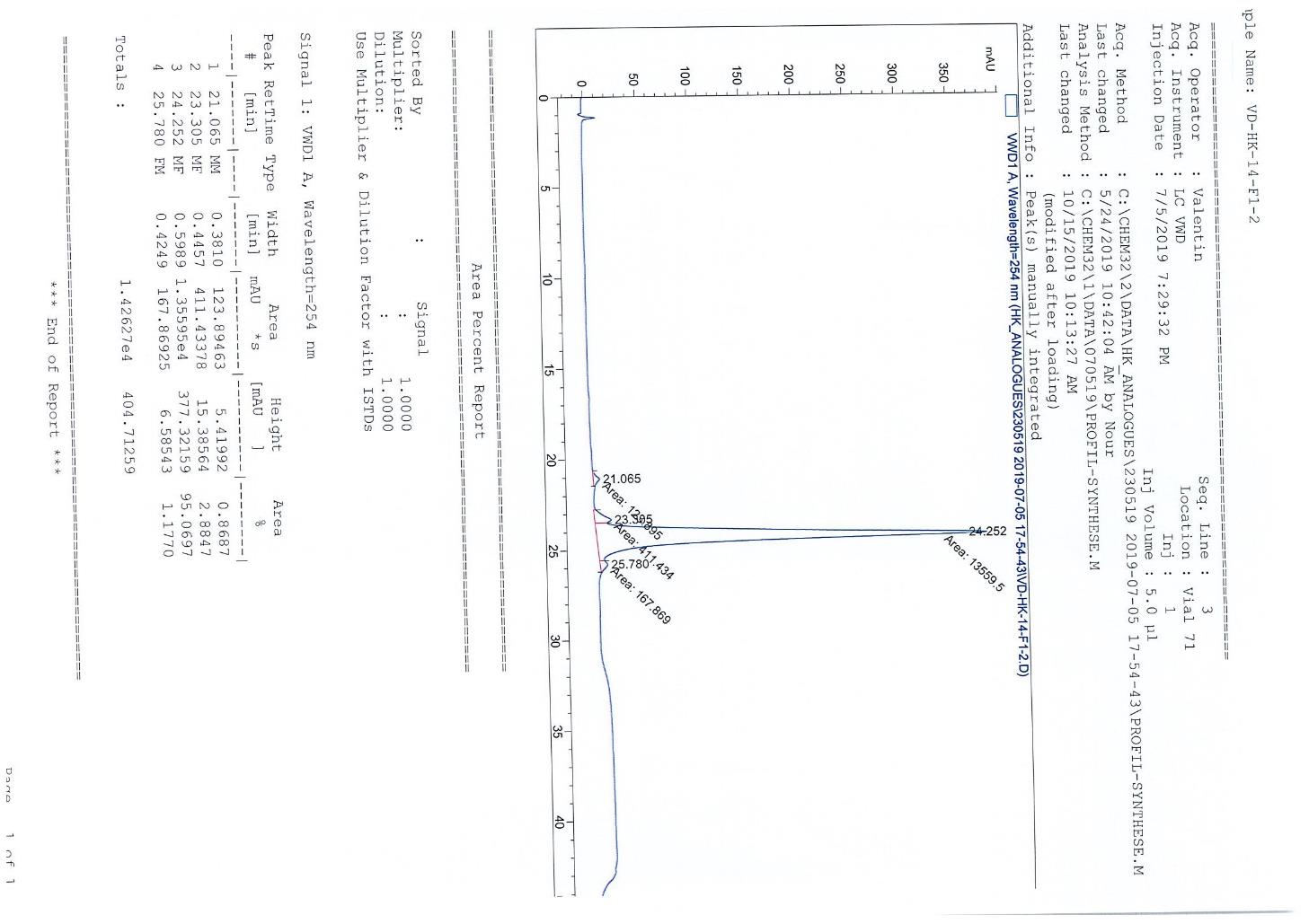


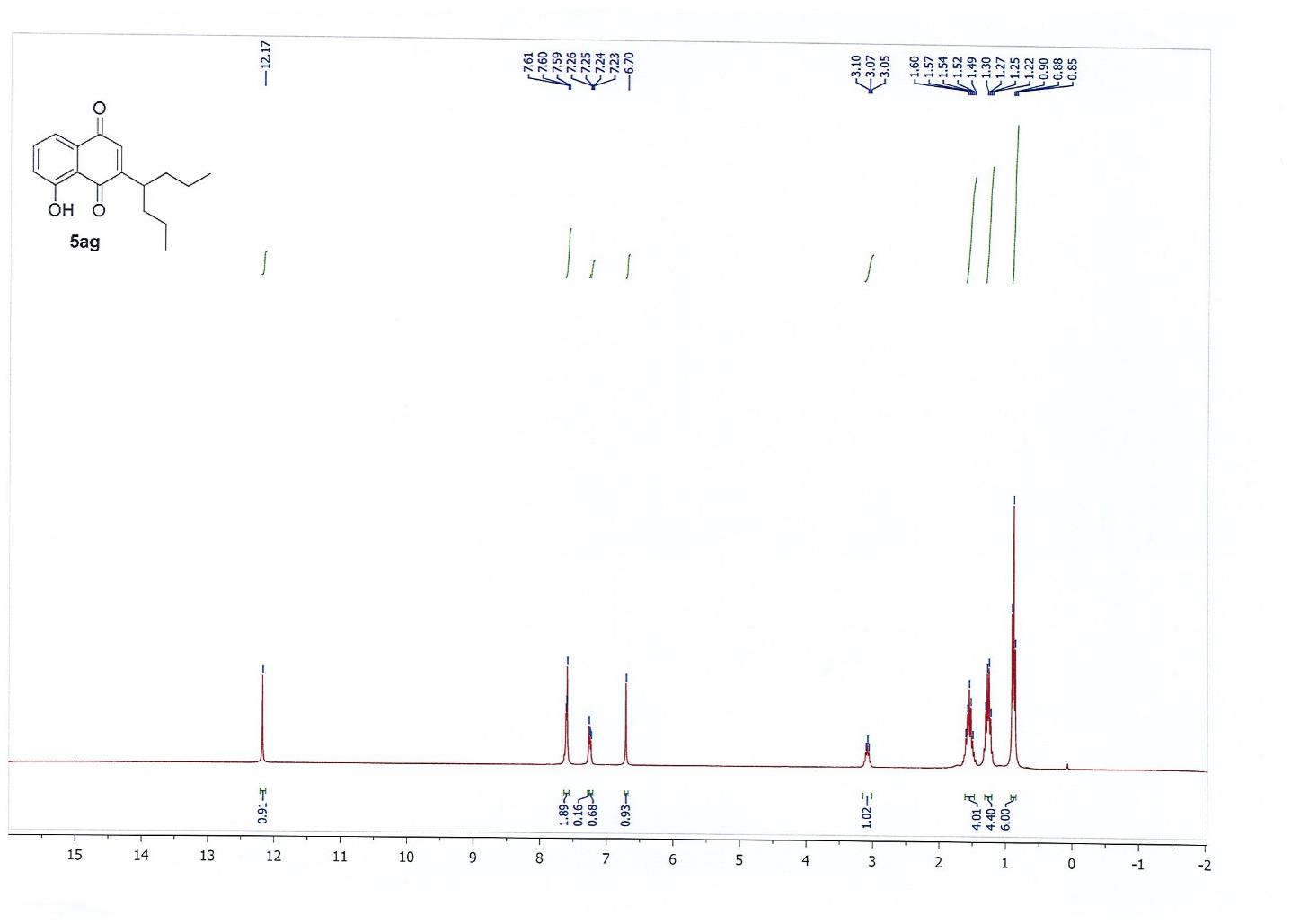


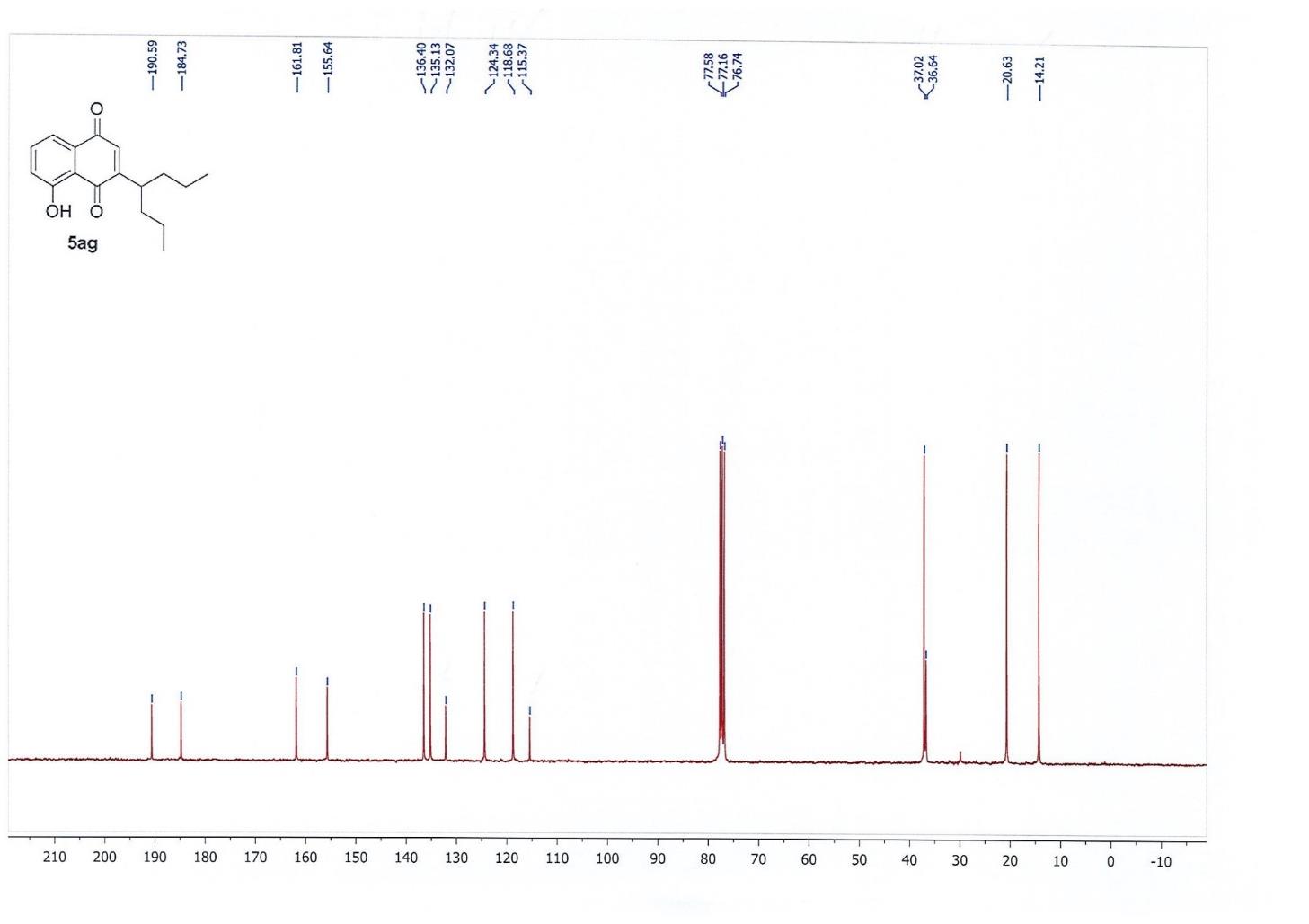


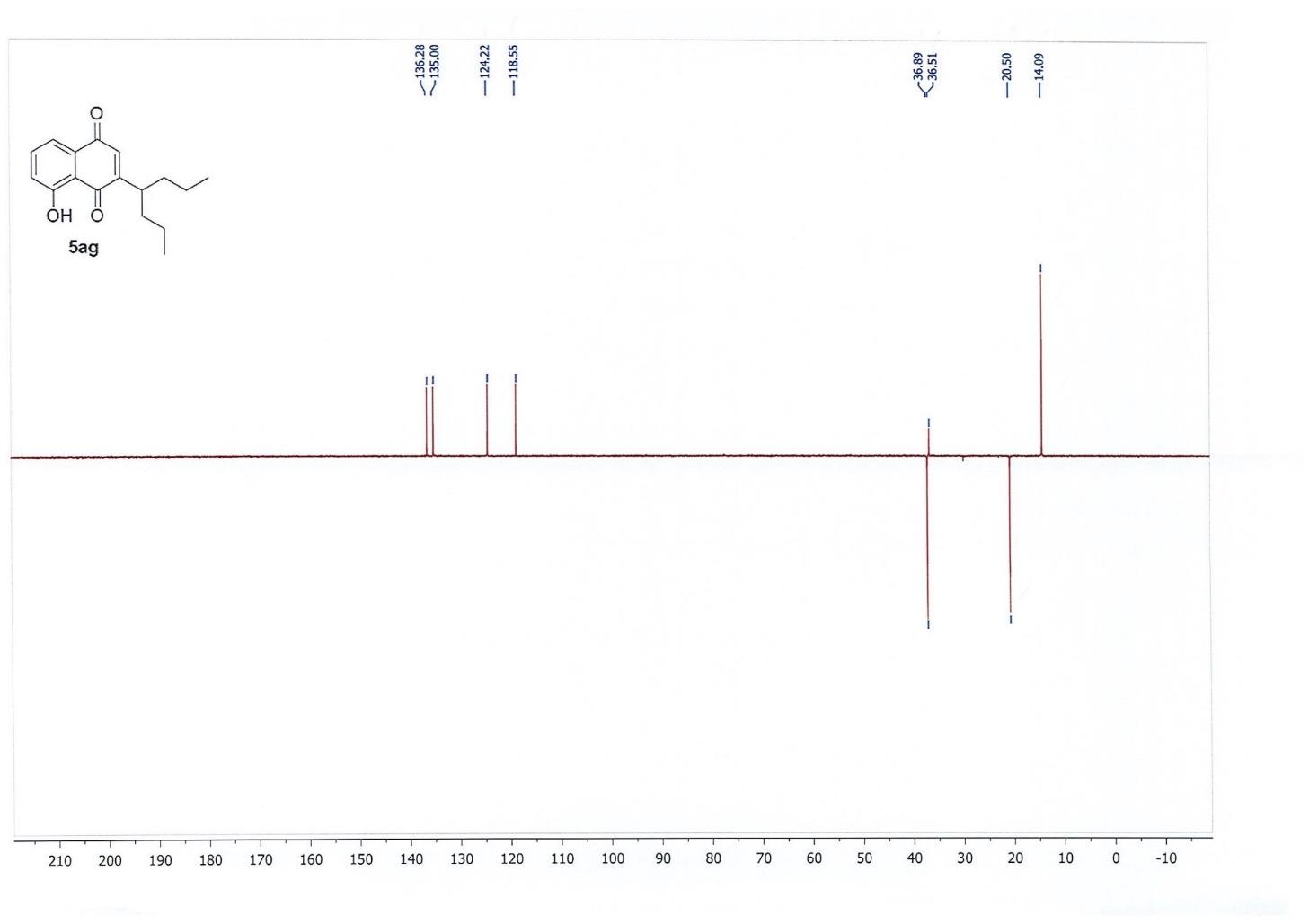


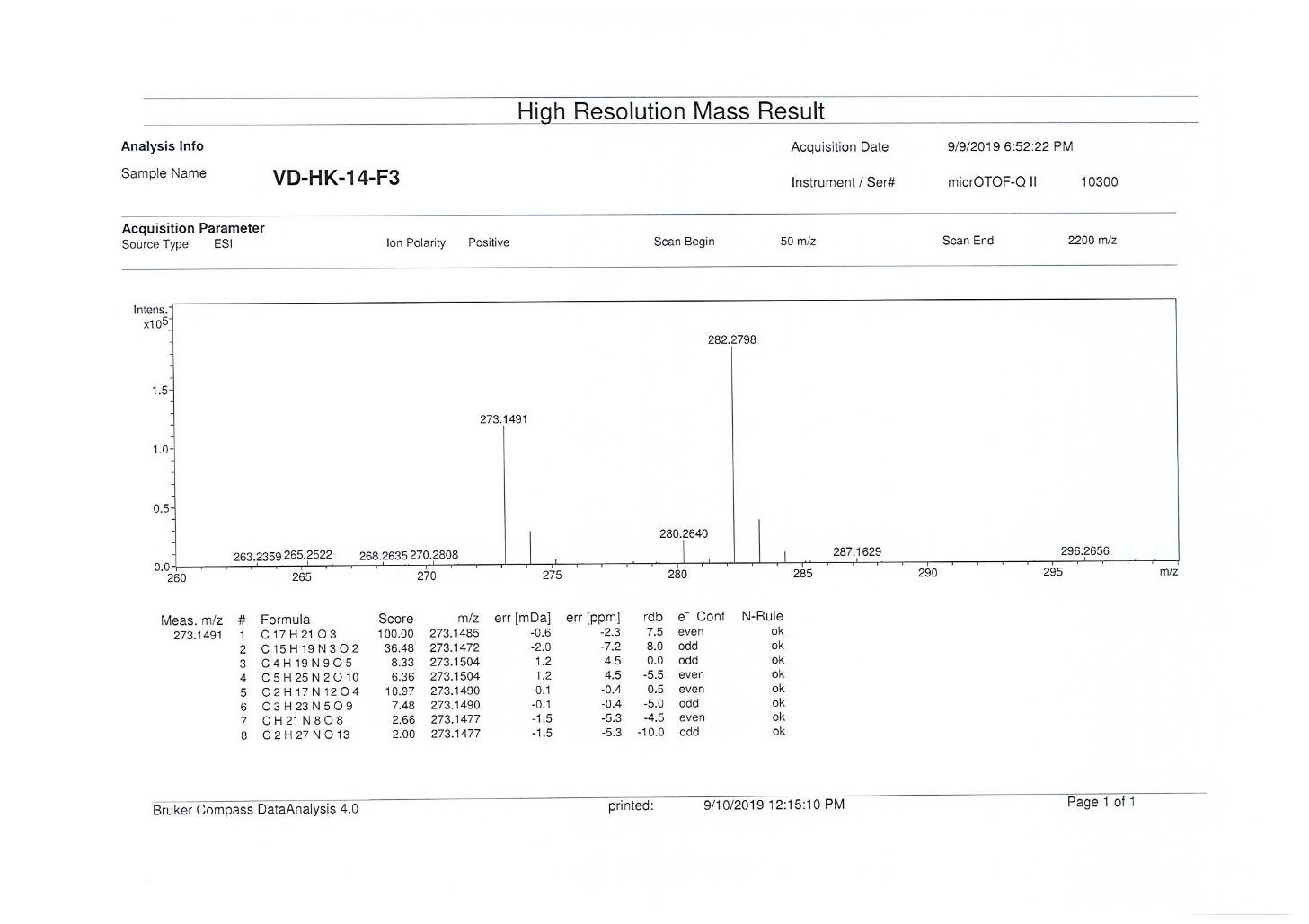


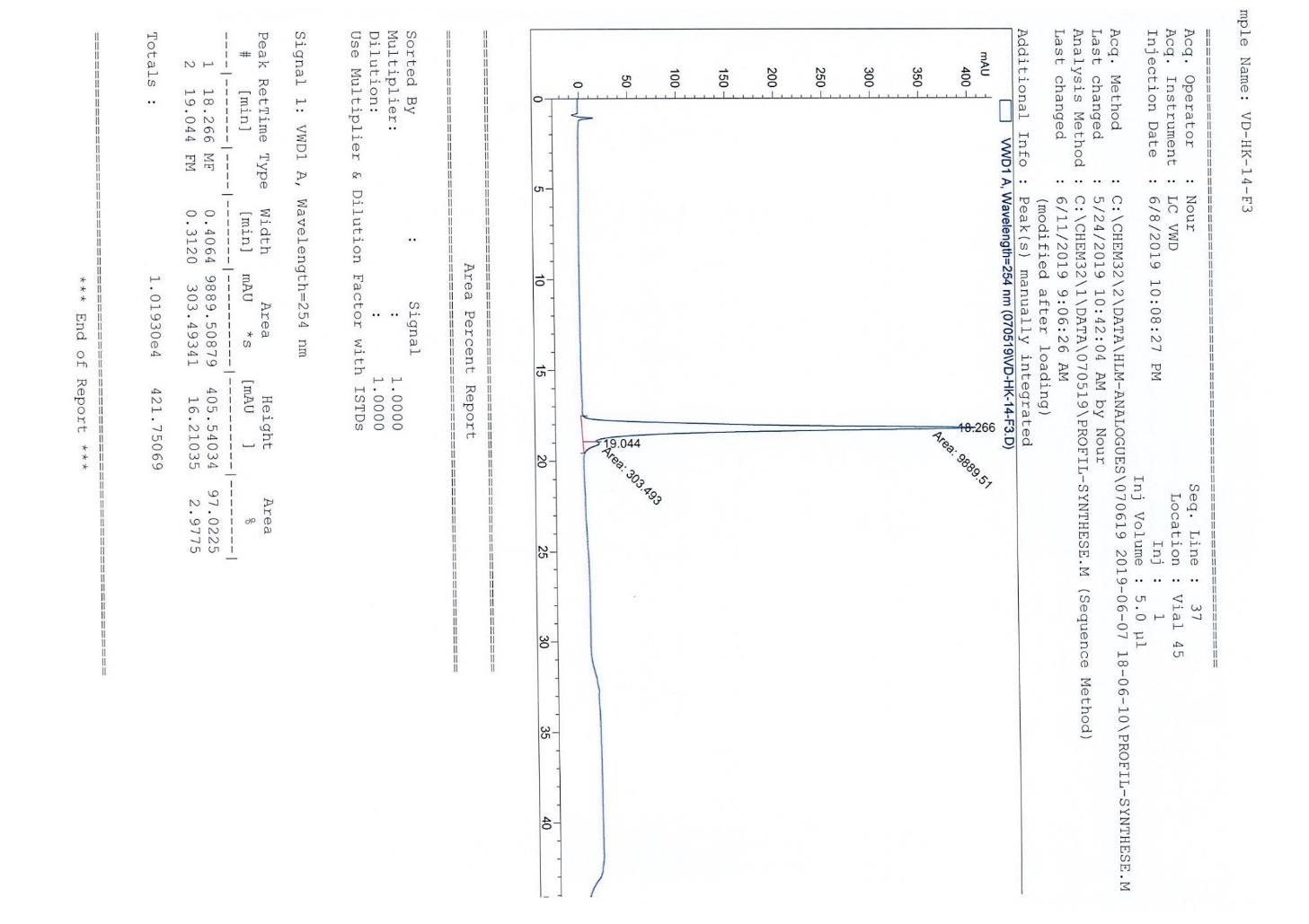


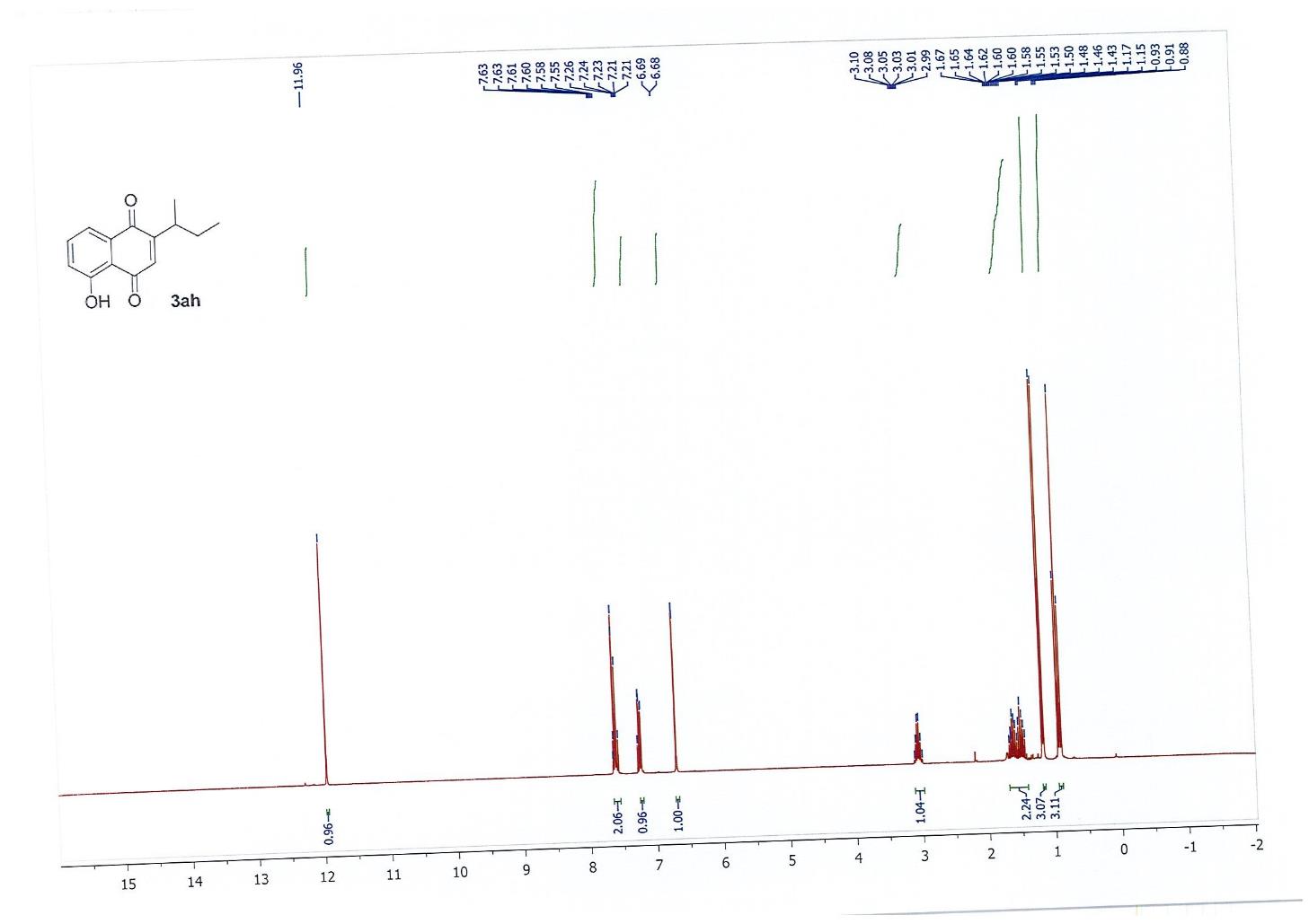


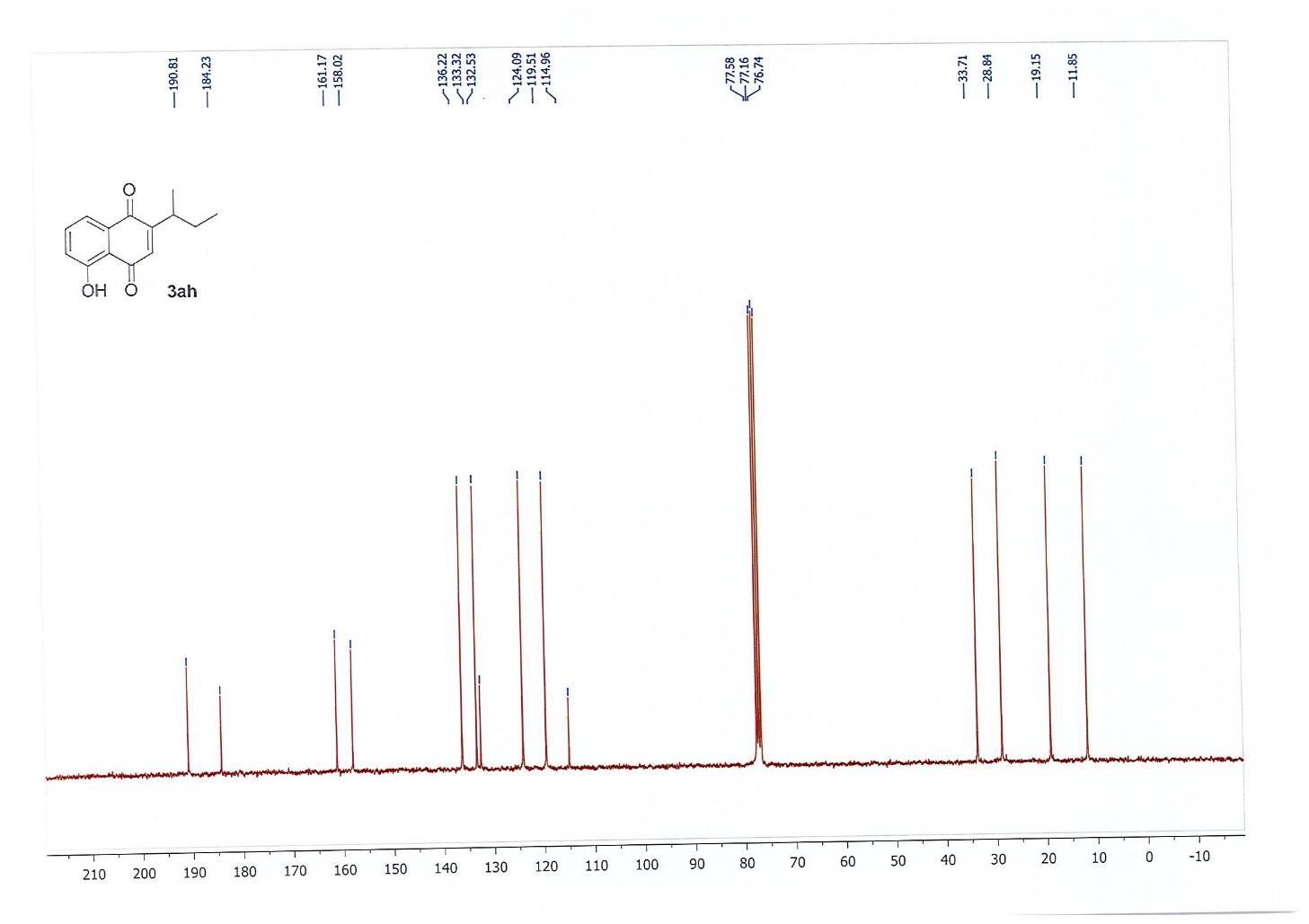


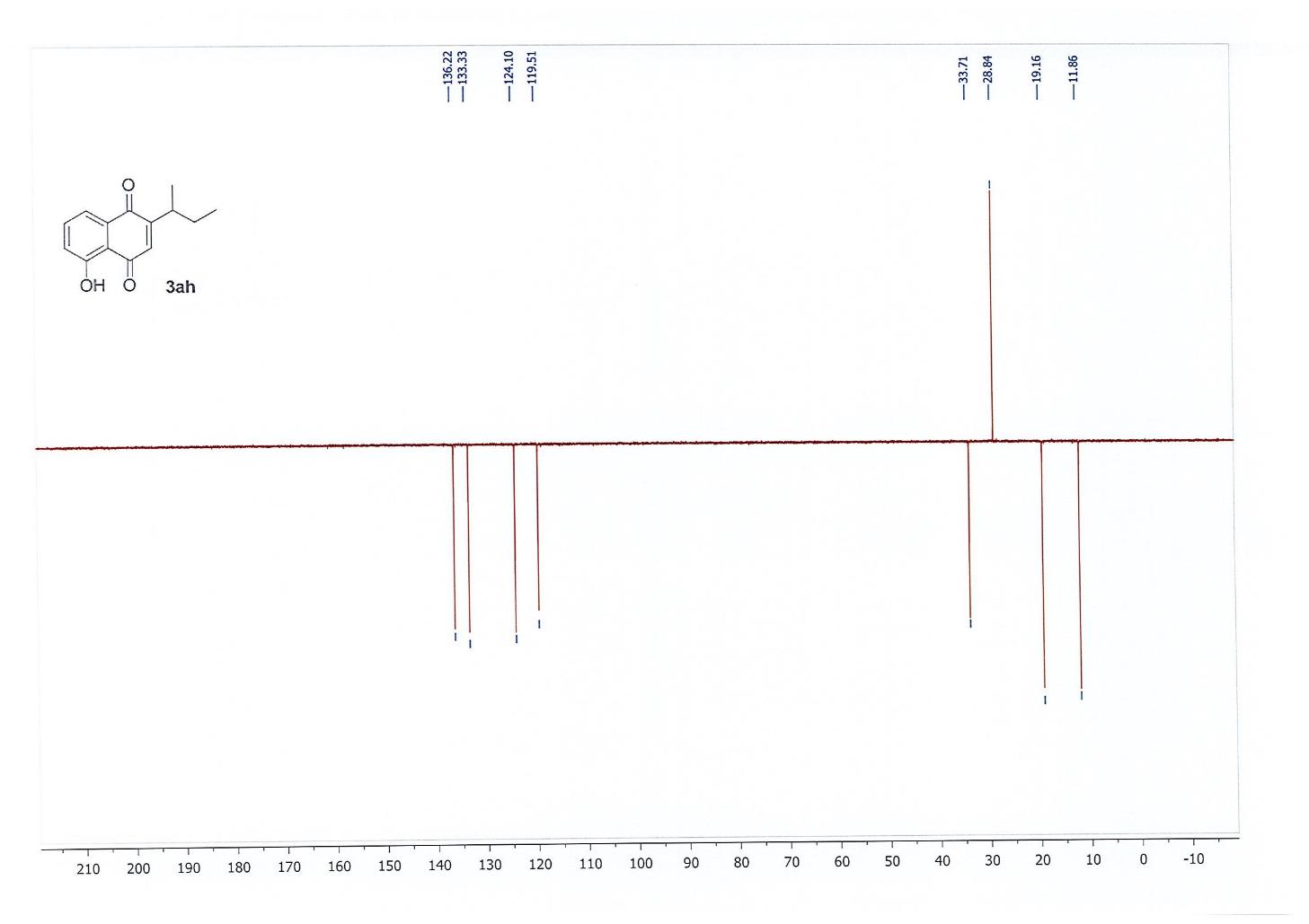


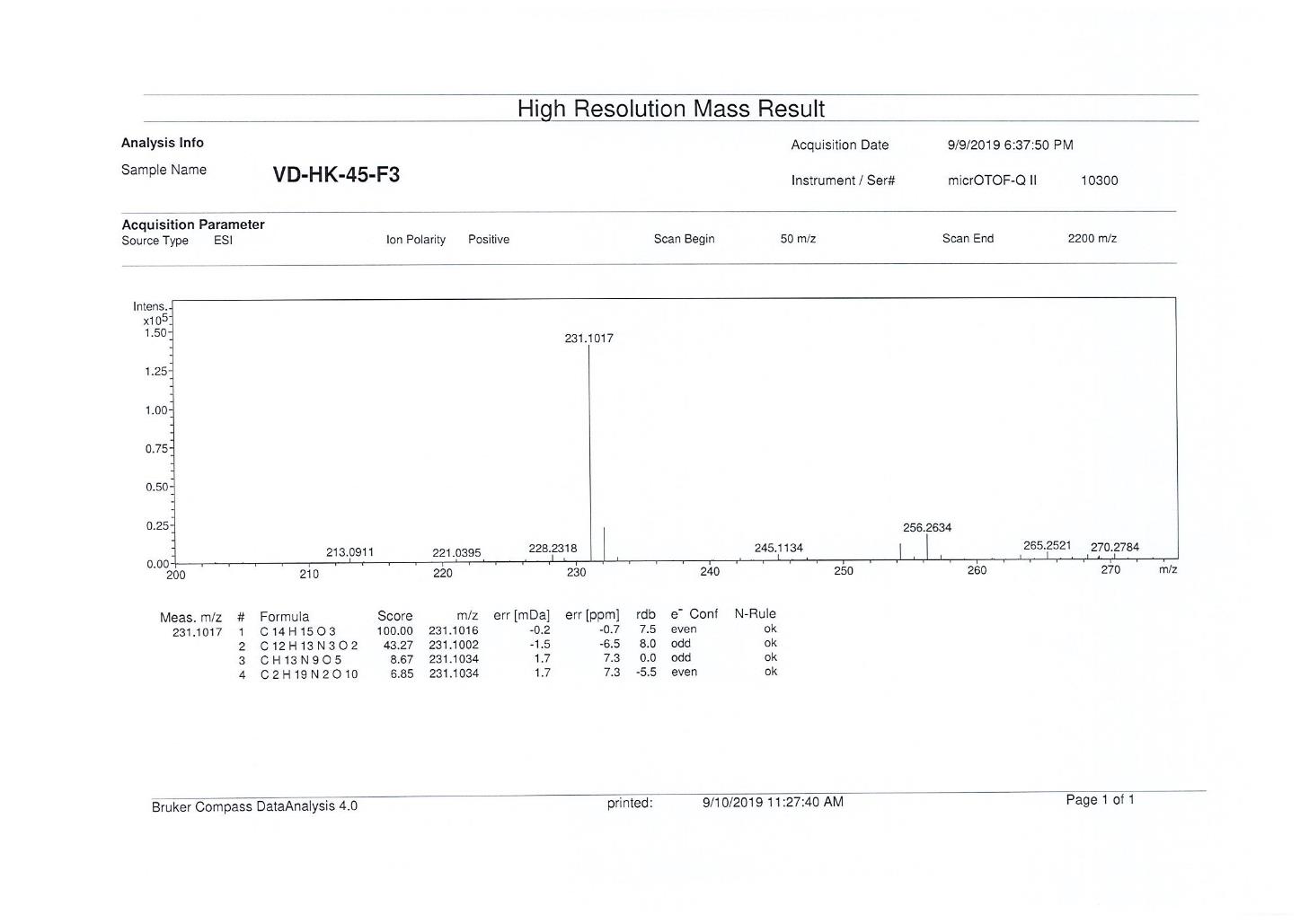


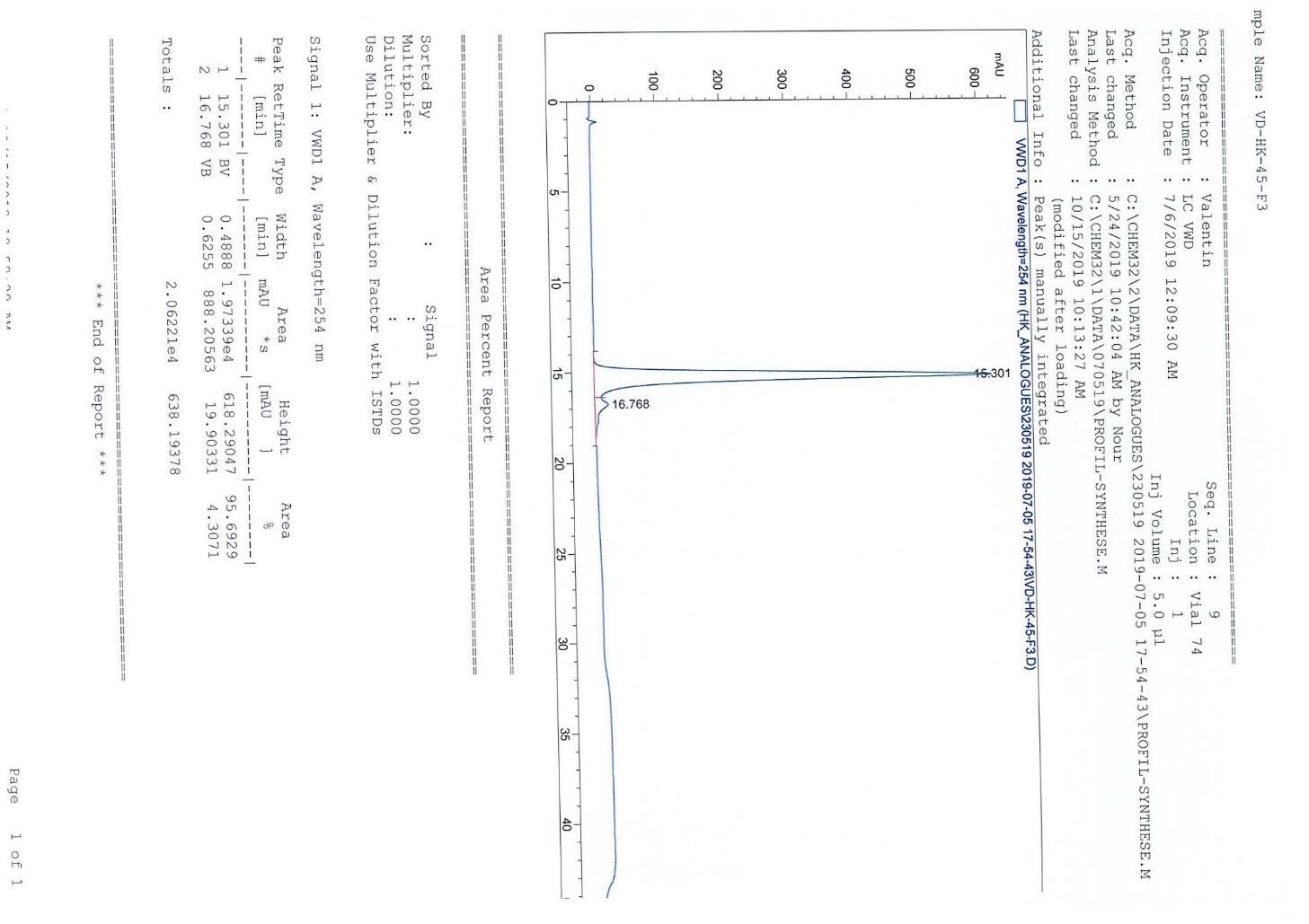


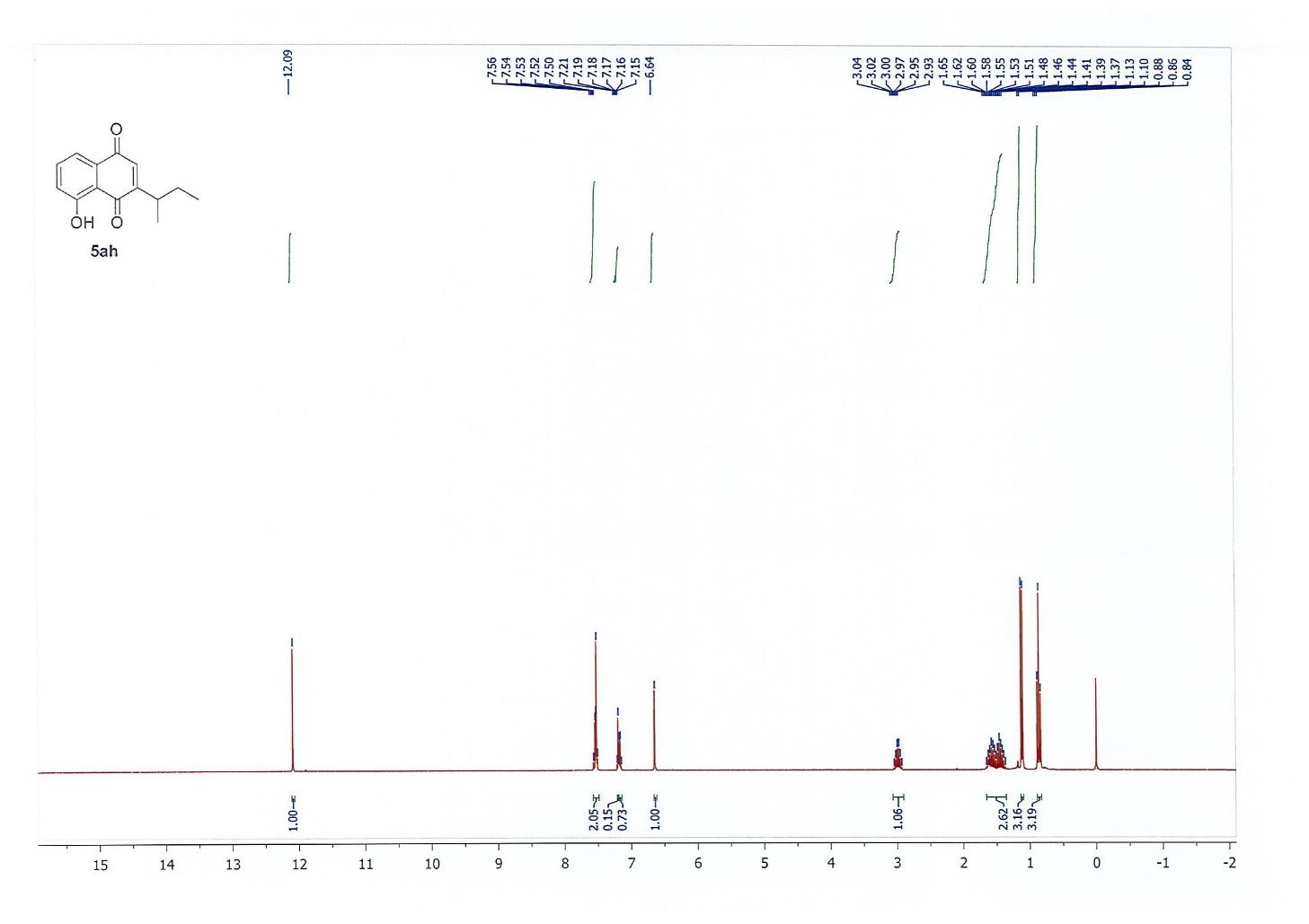


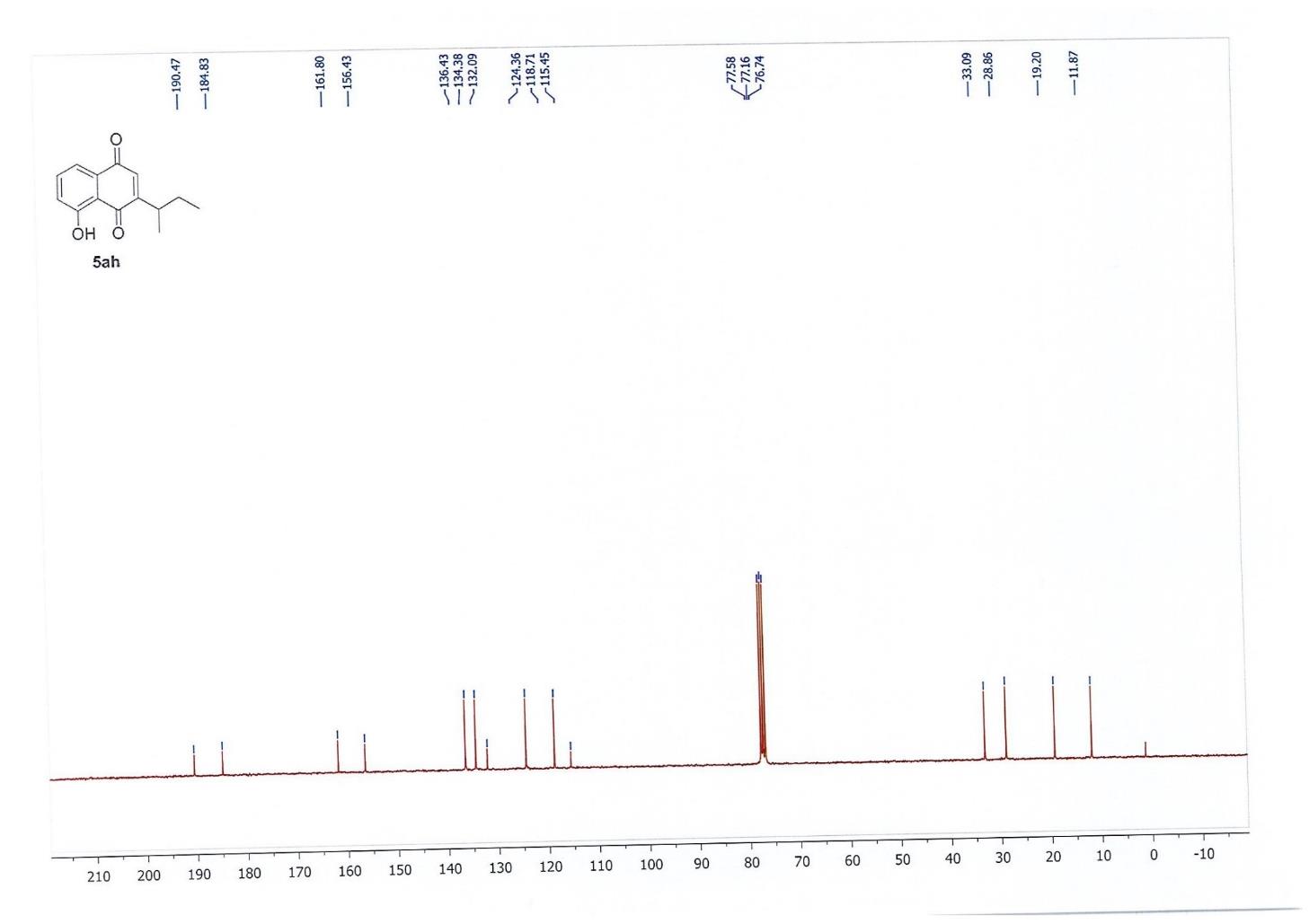


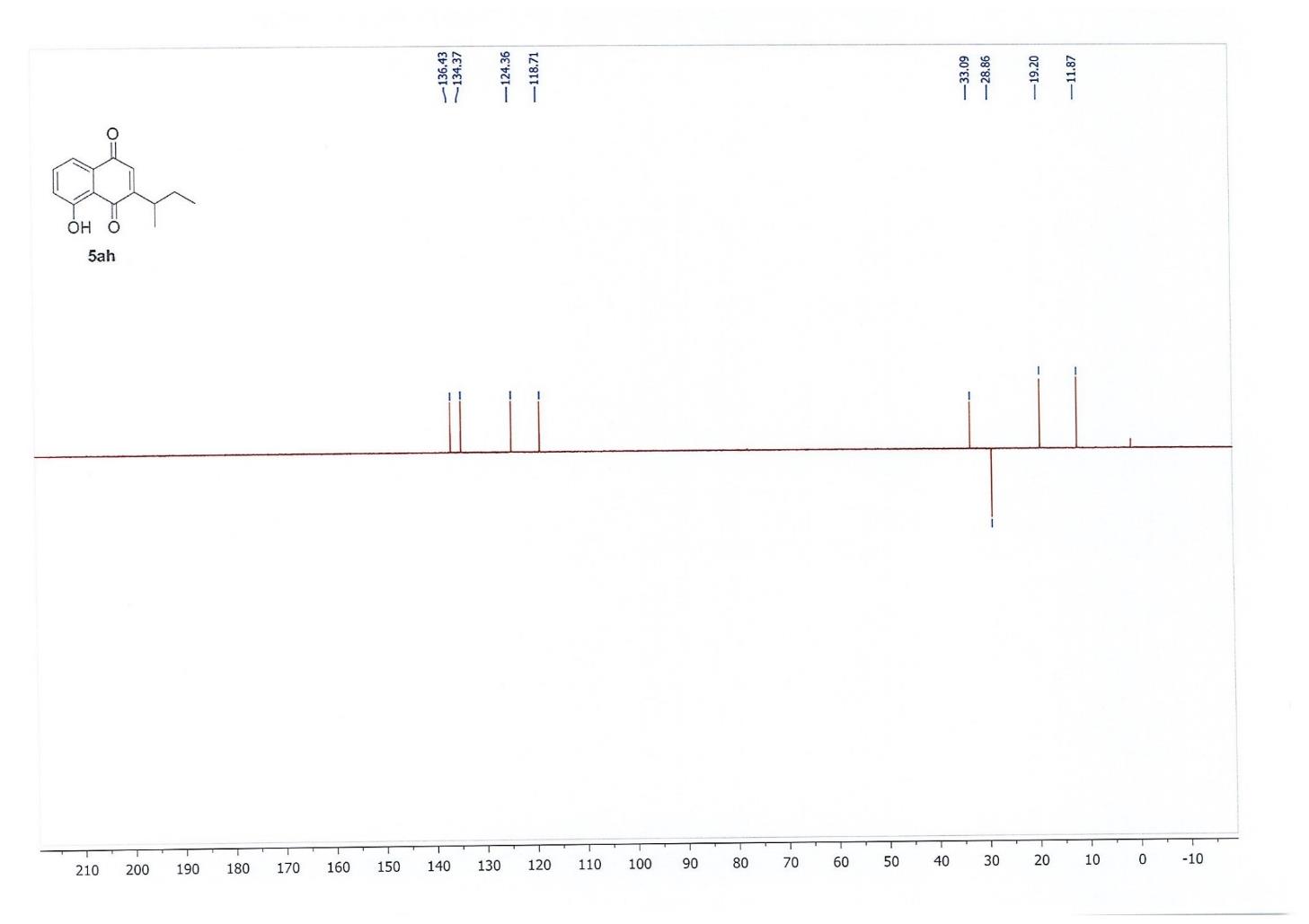


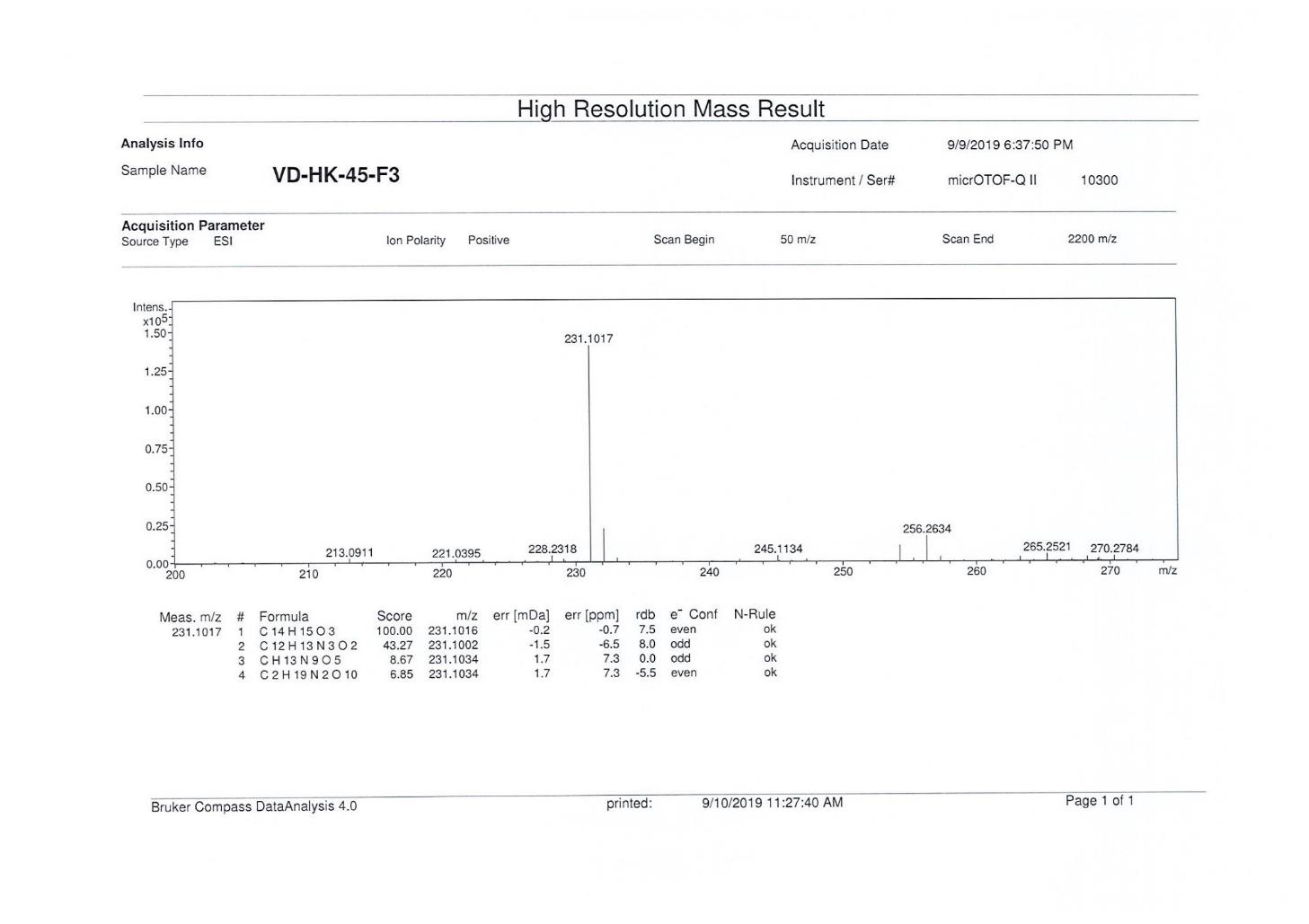


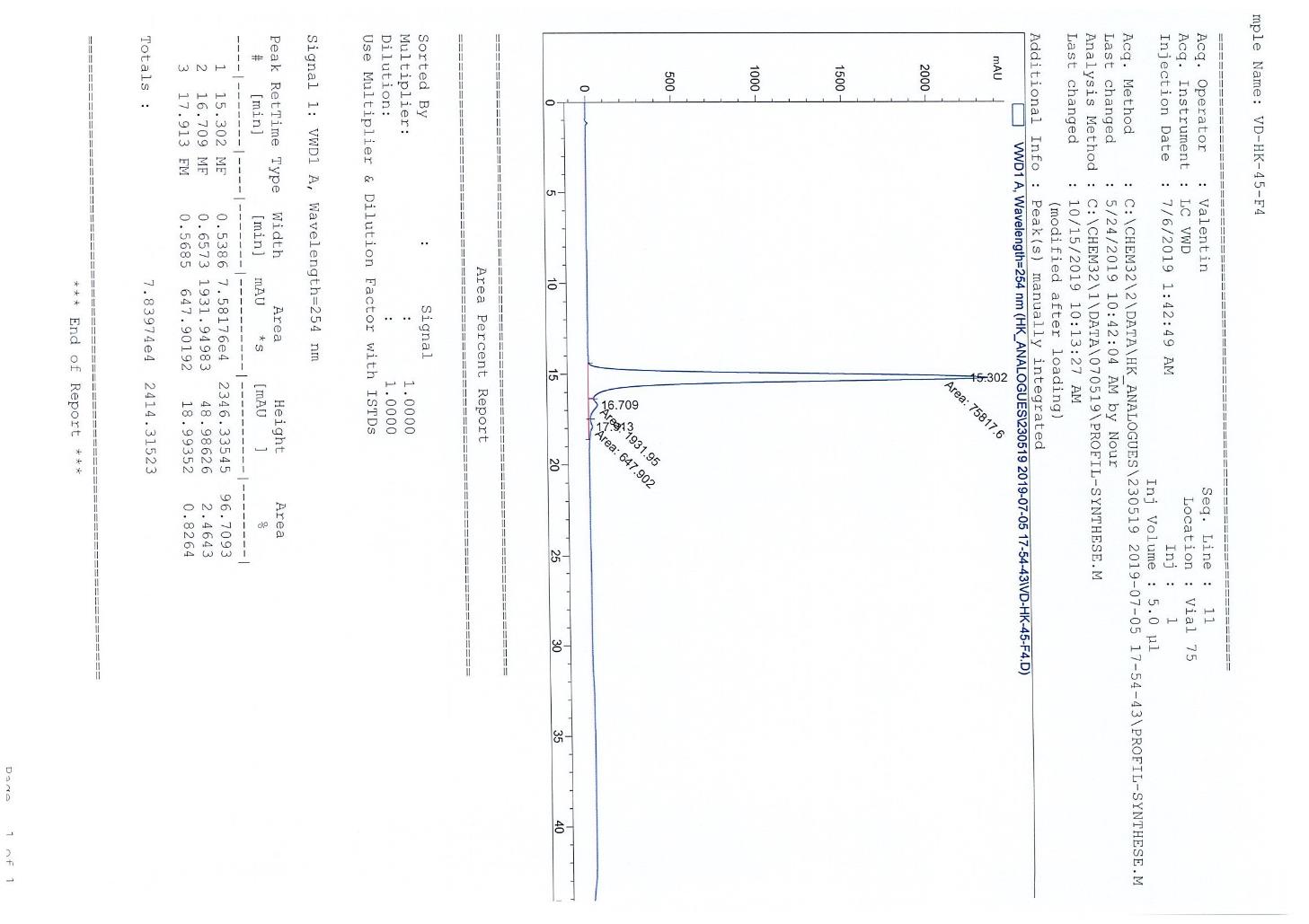


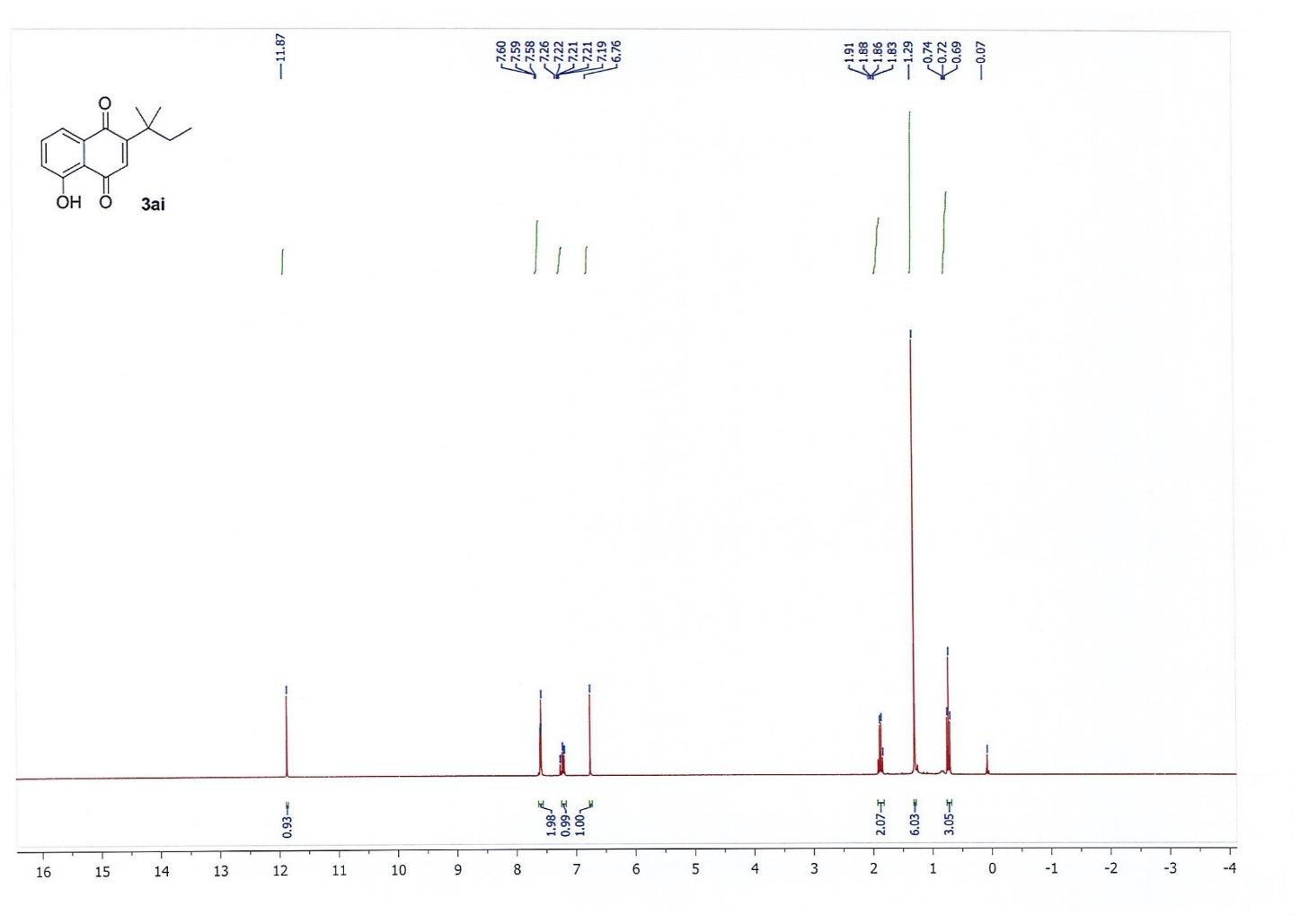


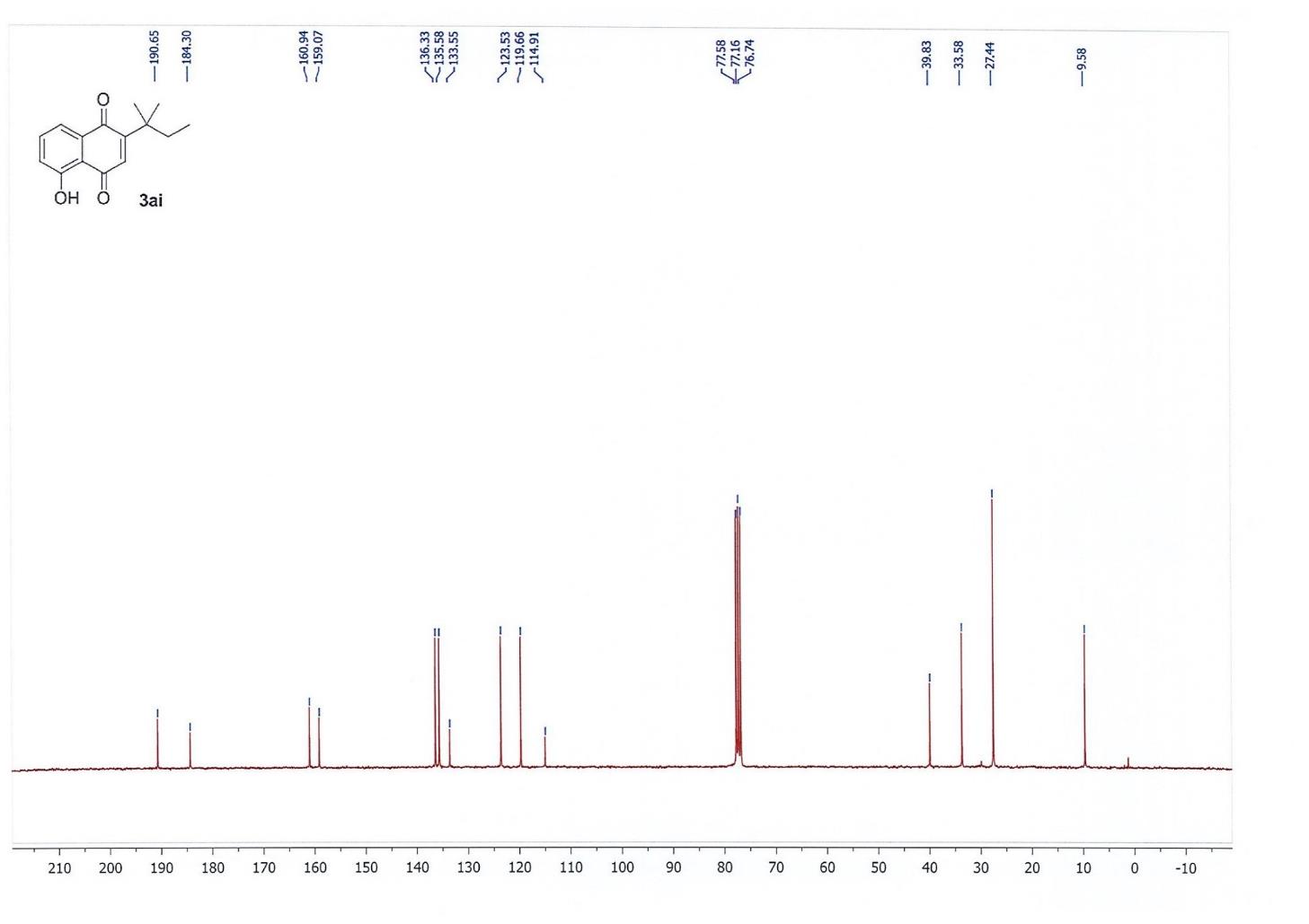


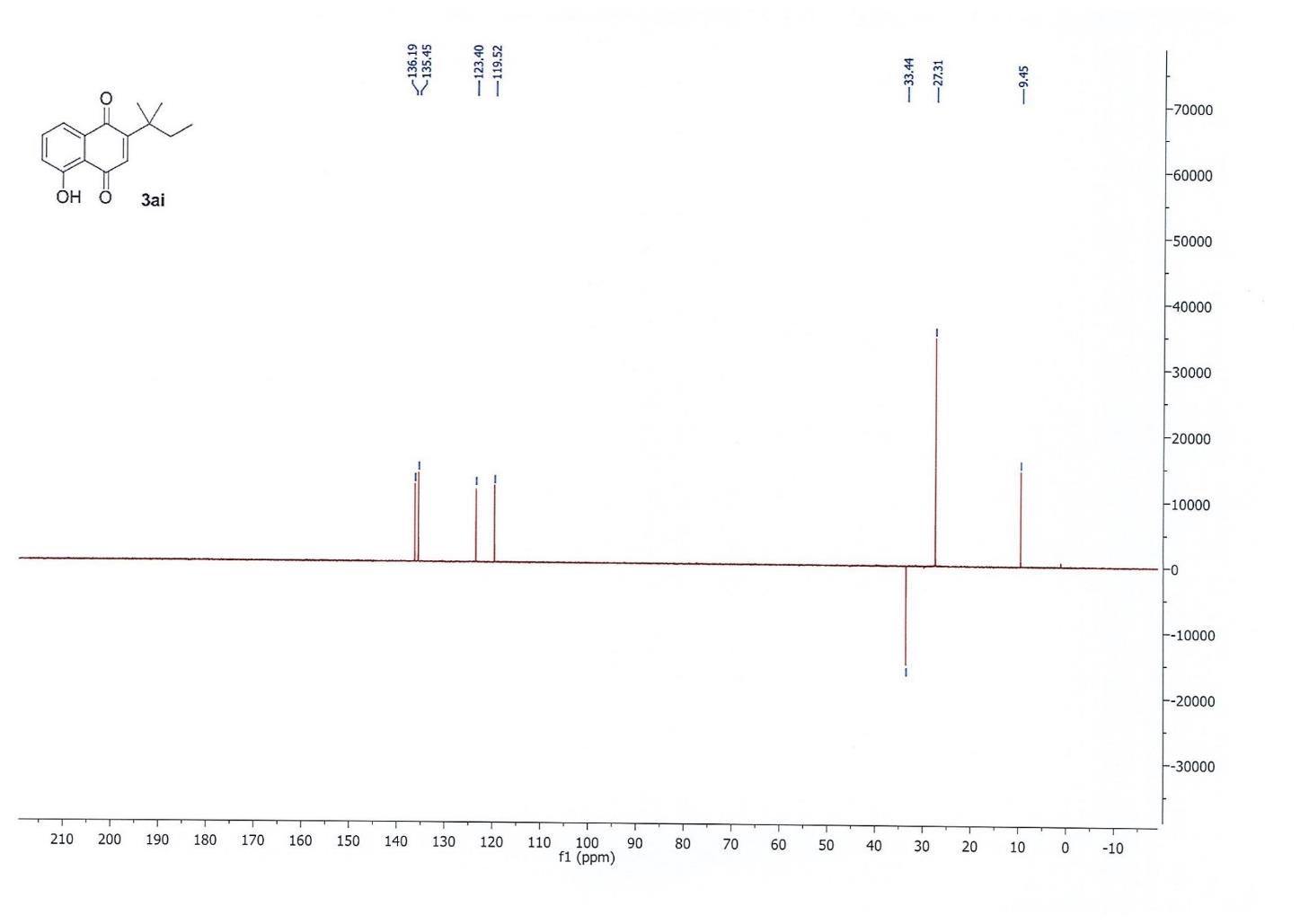

Supplement: Supplementary file 1 [file DataSheet1.docx]
